# A Systematic Study of the Effects of Complex Structure on Aryl Iodide Oxidative Addition at Bipyridyl‐Ligated Gold(I) Centers

**DOI:** 10.1002/anie.202108744

**Published:** 2021-10-18

**Authors:** Jamie A. Cadge, John F. Bower, Christopher A. Russell

**Affiliations:** ^1^ School of Chemistry University of Bristol Cantock's Close Bristol BS8 1TS United Kingdom; ^2^ Department of Chemistry University of Liverpool Crown Street Liverpool L69 7ZD United Kingdom

**Keywords:** anion effects, bipyridyl, gold, ligand effects, oxidative addition

## Abstract

A combined theoretical and experimental approach has been used to study the unusual mechanism of oxidative addition of aryl iodides to [bipyAu(C_2_H_4_)]^+^ complexes. The modular nature of this system allowed a systematic assessment of the effects of complex structure. Computational comparisons between cationic gold and the isolobal (neutral) Pd^0^ and Pt^0^ complexes revealed similar mechanistic features, but with oxidative addition being significantly favored for the group 10 metals. Further differences between Au and Pd were seen in experimental studies: studying reaction rates as a function of electronic and steric properties showed that ligands bearing more electron‐poor functionality increase the rate of oxidative addition; in a complementary way, electron‐rich aryl iodides give faster rates. This divergence in mechanism compared to Pd suggests that Ar−X oxidative addition with Au can underpin a broad range of new or complementary transformations.

## Graphical abstract

An assessment of the effects of complex structure on the oxidative addition mechanism of aryl iodides at bipyridyl‐ligated Au^I^‐centers is described. It is shown that the hemilability of the bipy ligand and the electropositivity of the Au‐center are important factors, leading to facile oxidative addition with electron‐poor ligands and electron‐rich aryl iodides. These unusual observations contrast established Pd‐based systems.

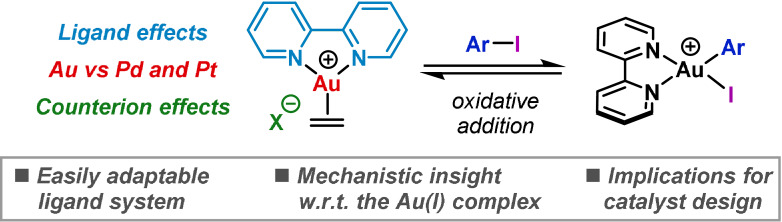

## Introduction

**Scheme 1:**
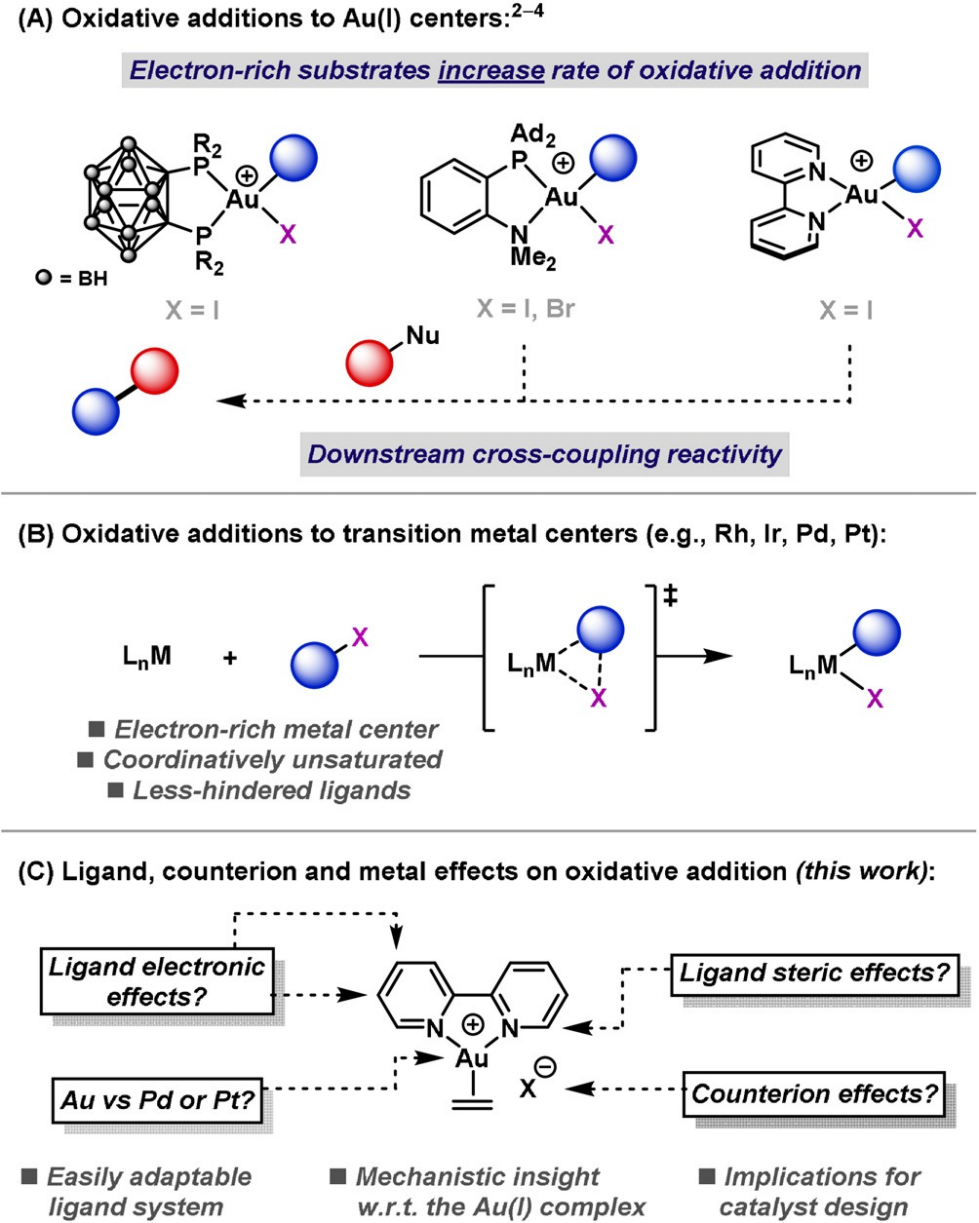
Oxidative addition at transition metal centers.

Ar−X (X=Br, I) oxidative additions involving the Au^I^/Au^III^ redox couple have been developed recently and exploited in the design of redox neutral transformations.^[1]^ Because of the infancy of this area, the mechanistic details of Au^I^‐mediated oxidative addition are still poorly understood. Accordingly, the rational design of more efficient catalyst systems is difficult, and this issue is compounded by the narrow range of suitable ligands, which offer limited scope for modification (Scheme 1 A).[^2^, ^3^, ^4^] Further insight into the requirements for oxidative addition is necessary to enable the design of new ligand architectures and, in turn, the expansion of this area of catalysis. Indeed, for other 2^nd^ and 3^rd^ row transition metals, such as Rh, Ir, Pd and Pt, the qualitative trends, and mechanisms of oxidative addition (and its microscopic reverse, reductive elimination) have been widely studied and are well understood.^[5]^ In these cases, Ar−X oxidative addition proceeds in a concerted manner via three‐centered transition states, and is most efficient at less‐hindered, coordinatively unsaturated and electron‐rich centers (Scheme 1 B). This insight has been instrumental in advancing associated areas of catalysis.

Although the Pd^0^/Pd^II^ and Au^I^/Au^III^ redox couples are isoelectronic, there is a significantly higher barrier associated with the Au^I^ to Au^III^ oxidation (*E*_red_°: Au^III/I^=1.41 V vs. Pd^II/0^=0.92 V).^[6]^ As a result, efficient Ar−X oxidative addition with Au^I^ had, for a long time, been considered “unlikely”,^[7]^ and it is only recently that this dogma has been overturned. Accordingly, established methods that exploit the Au^I^/Au^III^ redox couple have circumvented this issue by instead employing highly reactive external (e.g., hypervalent iodine reagents)^[8]^ or internal oxidants (e.g., diazonium salts).^[9]^ Consequently, substrate availability and utility are compromised in comparison to, for example, Pd‐catalyzed cross‐couplings.

Recent studies have shown that specific bidentate ligands, which possess tight (approx. 90°) bite angles in the κ^2^ mode, can be used to promote efficient Ar−X (X=Br, I) oxidative addition with Au^I^ (Scheme 1 A). Amgoune, Bourissou, and co‐workers demonstrated this activity with a carboranyl diphosphine^[2]^ and with a hemilabile P,N‐system, MeDalPhos,^[3]^ whereas our group showed similar reactivity using commonplace 2,2′‐bipyridyl systems.^[4]^ These fundamental studies have led to the use of the Au^I^/Au^III^ redox couple in an emerging family of catalytic and stoichiometric cross‐couplings.[^3^, ^10^, ^11^, ^12^, ^13^] In particular, MeDalPhos has been employed in Au‐catalyzed C−H arylations,^[3]^ C(sp^2^)−N cross‐couplings^[10]^ and alkene functionalizations.^[11]^ With 2,2′‐bipyridyl ligands, our group demonstrated all elementary steps of a Negishi‐type cross‐coupling at a Au center.^[4]^ An attractive feature of these processes is the rate enhancement of oxidative addition for C(sp^2^)−X substrates bearing electron‐donating substituents—this is the reverse of the trend observed with L_*n*_Pd(0).[^5^, ^14^] Although this observation provides insight into the effects of substrate structure on Ar−X oxidative addition, complementary systematic assessments of the effects of catalyst structure have not been undertaken. Without this information, the rational design of new catalyst systems is challenging, such that advances must rely largely on speculation. This situation is especially unsatisfactory for Au^I^ because the reactivity trends that have already been uncovered are both unique and unexpected.

Herein, we describe fundamental studies on Ar−I oxidative addition with Au^I^ using a number of approaches (Scheme 1 C): i) by in silico comparison of a 2,2′‐bipyridine ligated Au^I^ complex to Pd‐ and Pt‐analogues; ii) by exploiting the adaptability of the bipyridyl unit to investigate how the kinetics of oxidative addition are affected by the electronic and steric parameters of the ligand; iii) by exploring counteranion effects, an aspect that is important in other reactions.[^3a^, ^15^] Our collective observations provide, for the first time, a coherent mechanistic picture of Ar−X oxidative addition from the viewpoint of the Au^I^ complex. Consequently, we hope that these insights will be of wide use in the design of new catalysts and processes.

## Results and Discussion

**Figure 1:**
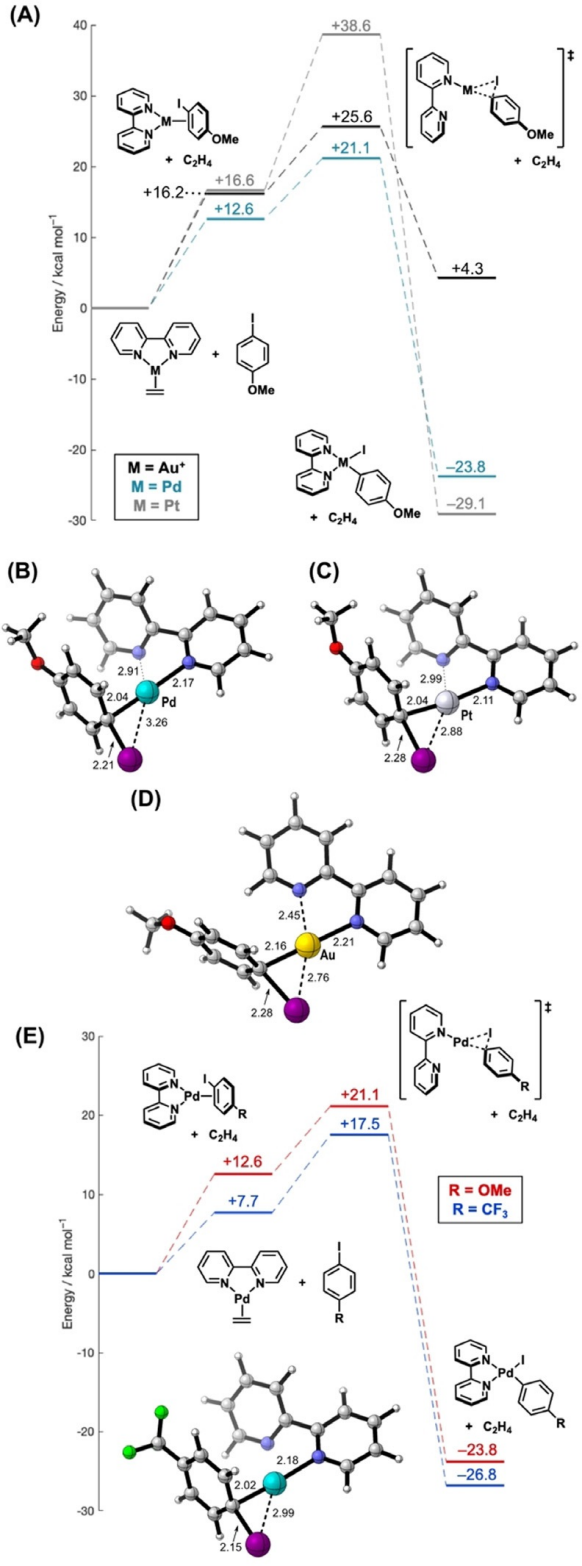
A) Calculated potential energy surface for the oxidative addition of 4‐iodoanisole with theoretical [bipyM(C_2_H_4_)] (M=Pd, Pt) complexes and associated transition state geometries for B) Pd and C) Pt. Oxidative addition with the analogous cationic Au complex is given for comparison.^[4a]^ D) Calculated oxidative addition transition state for Au.^[4a]^ E) Calculated potential energy surface for the oxidative addition of 4‐iodoanisole and 4‐iodobenzotrifluoride with (inset) associated transition state geometry for the latter. Additional potential energy surfaces for R=^*t*^Bu, H and CHO are given in the SI. Calculations were performed using the ωB97‐XD functional, a def2‐TZVP basis set with associated 28‐ and 60‐electron pseudopotentials on Pd and Pt, respectively, def2‐SVP with an associated 28‐electron pseudopotential on I, def2‐SVP on C and N and def2‐SV on all other atoms. The effects of solvation were modelled using the SMD solvation model (CH_2_Cl_2_). Energies shown include zero‐point energy corrections. Bond lengths are quoted in Å.

Initially, we utilized DFT at the ωB97‐XD level of theory to investigate the potential energy (PE) surface for oxidative addition of aryl iodides to [bipyM(C_2_H_4_)] (M=Pd, Pt) with a CH_2_Cl_2_ solvent model (full details are given in the Figure 1 caption). There is sparce experimental data for the Pd‐ and Pt‐complexes; however, these offer an excellent theoretical comparison to the PE surface for the cationic Au‐complex [bipyAu(C_2_H_4_)]^+^, which has already been obtained at the same level of theory,^[4a]^ and allow a direct comparison of oxidative addition mechanisms. Starting from the corresponding ethylene complexes, initial displacement with the aryl iodide leads to the corresponding η^2^‐π‐bound complexes. Side‐on metal to C−I contacts then develop prior to oxidation addition. Strikingly, the thermodynamics of the reactions differ significantly, such that the reactions of the Pd and Pt complexes (Δ*E*=−23.8 kcal mol^−1^ and −29.1 kcal mol^−1^ for Pd and Pt, respectively) are exothermic whereas the Au complex is endothermic (Δ*E*=+4.3 kcal mol^−1^).^[16]^ These differences show that oxidative addition of both the Pd and Pt complexes is more thermodynamically favorable compared to the Au analogue. For the latter, our previous experimental studies have shown that the oxidative addition is reversible.^[4]^ Conversely, Bourissou, Amgoune and co‐workers showed that the P,N‐ligand MeDalPhos provides a thermodynamically favorable oxidative addition.^[3]^ The transition state geometries for the Pd and Pt complexes (Figure 1 B,C) show an approximately linear N−M−C_*ipso*_ vector (approx. 175°) indicating an early transition state as observed with Au. C−X bond insertion at Pt^0^ centers is generally slower than for equivalent Pd^0^ complexes, and this agrees with the larger computed activation barrier seen here.^[17]^ Comparison of the transition state geometries (Figure 1 B–D) revealed marginally shorter C_*ipso*_−M bond lengths for Pd and Pt (2.04 Å) than for Au (2.16 Å).^[4a]^ In all cases, the C_*ipso*_−I bond is lengthened (2.21–2.28 Å) versus the free aryl iodide (approx. 2.11 Å). Significantly, compared to Au, the M−I bond in the Pd and Pt transition states is elongated, with this effect being most pronounced for Pd (Pd: 3.26 Å vs. Pt: 2.88 Å vs. Au: 2.76 Å). These observations are consistent with the higher electropositivity of the Au‐center facilitating electron donation via the iodine center, which results in a shorter Au−I bond.^[18]^ Amgoune, Bourissou and co‐workers have disclosed similar findings with the hemilabile MeDalPhos ligand; in their study, charge analysis of the transition states provided further insight.^[3b]^ All three transition states show increased M−N bond lengths for one of the pyridyl units of the bipy ligand. This effect is most pronounced for the Pd and Pt complexes (Pd: 2.91 Å vs. Pt: 2.99 Å vs. Au: 2.45 Å), where the pyridyl unit is rotated by approximately 50° to give a three‐coordinate center. This aligns with previous computational studies with Pd, where the barrier for oxidative addition decreases at lower coordination numbers.[^14c^, ^19^] Conversely, for the Au‐complex, the pyridyl unit is associated with the metal center, giving a four‐coordinate transition state. Here, bidentate coordination of the bipy ligand is likely required to facilitate bending and allow oxidative addition.^[6b]^

Next, the effects of aryl iodide electronics on oxidative additions with bipy‐ligated Pd and Au complexes were compared. For [bipyAu(C_2_H_4_)]^+^, oxidative addition with 4‐iodobenzotrifluoride has a higher barrier than with 4‐iodoanisole (Δ*E*=27.7 kcal mol^−1^ vs. 25.6 kcal mol^−1^).^[4a]^ For [bipyPd(C_2_H_4_)], the opposite trend is evident (Δ*E*=17.5 kcal mol^−1^ vs. 21.1 kcal mol^−1^), wherein oxidative addition of the more electron‐poor aryl iodide is more facile (Figure 1 E). The Pd−C_*ipso*_ and C_*ipso*_−I bonds are of similar length in the oxidative addition transition state structures for both 4‐iodoanisole and 4‐iodobenzotrifluoride. The Pd−I bond, however, is shorter for the latter, which is consistent with enhanced Pd→σ* donation;^[18]^ similar trends have been calculated for phosphine‐ligated Pd^0^ systems.^[14c]^ By contrast, for the Au‐complex, the most pronounced difference in transition state structure is associated with the Au−C_*ipso*_ bond length, which is shorter for 4‐iodoanisole (2.16 Å vs. 2.23 Å for 4‐iodobenzotrifluoride). This is indicative of a dominant C_*ipso*_→Au interaction during oxidative addition.^[4a]^

The calculations described so far support the notion that electron donation from the C_*ipso*_−I unit of the aryl iodide to the Au‐center is a key factor in facilitating oxidative addition. This contrasts Pd‐based systems and, in turn, suggests that ligand effects might be distinct for Au‐based oxidative additions. Accordingly, studies were undertaken to assess the electronic effects of the 2,2′‐bipyridyl ligand on both complex structure and oxidative addition kinetics. This approach is advantageous because many bipyridyl derivatives are either commercially available or can be readily prepared.[^20^, ^21^]

**Figure 2:**
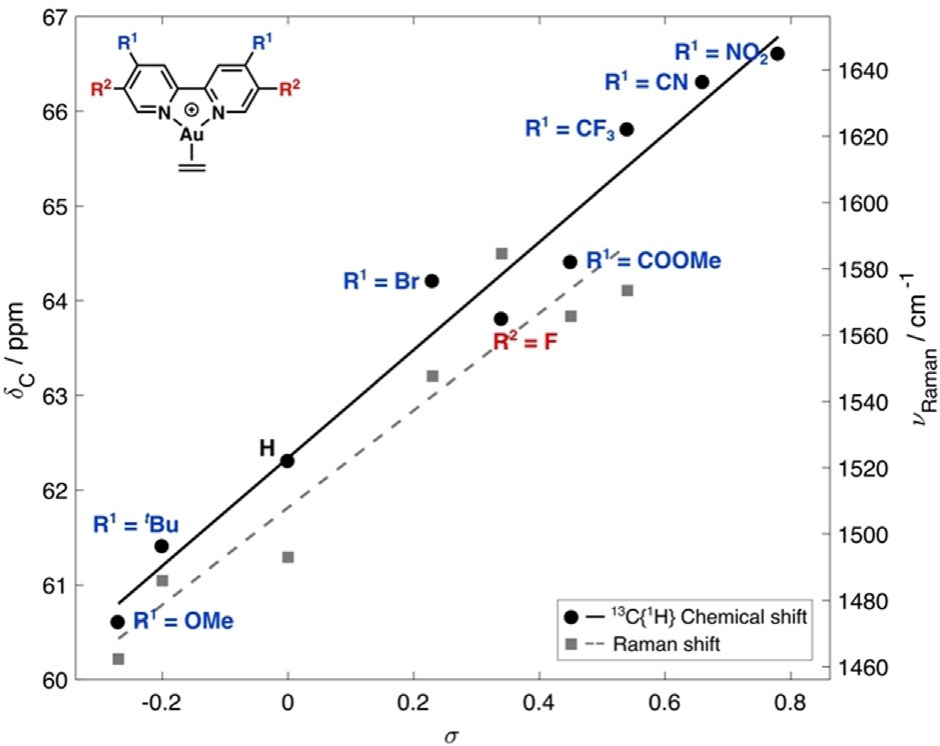
Linear relationships between ethylene ^13^C NMR chemical shift and Raman shift with *σ*.

**Scheme 2:**
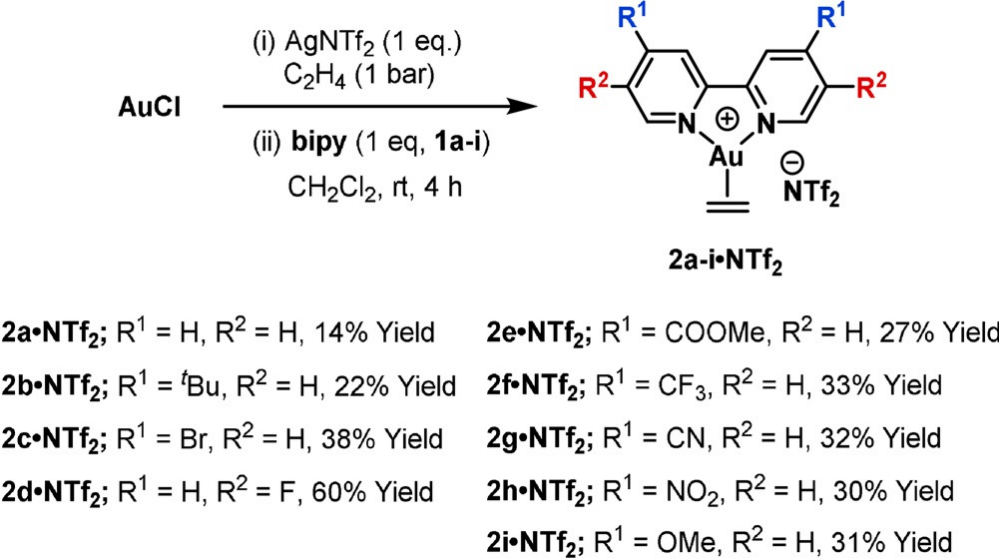
Synthesis of Au^I^ 2,2′‐bipyridyl ethylene complexes **2 a**–**i**.

Complexes [(R_2_‐bipy)Au(η^2^‐C_2_H_4_)]NTf_2_ (**2 a**–**i⋅NTf_2_**) were accessed in 14–60 % yield by direct reaction of the 2,2′‐bipyridyl ligand (R_2_‐bipy, **1 a**–**i**) with freshly prepared [(Au(C_2_H_4_)_3_]NTf_2_ in CH_2_Cl_2_ (Scheme 2). Examination of the ^13^C NMR^[14b]^ chemical shifts (*δ*_C_) and the Raman frequencies (*ν*_Raman_) of the ethylene ligand of **2 a**–**i⋅NTf_2_** indicated that substitution at the 4/4′‐ and 5/5′‐positions has a significant impact on the degree of back‐bonding. Correlation of these spectroscopic datasets with the Hammett electronic parameters (σ)[^22^, ^23^] revealed a linear relationship, indicating that σ is an appropriate electronic descriptor (Figure 2). Bipyridyl ligands with electron‐donating substituents (e.g., R^1^=OMe) show a higher degree of back‐donation to ethylene, whereas ligands with electron‐withdrawing groups (e.g., R^1^=NO_2_) display a Raman spectroscopic signature tending towards that of free ethylene ($\tilde{\nu }$_Raman_=1623 cm^−1^). These data correlate with the qualitative observation that complexes with electron‐poor bipyridyl ligands are less stable in CH_2_Cl_2_ solution than their electron‐rich counterparts. This effect was particularly apparent with bipyridyl ligands bearing the 4‐CN (**2 g⋅NTf_2_**) and 4‐NO_2_ (**2 h⋅NTf_2_**) substituents, where, after only a few minutes, the solution turned an intense purple color, which we attribute to the formation of Au nanoparticles. Collectively, these observations show that more electron‐rich bipyridyl ligands provide greater electron density at the Au^I^ center and facilitate back‐bonding. Similar stabilization trends for Au^I^−(η^2^‐C_2_H_4_) binding have been observed by Dias and co‐workers using electron‐rich scorpionate ligands.^[24]^ It is also pertinent to note that Gatineau, Gimbert and co‐workers analyzed the dissociation of CO from LAu−CO complexes {L=phosphine or N‐heterocyclic carbene (NHC)} by mass spectrometry.^[25]^ In these studies, strongly σ‐donating NHC ligands stabilized the Au−CO bond more efficiently than weaker P‐based donors.

**Figure 3:**
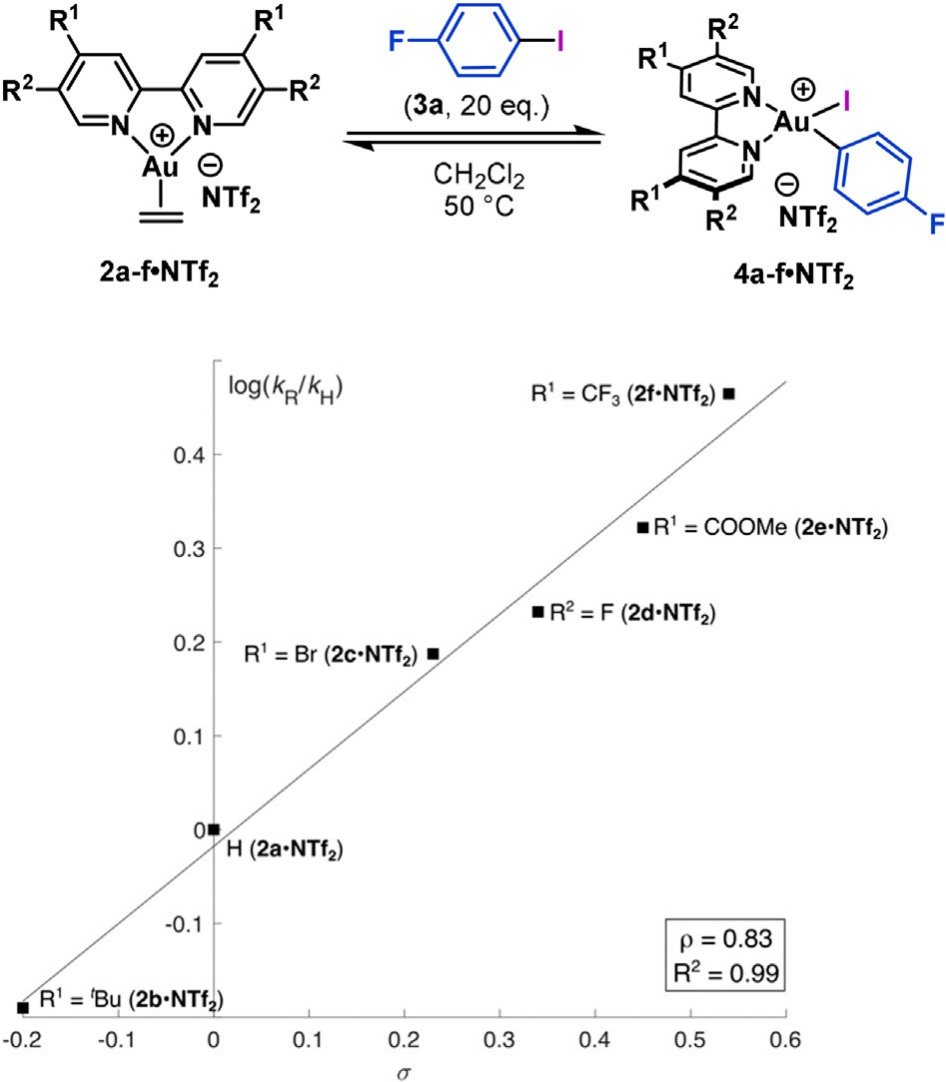
Hammett plot for the oxidative addition of 4‐fluoroiodobenzene to Au^I^ ethylene complexes **2 a**–**f⋅NTf_2_**.

The influence of ligand electronics on the rates of Ar−I oxidative addition to complexes **2 a**–**f⋅NTf_2_** and **2 i⋅NTf_2_** was investigated next. Initial rates for the addition of (excess) 4‐fluoroiodobenzene (**3 a**) to complexes **2 b**–**f⋅NTf_2_** were determined and compared to data for the parent bipyridine complex **2 a⋅NTf_2_** (Figure 3).^[26]^ The associated Hammett plot revealed a linear correlation with *ρ*=0.83, suggestive of a small electronic effect where electron‐poor bipyridyl ligands give an increased rate. At one extreme, the rate of oxidative addition with the highly electron‐rich 4‐OMe‐substituted bipyridyl ligand (**2 i⋅NTf_2_**) was too slow to get any meaningful rate data by ^19^F NMR spectroscopy. Analysis of the reaction mixture by mass spectrometry (ESI^+^) showed a signal for the oxidative addition product at *m*/*z* 634.9883 (calcd 634.9906, [M−NTf_2_]^+^) indicating that oxidative addition, although slow, is indeed feasible. The electronic preferences of the process are interesting because they are the inverse of oxidative additions with L_*n*_Pd^0^ complexes. For example, a kinetic study with differently *p*‐substituted triarylphosphine‐ligated Pd^0^ complexes gave *ρ*=−2.8.^[27]^ Bipy‐like ligands (e.g., phen) have received attention for their role in enabling Au^I^/Au^III^ catalysis with hypervalent iodine(III) reagents.^[28]^ Importantly, the results described here are distinct; for example, Hashmi and co‐workers showed that the rate of oxidative addition of alkynyl−iodine(III) reagents to [(phen)AuPR_3_]NTf_2_ complexes does not have a linear relationship with the electronics of substituted phen ligands.^[29]^ Instead, a strong linear correlation to the electronics of the PR_3_ ligand was observed, with more weakly donating variants being most efficient (*ρ*=3.75). This trend was rationalized on the basis that PR_3_ ligands with a smaller *trans* influence facilitate access to tri‐ or tetra‐coordinated Au^I^‐complexes involved in the oxidative addition pathway. In the current work, similar but smaller electronic effects are observed by a linear free energy relationship directly associated with the bidentate bipyridyl ligand framework.

**Figure 4:**
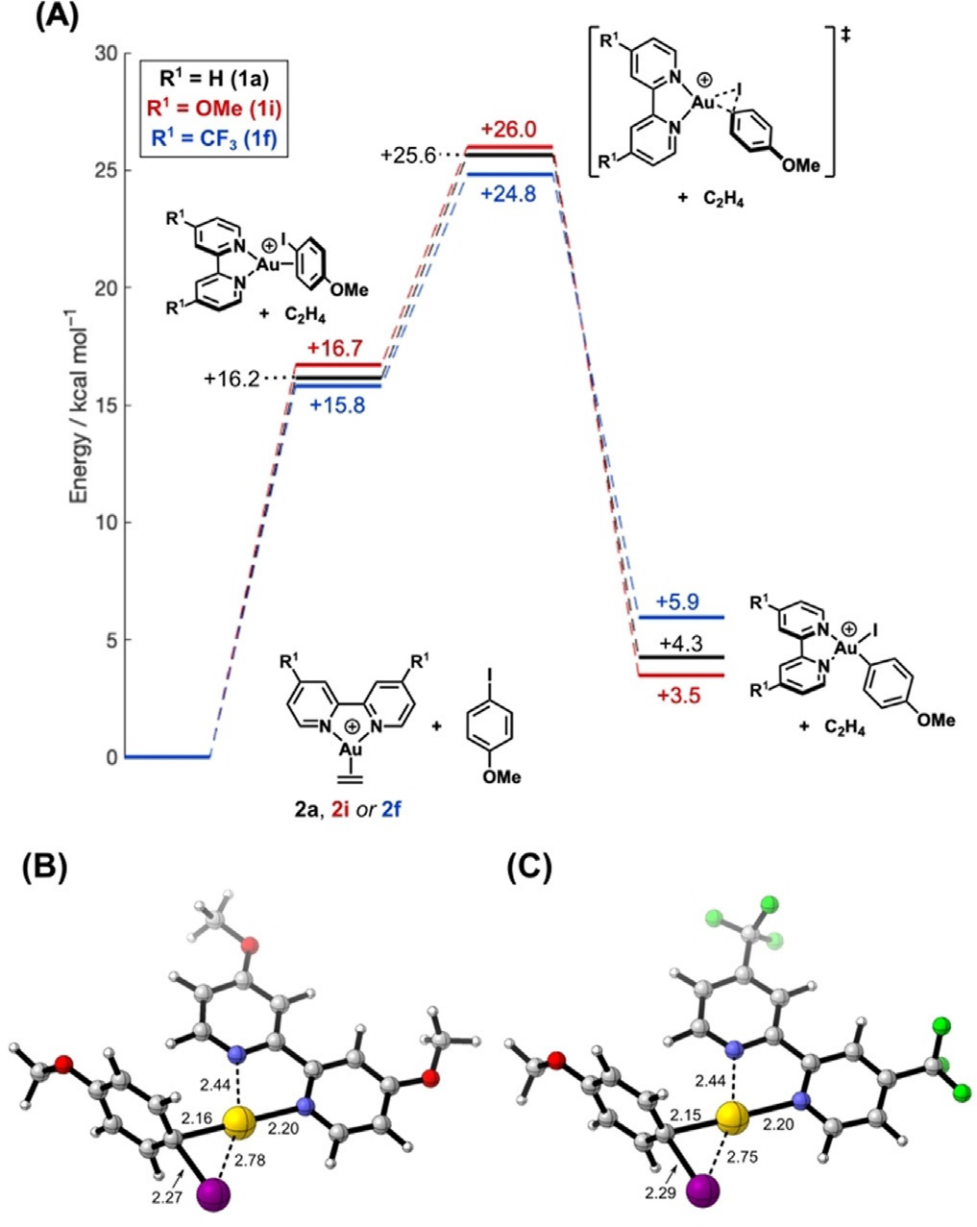
A) Calculated potential energy surface for the oxidative addition of 4‐iodoanisole with ligands **1 a**, **1 f** and **1 i** and associated transition state geometries with B) **1 i** and C) **1 f** and selected bond lengths (Å). Additional potential energy surfaces for R^1^=^*t*^Bu and CO_2_Me are given in the SI. Calculations were performed using the ωB97‐XD functional, a def2‐TZVP basis set with an associated 60‐election pseudopotential on Au, def2‐SVP with an associated 28‐electron pseudopotential on I, def2‐SVP on C and N and def2‐SV on all other atoms. The effects of solvation were modelled using the SMD solvation model (CH_2_Cl_2_). Energies shown include zero‐point energy corrections.

The unusual effect of ligand electronics on the facility of oxidative addition was examined computationally using DFT (Figure 4). R^1^=OMe (**1 i**) and R^1^=CF_3_ (**1 f**) were chosen as representative electron‐donating and electron‐withdrawing substituents on R_2_‐bipy (i.e., giving Au^I^ cations **2 i** and **2 f**), and 4‐iodoanisole (**3 c**) was selected as a representative aryl iodide. Following similar PE surfaces as in Figure 1, the electron‐rich OMe‐substituted ligand **1 i** gives a more exothermic oxidative addition (+3.5 kcal mol^−1^) than the electron‐poor CF_3_‐substituted ligand **1 f** (+5.9 kcal mol^−1^, Figure 4 A). The differences in energies of the η^2^‐π‐arene intermediates and ensuing transition states are small, but display trends consistent with the synthetic data, with the electron‐poor ligand giving a lower energy transition state (CF_3_: +24.8 kcal mol^−1^ vs. OMe: +26.0 kcal mol^−1^). The transition state geometries (Figure 4 B,C) show similar structural features to that calculated for complex **2 a**, which bears the parent bipy ligand **1 a** (Figure 1 D). Minor differences, such as the Au−I bond length {**2 i** (R=OMe): 2.78 Å vs. **2 a** (R=H): 2.76 Å vs. **2 f** (R=CF_3_): 2.75 Å}, correlate with the small differences in experimental rate data and the small *ρ* value (see SI).

**Figure 5:**
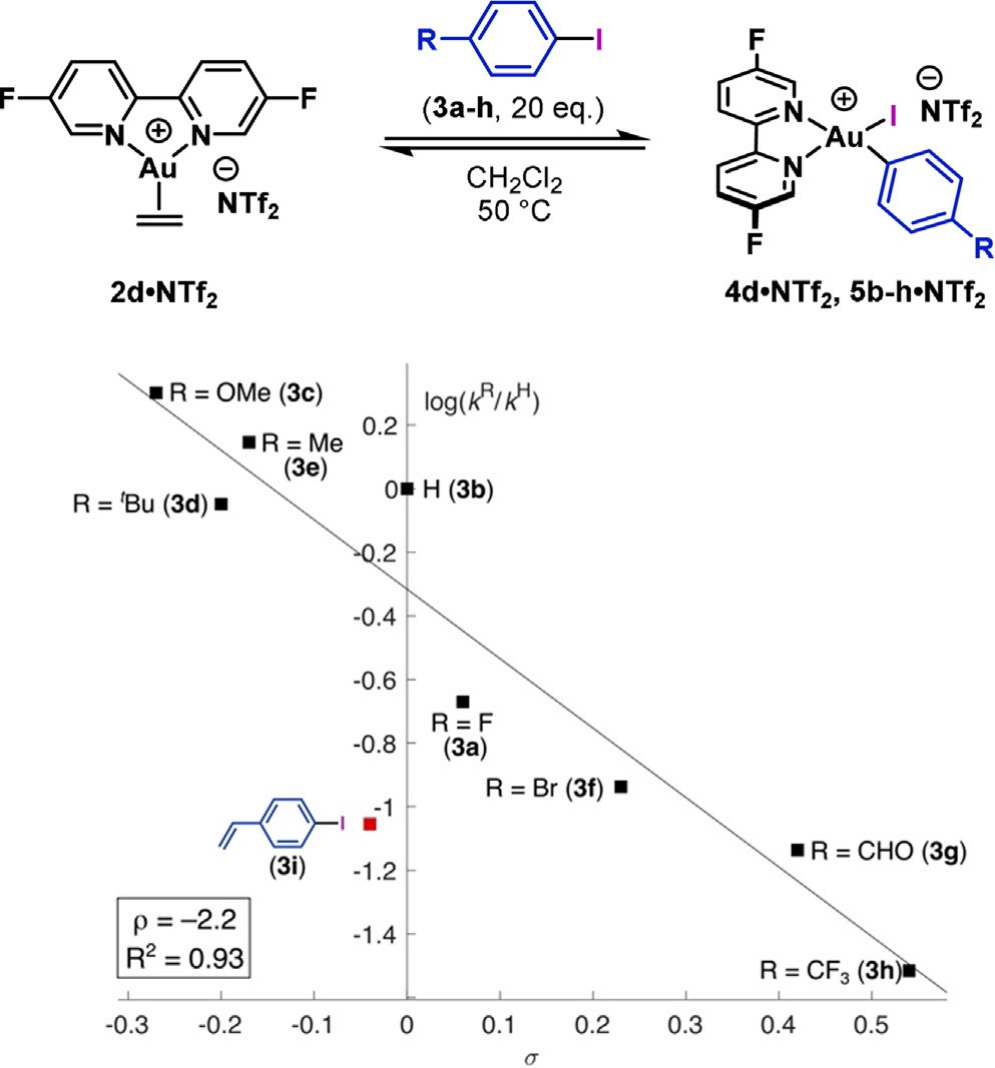
Hammett plot for the oxidative addition of *p*‐substituted aryl iodides (**3 a**–**i**) with varying electronics.

Using F_2_‐bipy system **2 d⋅NTf_2_**, an equivalent Hammett analysis was performed by varying the 4‐substituent on the aryl iodide (Figure 5). In line with our previously reported DFT studies,^[4a]^ a larger absolute reaction constant (*ρ*=−2.2) was observed, which shows that substitution on the aryl iodide has a more significant effect than on the bipyridyl ligand. The reaction constant is also more negative than that reported by Amgoune, Bourissou and co‐workers for a MeDalPhos‐ligated Au center (*ρ*=−1.1)^[3b]^ and of similar magnitude to oxidative additions at phosphine‐ (*ρ*=2.3)^[14a]^ and methyl imidazole‐ligated (*ρ*=1.5)^[14b]^ Pd^0^‐centers. The larger absolute reaction constant determined for **2 d⋅NTf_2_** is consistent with the poor donor properties of the bipy ligand enhancing the electropositivity of the Au^I^ center.

**Scheme 3:**
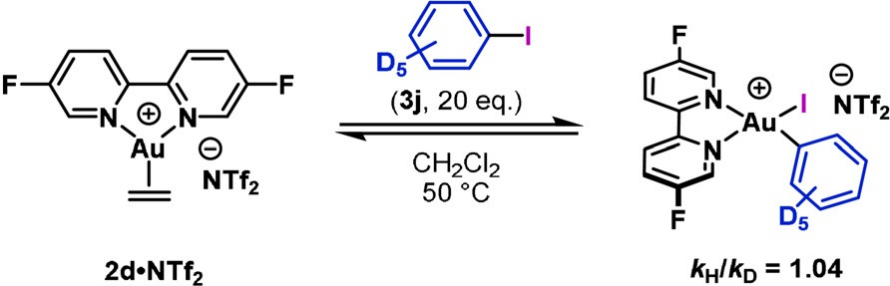
Kinetic isotope effect experiment with **2 d⋅NTf_2_** and iodobenzene‐*d*_5_ (**3 h**).

Further observations have provided insights into the effect of π‐donation in the pre‐oxidative addition complexes shown in Figure 4. With 4‐vinyliodobenzene (**3 i**), a lower‐than‐expected rate of oxidative addition was observed (Figure 5). This result suggests that π‐binding of the vinyl unit to the Au^I^ center competes with η^2^‐π‐binding to the arene, thereby suppressing the rate of oxidative addition. This hypothesis was verified computationally (see SI). Similarly, Patil and co‐workers have shown that alkenes decrease the rate of oxidative addition of aryl iodides using MeDalPhos as the ligand.^[11d]^ Comparison of the rates of oxidative addition of iodobenzene (**3 b**) and iodobenzene‐*d*_5_ (**3 j**) to **2 d⋅NTf_2_** revealed that secondary kinetic isotope effects are minimal (*k*_H_/*k*_D_=1.04) (Scheme 3). Overall, these observations indicate that π‐binding of the arene is an important feature of the mechanism of oxidative addition, but is not rate‐limiting. This mirrors oxidative additions to Pd^0^, where arene π‐binding is reversible, but contrasts examples involving Ni, where arene π‐binding can be the first irreversible step.^[25]^ Additionally, a recent study from our group on oxidative additions of alkynyl and alkenyl iodides to Au^I^ centers showed that π‐binding is a key feature, and this was most apparent for more electron‐rich C−C multiple bonds.^[4b]^

**Scheme 4:**
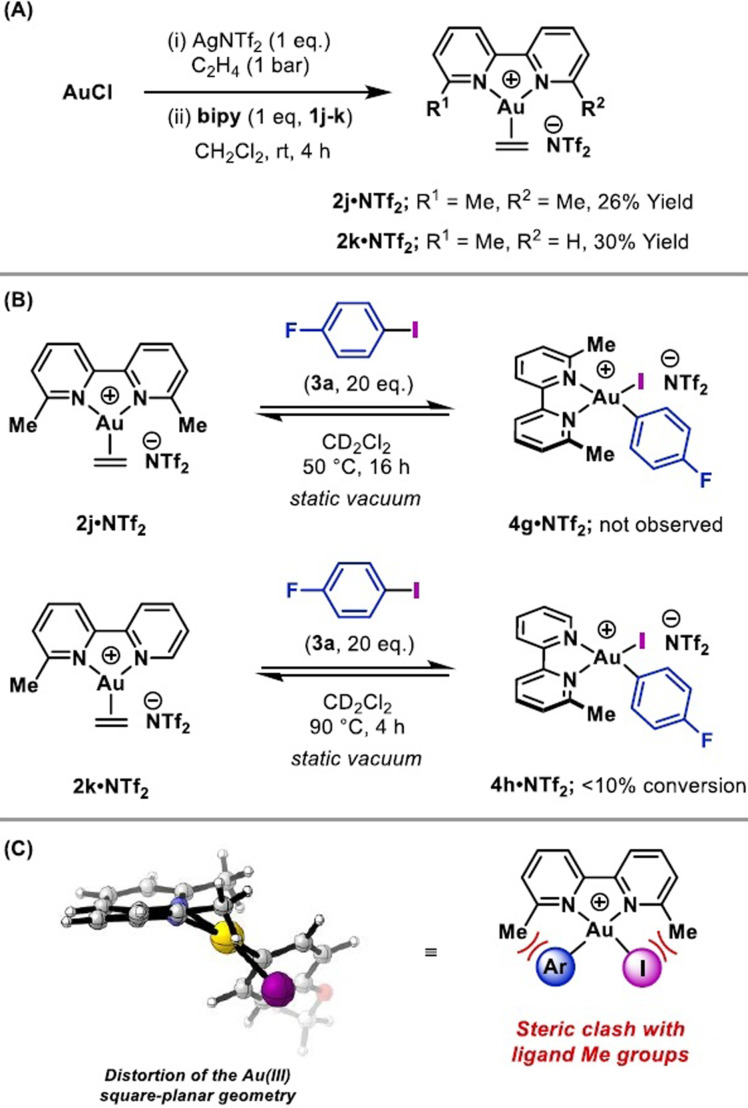
A) Synthesis of Au^I^ ethylene complexes with methyl groups at the 6‐ and/or 6′‐positions, B) attempted oxidative addition of 4‐fluoroiodobenzene and C) calculated minimum energy geometry of the oxidative addition product. For calculation details, see Figure 4 caption.

The studies outlined so far delineate the electronic effects of the bipyridyl framework on oxidative addition. Steric effects have also been investigated by placing substituents at the 6‐ and/or 6′‐positions of the 2,2′‐bipyridyl framework. Di‐ and mono‐methylated complexes **2 j⋅NTf_2_** and **2 k⋅NTf_2_** were synthesized in 26 % and 30 % yield, respectively, using the method outlined earlier (Scheme 4 A). Exposure of Au^I^ complex **2 j⋅NTf_2_** to 4‐fluoroiodobenzene (**3 a**) at 50 °C for 16 hours under static vacuum resulted in no reaction, as evidenced by ^1^H and ^19^F NMR spectroscopy (Scheme 4 B).^[30]^ Increasing the temperature and duration of the reaction led to decomposition. An analogous experiment at 90 °C with mono‐methylated system **2 k⋅NTf_2_** resulted in low conversion (<10 % by ^1^H NMR spectroscopy) to Au^III^ complex **4 h⋅NTf_2_**. The surprising inactivity associated with the methyl groups of bipyridyl ligand **1 j** was investigated by calculating the ground state geometry of the product of oxidative addition of 4‐iodoanisole with **2 j⋅NTf_2_** (Scheme 4 C). This revealed a distortion to the Au^III^ square planar geometry, which presumably renders oxidative addition thermodynamically unfavorable.

**Scheme 5:**
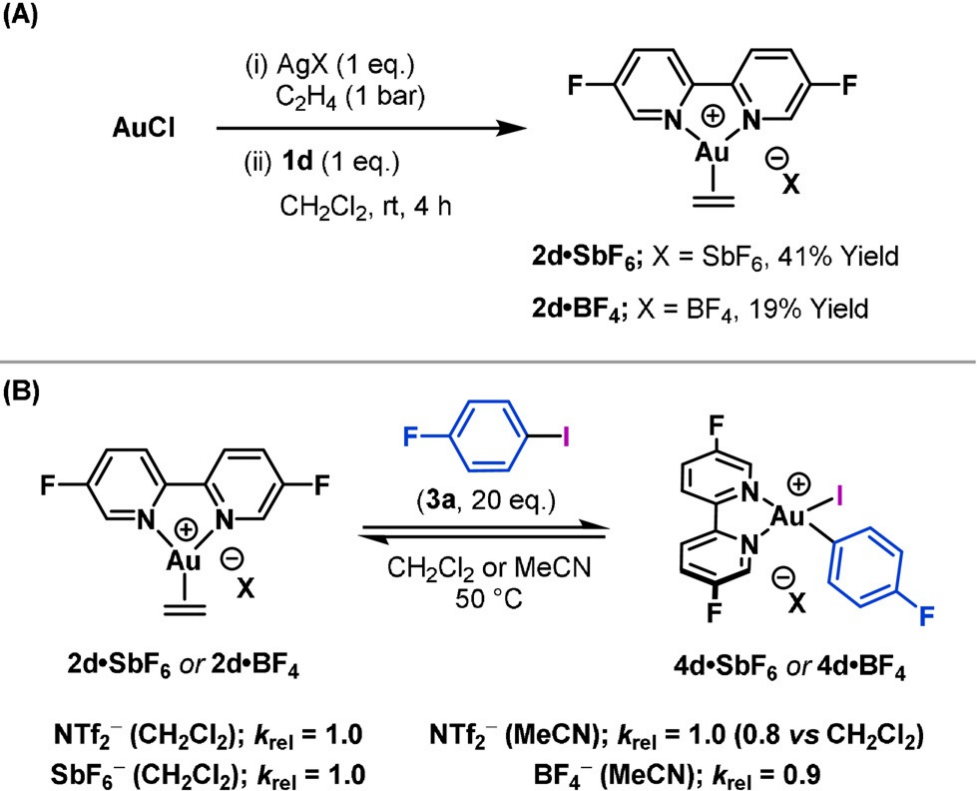
A) Synthesis of Au^I^ complexes **2 d⋅SbF_6_** and **2 d⋅BF_4_** and B) rates of oxidative addition with 4‐fluoroiodobenzene (**3 a**).

The effects of the anion on the oxidative addition reaction were investigated by studying Au^I^ ethylene complexes **2 d⋅SbF_6_** and **2 d⋅BF_4_** (Scheme 5 A). These complexes were generated in 41 % and 19 % yield, respectively, by using the appropriate Ag^I^ salt in the method described earlier. The rates of oxidative addition of 4‐fluoroiodobenzene (**3 a**) to **2 d⋅SbF_6_** (*k*_rel_=1.0) **2 d⋅BF_4_** (*k*_rel_=0.9) were found to be similar to **2 d⋅NTf_2_** (Scheme 5 B). The comparison with **2 d⋅BF_4_** was conducted in MeCN (rather than CH_2_Cl_2_) to aid solubility (Scheme 5 B). Given that initial arene π‐binding is not rate limiting (vide supra), any difference in observed rate can be attributed to the barrier associated with oxidative addition, and the results show that this is largely unaffected by the nature of the anion or the coordinating ability of the solvent. Nevertheless, at the outset, the effects of anion association at the Au center could not be taken for granted. Indeed, Amgoune, Bourissou and co‐workers have shown that Ar−I oxidative addition with [(MeDalPhos)Au]X (X=SbF_6_^−^ or NTf_2_^−^) is significantly slower for the triflate complex, presumably because the triflate anion coordinates more strongly and suppresses π‐coordination or the aryl iodide. This observation was instrumental in developing associated catalysis.^[3a]^

## Conclusion

In summary, the mechanism of oxidative addition of aryl iodides^[31]^ to R_2_‐bipy‐ligated Au^I^ complexes has been investigated using a range of theoretical and experimental approaches. This provides valuable insight into this process from the viewpoint of the metal complex. By direct computational comparison with group 10 elements (Pd and Pt), oxidative addition to Au^I^ is shown to be much less thermodynamically feasible. The highly adaptable [bipyAu(C_2_H_4_)]^+^ complex facilitated the systematic experimental assessment of the effects of complex structure on different mechanistic aspects, including ligand electronic and steric effects, substrate electronic effects and anion effects. Most significantly, faster rates of oxidative addition were observed using more electron‐poor ligands or more electron‐rich aryl iodides. These findings can be rationalized on the basis that the electropositivity of the Au‐center is a key factor in facilitating electron donation from the C(sp^2^)−I unit. Partial dissociation of the hemilabile bipy ligand enhances this aspect, but this effect is finely balanced because reassociation to a “full” κ^2^‐binding mode is required to facilitate bending and complete the oxidative addition process. Significantly, these unusual observations directly contrast Ar−X oxidative addition with Pd^0^ and other late transition metals. Accordingly, Au‐mediated Ar−X oxidative addition has the potential to underpin new or complementary transformations that offer unusual selectivity. The design of Au complexes for such processes may be facilitated by the “at metal” insights outlined in this study.

## Conflict of interest

The authors declare no conflict of interest.

## Supporting information

As a service to our authors and readers, this journal provides supporting information supplied by the authors. Such materials are peer reviewed and may be re‐organized for online delivery, but are not copy‐edited or typeset. Technical support issues arising from supporting information (other than missing files) should be addressed to the authors.

Supporting Information

Supporting Information
