# Supplementary material for: A Systematic Study of the Effects of Complex Structure on Aryl Iodide Oxidative Addition at Bipyridyl‐Ligated Gold(I) Centers
Source: Angew Chem Int Ed Engl. 2021 Oct 18;60(47):24976–83. doi: 10.1002/anie.202108744 (PMC9298241; doi:10.1002/anie.202108744)
Supplement: Supplementary file 2 — Supporting Information [file ANIE-60-24976-s002.pdf]

Angewandte Chemie
Eine Zeitschrift der Gesellschaft Deutscher Chemiker

Supporting Information

A Systematic Study of the Effects of Complex Structure on Aryl Iodide Oxidative Addition at Bipyridyl-Ligated Gold(I) Centers

Jamie A. Cadge, John F. Bower, and Christopher A. Russell**

anie_202108744_sm_miscellaneous_information.pdf
anie_202108744_sm_cif.zip

Supporting Information

1. Materials and methods **S2**

2. Experimental **S4**

2.1. Aryl iodide substrate synthesis **S4**

2.2. 2,2′-Bipyridyl ligand synthesis **S4**

2.3. 2,2′-Bipyridyl Au(I) ethylene complex synthesis **S8**

2.3.1. General Procedure A – Crystallization of complex S8

2.2.2. General Procedure B – Precipitation of complex S8

2.4. *Bona fide* oxidative addition complex synthesis **S14**

2.4.1. General Procedure C – Oxidative addition complex synthesis S14

3. Crystallographic information **S22**

4. Mechanistic studies **S27**

4.1. Linear free energy relationships **S27**

4.1.1. General considerations S27

4.1.2. Representative experiment S27

4.1.3. Kinetic data S28

4.2. van't Hoff analysis **S29**

4.3. Aryl iodide kinetic isotope effect **S30**

4.4. Counterion and solvent kinetic data **S31**

5. Computational details **S32**

5.1. General considerations **S32**

5.2. Palladium and platinum comparison **S33**

5.2.1 Full potential energy surface comparision for palladium oxidative addition S33

5.2.2. Energies and Cartesian coordinates (Å) for stationary points S34
5.3. Ligand electronic effect S43
5.3.1 Full potential energy surface comparision... S43
5.3.2. Energies and Cartesian coordinates (Å) for stationary points S44
5.3.3. Comparison with experimental rate data... S53
5.4. Oxidative addition with 4-vinyliodobenzene S54
5.4.1. Calculated potential energy surface S54
5.4.2. Energies and Cartesian coordinates (Å) for stationary points S55
5.5. Additional aryl iodides S57
5.5.1. Full potential energy surface comparison S57
5.5.2. Energies and Cartesian coordinates (Å) for stationary points S58
6. Selected spectra S62
7. References S90

1. Materials and methods

Materials

All starting materials were obtained from commercial supplied and, except where otherwise state, used as purchased. Anhydrous solvents (CH_2Cl_2, MeCN, hexane and Et_2O) were dried using an Anhydrous Engineering Grubbs-type system (alumina)[1] and stored over 4 Å molecular sieves. Mesitylene and TMEDA were distilled over CaH. DMF, MeOH and acetone were used as purchased. CD_2Cl_2 was distilled over calcium hydride for NMR spectroscopy. $CDCl_3$ was used directly as purchased.

Methods

For all reactions performed, unless stated otherwise, inert conditions were employed using standard Schlenk line and/or glove box techniques under an atmosphere of dinitrogen or argon using oven-dried glassware. Room temperature (rt) typically fluctuated between 18 – 25 °C depending on the season and time of day. In instances where reactions were monitored, they were followed by NMR spectroscopy or analytical thin-layer chromatography (TLC). Merck TLC silica gel 60 F254 plates were used for TLC and were visualised using UV light and/or with a potassium permanganate solution and exposure to heat. Normal phase flash chromatography was carried out using 60 Å silica with solvent systems specified below.

Instrumentation

^{1}H NMR spectra were measured at 300 MHz on a Jeol ECS 300, at 400 MHz on a Bruker Avance 400, Varian 400-MR, Jeol ECZ400 or Jeol ECS400 and at 500 MHz on a Bruker Avance III HD Cryo. Corresponding ^{13}C frequencies are 75.4 MHz, 101 MHz and 126 MHz. Corresponding ^{19}F frequencies are 282 MHz, 376 MHz and 470 MHz. NMR samples were analysed as solutions with solvents specified below and, unless otherwise stated, at 298 K. ^{1}H and ^{13}C{^{1}H}. NMR spectra were referenced to residual solvent peaks ($CDCl_3$ δ_H 7.26 and δ_C 77.2; CD_2Cl_2 δ_H 5.32 and δ_C 53.8). Chemical shifts (^{1}H and ^{13}C) are reported in parts per million (ppm) relative to tetramethylsilane standard. ^{19}F spectra were measured against CF_3Cl as an external standard. Other internal standards are specified below. Coupling constants (J) are reported to the nearest 0.1 Hz and were calculated using *MestReNova 11.0.* Coupling constants that did not match as a result of digitisation are reported as rounded averages. Multiplicities are defined as followed: s singlet; d doublet; t triplet; q quartet; m multiplet and combinations thereof. Characterisation of novel compounds was supported by 2D NMR experiments.

Mass spectra were recorded on a Bruker microTOF II spectrometer or a Waters Synapt G2S spectrometer by the University of Bristol mass spectrometry service.

Infra-red (IR) spectra were recorded on a Pekin-Elmer Spectrum Two FTIR spectrometer with an attenuated total reflectance (ATR) attachment.

Raman spectra were recorded on a Renishaw 2000 laser Raman spectrometer using a 785 nm excitation wavelength. Spectra were collected at room temperature in air with the sample as a fine powder.

2. Experimental

2.1. *Aryl iodide substrate synthesis*

1-Iodo-4-vinylbenzene (3g)

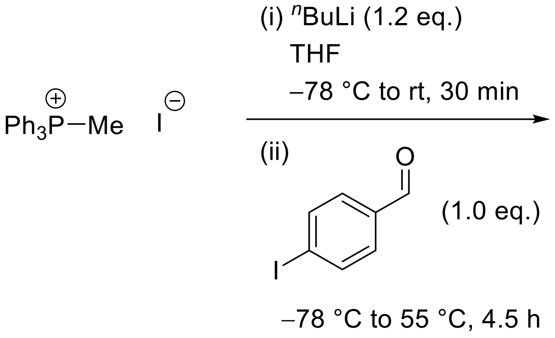

Following a modified literature procedure,[2] methyltriphenylphosphonium iodide (3.50 g, 8.62 mmol) was suspended in THF (40 mL) and cooled to –78 °C. *n*-Butyllithium (4.1 mL of a 2.5 M solution in hexane) was added dropwise and the reaction mixture was stirred for 30 minutes. 4-Iodobenzaldehyde (2.00 g, 8.62 mmol) was added, the reaction mixture stirred for a further 30 minutes then warmed to room temperature. After heating to 55 °C for four hours and cooling to room temperature, sat. aq. NH_4Cl (30 mL) was added followed by ethyl acetate (40 mL). The organic portion was separated and the aqueous phase was washed with ethyl acetate (3 × 40 mL). The organic extracts were combined, dried ($MgSO_4$), filtered and evaporated *in vacuo*. The crude material was purified by normal phase flash chromatography (hexane) to give **3g** (1.00 g, 50%) as a colorless solid; **^{1}H NMR (400 MHz, CDCl$_3$)** δ 7.65 (d, *J* = 8.4 Hz, 2H, C_{Ar}-*H*), 7.15 (d, *J* = 8.4 Hz, 2H, C_{Ar}-*H*), 6.64 (dd, *J* = 17.6, 10.9 Hz, 1H, C*H*=CH_2), 5.76 (d, *J* = 17.6 Hz, 1H, CH=C-*H*), 5.28 (d, *J* = 10.9 Hz, 1H, CH=C-*H*); **^{13}C{^{1}H} NMR (101 MHz, CDCl$_3$)** δ 137.7 (*C*$_{Ar}$), 137.2 (*C*$_{Ar}$), 136.0 (*C*$_{Ar}$), 128.1 (*C*$_{Ar}$), 114.9 (*C*=CH_2), 93.3 (C=*C*H_2). The spectroscopic properties of this compound were consistent with literature data.[3]

2.2. *2,2′-Bipyridyl ligand synthesis*

Many of the ligands used in this study were available from commerical suppliers (2,2′-bipyridine (**1a**), 4,4′-di(*tert*-butyl)-2,2′-bipyridyl (**1b**), 4,4′-dibromo-2,2′-bipyridyl (**1c**), 4,4′-dinitro-2,2′-bipyridyl (**1h**), 4,4′-dimethoxy-2,2′-bipyridyl (**1i**) and 6,6′-dimethyl-2,2′-bipyridyl (**1j**). Others ligands (**1d-g** and **1k**) were synthesised *via* halopyridine homocoupling reactions or further derivitization of commerically available 2,2′-bipyridyls which are outlined below.

5,5′-Difluoro-2,2′-bipyridyl (1d)

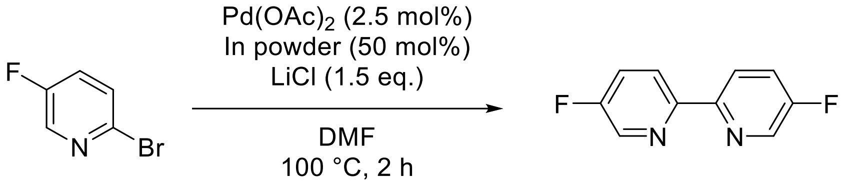

Following a modified literature procedure,[4] 2-bromo-5-fluoropyridine (3.50 g, 19.9 mmol), indium powder (1.14 g, 10.0 mmol), lithium chloride (1.27 g, 30.0 mmol) and palladium(II) acetate (112 mg, 0.50 mmol) were suspended in DMF (40 mL) which was deoxygenated by sparging with dinitrogen for 10 minutes. The reaction mixture was heated at 100 °C for two hours, after which sat. aq. $NaHCO_3$ (50 mL), water (100 mL) and ethyl acetate (50 mL) were added sequentially. The organic portion was separated and the aqueous phase was washed with ethyl acetate (3 × 50 mL). The organic extracts were combined, dried ($MgSO_4$), filtered and evaporated *in vacuo*. The crude material was purified by normal phase flash chromatography (5% ethyl acetate in hexane) to give **1d** (1.52 g, 80%) as a colorless solid; **^{1}H NMR (400 MHz, $CDCl_3$)** δ 8.49 (d, *J* = 2.9 Hz, 2H, C_{Ar}-*H*), 8.38 (dd, *J* = 8.8, 4.4 Hz, 2H, Ar-*H*), 7.51 (app. td, *J* = 8.4, 2.9 Hz, 2H, C_{Ar}-*H*); **^{13}C{^{1}H} NMR (101 MHz, $CDCl_3$)** δ 159.9 (d, *J* = 260 Hz, C_{Ar}), 151.7 (d, *J* = 4.1 Hz, C_{Ar}), 137.4 (d, *J* = 24 Hz, C_{Ar}), 123.8 (d, *J* = 18 Hz, C_{Ar}), 122.2 (d, *J* = 5.4 Hz, C_{Ar}). The spectroscopic properties of this compound were consistent with literature data.[5]

Dimethyl [2,2′-bipyridyl]-4,4′-dicarboxylate (1e)

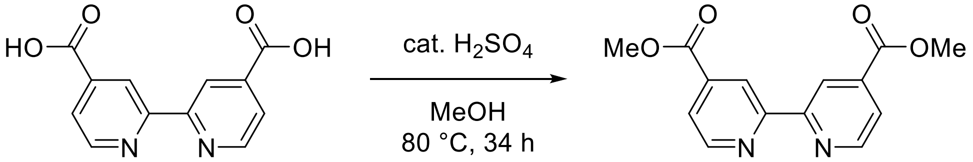

Following a modified literature procedure,[6] 2,2′-dipyridyl-4,4′-dicarboxylic acid (1.00 g, 4.09 mmol) was suspended in methanol (30 mL) and *approx*. 10 drops of conc. H_2SO_4 were added. After heating at 80 °C for 34 hours, the reaction mixture was cooled to room temperature and added to water (75 mL). To the resultant slurry, 25% *w/v* aq. NaOH (75 mL) was added followed by chloroform (75 mL). The organic portion was separated and the aqueous phase was washed with chloroform (3 × 75 mL). The organic extracts were combined, dried ($MgSO_4$), filtered and evaporated *in vacuo* to give **1e** (638 mg, 58%) as a colourless solid; **^{1}H NMR (400 MHz, $CDCl_3$)** δ 8.97 (dd, *J* = 1.6, 0.9 Hz, 2H, C_{Ar}-*H*), 8.87 (dd, *J* = 5.0, 0.9 Hz, 2H, C_{Ar}-*H*), 7.91 (dd, *J* = 5.0, 1.6 Hz, 2H, C_{Ar}-*H*), 4.00 (s, 6H, CH_3); **^{13}C{^{1}H} NMR (101 MHz, $CDCl_3$)** δ

165.8 (*C*=O), 156.6 (*C*Ar), 150.3 (*C*Ar), 138.8 (*C*Ar), 123.4 (*C*Ar), 120.7 (*C*Ar), 52.9 (*C*H_3). The spectroscopic properties of this compound were consistent with literature data.[6]

4,4′-Bis(trifluoromethyl)-2,2′-bipyridyl (1f)

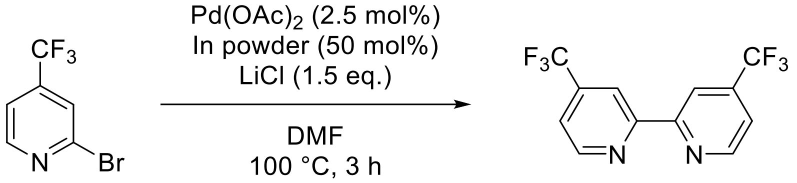

Following a modified literature procedure,[4] 3-bromo-4-bis(trifluoromethyl)pyridine (1.20 mL, 8.89 mmol), palladium(II) acetate (50.0 mg, 0.222 mmol), indium powder (511 mg, 4.45 mmol) and lithium chloride (564 mg, 13.3 mmol) were suspended in DMF (18 mL) which was deoxygenated by sparging with dinitrogen for 10 minutes. After three hours of heating at 100 °C, sat. aq. $NaHCO_3$ (20 mL), water (20 mL) and ethyl acetate (20 mL) were added. The organic portion was separated and the aqueous phase was washed with ethyl acetate (3 × 30 mL). The organic extracts were combined, dried ($MgSO_4$), filtered and evaporated *in vacuo*. The crude material was purified by normal phase flash chromatography (5% to 10% ethyl acetate in hexane) to give **1f** (688 mg, 53%) as a colorless solid; **^{1}H NMR (400 MHz, $CDCl_3$)** δ 8.89 (d, *J* = 5.0 Hz, 2H, CAr-*H*), 8.75 – 8.72 (m, 2H, CAr-*H*), 7.59 (ddd, *J* = 5.0, 1.7, 0.8 Hz, 2H, CAr-*H*); **^{13}C{^{1}H} NMR (126 MHz, $CDCl_3$)** δ 156.2 (*C*Ar), 150.4 (*C*Ar), 139.8 (q, *J* = 34 Hz, *C*Ar), 122.9 (q, *J* = 273 Hz, *C*F_3), 120.1 (*C*Ar), 117.4 (*C*Ar); **^{19}F NMR (377 MHz, $CDCl_3$)** δ -64.83. The spectroscopic properties of this compound were consistent with literature data.[7]

[2,2′-Bipyridyl]-4,4′-dicarbonitrile (1g)

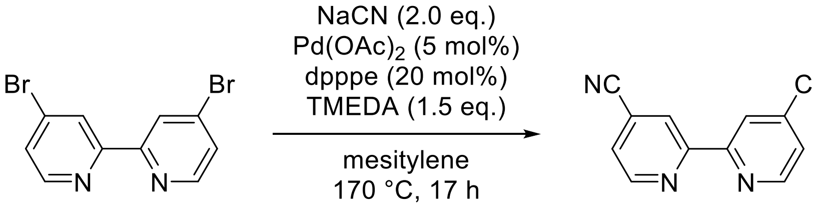

Following a modified literature procedure,[8] 4,4′-dibromobipyridyl (1.90 g, 6.12 mmol), sodium cyanide (600 mg, 12.2 mmol), palladium(II) acetate (69.0 mg, 0.306 mmol), 1,5-bis(diphenylphosphino)-pentane (539 mg, 1.22 mmol) and TMEDA (1.40 mL, 9.18 mmol) were suspended in mesitylene and deoxygenated by sparging with dinitrogen for 10 minutes. The reaction was then heated at 170 °C for 17 hours after which was allowed to cool to room temperature and deoxygenated water (30 mL) was added. The resultant slurry was filtered and

the collected solid was washed with pentane, air-dried and purified by normal phase flash chromatography (30% to 50% ethyl acetate in hexane) to give **1g** (584 mg, 45%) as a colorless solid; **^{1}H NMR (400 MHz, CDCl$_3$)** δ 8.88 (dd, *J* = 5.0, 0.9 Hz, 2H, C$_{Ar}$-*H*), 8.72 (dd, *J* = 1.6, 0.9 Hz, 2H, C$_{Ar}$-*H*), 7.60 (dd, *J* = 5.0, 1.6 Hz, 2H, C$_{Ar}$-*H*); **^{13}C{^{1}H} NMR (101 MHz, CDCl$_3$)** δ 155.6 (*C*≡N), 150.5 (*C*$_{Ar}$), 126.0 (*C*$_{Ar}$), 123.3 (*C*$_{Ar}$), 122.0 (*C*$_{Ar}$), 116.5 (*C*$_{Ar}$). The spectroscopic properies of this compound were consistent with literature data.[8]

6-Methyl-2,2′-bipyridyl (1k)

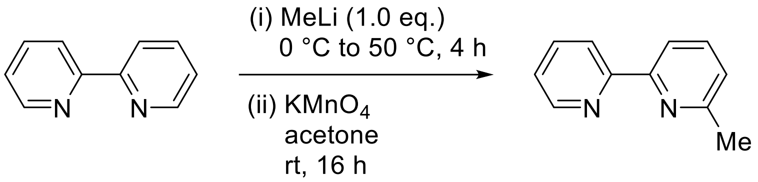

Following a modified literature procedure,[9] 2,2′-bipyridine (8.00 g, 51.2 mmol) was dissolved in diethyl ether (170 mL) and cooled to 0 °C. Methyl lithium (32 mL of a 1.63 M solution in diethyl ether) was added dropwise over 30 minutes. Once addition was complete, the reaction mixture was stirred at room temperature for one hour. After heating at 50 °C for a further four hours, water (120 mL) was added once the reaction was at room temperature. The organic portion was separated and the aqueous phase was washed with diethyl ether (3 × 100 mL). The organic extracts were combined, dried (Na_2SO_4), filtered and evaporated *in vacuo* to give an orange oil. This was dissolved in a sat. solution of potassium permanganate in acetone (200 mL) and stirred for 16 hours. The reaction mixture was filtered and the filtrate was evaporated *in vacuo*. The crude material was purified by normal phase flash chromatography (10% to 20% ethyl acetate in hexane) to give **1k** (3.23 g, 37%) as a colorless solid; **^{1}H NMR (400 MHz, CDCl$_3$)** δ 8.67 (ddt, *J* = 4.8, 1.7, 0.8 Hz, 1H, C$_{Ar}$-*H*), 8.40 (dq, *J* = 8.0, 1.0 Hz, 1H, C$_{Ar}$-*H*), 8.16 (d, *J* = 8.0 Hz, 1H, C$_{Ar}$-*H*), 7.79 (td, *J* = 7.7, 1.8 Hz, 1H, C$_{Ar}$-*H*), 7.69 (t, *J* = 7.7 Hz, 1H, C$_{Ar}$-*H*), 7.31 – 7.23 (m, 1H, C$_{Ar}$-*H*), 7.16 (d, *J* = 7.7 Hz, 1H, C$_{Ar}$-*H*), 2.63 (s, 3H, C*H*$_3$); **^{13}C{^{1}H} NMR (101 Hz, CDCl$_3$)** δ 158.1 (*C*$_{Ar}$), 156.7 (*C*$_{Ar}$), 155.7 (*C*$_{Ar}$), 149.3 (*C*$_{Ar}$), 137.2 (*C*$_{Ar}$), 136.9 (*C*$_{Ar}$), 123.6 (*C*$_{Ar}$), 123.4 (*C*$_{Ar}$), 121.3 (*C*$_{Ar}$), 118.2 (*C*$_{Ar}$), 24.8 (*C*H$_3$). The spectroscopic properties of this compound were consistent with literature data.[9]

2.3. 2,2′-Bipyridyl Au(I) ethylene complex synthesis

2.3.1. General Procedure A – Crystallization of complex

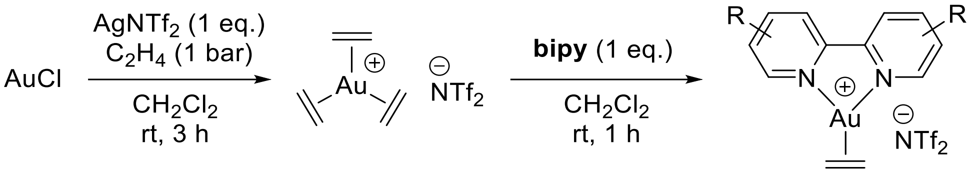

Following a modified literature procedure,[5] in a glovebox, a J. Young's tube was charged with gold(I) chloride (1.0 eq.) and silver(I) triflimide (1.0 eq.) in the absence of light. Outside the glove box, the tube was placed under an atmosphere of ethylene (1 bar) and dichloromethane was added to give a 0.02 M solution. After three hours stirring at room temperature, the reaction mixture was filtered through a pad of Celite into a flask containing the 2,2′-bipyridyl ligand (1 eq.) whilst maintaining darkness. After stirring for one hour, the solution was evaporated *in vacuo* to *approx.* 2 mL and filtered through a pad of Celite. The filtrate was layered with diethyl ether and stored for 24 hours at –18 °C to give the desired Au(I) complex.

2.2.2. General Procedure B – Precipitation of complex

Complexes were prepared in a manner identical to that given in General Procedure A, however the Au(I) complexes were precipitated by adding an excess of diethyl ether to the dichloromethane solution. The mother liquor was removed and the complex was washed thrice with diethyl ether and dried *in vacuo*.

[(κ²-2,2′-Bipy)-(η²-ethylene)gold(I)] [NTf₂] (2a•NTf₂)

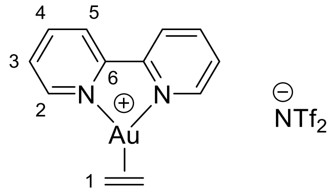

Following General Procedure A on a 0.861 mmol scale with bipyridyl ligand **1a** gave **2a•NTf₂** (136 mg, 24%) as pale yellow needles; **ν_{Raman} (neat) / cm^{-1}** 1493 (C=C); **^{1}H NMR (400 MHz, CD_2Cl_2)** δ 8.86 – 8.81 (m, 2H, C2-*H*), 8.48 (app. dt, *J* = 7.8, 1.4 Hz, 2H, C5-*H*), 8.31 (app. td, *J* = 7.8, 1.4, 2H, C4-*H*), 7.85 (ddd, *J* = 7.8, 5.2, 1.4 Hz, 2H), 3.83 (s, 4H, C1-H_2); **^{13}C{^{1}H} NMR (126 MHz, CD_2Cl_2)** δ 152.5 (*C*6), 152.4 (*C*2), 142.4 (*C*4), 128.5 (*C*3), 124.4 (*C*5), 120.4 (q, *J* = 322 Hz, anion CF_3), 62.3 (*C*1); **HRMS (ESI$^+$)** calcd. 381.0666 for $[M–NTf_2]^+$

$[C_{12}H_{12}AuN_2]^+$, found 381.0645. The spectroscopic properties of this compound were consistent with literature data.[3]

[(κ²-4,4′-Di(*tert*-butyl)-bipy)-(η²-ethylene)gold(I)] [NTf₂] (2b•NTf₂)

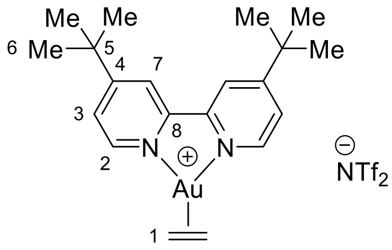

Following General Procedure A on a 0.430 mmol scale with bipyridyl ligand **1b** gave **2b•NTf$_2$** (72 mg, 22%) as a colorless crystalline solid; **ν$_{Raman}$ (neat) / cm^{-1}** 1486 (C=C); **^{1}H NMR (400 MHz, CD$_2$Cl$_2$)** δ 8.73 (dd, *J* = 5.7, 0.7 Hz, 2H, C2-*H*), 8.28 (dd, *J* = 1.9, 0.7 Hz, 2H, C7-*H*), 7.80 (dd, *J* = 5.7, 1.9 Hz, 2H, C3-*H*), 3.77 (s, 4H, C1-*H*$_2$), 1.48 (s, 9H, C6-*H*$_3$); **^{13}C{^{1}H} NMR (101 MHz, CD$_2$Cl$_2$)** δ[a] 167.3 (*C*8), 152.8 (*C*4), 152.1 (*C*2), 125.6 (*C*3), 120.6 (*C*7), 61.4 (*C*1), 36.6 (*C*5), 30.6 (*C*6); **HRMS (ESI$^+$)** calcd. 493.1918 for $[M–NTf_2]^+$ $[C_{20}H_{28}AuN_2]^+$, found 493.1919.

[(κ²-4,4′-Dibromo-bipy)-(η²-ethylene)gold(I)] [NTf₂] (2c•NTf₂)

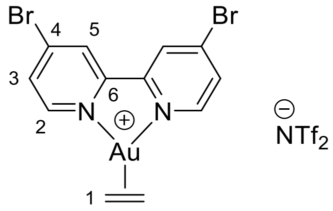

Following General Procedure A on a 0.430 mmol scale with bipyridyl ligand **1c** gave **2c•NTf$_2$** (134 mg, 38%) as a colorless crystalline solid; **ν$_{Raman}$ (neat) / cm^{-1}** 1547 (C=C); **^{1}H NMR (500 MHz, CD$_2$Cl$_2$)** δ 8.76 – 8.64 (m, 2H, C2-*H*), 8.60 – 8.48 (m, 2H, C5-*H*), 8.04 (dd, *J* = 5.6, 1.8 Hz, 2H, C3-*H*), 3.90 (s, 4H, C1-*H*$_2$); **^{13}C{^{1}H} NMR (126 MHz, CD$_2$Cl$_2$)** δ 152.3 (*C*2), 151.8 (*C*6), 139.0 (*C*4), 131.7 (*C*3), 127.3 (*C*5), 119.8 (q, *J* = 322 Hz, anion *C*F$_3$), 63.6 (*C*1); **HRMS (ESI$^+$)** calcd. 538.8856 for $[M–NTf_2]^+$ $[C_{12}H_{10}Au^{79}Br_2N_2]^+$, found 538.8873. Crystals suitable for single-crystal X-ray diffraction were obtained by layering a dichloromethane solution of **2c•NTf$_2$** with diethyl ether followed by storage at –18 °C (X-ray data are given in Section 3).

---

[a] With carbon NMR spectra aquired on lower field instruments (101 MHz), the carbon signal corresponding to the NTf_2^- anion was not observed.

[(κ²-5,5′-Difluoro-bipy)-(η²-ethylene)gold(I)] [NTf₂] (2d•NTf₂)

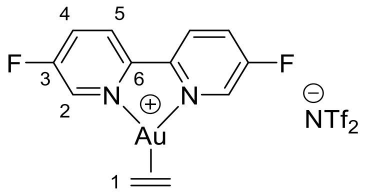

Following General Procedure A on a 0.861 mmol scale with bipyridyl ligand **1d** gave **2d•NTf₂** (360 mg, 60%) as colorless needles; **ν_{Raman} (neat) / cm^{-1}** 1585 (C=C); **^{1}H NMR (400 MHz, CD_2Cl_2)** δ 8.71 (d, *J* = 2.7 Hz, 2H, C2-*H*), 8.58 (dd, *J* = 9.1, 4.2 Hz, 2H, C5-*H*), 8.11 – 8.03 (m, 2H, C4-*H*), 3.93 (s, 4H, C1-H_2); **^{13}C{^{1}H} NMR (126 MHz, CD_2Cl_2)** δ 161.1 (d, *J* = 260 Hz, *C*3), 147.8 (d, *J* = 3.1 Hz, *C*6), 140.6 (d, *J* = 320 Hz, *C*2), 129.0 (d, *J* = 18.6 Hz, *C*4), 125.7 (d, *J* = 7.4 Hz, *C*5), 119.8 (q, *J* = 320 Hz, anion CF_3), 63.8 (*C*1); **^{19}F NMR (377 MHz, CD_2Cl_2)** δ -77.53 (anion CF_3), -115.71 – -116.72 (C3-*F*); **HRMS (ESI$^+$)** calcd. 417.0472 for $[C_{12}H_{10}AuF_2N_2]^+$ [M–NTf_2]$^+$, found 417.0478. The spectroscopic properties of this compound were consistent with literature data.[5]

[(κ²-4,4′-Dimethylcarboxylate)-bipy)-(η²-ethylene)gold(I)] [NTf₂] (2e•NTf₂)

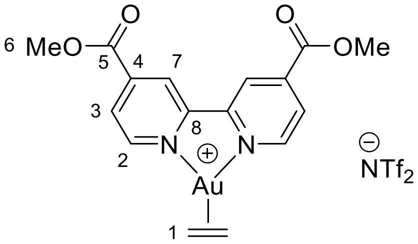

Following General Procedure A on a 0.430 mmol scale with bipyridyl ligand **1e** gave **2e•NTf₂** (89 mg, 27%) as light yellow needles; **ν_{max} (ATR, neat) / cm^{-1}** 1732 (C=O); **ν_{Raman} (neat) / cm^{-1}** 1566 (C=C); **^{1}H NMR (400 MHz, CD_2Cl_2)** δ 9.04 (dd, *J* = 5.4, 0.7 Hz, 2H, C2-*H*), 9.02 (dd, *J* = 1.6, 0.7 Hz, C7-*H*), 8.39 (dd, *J* = 5.4, 1.6 Hz, 2H, C3-*H*), 4.09 (s, 6H, C6-H_3), 3.69 (s, 4H, C1-H_2); **^{13}C{^{1}H} NMR (101 MHz, CD_2Cl_2)** δ 163.8 (*C*5), 153.6 (*C*2), 152.9 (*C*4), 143.0 (*C*8), 128.2 (*C*3), 123.8 (*C*7), 64.4 (*C*1), 54.3 (*C*6); **HRMS (ESI$^+$)** calcd. 497.0776 for [M–NTf_2]$^+$ $[C_9H_{19}AuN_2O_4]^+$, found 497.0779. Crystals suitable for single-crystal X-ray diffraction were obtained by layering a dichloromethane solution of **2e•NTf₂** with diethyl ether followed by storage at –18 °C (X-ray data are given in Section 3).

[(κ²-4,4′-Bis(trifluoromethyl)-bipy)-(η²-ethylene)gold(I)] [NTf₂] (2f•NTf₂)

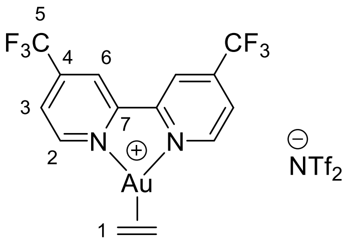

Following General Procedure A on a 0.274 mmol scale with bipyridyl ligand **1f** gave **2f•NTf₂** (72 mg, 33%) as a colorless crystalline solid; **ν_{Raman} (neat) / cm^{-1}** 1573 (C=C); **^{1}H NMR (400 MHz, CD_2Cl_2)** δ 9.13 (d, *J* = 5.4 Hz, 2H, C2-*H*), 8.65 (s, 2H, C6-*H*), 8.15 (d, *J* = 5.4 Hz, 2H, C3-*H*), 4.05 (s, 4H, C1-H_2); **^{13}C{^{1}H} NMR (126 MHz, CD_2Cl_2)** δ 154.2 (*C*2), 152.8 (*C*7), 143.6 (q, *J* = 35.8 Hz, *C*4), 125.3 (q, *J* = 3.4 Hz, *C*3), 122.4 (q, *J* = 274 Hz, *C*5), 120.7 (q, *J* = 3.4 Hz, *C*6), 120.1 (q, *J* = 321 Hz, anion CF_3), 65.7 (*C*1); **^{19}F NMR (377 MHz, CD_2Cl_2)** δ -65.31 (C5-F_3), -79.47 (anion CF_3); **HRMS (ESI$^+$)** calcd. 517.0414 for $[M–NTf_2]^+$ $[C_{14}H_{10}AuN_2F_6]^+$, found 517.0396. Crystals suitable for single-crystal X-ray diffraction were obtained by layering a dichloromethane solution of **2f•NTf₂** with diethyl ether followed by storage at –18 °C (X-ray data are given in Section 3).

[(κ²-4,4′-Dicarbonitrile-bipy)-(η²-ethylene)gold(I)] [NTf₂] (2g•NTf₂)

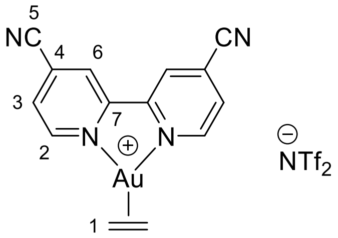

Following General Procedure B on a 0.430 mmol scale with bipyridyl ligand **1g** gave **2g•NTf₂** (97 mg, 32%) as a colorless solid; **^{1}H NMR (400 MHz, CD_2Cl_2)** δ 9.07 (d, *J* = 5.7 Hz, 2H, C2-*H*), 8.66 (s, 2H, C6-*H*), 8.14 (d, *J* = 5.7 Hz, 2H, C3-*H*), 4.03 (s, 4H, C1-H_2); **^{13}C{^{1}H} NMR (101 MHz, CD_2Cl_2)** δ 153.1 (*C*2), 151.5 (*C*4), 130.4 (*C*3), 126.3 (*C*6), 125.8 (*C*7), 114.4 (*C*5), 65.7 (*C*1); **HRMS (ESI$^+$)** calcd. 431.0571 for $[M–NTf_2]^+$ $[C_{14}H_{10}AuN_4]^+$, found 431.0567.

[(κ^2-4,4′-Dinitro-bipy)-(η^2-ethylene)gold(I)] [NTf_2] (2h•NTf_2)

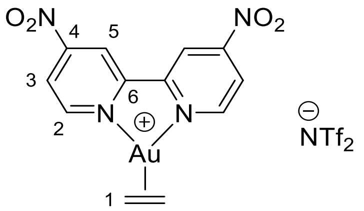

Following General Procedure A on a 0.861 mmol scale with bipyridyl ligand **1h** gave **2h•NTf_2** (98 mg, 30%) as a colorless crystalline solid; **^{1}H NMR (400 MHz, CD_2Cl_2)** δ 9.22 (d, J = 5.6 Hz, 2H, C2-*H*), 9.19 (d, J = 2.1 Hz, 2H, C5-*H*), 8.59 (d, J = 4.4 Hz, 2H, C3-*H*), 4.08 (s, 4H, C1-H_2); **^{13}C{^{1}H} NMR (101 MHz, CD_2Cl_2)** δ 156.5 (*C*4), 155.4 (*C*2), 154.1 (*C*6), 122.3 (*C*3), 118.4 (*C*5), 66.6 (*C*1). Due to the instability of the complex, analysis by HRMS was not possible.

[(κ^2-4,4′-Dimethoxy-bipy)-(η^2-ethylene)gold(I)] [NTf_2] (2i•NTf_2)

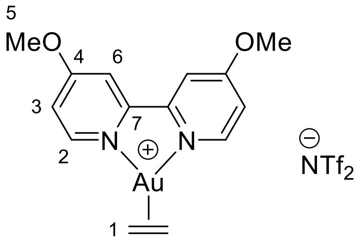

Following General Procedure A on a 0.215 mmol scale with bipyridyl ligand **1i** gave **2i** (44 mg, 31%) as colorless needles; **ν_{Raman} (neat) / cm^{-1}** 1462 (C=C); **^{1}H NMR (500 MHz, CD_2Cl_2)** δ 8.62 (d, J = 6.2 Hz, 2H, C2-*H*), 7.77 (d, J = 2.5 Hz, 2H, C6-*H*), 7.25 (dd, J = 6.2, 2.5 Hz, 2H, C3-*H*), 4.08 (s, 6H, C5-H_3), 3.69 (s, 4H, C1-H_2); **^{13}C{^{1}H} NMR (126 MHz, CD_2Cl_2)** δ 169.6 (*C*4), 154.4 (*C*7), 153.4 (*C*2), 120.3 (q, J = 321.6 Hz, anion CF_3), 113.1 (*C*3), 110.6 (*C*6), 60.3 (*C*1), 57.4 (*C*5); **HRMS (ESI$^+$)** calcd. 441.0877 for $[M–NTf_2]^+$ $[C_{14}H_{16}AuN_2O_2]^+$, found 441.0875. Crystals suitable for X-ray diffraction were obtained by layering a dichloromethane solution of **2i•NTf_2** with diethyl ether followed by storage at –18 °C (X-ray data are given in Section 3).

[(κ^2-6,6′-Dimethyl-bipy)-(η^2-ethylene)gold(I)] [NTf_2] (2j•NTf_2)

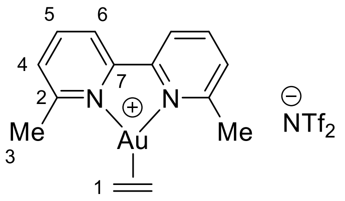

Following General Procedure A on a 0.215 mmol scale with bipyridyl ligand **1j** gave **2j•NTf_2** (40 mg, 26%) as colorless needles; **^{1}H NMR (400 MHz, CD_2Cl_2)** δ 8.26 (d, *J* = 8.0 Hz, 2H, C4-*H*), 8.16 (t, *J* = 7.8 Hz, 2H, C6-*H*), 7.72 (ddd, *J* = 7.8, 1.2, 0.6 Hz, 2H, C5-*H*), 3.79 (s, 4H, C1-H_2), 2.92 (s, 6H, C3-H_3); **^{13}C{^{1}H} NMR (101 MHz, CD_2Cl_2)** δ 160.5 (*C*2), 142.3 (*C*4), 128.5 (*C*6), 121.8 (*C*4), 101.0 (*C*5), 61.2 (*C*1), 28.8 (*C*3); **HRMS (ESI$^+$)** calcd. 409.0979 for $[M–NTf_2]^+$ $[C_{14}H_{16}AuN_2]^+$, found 409.0966.

[(κ^2-6-Methyl-bipy)-(η^2-ethylene)gold(I)] [NTf_2] (2k•NTf_2)

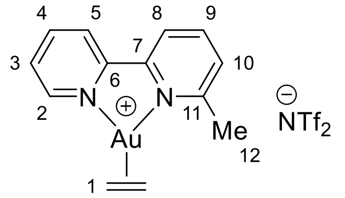

Following General Procedure A on a 0.430 mmol scale with bipyridyl ligand **1k** gave **2k•NTf_2** (86 mg, 30%) as a colorless solid; **^{1}H NMR (500 MHz, CD_2Cl_2)** δ 8.82 (ddd, *J* = 5.2, 1.7, 0.8 Hz, 1H, C2-*H*), 8.45 (dt, *J* = 8.3, 1.0 Hz, 1H, C5-*H*), 8.33 – 8.25 (m, 2H, C4-*H* and C10-*H*), 8.18 (t, *J* = 7.9 Hz, 1H, C8-*H*), 7.83 (ddd, *J* = 7.7, 5.2, 1.2 Hz, C3-*H*), 7.74 (dd, *J* = 7.9, 1.0 Hz, 1H, C9-*H*), 3.81 (s, 4H, C1-H_2), 2.92 (s, 3H, C12-H_3); **^{13}C{^{1}H} NMR (126 MHz, CD_2Cl_2)** δ 160.5 (*C*11), 153.3 (*C*6), 152.4 (*C*2), 152.2 (*C*7), 142.3 (*C*8), 142.3 (*C*4), 128.6 (*C*9), 128.3 (*C*3), 124.5 (*C*5), 121.6 (*C*10), 120.6 (q, *J* = 322 Hz, anion CF_3), 61.6 (*C*1), 28.9 (*C*12); **HRMS (ESI$^+$)** calcd. 395.0823 for $[M–NTf_2]^+$ $[C_{13}H_{14}AuN_2]^+$, found 395.0808.

[(κ^2-5,5′-Difluoro-bipy)-(η^2-ethylene)gold(I)] [SbF_6] (2d•SbF_6)

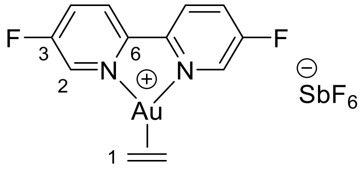

Following General Procedure B on a 0.430 mmol scale with bipyridyl ligand **1d** and $AgSbF_6$ gave **2d•SbF_6** (114 mg, 41%) as a colorless solid; **^{1}H NMR (400 MHz, CD_2Cl_2)** δ 8.71 (d, *J*

= 2.5 Hz, 2H, C2-*H*), 8.52 (dd, *J* = 9.0, 4.2 Hz, 2H, C5-*H*), 8.10 – 8.03 (m, 2H, C4-*H*), 3.94 (s, 4H, C1-H_2); **^{13}C{^{1}H} NMR (126 MHz, CD_2Cl_2)** δ 161.5 (d, *J* = 262.5 Hz, *C*3), 148.1 (d, *J* = 3.3 Hz, *C*6), 141.1 (d, *J* = 30.3 Hz, *C*2), 129.4 (d, *J* = 18.4 Hz, *C*4), 125.9 (d, *J* = 7.4 Hz, *C*5), 64.3 (*C*1); **^{19}F NMR (377 MHz, CD_2Cl_2)** δ -118.00 – -118.06 (m, C3-*F*); **HRMS (ESI$^+$)** calcd. 417.0478 for $[M–SbF_6]^+$ $[C_{12}H_{10}AuF_2N_2]^+$, found 417.0480.

[(κ^2-5,5′-Difluoro-bipy)-(η^2-ethylene)gold(I)] [BF_4] (2d•BF_4)

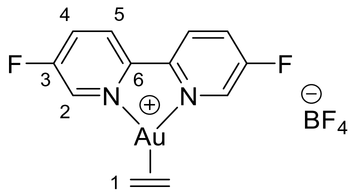

Following General Procedure B on a 0.220 mmol scale with bipyridyl ligand **1d** and $AgBF_4$ gave **2d•BF_4** (58 mg, 54%) as a colorless solid; **^{1}H NMR (400 MHz, CD_3CN)** δ 8.83 (d, *J* = 2.4 Hz, 2H, C2-*H*), 8.52 (dd, *J* = 8.8, 4.6 Hz, 2H, C5-*H*), 8.09 (ddd, *J* = 8.8, 7.6, 2.4 Hz, 2H, C4-*H*), 3.87 (s, 4H, C1-H_2); **^{13}C{^{1}H} NMR (126 MHz, CD_3CN)** δ 162.0 (d, *J* = 258 Hz, *C*3), 148.9 (*C*6), 141.8 (d, *J* = 30.4 Hz, *C*2), 129.6 (d, *J* = 19.4 Hz, *C*4), 126.5 (d, *J* = 4.9 Hz, *C*5), 64.3 (*C*1); **^{19}F NMR (377 MHz, CD_3CN)** δ -118.22 (C4-*F*), -152.41 (BF_4), 152.35 (BF_4); **HRMS (ESI$^+$)** calcd. 417.0478 for $[M–BF_4]^+$ $[C_{12}H_{10}AuF_2N_2]^+$, found 417.0478.

2.4. Bona fide oxidative addition complex synthesis

To verify the presence of the oxidative addition complexes in solution for the kinetic studies, *bona fide* samples were synthesised according to the General Procedure below. Literature spectroscopic data was used for complexes **4a•NTf_2**, **4d•NTf_2**, **5b•NTf_2** and **5f•NTf_2**.[5]

2.4.1. General Procedure C – Oxidative addition complex synthesis

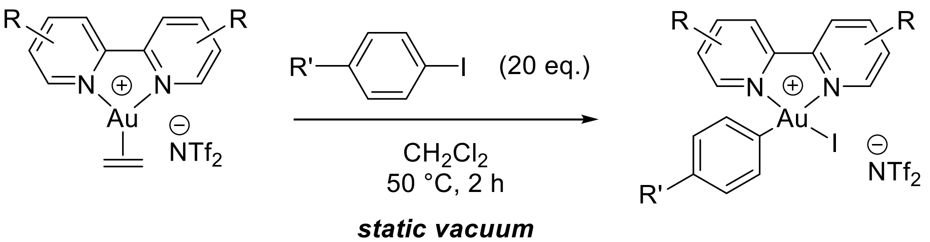

Following a modified literature procedure,[5] gold(I) complex (1.0 eq.) was dissolved in dichloromethane (to give a 0.01 M solution) and the aryl iodide (20 eq.) was added. In the cases where the aryl iodides were solids, they were added at the same time as the gold(I) complex.

The reaction mixture was subjected to three freeze-pump-thaw cycles and heated to 50 °C under static vacuum. After one hour, the reaction mixture was again subjected to three freeze-pump-thaw cycles and returned to heat under static vacuum. This process was continued until the reaction was complete by ^{19}F NMR spectroscopy (typically ~2 h). The reaction mixture was filtered (using a Millipore Millex-HV 0.45 μm PVDF membrane syringe filter) and evaporated *in vacuo* to a minimum volume (*approx.* 0.5 mL). Hexane was added to precipitate the gold(III) oxidative addition complexes which were subsequently washed with hexane and dried under vacuum.

[(κ^2-4,4′-Di(*tert*-butyl)-bipy)Au(4-FC$_6$H$_4$)I][NTf$_2$] (4b•NTf$_2$)

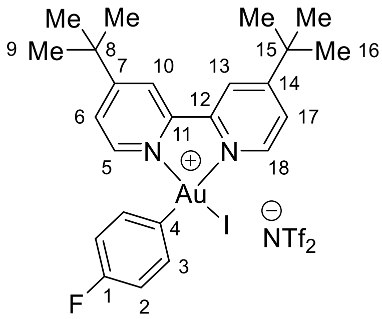

Following General Procedure C on a 25.9 μmol scale with Au(I) complex **2b•NTf$_2$** and 4-fluoroiodobenzene (**3a**) gave **4b•NTf$_2$** (8 mg, 32%) as light brown solid; **^{1}H NMR (500 MHz, CD$_2$Cl$_2$)** δ 9.59 (d, *J* = 6.0 Hz, 1H, C5-*H* or C18-*H*), 8.35 (d, *J* = 2.0 Hz, 1H, C10-*H* or *C*13-*H*), 8.29 (d, *J* = 2.1 Hz, 1H, C10-*H* or C13-*H*), 7.90 (dd, *J* = 6.0, 2.1 Hz, 1H, C6-*H* or C17-*H*), 7.70 (dd, *J* = 6.0, 2.1 Hz, 1H, C5-*H* or C18-*H*), 7.45 (d, *J* = 6.0 Hz, 1H, C6-*H* or C17-*H*), 7.31 – 7.25 (m, 2H, C3-*H*), 7.21 – 7.09 (m, 2H, C2-*H*), 1.51 (s, 9H, C9-H_3 or C16-H_3), 1.46 (s, 9H, C9-H_3 or C16-H_3); **^{13}C{^{1}H} NMR (126 MHz, CD$_2$Cl$_2$)** δ 170.3 (*C*7 or *C*14), 169.2 (*C*7 or *C*14), 163.8 (d, *J* = 248 Hz, *C*1), 155.6 (*C*11 or *C*12), 154.2 (*C*11 or *C*12), 151.0 (*C*5 or *C*18), 146.6 (*C*6 or *C*17), 134.9 (d, *J* = 7.2 Hz, *C*3), 127.4 (*C*6 or *C*17), 126.3 (*C*5 or *C*9), 122.4 (*C*10 or *C*13), 120.1 (q, *J* = 320 Hz, anion *C*F$_3$), 119.1 (*C*4), 118.3 (d, *J* = 21.5 Hz, *C*2), 37.1 (*C*8 or *C*15), 37.0 (*C*8 or *C*15), 30.4 (*C*9 or *C*16), 30.3 (*C*9 or *C*16); **^{19}F NMR (377 MHz, CD$_2$Cl$_2$)** δ -79.37 (anion C*F*$_3$), -114.61 – -114.78 (m, C1-*F*).

[(κ^2-4,4′-Dibromo-bipy)Au(4-FC$_6$H$_4$)I][NTf$_2$] (4c•NTf$_2$)

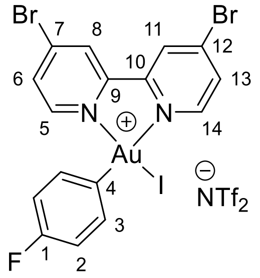

Following General Procedure C on a 26.9 μmol scale with Au(I) complex **2c•NTf$_2$** and 4-fluoroiodobenzene (**3a**) gave **4c•NTf$_2$** (24 mg, 87%) as a yellow solid; **^{1}H NMR (500 MHz, CD$_2$Cl$_2$)** δ 9.48 (d, *J* = 6.1 Hz, 1H, C5-*H* or C14-*H*), 8.62 (d, *J* = 2.0 Hz, 1H, C8-*H* or C11-*H*), 8.57 (d, *J* = 2.0 Hz, 1H, C8-*H* or C11-*H*), 8.12 (dd, *J* = 6.1, 2.0 Hz, 1H, C6-*H* or C13-*H*), 7.93 (dd, *J* = 6.1, 2.0 Hz, 1H, C6-*H* or C13-*H*), 7.34 (d, *J* = 6.1 Hz, 1H, C5-*H* or C14-*H*), 7.30 – 7.24 (m, 2H, C3-*H*), 7.17 (t, *J* = 8.6 Hz, 2H, C2-*H*); **^{13}C{^{1}H} NMR (126 MHz, CD$_2$Cl$_2$)** δ 164.0 (d, *J* = 248 Hz, *C*1), 155.2 (*C*9 or *C*10), 153.9 (*C*9 or *C*10), 151.6 (*C*5 or *C*14), 147.2 (*C*5 or *C*14), 142.5 (*C*7 or *C*12), 141.3 (*C*7 or *C*14), 135.0 (d, *J* = 7.3 Hz, *C*3), 133.8 (*C*6 or *C*13), 132.7 (*C*6 or *C*13), 129.6 (*C*8 and *C*11), 120.3 (q, *J* = 321 Hz, anion *C*F$_3$), 118.6 (*C*4), 118.5 (d, *J* = 21.7 Hz, *C*2); **^{19}F NMR (377 MHz, CD$_2$Cl$_2$)** δ -79.17 (anion C*F*$_3$), -114.02 (C1-*F*); **HRMS (ESI$^+$)** calcd. 730.7905 for [M–NTf$_2$]$^+$ [$C_{16}H_{10}N_2{}^{79}Br_2AuIF$]$^+$, found 730.7916.

[(κ^2-Dimethyl(bipy)-4,4′-dicarboxylate)Au(4-FC$_6$H$_4$)I][NTf$_2$] (4e•NTf$_2$)

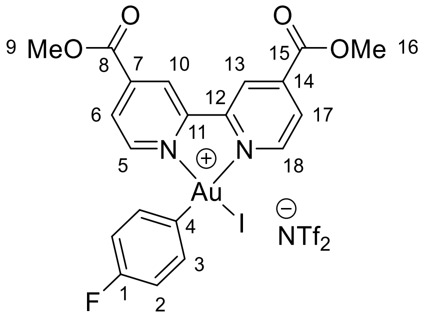

Following General Procedure C on a 14.1 μmol scale with Au(I) complex **2e•NTf$_2$** and 4-fluoroiodobenzene (**3a**) to give **4e•NTf$_2$** (11 mg, 80%) as a yellow solid; **ν$_{max}$ (ATR, neat) / cm^{-1}** 3088, 2958, 1732 (C=O), 1483, 1347, 1179, 1132, 1051, 824, 762, 614; **^{1}H NMR (500 MHz, CD$_2$Cl$_2$)** δ 9.85 (dd, *J* = 5.7, 0.7 Hz, 1H, C5-*H* or C18-*H*), 9.07 (d, *J* = 1.5 Hz, 1H, C10-*H* or *C*13-*H*), 9.01 (d, *J* = 1.7 Hz, 1H, C10-*H* or *C*13-*H*), 8.46 (dd, *J* = 5.7, 1.7 Hz, 1H, C6-*H* or C17-*H*), 8.26 (dd, *J* = 5.7, 1.7 Hz, 1H, C5-*H* or C18-*H*), 7.71 (dd, *J* = 5.7, 0.7 Hz, 1H, C6-*H* or C17-*H*), 7.36 – 7.26 (m, 2H, C3-*H*), 7.20 – 7.10 (m, 2H, C2-*H*), 4.12 (s, 3H, C9-*H*$_3$ or C16-

*H*3), 4.08 (s, 3H, C9-*H*3 or C16-*H*3); **^{13}C{^{1}H} NMR (126 MHz, CD_2Cl_2)** δ 164.0 (d, *J* = 248 Hz, *C*1), 163.2 (*C*8 or *C*15), 162.9 (*C*8 or *C*15), 156.2 (*C*7 or *C*14), 154.9 (*C*7 or *C*14), 152.6 (*C*5 or *C*18), 148.2 (*C*6 or *C*17), 144.5 (*C*11 or *C*12), 143.8 (*C*11 or *C*12), 139.6 (d, *J* = 7.7 Hz, *C*3), 135.0 (d, *J* = 7.3 Hz, *C*4), 129.7 (*C*6 or *C*17), 128.7 (*C*5 or *C*18), 125.5 (*C*10 or *C*13), 125.4 (*C*10 or *C*13), 120.1 (q, *J* = 322 Hz, anion *C*F_3), 118 (d, *J* = 21.5 Hz, *C*2); **^{19}F NMR (377 MHz, CD_2Cl_2)** δ -79.29 (anion C*F*3), -114.15 (tt, *J* = 8.8, 5.3 Hz, C1-*F*); **HRMS (ESI$^+$)** calcd. 690.9804 for $[M–NTf_2]^+$ $[C_{20}H_{16}AuFIN_2O_4]^+$, found 690.9830.

[(κ^2-4,4′-Bis(trifluoromethyl)-bipy)Au(4-FC_6H_4)I][NTf_2] (4f•NTf_2)

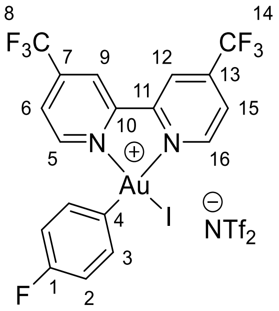

Following General Procedure C on a 46.2 μmol scale with Au(I) complex **2f•NTf_2** and 4-fluoroiodobenzene (**3a**) gave **4f•NTf_2** (33 mg, 78%) as a yellow solid; **^{1}H NMR (500 MHz, CD_2Cl_2)** δ 9.91 (d, *J* = 5.9 Hz, 1H, C5-*H* or C16-*H*), 8.74 (s, 1H, C9-*H* or C12-*H*), 8.68 (s, 1H, C9-*H* or C12-*H*), 8.21 (dd, *J* = 5.9, 1.8 Hz, 1H, C6-*H* or C15-*H*), 8.02 (dd, *J* = 5.9, 1.8 Hz, 1H, C6-*H* or C15-*H*), 7.77 (d, *J* = 5.9 Hz, 1H, C5-*H* or C16-*H*), 7.32 – 7.26 (m, 2H, C3-*H*), 7.16 (t, *J* = 8.9 Hz, 2H, C2-*H*); **^{13}C{^{1}H} NMR (126 MHz, CD_2Cl_2)** δ 164.1 (d, *J* = 249 Hz, *C*1), 156.2 (*C*10 or *C*11), 155.0 (*C*10 or *C*11), 153.1 (*C*5 or *C*16), 148.8 (*C*5 or *C*16), 145.1 (q, *J* = 36.6 Hz, *C*7 or *C*13), 144.5 (q, *J* = 36.6 Hz, *C*7 or *C*13), 135.0 (d, *J* = 7.3 Hz, *C*3), 126.8 (q, *J* = 3.2 Hz, *C*6 or *C*15), 125.9 (q, *J* = 3.1 Hz, *C*6 or *C*15), 122.8 (q, *J* = 3.6 Hz, *C*9 or *C*12), 122.6 (q, *J* = 3.4 Hz, *C*9 or *C*12), 118.7 (d, *J* = 21.7 Hz, *C*2), 116.4 (*C*4); **^{19}F NMR (377 MHz, CD_2Cl_2)** δ -65.34 (C8-*F*3 or C14-*F*3), -65.44 (C8-*F*3 or C14-*F*3), -79.20 (anion C*F*3), -113.70 (C1-*F*); **HRMS (ESI$^+$)** calcd. 710.9442 for $[M–NTf_2]^+$ $[C_{18}H_{10}N_2F_7IAu]^+$, found 710.9422.

[(κ^2-5,5′-Difluoro-bipy)Au(4-OMe-C_6H_4)I][NTf_2] (5c•NTf_2)

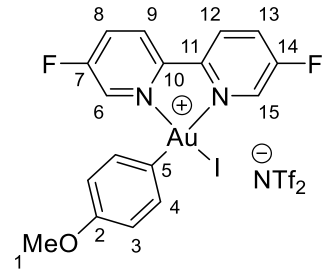

Following General Procedure C on a 27.2 μmol scale with Au(I) complex **2d•NTf_2** and 4-iodoanisole (**3c)** gave **5c•NTf_2** (21 mg, 85%) as a yellow solid; **^{1}H NMR (500 MHz, CD_2Cl_2)** δ 9.53 (dd, *J* = 2.7, 1.9 Hz, 1H, C6-*H* or C15-*H*), 8.73 (dd, *J* = 9.1, 4.5 Hz, 1H, C9-*H* or C12-*H*), 8.69 (dd, *J* = 9.1, 4.5 Hz, 1H, C9-*H* or C12-*H*), 8.24 – 8.17 (m, 2H, C8-*H* and C13-*H*), 7.45 (t, *J* = 2.4 Hz, 1H, C6-*H* or C15-*H*), 7.16 – 7.10 (m, 2H, C4-*H*), 7.00 – 6.94 (m, 2H, C3-*H*), 3.88 (s, 3H, C1-H_3); **^{13}C{^{1}H} NMR (126 MHz, CD_2Cl_2)** δ 163.2 (d, *J* = 178.3 Hz, *C*7 or *C*14), 161.3 (*C*2), 161.1 (d, *J* = 178.0 Hz, *C*7 or *C*14), 151.4 (d, *J* = 4.5 Hz, *C*10 or *C*11), 150.1 (d, *J* = 4.9 Hz, *C*10 or *C*11), 140.5 (d, *J* = 33.2 Hz, *C*6 or *C*15), 136.5 (d, *J* = 34.1 Hz, *C*6 or *C*15), 133.9 (*C*4), 132.1 (d, *J* = 19.1 Hz, *C*8 or *C*13), 131.0 (d, *J* = 19.1 Hz, *C*8 or *C*13), 128.4 (d, *J* = 7.2 Hz, *C*9 or *C*12), 128.2 (d, *J* = 7.2 Hz, *C*9 or *C*12), 120.0 (q, *J* = 320 Hz, anion *C*F_3), 117.6 (*C*3), 114.8 (*C*5), 56.1 (*C*1); **^{19}F NMR (377 MHz, CD_2Cl_2)** δ -79.34 (anion C*F*$_3$), -112.50 (ddt, *J* = 6.9, 4.7, 2.3 Hz, C7-*F* or C14-*F*), -113.61 (ddt, *J* = 6.7, 4.4, 2.2 Hz, C7-*F* or C14-*F*); **HRMS (ESI$^+$)** calcd. 622.9706 for [M–NTf_2]$^+$ [$C_{17}H_{13}AuF_2IN_2O$]$^+$, found 622.9700.

[(κ^2-5,5′-Difluoro-bipy)Au(4-(*tert*-butyl)-C_6H_4)I][NTf_2] (5d•NTf_2)

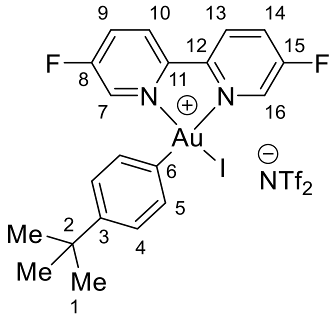

Following General Procedure C on a 27.2 μmol scale with Au(I) complex **2d•NTf_2** and 4-*tert*-butyl iodobenzene (**3d**) gave **5d•NTf_2** (13 mg, 51%)[b] as a dark yellow solid; **^{1}H NMR (500 MHz, CD_2Cl_2)** δ 9.52 (app. t, *J* = 2.3 Hz, 1H, C7-*H* or C16-*H*), 8.76 – 8.65 (m, 2H, C10-*H* and C13-*H*), 8.25 – 8.16 (m, 2H, C9-*H* or C14-*H*), 7.39 (d, *J* = 8.7 Hz, C5-*H*), 7.31 (t, *J* = 2.5 Hz,

[b] Contains Au(I) ethylene complex **2d•NTf_2**

1H, C7-*H* or C16-*H*), 7.19 – 7.11 (m, 2H, C4-*H*), 1.39 (s, 9H, C1-H_3); **^{13}C{^{1}H} NMR (126 MHz, CD_2Cl_2)** δ 163.2 (d, *J* = 181 Hz, *C*8 or *C*15), 161.1 (d, *J* = 181 Hz, *C*8 or *C*15), 153.4 (*C*3), 151.4 (d, *J* = 4.2 Hz, *C*11 or *C*12), 150.0 (d, *J* = 4.1 Hz, *C*11 or *C*12), 140.4 (d, *J* = 32.7 Hz, *C*7 or *C*16), 136.6 (d, *J* = 34.3 Hz, *C*7 or *C*16), 133.0 (*C*4), 132.0 (d, *J* = 19.0 Hz, *C*9 or *C*14), 130.9 (d, *J* = 19.0 Hz, *C*9 or *C*14), 129.3 (*C*5), 128.3 (d, *J* = 7.1 Hz, *C*10 or *C*13), 128.2 (d, *J* = 6.3 Hz, *C*10 or *C*13), 122.9 (*C*6), 120.3 (q, *J* = 322 Hz, anion CF_3), 35.0 (*C*2), 31.7 (*C*1); **^{19}F NMR (377 MHz, CD_2Cl_2)** δ -80.3 (anion CF_3), -113.9 (ddt, *J* = 6.8, 4.6, 2.5 Hz, C8-*F* or C15-*F*), -114.8 (ddt, *J* = 6.5, 4.3, 2.1 Hz, C8-*F* or C15-*F*), **HRMS (ESI+)** calcd. 649.0226 for $[M–NTf_2]^+$ $[C_{20}H_{19}N_2F_2AuI]^+$, found 649.2030.

[(κ^2-5,5′-Difluoro-bipy)Au(4-Me-C_6H_4)I][NTf_2] (5e•NTf_2)

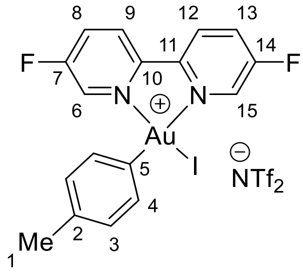

Following General Procedure C on a 18.6 μmol scale with Au(I) complex **2d•NTf_2** and 4-iodotoluene (**3e**) gave **5e•NTf_2** (16 mg, 97%) as a yellow solid; **^{1}H NMR (500 MHz, CD_2Cl_2)** δ 9.52 (dd, *J* = 2.7, 1.8 Hz, 1H, C6-*H* or C15-*H*), 8.73 (dd, *J* = 9.1, 4.5 Hz, 1H, C9-*H* or C12-*H*), 8.69 (dd, *J* = 9.1, 4.5 Hz, 1H, C9-*H* or C12-*H*), 8.24 – 8.17 (m, 2H, C8-*H* or C13-*H*), 7.40 (app. t, *J* = 2.4 Hz, 1H, C6-*H* or C15-*H*), 7.21 – 7.16 (m, 2H, C4-*H*), 7.13 – 7.07 (m, 2H, C3-*H*), 2.48 (s, 3H, C1-H_3); **^{13}C{^{1}H} NMR (126 MHz, CD_2Cl_2)** δ 163.2 (d, *J* = 176.0 Hz, *C*7 or *C*14), 161.1 (d, *J* = 175.7 Hz, *C*7 or *C*14), 151.4 (d, *J* = 4.2 Hz, *C*10 or *C*11), 150.0 (d, *J* = 4.3 Hz, *C*10 or *C*11), 140.4 (d, *J* = 32.9 Hz, *C*6 or *C*15), 140.4 (*C*2), 136.5 (d, *J* = 34.1 Hz, *C*6 or *C*15), 133.3 (*C*3), 132.8 (*C*4), 132.1 (d, *J* = 19.0 Hz, *C*8 or *C*13), 130.9 (d, *J* = 19.1 Hz, *C*8 or *C*13), 128.4 (d, *J* = 6.9 Hz, *C*9 or *C*12), 128.2 (d, *J* = 7.1 Hz, *C*9 or *C*12), 122.5 (*C*5), 120.3 (q, *J* = 320 Hz, anion CF_3), 20.3 (*C*1); **^{19}F NMR (377 MHz, CD_2Cl_2)** δ -77.43 (anion CF_3), -110.82 (ddt, *J* = 6.6, 4.5, 2.2 Hz, C6-*F* or C14-*F*), -111.74 (ddt, *J* = 6.8, 4.6, 2.2 Hz, C6-*F* or C14-*F*); **HRMS (ESI$^+$)** calcd. 606.9757 for $[M–NTf_2]^+$ $[C_{17}H_{13}AuF_2N_2I]^+$, found 606.9767.

[(κ^2-5,5′-Difluoro-bipy)Au(4-Br-C_6H_4)I][NTf_2] (5f•NTf_2)

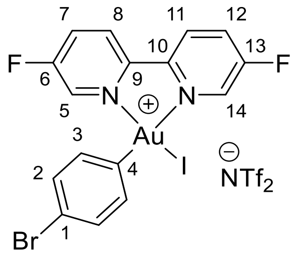

Following General Procedure C on a 33.0 μmol scale with Au(I) complex **2d•NTf_2** and 1-bromo-4-iodobenzene (**3f**) gave **5f•NTf_2** (19 mg, 62%) as a yellow solid; **^{1}H NMR (500 MHz, CD_2Cl_2)** δ 9.56 (app. t, *J* = 2.3 Hz, 1H, C5-*H* or C14-*H*), 8.69 (dd, *J* = 9.1, 4.6 Hz, 1H, C8-*H* or C11-*H*), 8.65 (dd, *J* = 9.1, 4.6 Hz, 1H, C8-*H* or C11-*H*), 8.25 – 8.16 (m, 2H, C7-*H* and C12-*H*), 7.54 – 7.49 (m, 2H, C2-*H*), 7.45 (app. t, *J* = 2.4 Hz, 1H, C5-*H* or C14-*H*), 7.19 – 7.14 (m, 2H, C3-*H*); **^{13}C{^{1}H} NMR (126 MHz, CD_2Cl_2)** δ 163.2 (d, *J* = 176 Hz, *C*6 or *C*13), 161.1 (d, *J* = 177 Hz, *C*6 or *C*13), 151.6 (d, *J* = 4.1 Hz, *C*9 or *C*10), 150.22 (d, *J* = 4.1 Hz, *C*9 or *C*10), 140.7 (d, *J* = 33.2 Hz, *C*5 or *C*14), 136.4 (d, *J* = 33.9 Hz, *C*5 or *C*14), 135.4 (*C*3), 134.5 (*C*2), 132.1 (d, *J* = 19.0 Hz, *C*7 or *C*12), 131.0 (d, *J* = 19.1 Hz, *C*7 or *C*12), 128.4 (d, *J* = 7.1 Hz, *C*8 or *C*11), 128.3 (d, *J* = 7.3 Hz, *C*8 or *C*11), 124.5 (*C*1), 123.1 (*C*4), 120.4 (q, *J* = 321 Hz, anion *C*F_3); **^{19}F NMR (377 MHz, CD_2Cl_2)** δ -79.19 (anion C*F*$_3$), -112.23 – -112.33 (m, C6-*F* or C13-*F*), -113.29 - -113.37 (m, C6-*F* or C13-*F*); **HRMS (ESI$^+$)** calcd. 670.8706 for [M–NTf_2]$^+$ [$C_{16}H_{10}N_2F_2{}^{79}BrIAu$]$^+$, found 670.8726.

[(κ^2-5,5′-Difluoro-bipy)Au(4-formyl-C_6H_4)I][NTf_2] (5g•NTf_2)

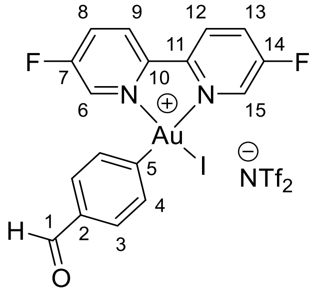

Following General Procedure C on a 27.2 μmol scale with Au(I) complex **2d•NTf_2** and 4-iodobenzaldehyde (**3g**) gave **5g•NTf_2** (16 mg, 18.2 mmol 67%)[c] as a yellow solid; **^{1}H NMR (500 MHz, CD_2Cl_2)** δ 10.08 (s, 1H, C1-*H*), 9.58 (app. t, *J* = 2.4 Hz, 1H, C6-*H* or C15-*H*), 8.67 (m, 2H, C9-*H* and C10-*H*), 8.58 (d, *J* = 2.9 Hz, 1H, C6-*H* or C15-*H*), 8.21 (m, 2H, C8-*H* and C13-*H*), 7.84 (d, *J* = 8.5 Hz, 2H, C3-*H*), 7.54 (d, *J* = 8.5 H, 2H, C4-*H*); **^{13}C{1H} NMR (126**

[c] Contains traces of aryl iodide substrate

MHz, CD_2Cl_2) δ 191.5 (*C*1), 163.2 (d, *J* = 180 Hz, *C*7 or *C*14), 161.1 (d, *J* = 180 Hz, *C*7 or *C*14), 151.7 (d, *J* = 4.0 Hz, *C*10 or *C*11), 150.2 (d, *J* = 3.8 Hz, *C*10 or *C*11), 140.8 (d, *J* = 33.2 Hz, *C*6 or *C*15), 137.6 (*C*2), 136.4 (d, *J* = 33.5 Hz, *C*6 or *C*15), 135.0 (*C*4), 132.8 (*C*5), 132.1 (d, *J* = 18.8 Hz, *C*8 or *C*13), 131.8 (*C*3), 131.0 (d, *J* = 18.9 Hz, *C*8 or *C*13), 128.4 (d, *J* = 7.2 Hz, *C*9 or *C*12), 128.3 (d, *J* = 7.5 Hz, *C*9 or *C*12), 120.3 (q, *J* = 321 Hz, anion CF_3); **^{19}F NMR (377 MHz, CD_2Cl_2)** δ -80.18 (anion C*F*$_3$), -113.39 (C7-*F* or C14-*F*), -114.30 (C7-*F* or C14-*F*); **HRMS (ESI+)** calcd. 620.9550 for $[M–NTf_2]^+$ $[C_{17}H_{11}N_2OF_2IAu]^+$, found 620.9541.

3. Crystallographic information

X-ray diffraction experiments on **2c•NTf2**, **2e•NTf2**, **2f•NTf2** and **2i•NTf2** (Figures S1 to S4) were carried out at 100(2) K on a Bruker APEX II diffractometer using Mo-K_{α} radiation (λ = 0.71073 Å). Intensities were integrated in SAINT[10] and absorption corrections based on equivalent reflections were applied using SADABS.[11] The structures were solved using Superflip[12] (**2c•NTf2**, **2e•NTf2** and **2i•NTf2**) or ShelXT[13] (**2f•NTf2**) and refined by full matrix least squares against F^2 in ShelXL[14] using Olex2.[15] All of the non-hydrogen atoms were refined anisotropically, while all of the hydrogen atoms were located geometrically and refined using a riding model. Crystal structure and refinement data are given in Tables S1 and S2. Crystallographic data for compounds **2c•NTf2**, **2e•NTf2**, **2f•NTf2** and **2i•NTf2** have been deposited with the Cambridge Crystallographic Data Centre as supplementary publication. CCDC deposition numbers: 2056126 (**2c•NTf2**); 2056141 (**2e•NTf2**); 2058337 (**2f•NTf2**); 2056142 (**2i•NTf2**). Copies of the data can be obtained free of charge on application to the CCDC, 12 Union Road, Cambridge, CB2 1EZ, U.K. Fax: (+44) 1223 336033. Email: deposit@ccdc.cam.ac.uk.

Figure S1: Thermal ellipsoid plot of complex **2c•NTf$_2$** determined by single-crystal X-ray diffraction. Thermal ellipsoids are shown at the 50% probability level with counterion and hydrogen atoms omitted for clarity. Selected bond lengths (Å) and bond angles (°); Au1-N1 2.166(4), Au1-N2 2.175(5), N1-Au1-N2 75.06(17), Au1-C1 2.071(6), Au1-C2 2.099(6), Au1-C1=C2(centroid) 1.964, C1-C2 1.397(10).

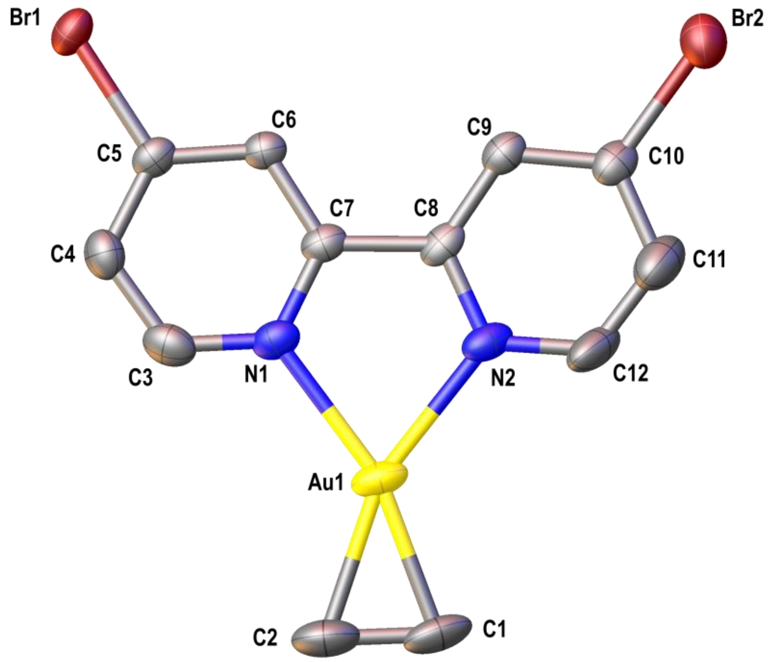

Figure S2: Thermal ellipsoid plot of the grown structure of complex **2e•NTf$_2$** determined by single-crystal X-ray diffraction. Thermal ellipsoids are shown at the 50% probability level with counterion and hydrogen atoms omitted for clarity. Selected bond lengths (Å) and bond angles (°); Au1-N1 2.1793(17), N1-Au1-N1 74.581, Au1-C1 2.092(2), Au1-C1=C1(centroid) 1.970, C1-C1 1.408(5).

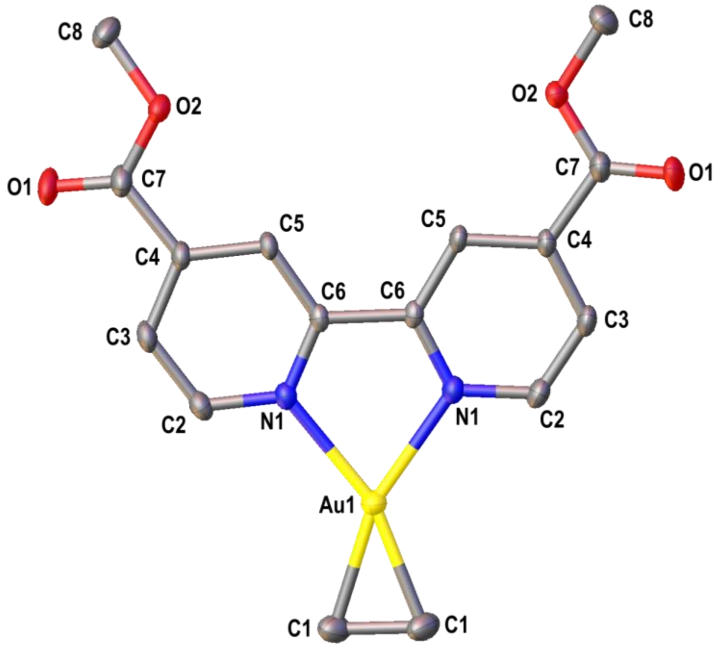

Table S1: Crystal data and structure refinement for **2c•NTf$_2$** and **2e•NTf$_2$**

**Identification code**	**2c•NTf$_2$**	**2e•NTf$_2$**
**Empirical formula**	$C_{14}H_{10}AuBr_2F_6N_3O_4S_2$	$C_{18}H_{16}AuF_6N_3O_8S_2$
**Formula weight**	819.16	777.42
**Temperature/K**	100(2)	100(2)
**Crystal system**	orthorhombic	monoclinic
**Space group**	*Pnna*	*C2/c*
***a*/Å**	19.9969(4)	15.7300(3)
***b*/Å**	22.6201(4)	13.8004(3)
***c*/Å**	9.7743(2)	12.6977(2)
**α/°**	90	90
**β/°**	90	117.4250(10)
**γ/°**	90	90
**Volume/Å^3**	4421.23(15)	2446.64(8)
**Z**	8	4
**ρ_{calc}g/cm^3**	2.461	2.111
**μ/mm^{-1}**	10.536	6.279
**F(000)**	3056.0	1496.0
**Crystal size/mm^3**	0.536 × 0.247 × 0.189	0.641 × 0.467 × 0.244
**Radiation**	MoKα (λ = 0.71073)	MoKα (λ = 0.71073)
**2θ range for data collection/°**	3.062 to 55.79	4.15 to 57.394
**Index ranges**	$-26 \leq h \leq 26$, $-29 \leq k \leq 29$, $-12 \leq l \leq 12$	$-20 \leq h \leq 21$, $-18 \leq k \leq 17$, $-15 \leq l \leq 17$
**Reflections collected**	57350	12290
**R_{int} / R_{sigma}**	0.0598 / 0.0291	0.0303 / 0.0249
**Data/restraints/parameters**	5286/286/425	3159/108/233
**Goodness-of-fit on F^2**	1.113	1.062
**Final R indexes [I>=2σ (I)]**	R_1 = 0.0379, wR_2 = 0.0735	R_1 = 0.0178, wR_2 = 0.0435
**Final R indexes [all data]**	R_1 = 0.0532, wR_2 = 0.0781	R_1 = 0.0191, wR_2 = 0.0441
**Largest diff. peak/hole / e Å^{-3}**	1.54/-1.46	1.37/-0.90

Figure S3: Thermal ellipsoid plot of complex **2f•NTf$_2$** determined by single-crystal X-ray diffraction. Thermal ellipsoids are shown at the 50% probability level with counterion and hydrogen atoms omitted for clarity. Selected bond lengths (Å) and bond angles (°); Au1-N1 2.177(7), Au1-N2 2.168(6), N1-Au1-N2 74.8(2), Au1-C1 2.087(8), Au1-C2 2.098(8), Au1-C1=C2(centroid) 1.970, C1-C2 1.413(14)

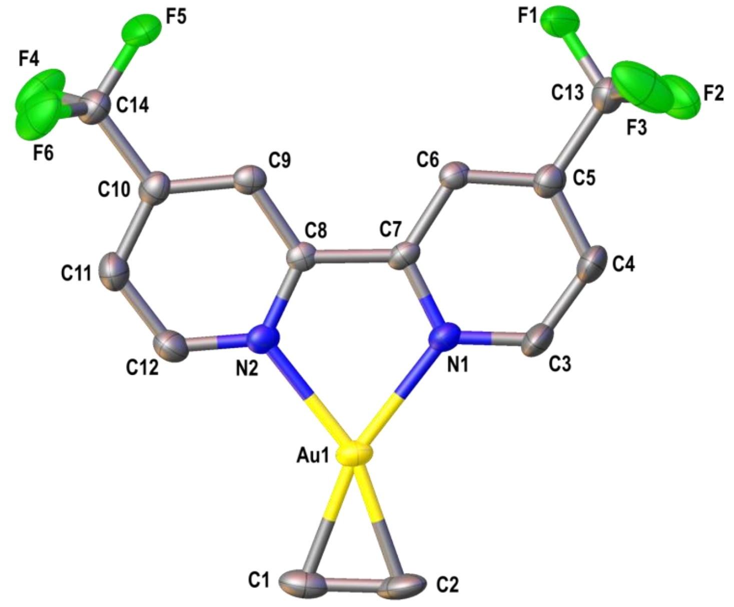

Figure S4: Thermal ellipsoid plot of complex **2i•NTf$_2$** determined by single-crystal X-ray diffraction. Thermal ellipsoids are shown at the 50% probability level with counterion and hydrogen atoms omitted for clarity. Selected bond lengths (Å) and bond angles (°); Au1-N1 2.177(3), Au1-N2 2.158(3), N1-Au1-N2 75.242, Au1-C1 2.095(3), Au1-C2 2.075(3), Au1-C1=C2(centroid) 1.963, C1-C2 1.408(5).

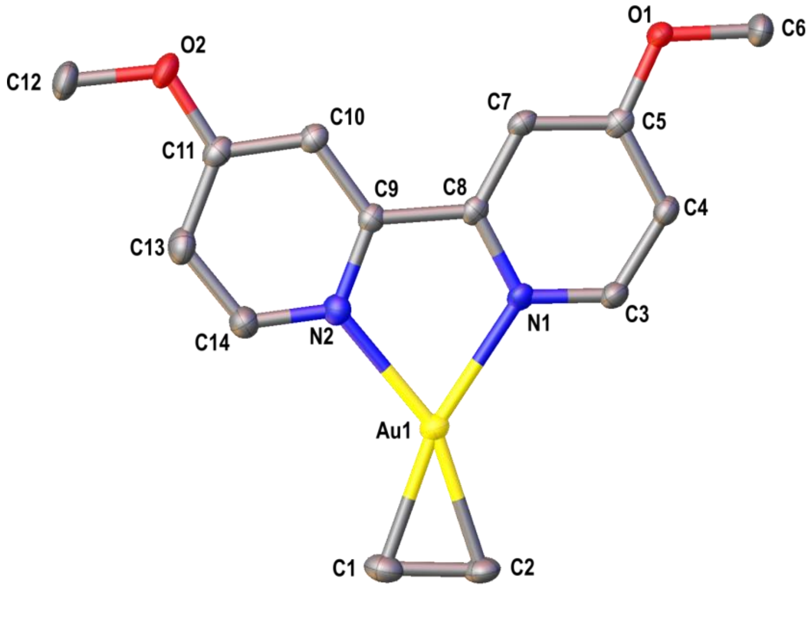

Table S2: Crystal data and structure refinement for **2f•NTf$_2$** and **2i•NTf$_2$**

**Identification code**	**2f•NTf$_2$**	**2i•NTf$_2$**
**Empirical formula**	$C_{16}H_{10}AuF_{12}N_3O_4S_2$	$C_{16}H_{16}AuF_6N_3O_6S_2$
**Formula weight**	797.36	721.40
**Temperature/K**	100(2)	100(2)
**Crystal system**	monoclinic	triclinic
**Space group**	*P2$_1$/c*	*P-1*
***a*/Å**	28.4787(5)	7.22700(10)
***b*/Å**	8.3437(2)	15.8266(3)
***c*/Å**	21.3689(4)	19.6496(3)
**α/°**	90	98.4070(10)
**β/°**	111.5147(10)	99.3110(10)
**γ/°**	90	95.6050(10)
**Volume/Å^3**	4723.84(17)	2177.04(6)
**Z**	8	4
**ρ_{calc}g/cm^3**	2.242	2.201
**μ/mm^{-1}**	6.530	7.041
**F(000)**	3024.0	1384.0
**Crystal size/mm^3**	0.56 × 0.436 × 0.072	0.38 × 0.3 × 0.2
**Radiation**	MoKα (λ = 0.71073)	MoKα (λ = 0.71073)
**2θ range for data collection/°**	1.538 to 52.744	3.092 to 55.128
**Index ranges**	$-35 \le h \le 35$<br>$-10 \le k \le 10$<br>$-26 \le l \le 26$	$-9 \le h \le 9$<br>$-20 \le k \le 20$<br>$-25 \le l \le 25$
**Reflections collected**	73238	38712
**R_{int} / R_{sigma}**	0.0447 / 0.0258	0.0406 / 0.0370
**Data/restraints/parameters**	9670/66/713	10062/30/710
**Goodness-of-fit on F^2**	1.268	1.027
**Final R indexes [I>=2σ (I)]**	R_1 = 0.0446,<br>wR_2 = 0.0989	R_1 = 0.0237,<br>wR_2 = 0.0480
**Final R indexes [all data]**	R_1 = 0.0503,<br>wR_2 = 0.1007	R_1 = 0.0327,<br>wR_2 = 0.0506
**Largest diff. peak/hole / e Å^{-3}**	1.95/-1.69	1.00/-0.88

4. Mechanistic studies

4.1. *Linear free energy relationships*

4.1.1. *General considerations*

Stock solutions were prepared by weighing reagents directly in to J. Young's tubes, which were subsequently placed under an atmosphere of dinitrogen. The necessary amount of solvent (CH_2Cl_2) was measured using 0.1 mL or gas-tight μL-syringes. In instances where reagents were liquids, the volume required was measured using a gas-tight μL syringe. All Au(I) complex stock solutions were freshly prepared as above for immediate use. Amounts of stock solution required were measured using a gas-tight μL syringe where volumes were ≤ 0.1 mL. Volumes above this were measured using a 0.1 mL syringe. Arrays of ^{19}F NMR spectra were collected at 50 ± 0.1 °C at 470 MHz on a Varian VNMRS 500 MHz spectrometer and were implemented using the standard Varian software. A stock solution of trifluorotoluene was used as an internal standard. Each acquisition had 8 scans per spectrum and a 15 s delay between the end of one spectrum and the start of the next. NMR array data was processed using *MestReNova 11.0*. Spectrometer probe temperature was periodically calibrated using the ethylene glycol/methanol-thermometer methods.[16]

4.1.2. *Representative experiment*

Au(I) complex (7.6 μmol), trifluorotoluene (7.6 μmol) and aryl iodide (0.153 mmol) were added as stock solutions in dichloromethane at −78 °C into a J. Young's NMR tube under an atmosphere of dinitrogen. Once the tube had been sealed, and the contents mixed by shaking, it was manually loaded into the NMR spectrometer which had been pre-heated, pre-tuned and pre-shimmed on a dummy sample. The kinetics experiment was started immediately without tuning or shimming. The time between removing the NMR tube from the cold bath and the middle of the first acquisition (*i.e.,* 4th scan, typically <90 s) and the time between collected arrays (typically <30 s) were measured using a stopwatch.

4.1.3. Kinetic data

In all NMR kinetics experiments, initial rates (*i.e.*, before the system reaches equilibrium) were used to determine the rate of oxidative addition as shown by a representative example in Figure S5. Initial rates are quoted in Table S3 for the ligands and aryl iodides with errors given as 95% confidence intervals.

Figure S5: Representative determination of initial rate from raw ^{19}F NMR spectroscopic concentration data. Example is for the oxidative addition of parent bipy complex **2a•NTf₂** and 4-fluoroiodobenzene (**3a**).

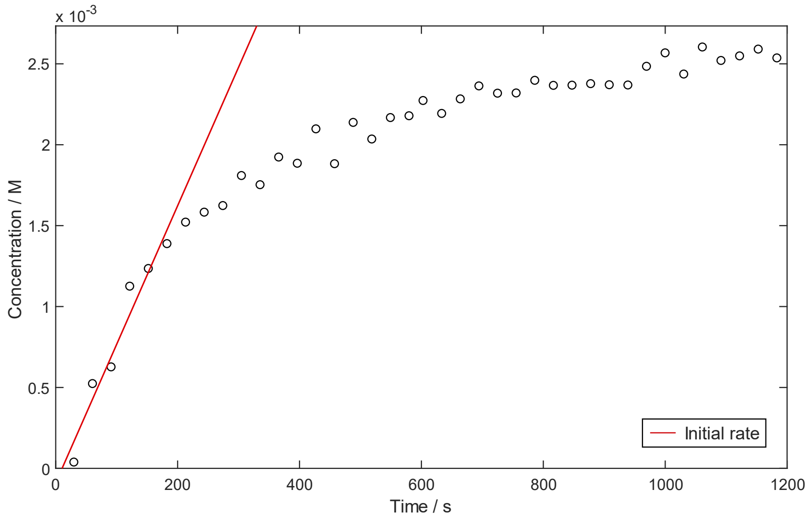

Table S3: Initial oxidative addition rates for ligand and aryl iodide with different substituents

Ligand substituent effects			Aryl iodide substiuent effects		
Subsituent	σ[17]	Initial rate / × 10^{-6} M s^{-1}	Subsituent	σ[17]	Initial rate / × 10^{-6} M s^{-1}
H	0	0.66 ± 0.2	H	0	8.6 ± 1.9
p-^{t}Bu	–0.2	0.43 ± 0.08	*p*-OMe	–0.27	17 ± 0.6
p-Br	0.23	1.0 ± 0.2	*p*-^{t}Bu	–0.20	7.7 ± 2.4
m-F	0.34	1.1 ± 0.3	*p*-Me	–0.17	12 ± 5.2
p-COOMe	0.45	1.4 ± 0.5	*p*-F	0.06	1.8 ± 0.2
p-CF_3	0.54	1.9 ± 0.3	*p*-Br	0.23	0.99 ± 0.09
			p-CHO	0.42	0.63 ± 0.06
			p-CF_3	0.54	0.26 ± 0.02
			p-CH=CH_2	–0.04	0.76 ± 0.8

4.2. *van't Hoff analysis*

Using a similar ^{19}F NMR method outlined above, equilibrium constants (*K*) were approximated[d] according to Equation 1 based on the concentration of Au(III) oxidative addition complex **4d•NTf₂** and starting Au(I) ethylene complex **2d•NTf₂** (from the reaction with 4-fluoroiodobenzene **3a**).

$$K \approx \frac{[Au(III)\ product]}{[Au(I)\ starting\ complex]} \qquad (1)$$

Equilibrium constants at the specified temperatures are given in Table S4 and added to the van't Hoff plot given in Figure S6.

Table S4: Equilibrium constants at different temperatures

T / °C	25	35	40	45	55	65
K	0.08	0.18	0.23	0.46	0.84	1.98

Figure S6: van't Hoff plot for the oxidative addition of 4-fluoroiodobenzene (**3a**) to Au(I) complex **2d•NTf₂**

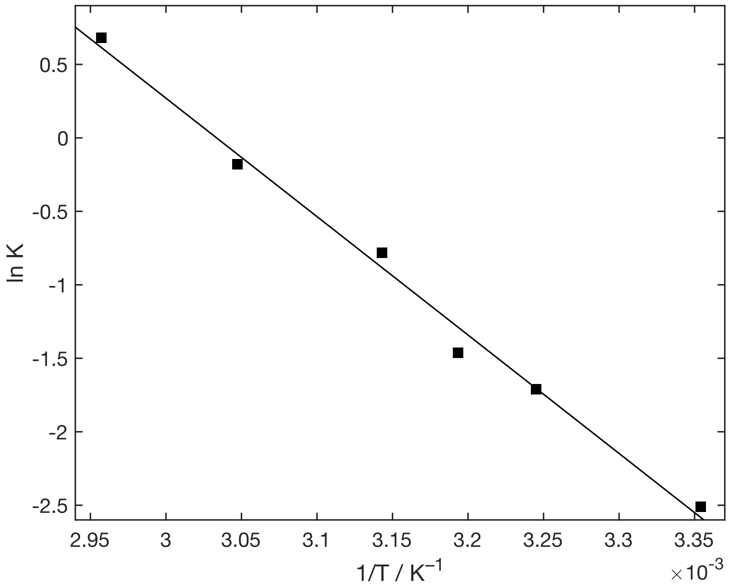

[d] Due to the nature of the reaction which follows bipyAu-C_2H_4 + ArI ⇌ bipyAu-ArI + C_2H_4, where ethylene is lost in the forward reaction and the determination of its concentration is non-trivial, the reaction equation may be simplified to bipyAu-C_2H_4 ⇌ bipyAu-ArI for the equilibrium constant approximation. Also, given the reaction is under pseudo-first-order conditions (20 eq.), the concentration of ArI is assumed to be constant.

The enthalpy of reaction (ΔH) may be approximated by extracting the gradient of the above by rearrangement of the van't Hoff equation (Equation 2) which gives $\Delta_{rxn}H \approx 16$ kcal mol^{-1}. Where K is the equilibrium constant, ΔH is the enthalpy of reaction, $R = 1.987 \times 10^{-3}$ kcal K^{-1} mol^{-1} is the gas constant, T is the temperature, ΔS is the entropy of reaction.

$$lnK = -\frac{\Delta H}{RT} + \frac{\Delta S}{R} \tag{2}$$

4.3. Aryl iodide kinetic isotope effect

Absolute initial oxidative addition rates with both iodobenzene (**3b**) and iodobenzene-d_5 (**3j**) were measured in a manner similar to above and the kinetic isotope effect was calculated according to Equation 3.

$$\frac{k_D}{k_H} = \frac{8.6\times10^{-6}\ M\,s^{-1}}{8.3\times10^{-6}\ M\,s^{-1}} = 1.04 \tag{3}$$

Figure S7: ^{19}F NMR spectroscopic raw data for oxidative addition of iodobenzene (**3b**) and iodobenzene-d_5 (**3j**) with bipy complex **2d•NTf$_2$**. Dashed lines are data fitted to a 6th order polynomial arbitrary function.

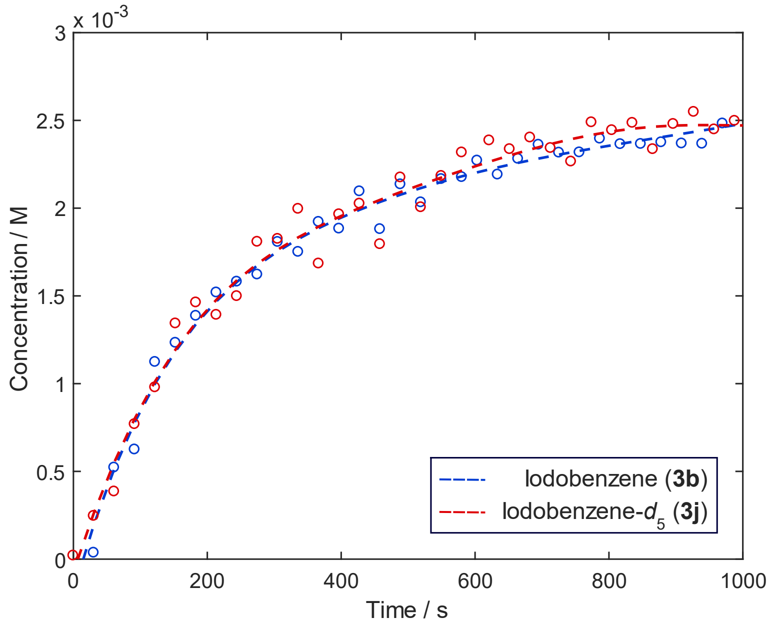

4.4. Counterion and solvent kinetic data

For these experiments related to changing of the Au(I) complex counterion and solvent, initial rates were measured in a manner identical to those described above. In all cases with Au(I) complexes **2d•NTf$_2$**, **2d•SbF$_6$** and **2d•BF$_4$**, 4-fluoroiodobenzene (**3a**) was used as the substrate. Initial rate data is given in Table S5. Errors are given as 95% confidence intervals.

Table S5: Initial rates for counterion and solvent switching experiments

Solvent	Counterion	Initial rate / × 10^{-6} M s^{-1}
CH_2Cl_2	NTf_2^-	1.1 ± 0.3
CH_2Cl_2	SbF_6^-	1.1 ± 0.4
MeCN	NTf_2^-	1.5 ± 0.2
MeCN	BF_4^-	1.3 ± 0.1

5. Computational details

5.1. *General considerations*

All calculations were perfomed using *Gaussian 09, Revision D.01*.[18] The ωB97-XD functional[19] with an ultrafine integration grid was used throughout with Ahlrichs' def2-TZVP basis set on Pd, Pt and Au; def2-SVP on C, N, I and def2-SV on all other atoms.[20] The 60-electron def2 pseudopotentials were used for Pt and Au; 28-electron def2 pseudopotentials were used for Pd and I.[21] Solvation (dichloromethane) was modelled using the SMD model.[22] The nature of all stationary points was confirmed by analysis of the harmonic vibrational frequencies. Energies and Cartesian coordinates relating to *p*-OMe *vs* *p*-CF_3 aryl iodide electronic effects on oxidative addition to $[bipyAu(C_2H_4)]^+$ can be found in reference 5.

5.2. Palladium and platinum comparison

5.2.1 Full potential energy surface comparision for palladium oxidative addition

Figure S8: Calculated potential energy surfaces for *p*-OMe, *p*-^{t}Bu, *p*-H, *p*-CHO and *p*-CF_3 subsituted aryl iodides. See Section 5.1 for computational details.

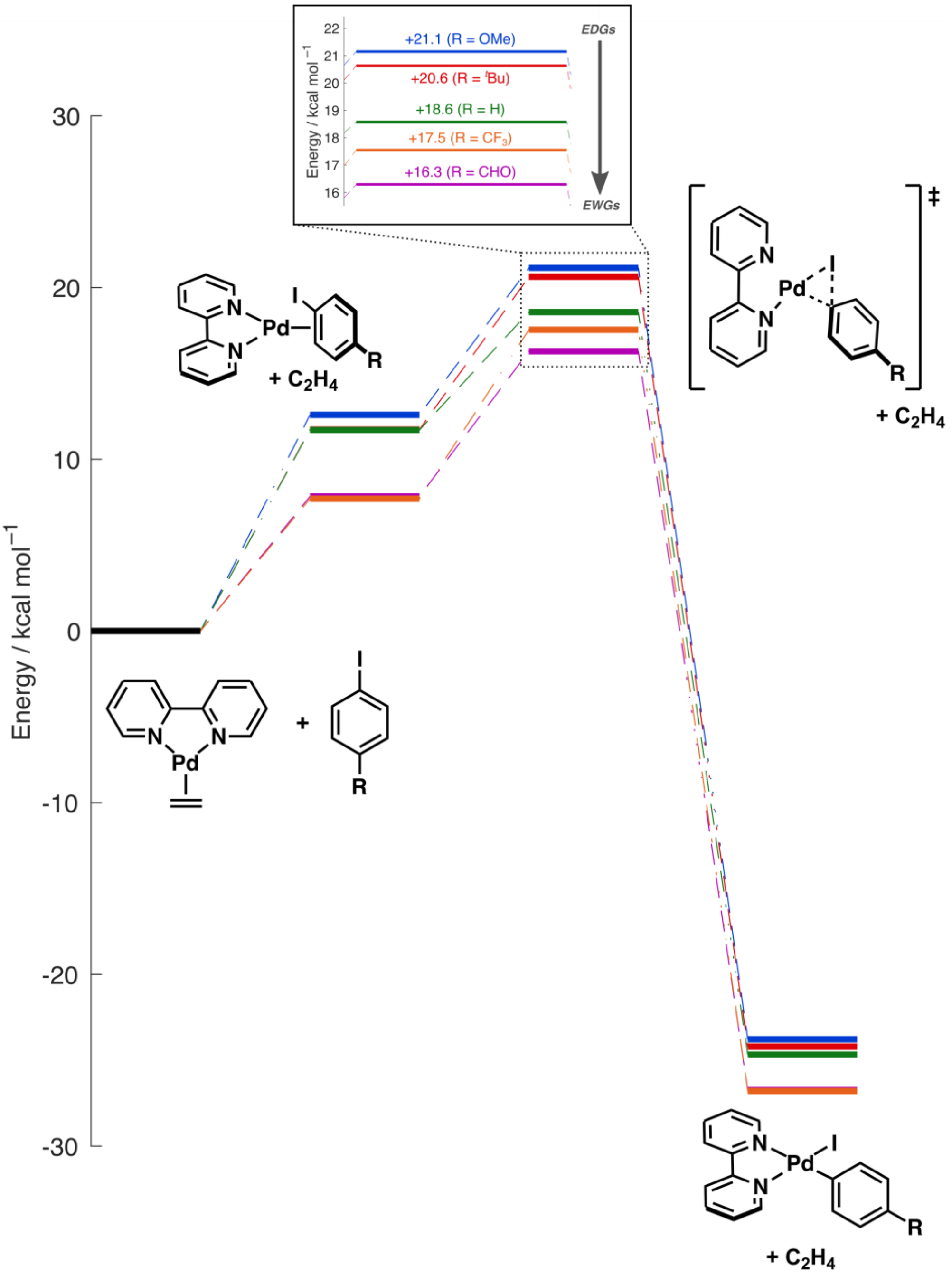

5.2.2. Energies and Cartesian coordinates (Å) for stationary points

(bipy)Pd(C_2H_4)

SCF = –701.37316 au
ZPE = –701.15787 au

C	-0.24520	-2.67340	-0.02870
C	-1.52890	-0.74690	0.00230
C	-2.70520	-1.50180	0.03460
C	-2.62280	-2.89120	0.03020
C	-1.36900	-3.49400	-0.00430
H	0.76170	-3.09870	-0.05210
H	-3.68350	-1.02140	0.06820
H	-3.53450	-3.49410	0.05470
H	-1.25460	-4.58020	-0.01000
C	-1.52870	0.74720	-0.00230
C	-2.70490	1.50220	-0.03460
C	-2.62230	2.89170	-0.03020
H	-3.68330	1.02200	-0.06820
C	-0.24470	2.67340	0.02870
C	-1.36830	3.49420	0.00430
H	-3.53380	3.49470	-0.05470
H	0.76230	3.09860	0.05210
H	-1.25370	4.58040	0.01000
N	-0.32300	-1.34070	-0.02460
N	-0.32270	1.34070	0.02460
Pd	1.44420	-0.00010	0.00000
C	3.37850	0.71380	0.01940
C	3.37850	-0.71420	-0.01940
H	3.65060	-1.28170	0.88490
H	3.65170	-1.23240	-0.95240
H	3.65180	1.23190	0.95240
H	3.65070	1.28130	-0.88490

(bipy)Pd(4-OMe-C_6H_4I) η^2 intermediate

SCF = –1266.42942 au
ZPE = –1266.14394 au

C	3.24240	3.19130	-1.44560
C	3.06440	-0.58520	0.48740
C	4.17790	-1.33930	0.86950
C	3.98380	-2.56500	1.50070
C	2.68570	-3.00900	1.73460
C	1.63080	-2.20350	1.31520
N	1.81730	-1.03060	0.70930
H	5.19220	-0.98830	0.67650
H	4.84460	-3.16690	1.80410
H	2.48590	-3.96250	2.22870
H	0.59140	-2.51100	1.46590
C	3.18090	0.74500	-0.18550
C	4.40770	1.38860	-0.37330
C	4.43730	2.62540	-1.01140
C	2.06190	2.49140	-1.21510
N	2.03300	1.30520	-0.60420
H	5.33830	0.94260	-0.02080
H	5.38910	3.14110	-1.16420
H	3.21650	4.15930	-1.95080
H	1.09670	2.89670	-1.53150
Pd	0.15040	0.20820	-0.15210
C	-2.72660	2.17400	1.01080
C	-2.26630	2.08730	-0.27970
C	-2.74560	1.01040	1.84530
H	-2.30590	2.94370	-0.95720
H	-3.11870	1.11620	2.86810
C	-1.74440	0.84030	-0.79630
C	-2.32340	-0.20410	1.37950
H	-2.38820	-1.08510	2.02530
C	-1.79130	-0.34030	0.04430
I	-2.13570	-2.28800	-0.87650
H	-1.76240	0.73500	-1.88830
O	-3.21160	3.29700	1.59820
C	-3.24070	4.48110	0.84990
H	-2.22810	4.78190	0.51740
H	-3.89740	4.39090	-0.03690
H	-3.64470	5.26270	1.51160

(bipy)Pd(4-OMe-C_6H_4I) transition state

SCF = –1266.41521 au
ZPE = –1266.18292 au

Pd	-0.20980	-0.72050	-0.06390
I	-3.46180	-0.88490	0.06970
C	2.35880	-4.27730	0.23360
C	2.87670	-0.09580	-0.21840
C	3.67640	0.65160	0.63880
C	3.74410	2.03580	0.45870
C	3.02900	2.61520	-0.58610
C	2.26180	1.77620	-1.39250
N	2.18770	0.46300	-1.21780
H	4.19560	0.16230	1.47900
H	3.03770	3.69100	-0.76950
H	1.68160	2.16880	-2.24650
C	2.73590	-1.58030	-0.05130
C	3.84620	-2.40760	0.10890
C	3.65210	-3.77770	0.25440
C	1.30440	-3.39000	0.08240
N	1.48530	-2.07350	-0.05200
H	4.84850	-1.97490	0.11060

H	2.15420	-5.35590	0.31410
H	0.26670	-3.75210	0.05810
C	-1.79280	0.56000	-0.03160
C	-1.73830	1.38940	-1.19370
C	-1.15200	0.97930	1.15720
C	-1.04100	2.57740	-1.14350
C	-0.45040	2.21810	1.17650
C	-0.39390	3.00630	0.04090
H	-1.35880	0.49750	2.11770
H	-0.97430	3.21180	-2.02800
H	-2.25100	1.10010	-2.12040
H	4.50740	-4.44190	0.39060
H	4.34730	2.64710	1.13720
O	0.23480	4.20340	-0.05110
H	-0.00380	2.54300	2.11140
C	0.79120	4.77400	1.09900
H	1.18870	5.74960	0.81230
H	0.02900	4.95000	1.88760
H	1.62080	4.15970	1.48700

(bipy)Pd(4-OMe-C_6H_4I) product

SCF = –1266.48953 au
ZPE = –1266.20189 au

Pd	-0.37690	-0.22420	0.02090
I	0.01370	-2.83480	0.05070
C	-1.47130	4.53090	0.07610
C	-0.13320	4.15630	0.10690
C	0.17690	2.80170	0.07900
N	-0.75990	1.84930	0.02420
C	-2.06520	2.19820	-0.01110
C	-2.44660	3.54030	0.01410
H	0.67020	4.89440	0.15290
H	1.21430	2.46280	0.10280
H	-3.49920	3.82210	-0.01140
C	-5.28810	0.20590	-0.20250
C	-4.75000	-1.07610	-0.18800
C	-3.36700	-1.21120	-0.11960
N	-2.54460	-0.16200	-0.06650
C	-3.05240	1.08590	-0.07750
C	-4.42990	1.30080	-0.14680
H	-5.38260	-1.96530	-0.22910
H	-2.89270	-2.19660	-0.10650
H	-4.84220	2.30970	-0.15740
C	4.35620	0.61310	0.18230
C	3.60500	0.52360	1.36330
C	2.24680	0.21860	1.31550
C	1.58910	0.00400	0.09250
C	2.35320	0.08440	-1.07500
C	3.72140	0.38850	-1.04390
H	1.69250	0.15380	2.25830
H	1.88720	-0.09010	-2.05100
H	4.27480	0.44120	-1.98450
H	-1.76060	5.58460	0.09850
H	-6.36870	0.36050	-0.25660
O	5.66860	0.91350	0.32400
H	4.11090	0.69130	2.31890
C	6.47900	0.96940	-0.82050
H	7.49660	1.20380	-0.47290
H	6.49950	0.00220	-1.35760
H	6.15140	1.76280	-1.51930

4-^{t}Bu-C_6H_4I

SCF = –686.26061 au
ZPE = –686.05718 au

C	-0.07260	0.81980	0.00000
C	-1.19610	-0.00900	0.00000
C	-1.02820	-1.39140	0.00000
C	0.24420	-1.98640	0.00000
C	1.35040	-1.12800	0.00000
C	1.20290	0.26190	0.00000
I	-0.31250	2.91540	0.00000
H	-2.20380	0.41460	0.00000
H	-1.92610	-2.01630	0.00000
C	0.37240	-3.51660	0.00000
H	2.36590	-1.52830	0.00000
H	2.09250	0.89760	0.00000
C	1.83650	-3.97640	0.00000
C	-0.31250	-4.08260	1.25830
C	-0.31250	-4.08260	-1.25830
H	1.87760	-5.07980	0.00000
H	2.37910	-3.62510	-0.89500
H	2.37910	-3.62510	0.89500
H	-0.22270	-5.18380	1.28070
H	0.15490	-3.68300	2.17610
H	-1.38780	-3.83590	1.28960
H	-0.22270	-5.18380	-1.28070
H	-1.38780	-3.83590	-1.28960
H	0.15490	-3.68300	-2.17610

(bipy)Pd(4-^{t}Bu-C_6H_4I) η^2 intermediate

SCF = –1309.11871 au
ZPE = –1308.75376 au

C	2.35920	3.81630	-1.73620
C	3.30720	0.42980	0.64380
C	4.55350	0.19230	1.23110
C	4.73740	-0.95850	1.99270
C	3.67520	-1.84410	2.14950

C	2.46700	-1.53720	1.53000
N	2.29150	-0.43440	0.80120
H	5.38190	0.88890	1.09790
H	5.70640	-1.15860	2.45790
H	3.77380	-2.75920	2.73750
H	1.60290	-2.20280	1.61830
C	3.02260	1.64230	-0.18380
C	3.95470	2.66940	-0.35900
C	3.61780	3.76810	-1.14460
C	1.48620	2.75630	-1.51080
N	1.80910	1.70240	-0.75820
H	4.93580	2.62730	0.11510
H	4.33660	4.57900	-1.28880
H	2.05070	4.65780	-2.36040
H	0.48460	2.74960	-1.94970
Pd	0.39650	0.04600	-0.30110
C	-3.06080	1.04850	0.43110
C	-2.46500	0.93930	-0.80240
C	-2.75700	0.03680	1.40510
H	-2.70680	1.64290	-1.60330
H	-3.21430	0.09010	2.39800
C	-1.52810	-0.10760	-1.12740
C	-1.91910	-1.01440	1.14130
H	-1.75150	-1.78180	1.90360
C	-1.24890	-1.12270	-0.13180
I	-0.84060	-3.16510	-0.78190
H	-1.40170	-0.33030	-2.19430
C	-4.24970	3.16170	-0.34910
C	-4.02780	2.17930	0.80820
C	-5.39360	1.58770	1.20430
C	-3.44670	2.96450	2.00020
H	-4.94200	3.96230	-0.03310
H	-3.30800	3.64330	-0.66630
H	-4.69640	2.66630	-1.22910
H	-6.09670	2.39190	1.48940
H	-5.83980	1.02790	0.36270
H	-5.31270	0.89810	2.06240
H	-4.11870	3.79610	2.28200
H	-3.31670	2.32580	2.89110
H	-2.46060	3.39380	1.74590

C	0.07140	-4.34510	-1.00340
C	0.82060	-3.45950	-1.77440
C	0.37750	-2.14200	-1.88150
N	-0.72500	-1.69500	-1.28550
H	-1.68190	-4.55020	0.24510
H	1.73500	-3.77650	-2.28220
H	0.94080	-1.41010	-2.47030
C	-2.67260	-1.98080	0.10040
C	-3.81730	-2.75880	0.30000
C	-4.93140	-2.19000	0.90880
C	-3.69820	-0.14820	1.06750
N	-2.62350	-0.69150	0.48240
H	-3.84270	-3.79660	-0.03930
H	-5.71950	-0.36240	1.78430
H	-3.60830	0.90100	1.36240
C	0.86820	1.58440	-0.08130
C	1.72040	1.16180	-1.13240
C	1.16200	1.18390	1.25010
C	2.76590	0.29250	-0.86310
C	2.22640	0.29030	1.47440
C	3.03430	-0.18350	0.44040
H	0.66990	1.65110	2.10700
H	3.37780	-0.03630	-1.70580
H	1.53610	1.49670	-2.15700
H	-5.83730	-2.78150	1.06670
H	0.38680	-5.38460	-0.87790
H	2.42010	-0.01490	2.50770
C	4.15820	-1.18710	0.74240
C	3.54240	-2.45760	1.35980
C	4.92810	-1.59520	-0.52130
C	5.15810	-0.56620	1.73580
H	4.32770	-3.20670	1.57050
H	3.02230	-2.24200	2.30920
H	2.80850	-2.91410	0.67130
H	5.71980	-2.31850	-0.25670
H	4.27160	-2.07980	-1.26530
H	5.41820	-0.73150	-1.00430
H	5.97340	-1.27820	1.96060
H	5.61150	0.35070	1.31830
H	4.67560	-0.29920	2.69180

(bipy)Pd(4-*t*Bu-C_6H_4I) transition state

SCF = –1309.10388 au
ZPE = –1308.73959 au

Pd	-0.83130	0.52090	0.08750
I	-0.38360	3.40050	-0.39400
C	-4.87220	-0.85570	1.30280
C	-1.45410	-2.54350	-0.55120
C	-1.08720	-3.88310	-0.38300

(bipy)Pd(4-*t*Bu-C_6H_4I) product

SCF = –1309.17812 au
ZPE = –1308.81103 au

Pd	0.81140	-0.24240	-0.00070
I	0.63700	-2.87580	-0.02310
C	1.50270	4.58760	-0.03580
C	0.20020	4.10260	-0.01880
C	0.00560	2.72650	-0.00120

N	1.02000	1.85550	0.00330
C	2.29180	2.31200	-0.00620
C	2.55860	3.68140	-0.02980
H	-0.66300	4.77120	-0.02100
H	-0.99990	2.30160	0.00890
H	3.58500	4.04730	-0.04540
C	5.67510	0.59630	0.04970
C	5.24610	-0.72610	0.03220
C	3.87770	-0.97630	0.00900
N	2.96990	0.00110	-0.00040
C	3.37150	1.28700	0.01100
C	4.72770	1.61660	0.03950
H	5.95250	-1.55890	0.03830
H	3.48660	-1.99750	-0.00210
H	5.05500	2.65640	0.05700
C	-3.99470	0.23430	0.04300
C	-3.27270	0.15890	-1.15980
C	-1.89290	-0.04650	-1.17870
C	-1.16500	-0.17730	0.01310
C	-1.87870	-0.12100	1.21460
C	-3.26350	0.08450	1.22770
H	-1.38120	-0.09660	-2.14620
H	-1.35730	-0.23270	2.17160
H	-3.76510	0.12480	2.19760
H	1.70290	5.66190	-0.05350
H	6.74050	0.83970	0.07170
H	-3.79470	0.26060	-2.11700
C	-6.11510	0.54480	1.42420
C	-5.51370	0.46580	0.01450
C	-5.81810	1.78920	-0.71250
C	-6.19300	-0.69480	-0.73670
H	-7.20340	0.72060	1.35610
H	-5.96540	-0.39160	1.98960
H	-5.68130	1.37450	2.00990
H	-6.90760	1.97420	-0.73740
H	-5.34040	2.64290	-0.19860
H	-5.45830	1.77740	-1.75600
H	-7.28840	-0.54830	-0.76570
H	-5.83860	-0.77120	-1.77940
H	-5.99040	-1.65990	-0.23900

C_6H_5I

SCF = –529.17186 au
ZPE = –529.08032 au

C	1.21410	1.24770	0.00000
C	0.00000	0.55700	0.00000
C	-1.21410	1.24760	0.00000
C	-1.20720	2.64350	0.00000
C	-0.00010	3.34360	0.00000
C	1.20710	2.64360	0.00000
H	2.16340	0.70570	0.00000
I	0.00000	-1.55310	0.00000
H	-2.16340	0.70560	0.00000
H	-2.15760	3.18480	0.00000
H	-0.00010	4.43730	0.00000
H	2.15750	3.18490	0.00000

(bipy)Pd(C_6H_5I) η^2 intermediate

SCF = –1152.02988 au
ZPE = –1152.01852 au

C	-3.92780	-2.58600	-1.00450
C	-2.59480	1.24290	0.25960
C	-3.42350	2.35020	0.46340
C	-2.86020	3.55290	0.88170
C	-1.48500	3.62320	1.08370
C	-0.72830	2.47810	0.85330
N	-1.26880	1.32630	0.45490
H	-4.49910	2.28940	0.29510
H	-3.49540	4.42760	1.04540
H	-0.99960	4.54530	1.41100
H	0.35740	2.48230	0.99060
C	-3.11560	-0.08680	-0.18470
C	-4.47860	-0.34000	-0.36460
C	-4.88780	-1.60390	-0.78040
C	-2.59120	-2.25700	-0.80010
N	-2.19830	-1.04490	-0.40300
H	-5.22520	0.43310	-0.17990
H	-5.95060	-1.81630	-0.92380
H	-4.20150	-3.59230	-1.32940
H	-1.79980	-2.99480	-0.95830
Pd	-0.07650	-0.51800	0.00720
C	1.97970	-2.93960	1.83200
C	1.63180	-2.99550	0.50650
C	2.31190	-1.68760	2.43100
H	1.43820	-3.96190	0.02780
H	2.58650	-1.65160	3.49030
C	1.55650	-1.80680	-0.30300
C	2.31290	-0.52810	1.69710
H	2.61410	0.41640	2.16100
C	1.92000	-0.53820	0.30740
I	2.89740	0.97770	-0.92530
H	1.64780	-1.94450	-1.38760
H	2.02970	-3.85480	2.43010

(bipy)Pd(C_6H_5I) transition state

SCF = –1152.01852 au
ZPE = –1151.76652 au

Pd	0.15450	-0.67880	0.08880
I	-2.62840	1.29910	-1.07590
C	4.04060	-2.48520	-1.10710
C	2.51320	1.17350	0.40290
C	3.27970	2.28920	0.74400
C	2.64470	3.42410	1.24230
C	1.26120	3.41860	1.38700
C	0.56480	2.26870	1.02760
N	1.17570	1.18300	0.55260
H	4.36450	2.28070	0.63250
H	0.72070	4.28610	1.77140
H	-0.52450	2.21450	1.11130
C	3.10610	-0.08500	-0.13580
C	4.46210	-0.21690	-0.44140
C	4.93280	-1.43150	-0.93310
C	2.70330	-2.27520	-0.78560
N	2.25280	-1.10940	-0.31710
H	5.15170	0.61710	-0.30780
H	4.36640	-3.45560	-1.48740
H	1.96040	-3.06840	-0.90600
C	-1.73030	-0.74750	0.43850
C	-2.35890	-0.67740	1.70210
C	-1.67470	-1.96280	-0.29540
C	-2.85020	-1.84630	2.25620
C	-2.20400	-3.13520	0.30690
C	-2.77350	-3.07750	1.56470
H	-1.46940	-1.97570	-1.37240
H	-3.28800	-1.81830	3.25920
H	-2.40910	0.26920	2.24710
H	5.99150	-1.54890	-1.17870
H	3.23240	4.30490	1.51400
H	-2.20850	-4.07180	-0.26010
H	-3.19100	-3.97940	2.02170

(bipy)Pd(C_6H_5I) product

SCF = –1152.08997 au
ZPE = –1151.83543 au

Pd	-0.12110	-0.12050	0.00750
I	-1.46320	-2.39160	-0.01380
C	2.65490	3.89220	0.00450
C	1.27290	4.03920	0.01930
C	0.48480	2.89430	0.02370
N	1.00370	1.66200	0.01420
C	2.34580	1.50300	0.00300
C	3.19550	2.60990	-0.00300
H	0.79820	5.02240	0.02690
H	-0.60450	2.96360	0.03440
H	4.27770	2.48140	-0.01350
C	4.60450	-1.54550	-0.00720
C	3.62890	-2.53600	0.00670
C	2.29300	-2.14740	0.01420
N	1.91800	-0.86710	0.01030
C	2.85260	0.10330	-0.00000
C	4.21300	-0.20940	-0.01010
H	3.88740	-3.59700	0.01070
H	1.48680	-2.88630	0.02310
H	4.97150	0.57380	-0.01970
C	-4.18990	2.41230	-0.00600
C	-3.57090	2.06420	-1.20810
C	-2.41960	1.27080	-1.20500
C	-1.86040	0.81960	0.00020
C	-2.49880	1.16350	1.20110
C	-3.65090	1.95580	1.19850
H	-1.95130	1.00880	-2.16020
H	-2.09600	0.81330	2.15770
H	-4.13260	2.21490	2.14730
H	3.31320	4.76460	-0.00060
H	5.66660	-1.80390	-0.01490
H	-3.98860	2.40970	-2.15970
H	-5.09250	3.03140	-0.00810

4-CHO-C_6H_4I

SCF = –642.37694 au
ZPE = –642.27646 au

C	0.62230	-1.12340	0.00000
C	2.01050	-1.04360	0.00000
C	2.64750	0.20430	0.00000
C	1.88110	1.37400	-0.00000
C	0.48910	1.30710	-0.00000
C	-0.13190	0.05600	-0.00000
H	0.13050	-2.09920	0.00010
H	2.61580	-1.95450	0.00010
C	4.12780	0.29350	0.00010
H	2.37650	2.35040	0.00000
H	-0.10280	2.22570	-0.00010
I	-2.23370	-0.05630	-0.00000
O	4.86910	-0.66020	-0.00010
H	4.53550	1.33410	0.00030

(bipy)Pd(4-CHO-C_6H_4I) η^2 intermediate

SCF = –1265.24141 au
ZPE = –1265.97926 au

Pd	0.09640	-0.28060	0.34220
I	-1.39450	-2.34570	-1.06260
C	3.73940	-1.33090	2.69200
C	2.74560	1.17120	-0.59680

C	3.65190	2.03520	-1.22160
C	3.21800	2.82280	-2.28420
C	1.89140	2.73270	-2.69570
C	1.05360	1.85370	-2.01460
N	1.46970	1.09830	-1.00110
H	4.68630	2.10920	-0.88480
H	1.50570	3.33210	-3.52360
H	-0.00100	1.75430	-2.29130
C	3.13930	0.29610	0.55130
C	4.46840	0.16920	0.96890
C	4.77130	-0.65230	2.05010
C	2.44490	-1.16000	2.21370
N	2.15300	-0.37280	1.17360
H	5.27200	0.69690	0.45410
H	3.92640	-1.98500	3.54640
H	1.60160	-1.67550	2.68180
C	-1.79600	-0.26330	-0.17310
C	-2.31130	0.72820	-1.06010
C	-2.17970	-0.25320	1.20510
C	-3.05530	1.76830	-0.54660
C	-2.91680	0.83160	1.69280
C	-3.35240	1.84650	0.83670
H	-2.01030	-1.12100	1.84780
H	-3.41650	2.55680	-1.21670
H	-2.08900	0.67360	-2.12900
H	5.80670	-0.76010	2.38370
H	3.91430	3.50390	-2.78100
H	-3.19400	0.87480	2.75080
C	-4.14100	2.97240	1.35450
O	-4.45870	3.13250	2.51440
H	-4.44940	3.72000	0.58020

(bipy)Pd(4-CHO-C_6H_4I) transition state

SCF = –1265.22715 au
ZPE = –1264.96579 au

C	3.53200	2.87810	-1.49460
C	2.92000	-0.81400	0.51220
C	3.93940	-1.63900	0.99570
C	3.60500	-2.82700	1.64000
C	2.26280	-3.16450	1.78580
C	1.30680	-2.29100	1.27600
N	1.62830	-1.15300	0.65960
H	4.98860	-1.36750	0.87700
H	4.39190	-3.48210	2.02300
H	1.95370	-4.08620	2.28370
H	0.23860	-2.51250	1.35980
C	3.19020	0.47780	-0.19050
C	4.48260	0.96940	-0.39340
C	4.65370	2.18310	-1.05350
C	2.27870	2.32240	-1.25580
N	2.11470	1.15800	-0.62450
H	5.35700	0.41990	-0.04330
H	5.65910	2.57960	-1.21780
H	3.61740	3.83460	-2.01500
H	1.36650	2.83020	-1.58120
Pd	0.13760	0.25320	-0.19730
C	-2.44930	2.42040	1.07160
C	-2.06070	2.29010	-0.24960
C	-2.55010	1.25460	1.89720
H	-2.04430	3.18050	-0.88680
H	-2.86510	1.36610	2.94070
C	-1.72620	1.02440	-0.81170
C	-2.27420	0.00940	1.40270
H	-2.39250	-0.87370	2.03770
C	-1.81810	-0.15730	0.03800
I	-2.36400	-2.04990	-0.88090
H	-1.76500	0.93320	-1.90360
C	-2.77630	3.74060	1.62410
O	-2.71770	4.79350	1.02280
H	-3.10170	3.72780	2.69580

(bipy)Pd(4-CHO-C_6H_4I) product

SCF = –1265.29863 au
ZPE = –1265.03435 au

Pd	0.30940	-0.17100	-0.15190
I	-0.32990	-2.80110	-0.11770
C	1.74720	4.44670	-0.19720
C	0.33740	4.15880	-0.35020
C	-0.04680	2.84200	-0.32950
N	0.78800	1.79150	-0.17970
C	2.18930	2.03580	-0.00450
C	2.62470	3.42480	-0.03000
H	-0.40510	4.94950	-0.47940
H	-1.10490	2.58510	-0.43560
H	3.69100	3.63870	0.09110
C	5.14330	-0.18550	0.56450
C	4.46130	-1.45380	0.56610
C	3.09990	-1.43870	0.36700
N	2.35650	-0.33340	0.17490
C	2.99700	0.93760	0.17700
C	4.43990	0.96220	0.37630
H	4.99210	-2.39710	0.71320
H	2.54150	-2.38170	0.35260
H	4.95810	1.92580	0.37640
C	-4.36320	0.83210	-0.28930
C	-3.72200	0.64980	-1.47780
C	-2.35400	0.33280	-1.57680
C	-1.61270	0.19230	-0.40680

C	-2.30460	0.41590	0.77420
C	-3.60350	0.75350	1.07150
H	-1.92600	0.19610	-2.57370
H	-1.70520	0.32510	1.68710
H	-3.52080	0.81760	2.17500
H	2.10310	5.48250	-0.21380
H	6.22810	-0.14440	0.71490
H	-4.30440	0.73790	-2.40770
C	-5.34360	1.09640	1.23400
O	-5.97240	1.17180	1.14010
H	-4.66020	0.99390	1.32460

(bipy)Pd(4-CF_3-C_6H_4I) η^2 intermediate

SCF = –1488.73849 au
ZPE = –1488.48090 au

C	2.54120	3.66680	-1.80790
C	3.26500	0.28820	0.66070
C	4.47700	0.01060	1.29950
C	4.58150	-1.12860	2.09310
C	3.47770	-1.96540	2.22700
C	2.30860	-1.62310	1.55420
N	2.20790	-0.52960	0.79750
H	5.33980	0.66750	1.18410
H	5.52240	-1.35750	2.60070
H	3.51420	-2.86970	2.83840
H	1.41440	-2.25010	1.62160
C	3.06150	1.49490	-0.19860
C	4.04470	2.47380	-0.36740
C	3.77980	3.57180	-1.18110
C	1.61340	2.65340	-1.58700
N	1.86680	1.60120	-0.80590
H	5.00980	2.39510	0.13380
H	4.53870	4.34650	-1.31940
H	2.28850	4.51010	-2.45430
H	0.62440	2.68470	-2.05240
Pd	0.37500	0.03380	-0.33060
C	-2.94870	1.17930	0.46370
C	-2.40890	1.06470	-0.79200
C	-2.70580	0.17300	1.45200
H	-2.64300	1.80300	-1.56390
H	-3.14640	0.27320	2.44830
C	-1.55620	-0.03560	-1.13850
C	-1.93870	-0.92090	1.15940
H	-1.79490	-1.70180	1.91210
C	-1.30890	-1.06120	-0.13490
I	-1.01980	-3.11310	-0.78910
H	-1.46610	-0.26760	-2.20640
F	-4.05960	3.16380	-0.18940
C	-3.78660	2.35750	0.84360
F	-4.96720	1.99350	1.37570
F	-3.18500	3.12150	1.77740

(bipy)Pd(4-CF_3-C_6H_4I) transition state

SCF = –1488.72187 au
ZPE = –1488.46522 au

Pd	0.97210	-0.39810	-0.08370
I	0.87340	-3.38560	0.02030
C	4.87700	1.64660	0.27770
C	0.82520	2.78980	-0.21680
C	0.26200	3.83050	0.52700
C	-1.09200	4.11660	0.35310
C	-1.82420	3.36510	-0.56130
C	-1.16900	2.35140	-1.26210
N	0.11880	2.06570	-1.09470
H	0.86360	4.38360	1.25730
H	-2.88710	3.55810	-0.72220
H	-1.71230	1.74890	-1.99080
C	2.25870	2.41860	-0.04600
C	3.25200	3.39600	0.06500
C	4.57890	3.00600	0.22750
C	3.83080	0.73590	0.16320
N	2.55330	1.10690	0.01120
H	2.98720	4.45470	0.01770
H	5.90070	1.28830	0.40230
H	4.01880	-0.34050	0.19970
C	-0.52100	-1.75550	-0.00110
C	-1.39550	-1.63730	-1.12420
C	-0.87190	-1.11090	1.22240
C	-2.54810	-0.88210	-1.02830
C	-2.04350	-0.33460	1.28270
C	-2.87410	-0.21940	0.17900
H	-0.34180	-1.33800	2.14510
H	-3.20190	-0.78340	-1.90870
H	-1.15360	-2.13010	-2.07230
H	5.37100	3.75560	0.31390
H	-1.56660	4.91070	0.94130
H	-2.30830	0.15690	2.21810
F	-4.12660	1.49280	1.22260
C	-4.14980	0.55700	0.26710
F	-4.46230	1.18650	-0.89070
F	-5.19510	-0.20850	0.51340

(bipy)Pd(4-CF_3-C_6H_4I) product

SCF = –1488.79551 au
ZPE = –1488.53587 au

Pd	0.78910	-0.23290	0.00460

I	0.55780	-2.86060	-0.03920
C	1.59090	4.57730	-0.04800
C	0.27730	4.12410	-0.01830
C	0.04970	2.75340	0.00910
N	1.04290	1.85820	0.01160
C	2.32550	2.28400	-0.00700
C	2.62510	3.64630	-0.04220
H	-0.56950	4.81350	-0.01860
H	-0.96660	2.35570	0.02820
H	3.65980	3.98800	-0.06770
C	5.66490	0.48630	0.05910
C	5.20350	-0.82520	0.04650
C	3.82960	-1.04220	0.02230
N	2.94550	-0.04310	0.00750
C	3.37880	1.23280	0.01440
C	4.74230	1.52900	0.04360
H	5.88910	-1.67510	0.05700
H	3.41440	-2.05370	0.01480
H	5.09320	2.56100	0.05800
C	-3.96980	0.27220	0.03990
C	-3.27520	0.23970	-1.17410
C	-1.89820	0.03160	-1.18340
C	-1.18420	-0.13870	0.01470
C	-1.89840	-0.11680	1.22110
C	-3.27910	0.08890	1.23810
H	-1.37490	0.00920	-2.14470
H	-1.37710	-0.26030	2.17280
H	-3.81330	0.10540	2.19150
H	1.81640	5.64630	-0.07510
H	6.73580	0.70420	0.08160
H	-3.81230	0.37600	-2.11810
F	-5.98540	0.56320	1.24480
C	-5.45490	0.49020	0.01900
F	-5.78600	1.62760	-0.61630
F	-6.10030	-0.49600	-0.62570

(bipy)Pt(C_2H_4)

SCF = –692.84247 au
ZPE = 692.62636 au

C	0.47870	-2.65510	0.02210
C	1.79000	-0.74360	-0.00230
C	2.95620	-1.51070	-0.02750
C	2.85660	-2.89860	-0.02280
C	1.59420	-3.48470	0.00500
H	-0.53300	-3.06700	0.03990
H	3.93830	-1.03730	-0.05530
H	3.76000	-3.51360	-0.04150
H	1.46570	-4.56920	0.01110
C	1.79000	0.74360	0.00230
C	2.95620	1.51070	0.02750
C	2.85650	2.89870	0.02280
H	3.93830	1.03740	0.05530
C	0.47860	2.65510	-0.02210
C	1.59410	3.48470	-0.00500
H	3.76000	3.51370	0.04150
H	-0.53310	3.06690	-0.03990
H	1.46550	4.56920	-0.01110
N	0.57360	-1.32090	0.01720
N	0.57350	1.32090	-0.01720
Pt	-1.16470	-0.00000	0.00000
C	-3.07830	0.72910	-0.01440
C	-3.07830	-0.72910	0.01440
H	-3.40280	-1.27560	-0.88770
H	-3.40350	-1.24010	0.93680
H	-3.40350	1.24000	-0.93680
H	-3.40270	1.27560	0.88770

(bipy)Pt(4-OMe-C_6H_4I) η^2 intermediate

SCF = –1257.89203 au
ZPE = –1257.605976 au

C	3.28000	3.07850	-1.47790
C	3.06850	-0.65050	0.51880
C	4.15130	-1.39490	0.98900
C	3.91320	-2.60550	1.63350
C	2.60100	-3.04220	1.79160
C	1.57450	-2.24620	1.29580
N	1.80490	-1.08410	0.67800
H	5.17430	-1.04010	0.85950
H	4.75000	-3.20090	2.00790
H	2.36700	-3.98480	2.29090
H	0.52710	-2.54510	1.39030
C	3.21160	0.65500	-0.18120
C	4.44390	1.26230	-0.42740
C	4.47770	2.48900	-1.08410
C	2.09140	2.41300	-1.20040
N	2.05920	1.23360	-0.57100
H	5.37460	0.78930	-0.11180
H	5.43440	2.97870	-1.28360
H	3.25720	4.04050	-1.99410
H	1.12540	2.83460	-1.48810
Pt	0.20990	0.17640	-0.12750
C	-2.73870	2.15900	1.05500
C	-2.23030	2.11380	-0.20870
C	-2.76370	0.97010	1.87660
H	-2.27030	2.98200	-0.87140
H	-3.17340	1.05970	2.88700
C	-1.64450	0.87900	-0.73970
C	-2.31300	-0.22400	1.41350

H	-2.38020	-1.11430	2.04690
C	-1.71900	-0.34130	0.08430
I	-2.30270	-2.23580	-0.90190
H	-1.74020	0.78310	-1.83060
O	-3.28470	3.24790	1.65270
C	-3.32410	4.44800	0.93120
H	-2.30950	4.78580	0.64340
H	-3.94240	4.35640	0.01730
H	-3.77690	5.20150	1.59380

(bipy)Pt(4-OMe-C_6H_4I) transition state

SCF = –1257.85590 au
ZPE = –1257.57092 au

Pt	0.23750	-0.69650	-0.00600
I	3.09830	-1.04030	-0.04280
C	-2.43760	-4.14000	-0.21690
C	-2.80970	0.06100	0.21480
C	-3.56480	0.81920	-0.68220
C	-3.60630	2.20360	-0.51350
C	-2.91140	2.77140	0.54940
C	-2.20270	1.92420	1.40400
N	-2.14910	0.60550	1.24280
H	-4.08860	0.33590	-1.51070
H	-2.90900	3.85060	0.72060
H	-1.64980	2.33400	2.25650
C	-2.69960	-1.41980	0.05420
C	-3.84400	-2.21230	-0.02900
C	-3.71580	-3.59150	-0.16620
C	-1.34260	-3.28880	-0.13920
N	-1.46130	-1.95620	-0.01160
H	-4.82800	-1.74170	0.02880
H	-2.27850	-5.21560	-0.32000
H	-0.32330	-3.67790	-0.18830
C	1.66880	0.73420	0.05400
C	1.67450	1.53170	1.23370
C	1.19930	1.29500	-1.15870
C	1.15010	2.80490	1.19980
C	0.65100	2.59470	-1.17130
C	0.60700	3.33710	0.00400
H	1.35320	0.78280	-2.11180
H	1.11090	3.41800	2.10430
H	2.06460	1.12240	2.16930
H	-4.60290	-4.22770	-0.22590
H	-4.17240	2.82780	-1.21090
O	0.07740	4.57110	0.10770
H	0.27700	2.99660	-2.11460
C	-0.49580	5.16370	-1.03200
H	-0.90360	6.13230	-0.70650
H	0.25940	5.33900	-1.82090
H	-1.31530	4.54680	-1.44440

(bipy)Pt(4-OMe-C_6H_4I) product

SCF = –1257.96689 au
ZPE = –1257.67885 au

Pt	-0.33910	-0.17030	0.02300
I	0.07230	-2.76970	0.03730
C	-1.51070	4.52930	0.03070
C	-0.16830	4.17100	0.07260
C	0.16360	2.82300	0.06370
N	-0.76260	1.85510	0.01790
C	-2.07520	2.18900	-0.02600
C	-2.47200	3.52530	-0.02140
H	0.62620	4.91890	0.11210
H	1.20440	2.49870	0.09570
H	-3.52880	3.78980	-0.05610
C	-5.25070	0.12700	-0.17960
C	-4.67940	-1.14060	-0.16380
C	-3.29430	-1.24460	-0.10540
N	-2.49580	-0.17420	-0.06380
C	-3.03770	1.06160	-0.07800
C	-4.41920	1.24240	-0.13630
H	-5.28900	-2.04590	-0.19560
H	-2.79820	-2.21830	-0.09060
H	-4.85320	2.24210	-0.14680
C	4.45100	0.62460	0.19040
C	3.68980	0.58040	1.36680
C	2.32410	0.31320	1.31540
C	1.65340	0.08580	0.09680
C	2.43900	0.12630	-1.06390
C	3.81430	0.39330	-1.03300
H	1.76760	0.28540	2.25890
H	1.97840	-0.05550	-2.04120
H	4.37210	0.41220	-1.97220
H	-1.81300	5.57940	0.03700
H	-6.33520	0.25380	-0.22530
O	5.77160	0.89040	0.33300
H	4.19360	0.75430	2.32260
C	6.58340	0.92120	-0.81100
H	7.60550	1.13770	-0.46470
H	6.58550	-0.05020	-1.34110
H	6.27190	1.71560	-1.51620

S5.3. *Ligand electronic effect*

5.3.1 *Full potential energy surface comparision*

Figure S9: Calculated potential energy surfaces for *p*-OMe, *p*-^{t}Bu, *p*-H, *p*-COOMe and *p*-CF_3 subsituted 2,2´-bipyridyl ligands. See Section 5.1 for computational details.

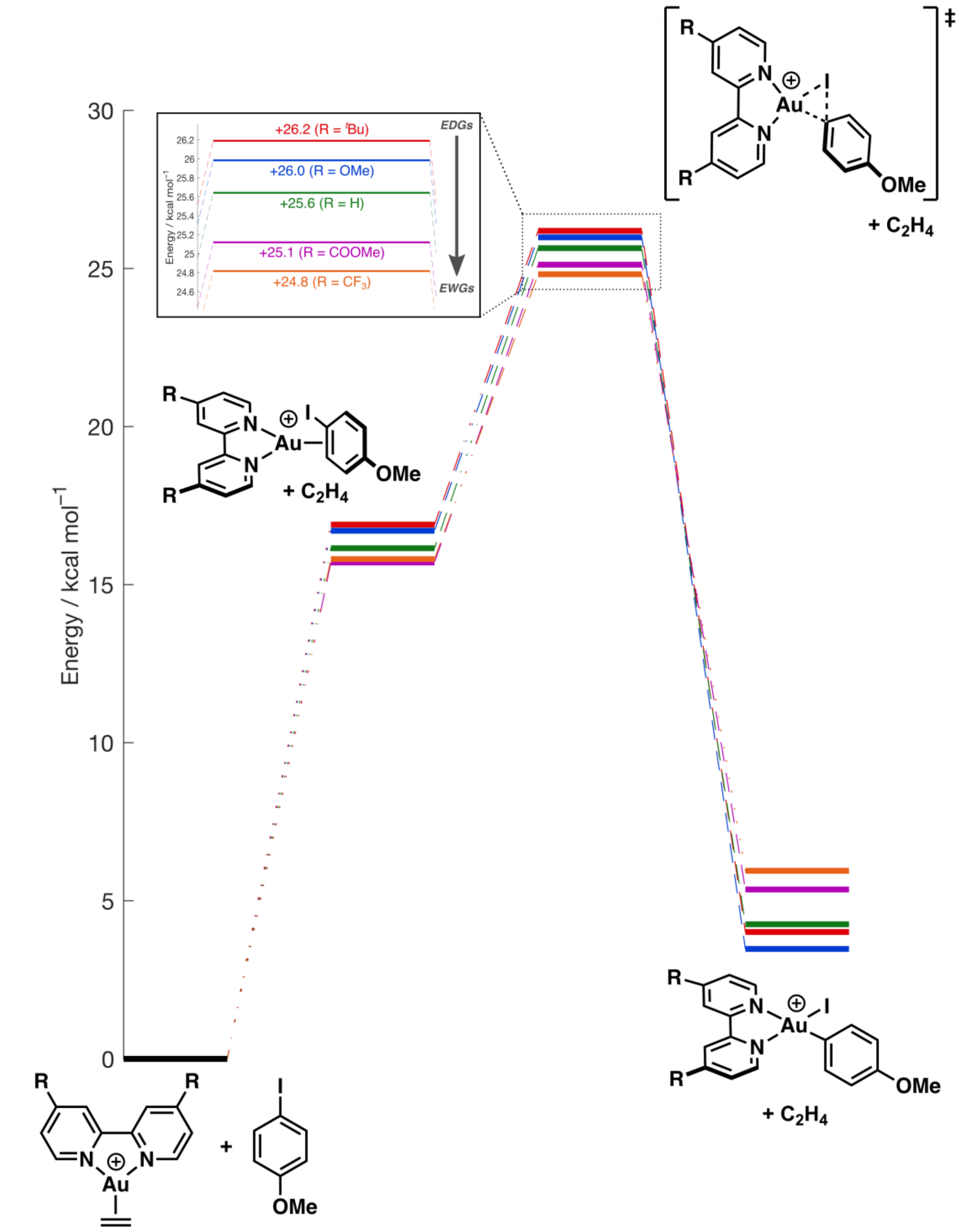

5.3.2. Energies and Cartesian coordinates (Å) for stationary points

(OMe-bipy)Au(C_2H_4)$^+$

SCF = –937.88856 au
ZPE = –937.60597 au

Au	1.90020	0.00010	-0.00000
C	-2.17110	2.89710	0.00570
C	-0.89910	3.48810	0.07520
C	0.21030	2.66900	0.09790
N	0.12620	1.33100	0.06440
C	-1.08130	0.74850	0.01210
C	-2.25530	1.49650	-0.02300
H	-0.79850	4.57450	0.10250
H	1.21600	3.09380	0.14330
H	-3.22320	1.00180	-0.08200
C	-2.17070	-2.89720	-0.00550
C	-0.89860	-3.48810	-0.07470
C	0.21060	-2.66890	-0.09720
N	0.12640	-1.33080	-0.06370
C	-1.08130	-0.74850	-0.01160
C	-2.25520	-1.49660	0.02320
H	-0.79790	-4.57440	-0.10200
H	1.21640	-3.09350	-0.14230
H	-3.22310	-1.00200	0.08190
O	-3.21290	3.71440	-0.03090
O	-3.21250	-3.71470	0.03060
C	-4.52560	-3.19050	0.12470
H	-5.19760	-4.05940	0.14930
H	-4.65300	-2.60520	1.05190
H	-4.76820	-2.56800	-0.75380
C	-4.52590	3.18990	-0.12540
H	-5.19810	4.05870	-0.15000
H	-4.65300	2.60480	-1.05260
H	-4.76860	2.56730	0.75300
C	3.88720	-0.70520	-0.03610
C	3.88750	0.70500	0.03540
H	4.09440	-1.30110	0.86250
H	4.09560	1.20810	0.98890
H	4.09480	1.30070	-0.86330
H	4.09490	-1.20820	-0.98970

(OMe-bipy)Au(4-OMe-C_6H_4I)$^+$ η^2 intermediate

SCF = –1502.93851 au
ZPE = –1502.58517 au

Au	-0.51190	-0.21850	-0.15650
C	3.81100	-2.61770	-0.92800
C	2.59240	-3.26360	-1.19230
C	1.41820	-2.57940	-0.95640
N	1.38870	-1.32430	-0.48450
C	2.54230	-0.69460	-0.21840
C	3.77510	-1.30750	-0.42870
H	2.58230	-4.28460	-1.57780
H	0.45170	-3.05150	-1.15090
H	4.69800	-0.77360	-0.21030
C	3.35820	2.74920	1.17900
C	2.04710	3.23990	1.29080
C	1.00230	2.42340	0.90740
N	1.18570	1.18420	0.43470
C	2.42820	0.70080	0.31840
C	3.54520	1.45210	0.67860
H	1.86820	4.24600	1.67460
H	-0.03220	2.77100	0.97970
H	4.54680	1.03720	0.57930
O	4.92140	-3.29870	-1.17120
O	4.33920	3.55580	1.56010
C	6.18450	-2.70450	-0.92710
H	6.93320	-3.45490	-1.21530
H	6.32190	-1.79770	-1.54150
H	6.30620	-2.46040	0.14260
C	5.68600	3.12740	1.46570
H	6.29590	3.96070	1.84100
H	5.86390	2.23540	2.09140
H	5.96300	2.91640	0.41810
C	-2.57720	0.32920	-0.00740
C	-2.53850	-1.00250	-0.53910
C	-2.96460	0.49890	1.37190
C	-2.90520	-2.11590	0.28720
H	-2.55340	-1.16000	-1.62250
I	-2.91550	1.99080	-1.31170
C	-3.27170	-0.58550	2.14230
C	-3.25440	-1.91130	1.60480
O	-3.61700	-2.87050	2.46700
C	-3.68380	-4.20460	2.01860
H	-2.69570	-4.56460	1.67690
H	-4.00650	-4.80530	2.88120
H	-4.41990	-4.31790	1.20180
H	-3.02090	1.50560	1.79350
H	-3.55880	-0.46070	3.18960
H	-2.92250	-3.10690	-0.16830

(OMe-bipy)Au(4-OMe-C_6H_4I)$^+$ transition state

SCF = –1502.92272 au
ZPE = –1502.57038 au

C	3.46740	-2.93840	-0.19430
C	2.23600	1.13020	-0.00220
C	3.11140	2.20750	0.02840
C	2.59580	3.51350	0.09250
C	1.20580	3.68390	0.12440
C	0.41420	2.54170	0.08580
N	0.90890	1.31050	0.02340
H	4.19480	2.09240	0.00360
H	0.73520	4.66590	0.17770
H	-0.67700	2.62650	0.10910
C	2.70840	-0.29400	-0.06610
C	4.06590	-0.60980	-0.05630
C	4.46350	-1.95250	-0.11570
C	2.14810	-2.54060	-0.19500
N	1.76890	-1.25280	-0.12900
H	4.81300	0.17930	0.00110
H	3.73730	-3.99450	-0.24590
H	1.34750	-3.28300	-0.24770
Au	-0.37110	-0.77090	-0.02780
C	-2.47880	-0.30050	0.09310
C	-2.83360	0.34920	-1.10520
C	-2.72000	0.34680	1.32850
C	-3.34100	1.64580	-1.08190
C	-3.22590	1.63390	1.34460
C	-3.53720	2.29970	0.14380
H	-2.50190	-0.16220	2.27090
H	-3.58630	2.13090	-2.02820
H	-3.39690	2.15260	2.29120
I	-2.49200	-2.56850	0.09000
H	-2.71110	-0.15830	-2.06560
O	-4.00980	3.54460	0.26700
C	-4.35540	4.27700	-0.88890
H	-3.48060	4.42640	-1.54770
H	-5.16440	3.78000	-1.45370
H	-4.71160	5.25580	-0.53670
O	3.48560	4.50000	0.11850
O	5.72270	-2.36450	-0.10250
C	6.77530	-1.42180	0.00380
H	7.70550	-2.00690	0.01120
H	6.78440	-0.73530	-0.86060
H	6.69820	-0.84740	0.94330
C	3.03960	5.84180	0.19840
H	3.94560	6.46360	0.20280
H	2.41900	6.10780	-0.67530
H	2.47280	6.01680	1.12970

(OMe-bipy)Au(4-OMe-C_6H_4I)$^+$ product

SCF = –1502.96092 au
ZPE = –1502.66405 au

Au	-0.30470	-0.53120	-0.01510
I	-1.39190	-2.88200	-0.01020
C	1.97660	3.78980	-0.03510
C	0.57280	3.75640	-0.06650
C	-0.06540	2.53710	-0.05780
N	0.60490	1.37280	-0.02350
C	1.95470	1.37860	0.00430
C	2.66950	2.56850	0.00090
H	-0.00060	4.68420	-0.09480
H	-1.15510	2.47620	-0.07940
H	3.75760	2.54860	0.02590
C	4.56010	-1.38070	0.07970
C	3.68880	-2.48200	0.10130
C	2.32940	-2.25500	0.08680
N	1.80230	-1.02160	0.05460
C	2.62160	0.04710	0.03750
C	4.00310	-0.09160	0.04860
H	4.08640	-3.49800	0.12630
H	1.62000	-3.08650	0.09920
H	4.64110	0.78980	0.03390
C	-4.66560	1.48800	-0.20090
C	-3.98700	1.14910	-1.38280
C	-2.76090	0.49570	-1.33300
C	-2.18810	0.18380	-0.09540
C	-2.86260	0.49970	1.08070
C	-4.09740	1.15570	1.03530
H	-4.44540	1.40450	-2.34190
H	-2.25540	0.23860	-2.26840
H	-2.43950	0.24440	2.05660
H	-4.60220	1.39320	1.97390
O	2.55190	4.97740	-0.04200
O	5.85700	-1.63380	0.08970
C	3.96580	5.09070	0.01670
H	4.18130	6.16770	0.00920
H	4.35780	4.64600	0.94720
H	4.43730	4.61800	-0.86180
C	6.79100	-0.56630	0.05040
H	7.78470	-1.03380	0.06370
H	6.67540	0.02260	-0.87560
H	6.68130	0.08500	0.93410
O	-5.84290	2.12140	-0.35200
C	-6.58410	2.48100	0.78940
H	-7.49240	2.98180	0.42310
H	-6.87740	1.59360	1.38020
H	-6.02380	3.18300	1.43480

(^{t}Bu-bipy)Au(C_2H_4)$^+$

SCF = –1023.26070 au
ZPE = –1023.81873 au

Au	-2.38880	0.00040	-0.00000
C	1.70890	2.90010	0.01280
C	0.43270	3.47890	0.04690
C	-0.69190	2.66960	0.05490
N	-0.61480	1.33550	0.03520
C	0.59000	0.74680	0.00830
C	1.76170	1.50290	-0.00650
H	0.29640	4.56200	0.06400
H	-1.69550	3.10140	0.07720
H	2.72390	0.99620	-0.03830
C	1.70810	-2.90050	-0.01270
C	0.43170	-3.47890	-0.04690
C	-0.69270	-2.66930	-0.05500
N	-0.61520	-1.33520	-0.03520
C	0.58980	-0.74690	-0.00820
C	1.76130	-1.50330	0.00660
H	0.29510	-4.56200	-0.06400
H	-1.69640	-3.10090	-0.07730
H	2.72360	-0.99690	0.03830
C	2.95980	3.77940	-0.01450
C	4.25150	2.95230	0.00910
C	2.93380	4.62150	-1.30440
C	2.94550	4.71030	1.21300
C	2.95870	-3.78030	0.01450
C	4.25070	-2.95360	-0.00940
C	2.93260	-4.62220	1.30450
C	2.94390	-4.71130	-1.21280
C	-4.37730	-0.70520	-0.02000
C	-4.37740	0.70580	0.02010
H	-4.58380	-1.22930	-0.96240
H	-4.58370	1.28190	-0.89160
H	-4.58380	1.23000	0.96250
H	-4.58350	-1.28120	0.89170
H	5.12090	3.63160	-0.00340
H	4.32830	2.33240	0.91970
H	4.33700	2.29240	-0.87180
H	3.82730	5.26920	-1.34700
H	2.93630	3.97600	-2.20060
H	2.04380	5.27250	-1.35340
H	3.85510	5.33620	1.21720
H	2.07500	5.38820	1.20890
H	2.92610	4.12990	2.15250
H	5.11980	-3.63330	0.00280
H	4.32750	-2.33360	-0.91980
H	4.33670	-2.29400	0.87160
H	3.82580	-5.27030	1.34700
H	2.93560	-3.97660	2.20060
H	2.04240	-5.27280	1.35370
H	3.85330	-5.33750	-1.21720
H	2.07310	-5.38890	-1.20850
H	2.92450	-4.13100	-2.15250

(^{t}Bu-bipy)Au(4-OMe-C_6H_4I)$^+$ η^2 intermediate

SCF = –1588.31076 au
ZPE = –1587.79792 au

Au	-1.00740	-0.30390	-0.13820
C	3.39960	-2.68810	-0.52480
C	2.19300	-3.36640	-0.74710
C	0.99070	-2.68640	-0.64220
N	0.92490	-1.38550	-0.34030
C	2.05870	-0.70280	-0.12570
C	3.30200	-1.33050	-0.20580
H	2.17130	-4.42810	-1.00160
H	0.04000	-3.20000	-0.80630
H	4.20160	-0.74930	-0.01450
C	2.82190	2.94260	0.70590
C	1.49540	3.38770	0.79620
C	0.45480	2.49680	0.58340
N	0.66170	1.21090	0.29440
C	1.91360	0.75000	0.20200
C	3.00990	1.59150	0.39820
H	1.25700	4.42690	1.03220
H	-0.58690	2.82450	0.64620
H	4.01670	1.18830	0.30780
C	4.73690	-3.42190	-0.64040
C	5.93020	-2.50300	-0.35080
C	4.75570	-4.58770	0.36600
C	4.87150	-3.97210	-2.07330
C	3.98300	3.91090	0.94240
C	5.34710	3.22710	0.79110
C	3.89020	5.05970	-0.07970
C	3.86900	4.47660	2.37070
C	-3.07620	0.25560	-0.06850
C	-3.03760	-1.14010	-0.39490
C	-3.46950	0.63350	1.26590
C	-3.39180	-2.11530	0.59380
H	-3.05320	-1.46040	-1.44160
I	-3.36570	1.70720	-1.61530
C	-3.77040	-0.32230	2.19370
C	-3.73770	-1.71440	1.86710
O	-4.08200	-2.53500	2.86840
C	-4.09410	-3.92560	2.63950
H	-3.09250	-4.29320	2.34890
H	-4.82690	-4.19840	1.85830
H	-4.38900	-4.39350	3.58980
H	-3.53310	1.69250	1.52820
H	-4.06020	-0.04020	3.20920
H	-3.40340	-3.16460	0.29550

H	6.86690	-3.07890	-0.44320
H	5.98730	-1.66260	-1.06440
H	5.89480	-2.09160	0.67300
H	5.71510	-5.12930	0.29150
H	4.65040	-4.22040	1.40220
H	3.94650	-5.31290	0.17410
H	5.83550	-4.49940	-2.18260
H	4.06860	-4.68910	-2.31630
H	4.84160	-3.15620	-2.81710
H	6.14900	3.96450	0.96700
H	5.48280	2.41070	1.52180
H	5.49190	2.81620	-0.22320
H	4.72190	5.76880	0.07760
H	3.95790	4.67770	-1.11390
H	2.94700	5.62390	0.01730
H	4.70570	5.16990	2.56710
H	2.92930	5.03580	2.51910
H	3.91040	3.66870	3.12270

(^{t}Bu-bipy)Au(4-OMe-C_6H_4I)$^+$ transition state

SCF = –1588.29445 au
ZPE = –1587.78312 au

C	2.89760	-3.06880	-0.13550
C	1.71900	1.01280	-0.06370
C	2.60900	2.08370	-0.00400
C	2.13650	3.40510	0.01380
C	0.75030	3.57700	-0.03500
C	-0.07510	2.45700	-0.08850
N	0.39850	1.21720	-0.10050
H	3.68330	1.90140	0.02990
H	0.28950	4.56520	-0.03030
H	-1.16420	2.56380	-0.12320
C	2.16950	-0.41800	-0.08850
C	3.51500	-0.77250	-0.05300
C	3.91630	-2.11580	-0.06420
C	1.57340	-2.65350	-0.17180
N	1.21540	-1.36740	-0.14250
H	4.27340	0.00900	-0.01190
H	3.10500	-4.13870	-0.15600
H	0.76370	-3.38670	-0.22030
Au	-0.91800	-0.84050	-0.06170
C	-3.01340	-0.33830	0.10650
C	-3.39540	0.33380	-1.07040
C	-3.20810	0.29220	1.35840
C	-3.88830	1.63460	-1.01090
C	-3.69920	1.58440	1.41040
C	-4.04180	2.27110	0.23060
H	-2.96790	-0.23380	2.28600
H	-4.15580	2.13750	-1.94180
H	-3.83570	2.08930	2.36990
I	-3.05460	-2.60870	0.07760
H	-3.30510	-0.15870	-2.04190
O	-4.50120	3.51700	0.38730
C	-4.87340	4.27150	-0.74590
H	-4.01490	4.42970	-1.42400
H	-5.69840	3.78770	-1.29900
H	-5.21640	5.24490	-0.36700
C	3.12650	4.57080	0.08860
C	5.40130	-2.47620	0.00670
C	3.93230	4.45620	1.39640
C	2.41690	5.93050	0.07170
C	4.08060	4.49890	-1.11830
C	5.98850	-1.90440	1.31140
C	6.12550	-1.85850	-1.20380
C	5.62370	-3.99350	-0.00700
H	4.64630	5.29540	1.47120
H	3.26690	4.49470	2.27690
H	4.51260	3.51890	1.44670
H	3.16890	6.73660	0.12240
H	1.83270	6.07890	-0.85310
H	1.74090	6.05330	0.93580
H	4.79950	5.33630	-1.07970
H	4.66240	3.56150	-1.13270
H	3.52340	4.57020	-2.06930
H	7.05810	-2.16790	1.38670
H	5.91240	-0.80420	1.35340
H	5.47120	-2.31880	2.19470
H	7.19800	-2.11940	-1.17120
H	5.71010	-2.23970	-2.15340
H	6.05060	-0.75750	-1.21280
H	6.70530	-4.20590	0.04440
H	5.14680	-4.48700	0.85760
H	5.23800	-4.45880	-0.93070

(^{t}Bu-bipy)Au(4-OMe-C_6H_4I)$^+$ product

SCF = –1588.332567 au
ZPE = –1587.818458 au

Au	0.73840	-0.72530	-0.02920
I	1.76520	-3.10360	0.03500
C	-1.44320	3.67010	-0.02450
C	-0.04940	3.58730	-0.05060
C	0.57170	2.34630	-0.06730
N	-0.12020	1.20510	-0.06590
C	-1.47280	1.23990	-0.05710
C	-2.14350	2.45370	-0.03510
H	0.58250	4.47520	-0.05230
H	1.66040	2.26380	-0.07770

H	-3.23340	2.45580	-0.02130
C	-4.16830	-1.44790	-0.05440
C	-3.31450	-2.55770	-0.11030
C	-1.94180	-2.37670	-0.13350
N	-1.38320	-1.16270	-0.10860
C	-2.17130	-0.07220	-0.07190
C	-3.55770	-0.18910	-0.04590
H	-3.70330	-3.57770	-0.12840
H	-1.25740	-3.22830	-0.16640
H	-4.16410	0.71350	-0.01510
C	5.13290	1.20340	0.25490
C	4.41170	0.89390	1.41960
C	3.17510	0.26410	1.33980
C	2.63580	-0.05460	0.08890
C	3.35220	0.23060	-1.06980
C	4.59730	0.86440	-0.99400
H	4.84440	1.15420	2.38930
H	2.63570	0.03040	2.26220
H	2.95500	-0.03120	-2.05480
H	5.13570	1.07930	-1.91910
O	6.31720	1.81580	0.43490
C	7.09260	2.16200	-0.68790
H	7.99750	2.65220	-0.29940
H	7.38990	1.26910	-1.26820
H	6.55790	2.86910	-1.34910
C	-2.20440	4.99390	0.02790
C	-5.68390	-1.63540	0.01030
C	-3.01190	5.03940	1.33930
C	-1.25710	6.19910	-0.01170
C	-3.16170	5.07490	-1.17640
C	-6.43330	-0.29830	0.05850
C	-6.14540	-2.41760	-1.23330
C	-6.01840	-2.43650	1.28350
H	-3.56950	5.99030	1.40000
H	-3.74470	4.21640	1.40130
H	-2.34720	4.97570	2.21870
H	-1.84850	7.12990	0.02480
H	-0.56800	6.21120	0.85060
H	-0.65840	6.22190	-0.93910
H	-3.69290	6.04240	-1.16190
H	-2.60850	5.00290	-2.12940
H	-3.92500	4.27820	-1.15850
H	-7.51890	-0.49060	0.10060
H	-6.16790	0.29240	0.95250
H	-6.24100	0.31490	-0.83880
H	-7.23870	-2.56620	-1.19470
H	-5.90760	-1.86750	-2.16080
H	-5.67480	-3.41370	-1.29390
H	-7.11110	-2.57380	1.36070
H	-5.55440	-3.43760	1.27550
H	-5.67520	-1.90450	2.18840

(COOMe-bipy)Au(C_2H_4)$^+$

SCF = –1164.34380 au
ZPE = –1164.03966 au

Au	-2.47690	0.00020	-0.00000
C	1.59160	-2.89300	0.00070
C	0.33490	-3.49140	0.03660
C	-0.78610	-2.67070	0.04920
N	-0.69050	-1.33990	0.03380
C	0.51330	-0.74610	0.00430
C	1.68450	-1.50180	-0.01940
H	0.23050	-4.57760	0.05310
H	-1.79390	-3.09160	0.07280
H	2.66480	-1.02840	-0.05710
C	1.59190	2.89290	-0.00060
C	0.33530	3.49140	-0.03620
C	-0.78590	2.67080	-0.04860
N	-0.69040	1.34000	-0.03320
C	0.51330	0.74610	-0.00390
C	1.68460	1.50170	0.01960
H	0.23100	4.57760	-0.05260
H	-1.79360	3.09190	-0.07190
H	2.66480	1.02820	0.05710
C	-4.47030	0.70420	-0.02030
C	-4.46990	-0.70470	0.01950
H	-4.67120	1.28090	0.89210
H	-4.67070	-1.22990	0.96250
H	-4.67010	-1.28150	-0.89290
H	-4.67100	1.22920	-0.96340
C	2.81020	-3.77440	-0.01240
C	2.81060	3.77410	0.01230
O	2.75210	-4.97720	-0.00920
O	3.93300	-3.07470	-0.02360
C	5.15670	-3.80690	-0.02930
H	5.95620	-3.05410	-0.03200
H	5.23160	-4.43790	0.87070
H	5.22450	-4.43670	-0.93060
O	2.75260	4.97690	0.00920
O	3.93330	3.07430	0.02330
C	5.15700	3.80640	0.02890
H	5.95650	3.05360	0.03100
H	5.23170	4.43780	-0.87090
H	5.22520	4.43590	0.93040

(COOMe-bipy)Au(4-OMe-C_6H_4I)$^+$ η^2 intermediate

SCF = –1729.39428 au
ZPE = –1729.02076 au

Au	-1.06250	-0.29280	-0.14440
C	3.26130	-2.75460	-0.57890
C	2.05870	-3.42470	-0.78730
C	0.87550	-2.70930	-0.65640
N	0.86020	-1.41060	-0.34610
C	2.00990	-0.74780	-0.14390
C	3.23790	-1.40010	-0.25130
H	2.04350	-4.48490	-1.04560
H	-0.09330	-3.19200	-0.80540
H	4.17600	-0.87180	-0.08700
C	2.81750	2.86050	0.72310
C	1.51970	3.36030	0.80110
C	0.46410	2.48620	0.56770
N	0.66020	1.20180	0.27540
C	1.89980	0.70400	0.19550
C	3.01390	1.51430	0.41670
H	1.33580	4.40950	1.03920
H	-0.57310	2.82900	0.61590
H	4.02750	1.12060	0.35730
C	3.96710	3.79630	0.97480
O	3.82160	4.95310	1.27660
C	-3.12420	0.31530	-0.05500
C	-3.13460	-1.07790	-0.38700
C	-3.47900	0.69780	1.28770
C	-3.49770	-2.04720	0.60060
H	-3.15930	-1.39320	-1.43490
I	-3.37570	1.78620	-1.58740
C	-3.79290	-0.25380	2.21680
C	-3.80900	-1.64330	1.88330
O	-4.15860	-2.45900	2.88530
C	-4.22250	-3.84770	2.64940
H	-3.23920	-4.24750	2.34030
H	-4.97780	-4.09030	1.88010
H	-4.51690	-4.30990	3.60260
H	-3.50590	1.75660	1.55640
H	-4.05580	0.03230	3.23840
H	-3.54370	-3.09410	0.29710
O	5.13960	3.20080	0.82460
C	6.30340	3.99460	1.04310
H	7.15880	3.32850	0.86940
H	6.33020	4.83900	0.33600
H	6.32140	4.37600	2.07650
C	4.54840	-3.51890	-0.72120
O	4.59250	-4.68350	-1.02510
O	5.60710	-2.76420	-0.47680
C	6.88650	-3.38430	-0.58660
H	7.62090	-2.60130	-0.35640
H	6.97460	-4.21290	0.13410
H	7.04300	-3.76480	-1.60850

(COOMe-bipy)Au(4-OMe-C_6H_4I)$^+$ transition state

SCF = –1729.37883 au
ZPE = –1729.00575 au

Au	-0.99070	-0.79140	-0.05210
I	-3.20230	-2.43410	0.06110
C	2.68170	-3.26100	-0.16730
C	1.75270	0.89050	-0.03620
C	2.70670	1.91070	-0.01730
C	2.27180	3.23540	0.02480
C	0.90530	3.50700	0.04510
C	0.02410	2.43070	0.01900
N	0.44820	1.17160	-0.01940
H	3.77500	1.70070	-0.03600
H	0.53960	4.53480	0.08000
H	-1.05960	2.58250	0.03160
C	2.12190	-0.56090	-0.07680
C	3.44990	-0.98810	-0.04270
C	3.73240	-2.35180	-0.08270
C	1.38580	-2.76430	-0.19190
N	1.12060	-1.45500	-0.14310
H	4.27220	-0.27720	0.01990
H	2.86830	-4.33550	-0.20440
H	0.52710	-3.43810	-0.24810
C	-3.04610	-0.15570	0.10510
C	-3.39160	0.53750	-1.07080
C	-3.22190	0.47460	1.35930
C	-3.83470	1.85600	-1.00680
C	-3.66190	1.78510	1.41530
C	-3.97190	2.49110	0.23740
H	-3.00800	-0.06510	2.28540
H	-4.07900	2.37280	-1.93640
H	-3.78460	2.28960	2.37680
H	-3.31410	0.04730	-2.04470
O	-4.38740	3.75130	0.39880
C	-4.73200	4.52250	-0.73220
H	-3.86620	4.65780	-1.40560
H	-5.56870	4.06570	-1.29070
H	-5.04750	5.50380	-0.34980
C	3.23600	4.38820	0.05310
O	2.88830	5.53850	0.13650
O	4.49590	3.98870	-0.02420
C	5.14040	-2.87640	-0.02940
C	5.50290	4.99700	0.00330

O	5.40890	-4.04850	-0.09470
O	6.03060	-1.90640	0.10290
C	7.40180	-2.28910	0.18320
H	6.46070	4.46840	-0.09190
H	5.37080	5.69770	-0.83650
H	5.46790	5.55050	0.95530
H	7.96870	-1.35480	0.29020
H	7.56870	-2.93830	1.05750
H	7.70830	-2.81770	-0.73350

(COOMe-bipy)Au(4-OMe-C_6H_4I)$^+$ product

SCF = –1729.41273 au
ZPE = –1729.03724 au

Au	0.84010	-0.66230	0.01530
I	2.13360	-2.90330	0.02550
C	-1.79310	3.44670	0.00720
C	-0.40560	3.54300	0.01360
C	0.34140	2.37360	0.01180
N	-0.23800	1.17010	0.00320
C	-1.58320	1.05140	-0.00840
C	-2.38910	2.18620	-0.00490
H	0.09230	4.51370	0.02080
H	1.43270	2.39870	0.01840
H	-3.47500	2.10210	-0.01340
C	-3.92650	-1.92500	-0.06280
C	-2.98160	-2.94530	-0.09070
C	-1.63500	-2.60250	-0.08260
N	-1.23240	-1.33150	-0.05150
C	-2.13290	-0.32880	-0.03180
C	-3.49780	-0.59780	-0.03620
H	-3.28520	-3.99320	-0.11490
H	-0.85250	-3.36570	-0.09820
H	-4.23190	0.20680	-0.01770
C	5.02210	1.69830	0.19890
C	4.34360	1.35520	1.37990
C	3.17330	0.60660	1.33180
C	2.65730	0.20510	0.09540
C	3.32870	0.52800	-1.08000
C	4.50750	1.27950	-1.03470
H	4.75800	1.68140	2.33740
H	2.66810	0.34500	2.26610
H	2.94970	0.20200	-2.05270
H	5.01350	1.52080	-1.97140
O	6.14780	2.41810	0.34800
C	6.89750	2.77000	-0.79090
H	7.77050	3.33020	-0.42510
H	7.24850	1.87560	-1.33800
H	6.31760	3.41600	-1.47600
C	-2.60200	4.71630	0.01180
C	-5.38640	-2.29090	-0.06140
O	-2.09960	5.81020	0.01250
O	-5.77770	-3.42900	-0.08130
O	-3.90210	4.48020	0.01560
C	-4.76650	5.61580	0.02000
O	-6.16580	-1.22360	-0.03490
C	-7.57480	-1.44620	-0.02480
H	-8.03600	-0.45040	0.00670
H	-7.88530	-1.98160	-0.93600
H	-7.86550	-2.02780	0.86440
H	-5.78890	5.21590	0.02550
H	-4.59130	6.22680	0.91950
H	-4.60050	6.22650	-0.88140

(CF_3-bipy)Au(C_2H_4)$^+$

SCF = –1382.46738 au
ZPE = –1382.24049 au

Au	2.36470	-0.00050	0.00000
C	-1.70390	2.88750	-0.00910
C	-0.45070	3.49260	0.02530
C	0.66870	2.67050	0.04030
N	0.57260	1.33960	0.02940
C	-0.62920	0.74620	0.00130
C	-1.80210	1.50120	-0.02550
H	-0.33340	4.57810	0.03850
H	1.67610	3.09220	0.06230
H	-2.78100	1.02500	-0.06300
C	-1.70460	-2.88700	0.00910
C	-0.45170	-3.49250	-0.02550
C	0.66800	-2.67070	-0.04060
N	0.57220	-1.33970	-0.02960
C	-0.62940	-0.74600	-0.00140
C	-1.80250	-1.50070	0.02550
H	-0.33460	-4.57800	-0.03880
H	1.67520	-3.09260	-0.06270
H	-2.78120	-1.02420	0.06310
C	-2.94460	3.75530	-0.01270
F	-4.06060	3.03840	-0.13230
F	-3.03200	4.45590	1.12020
F	-2.91310	4.62890	-1.02040
C	-2.94560	-3.75450	0.01270
F	-4.06140	-3.03730	0.13280
F	-3.03350	-4.45490	-1.12030
F	-2.91410	-4.62830	1.02030
C	4.35990	-0.70390	-0.01790
C	4.35900	0.70440	0.01810
H	4.55900	-1.27830	0.89630
H	4.55770	1.23240	0.95990

H	4.55760	1.27900	-0.89610
H	4.55920	-1.23160	-0.95970

(CF_3-bipy)Au(4-OMe-C_6H_4I)$^+$ η^2 intermediate

SCF = –1947.51823 au
ZPE = –1947.22142 au

Au	-0.99480	-0.32030	-0.15110
C	3.38210	-2.68440	-0.51260
C	2.20020	-3.38690	-0.72790
C	1.00310	-2.69150	-0.62250
N	0.95850	-1.38960	-0.32860
C	2.09070	-0.70170	-0.12160
C	3.33410	-1.32990	-0.20670
H	2.19600	-4.45130	-0.97090
H	0.04670	-3.19610	-0.77760
H	4.25800	-0.77760	-0.03970
C	2.81570	2.92490	0.71280
C	1.51140	3.41060	0.75780
C	0.47720	2.51330	0.51950
N	0.70180	1.22870	0.24970
C	1.94880	0.75190	0.19910
C	3.04650	1.58330	0.43210
H	1.29410	4.45880	0.97370
H	-0.56630	2.83870	0.54390
H	4.06700	1.20410	0.39950
C	4.70800	-3.40210	-0.64640
F	5.72460	-2.66410	-0.20390
F	4.70620	-4.54560	0.03990
F	4.95250	-3.70360	-1.92400
C	3.96630	3.87190	0.97890
F	5.15040	3.27620	0.84650
F	3.93660	4.90670	0.13650
F	3.89750	4.36510	2.21750
C	-3.06460	0.26320	-0.07160
C	-3.06410	-1.13180	-0.39390
C	-3.42280	0.65160	1.26820
C	-3.42050	-2.09740	0.59810
H	-3.07840	-1.45440	-1.43970
I	-3.31960	1.72250	-1.61490
C	-3.73150	-0.29660	2.20300
C	-3.73780	-1.68770	1.87820
O	-4.08330	-2.49990	2.88390
C	-4.13770	-3.89070	2.65640
H	-3.15100	-4.28580	2.35240
H	-4.88930	-4.14270	1.88650
H	-4.43170	-4.34870	3.61160
H	-3.45770	1.71190	1.53030

H	-3.99790	-0.00590	3.22240
H	-3.45750	-3.14650	0.30120

(CF_3-bipy)Au(4-OMe-C_6H_4I)$^+$ transition state

SCF = –1947.50295 au
ZPE = –1947.20706 au

C	-3.01220	-2.99870	0.13070
C	-1.72880	1.05980	0.02840
C	-2.58640	2.15980	-0.00010
C	-2.02840	3.43770	-0.02340
C	-0.64780	3.59310	-0.01590
C	0.13120	2.43800	0.01340
N	-0.40440	1.22460	0.03430
H	-3.67090	2.04490	-0.00420
H	-0.17640	4.57670	-0.03290
H	1.22420	2.49250	0.01920
C	-2.22130	-0.35400	0.05810
C	-3.57920	-0.66460	0.02180
C	-3.97310	-2.00010	0.05340
C	-1.67700	-2.61120	0.15880
N	-1.29950	-1.33190	0.11870
H	-4.33470	0.12000	-0.03430
H	-3.27420	-4.05720	0.16120
H	-0.88220	-3.35960	0.21040
Au	0.86610	-0.86150	0.03870
C	2.95950	-0.37930	-0.09740
C	3.35390	0.27390	1.08530
C	3.20990	0.23540	-1.34640
C	3.91880	1.54560	1.03290
C	3.77260	1.49850	-1.39130
C	4.13040	2.16930	-0.20650
H	2.95940	-0.27950	-2.27750
H	4.19820	2.03420	1.96790
H	3.95520	1.99200	-2.34910
I	2.93470	-2.67100	-0.05970
H	3.22020	-0.21080	2.05580
O	4.66180	3.38650	-0.35660
C	5.05350	4.12300	0.78230
H	4.19510	4.32040	1.44990
H	5.84870	3.60130	1.34450
H	5.44570	5.07950	0.40830
C	-2.95490	4.63340	-0.07300
C	-5.45140	-2.32690	0.01420
F	-3.64350	4.65530	-1.21790
F	-2.29430	5.78520	0.02210
F	-3.84500	4.59340	0.92210
F	-6.04260	-1.73690	-1.02740

F	-6.06420	-1.89090	1.11760
F	-5.67190	-3.63540	-0.08170

$(CF_3$-bipy)Au(4-OMe-$C_6H_4I)^+$ product

SCF = –1947.53507 au
ZPE = –1947.23713 au

Au	0.72190	-0.72270	0.01440
I	1.76420	-3.08720	0.03010
C	-1.44900	3.65020	0.02650
C	-0.06330	3.60250	0.05020
C	0.55000	2.35440	0.04310
N	-0.15460	1.22290	0.01620
C	-1.50500	1.25160	-0.00620
C	-2.18200	2.46490	-0.00210
H	0.55020	4.50390	0.07430
H	1.63780	2.26230	0.06130
H	-3.27200	2.49580	-0.02030
C	-4.15830	-1.44190	-0.07430
C	-3.33840	-2.56550	-0.09500
C	-1.96300	-2.37250	-0.08060
N	-1.42220	-1.15340	-0.04980
C	-2.20410	-0.05900	-0.03390
C	-3.59180	-0.17210	-0.04410
H	-3.74590	-3.57810	-0.12050
H	-1.27020	-3.21780	-0.09320
H	-4.22740	0.71260	-0.03080
C	5.11650	1.20560	0.18460
C	4.43560	0.88130	1.36970
C	3.20050	0.24500	1.32590
C	2.62420	-0.05950	0.08870
C	3.29860	0.23740	-1.09180
C	4.54210	0.87640	-1.04990
H	4.89990	1.13290	2.32690
H	2.69320	-0.00340	2.26230
H	2.87050	-0.01880	-2.06510
H	5.04930	1.10210	-1.99000
O	6.30220	1.82150	0.33130
C	7.04720	2.16880	-0.81230
H	7.96230	2.65780	-0.44750
H	7.32750	1.27590	-1.40100
H	6.49530	2.87700	-1.45790
C	-2.19890	4.96740	0.02650
C	-5.66370	-1.61880	-0.06290
F	-3.05040	5.02440	1.05180
F	-1.37960	6.01000	0.11640
F	-2.91080	5.10030	-1.09370
F	-6.30350	-0.46380	-0.23350
F	-6.05310	-2.44780	-1.03060
F	-6.06560	-2.13890	1.09810

5.3.3. Comparison with experimental rate data

The oxidative addition barriers ($\Delta G^{\ddagger}$) may be calculated from the observed rates (k_{obs}) given in Table 1 using the Eyring equation (Equation 4) which is rearranged to give Equation 5. k_B = 1.38×10^{-23} J K^{-1} is the Boltzmann constant, T is the temperature in K, $h = 6.626 \times 10^{-34}$ J s is Planck's constant and $R = 8.314$ J K^{-1} mol^{-1} is the gas constant. Free energies were then converted to kcal mol^{-1}.

$$k_{obs} = \frac{k_B T}{h} exp\left(-\frac{\Delta G^{\ddagger}}{RT}\right) \quad (4)$$

$$\Delta G^{\ddagger} = -RTln\left\{k_{obs}\left(\frac{h}{k_B T}\right)\right\} \quad (5)$$

Table S6: Comparison of Gibbs free energies of experimental and calculated oxidative addition barriers.

Ligand subsituent	k_{obs} / s^{-1}	$\Delta G^{\ddagger}$ / kcal mol^{-1}
**Experimental oxidative addition barriers**		
H	6.6×10^{-7}	28.1
EDG (p-^{t}Bu)	4.3×10^{-7}	28.4
EWG (p-CF_3)	1.9×10^{-6}	27.4
**Calculated oxidative addition barriers**		
H	-	27.1
EDG (p-^{t}Bu)	-	28.7
EWG (p-CF_3)	-	26.7

The calculated Gibbs free energy for the oxidative addition barriers show good agreement compared to the experimentally obtained values. Differences in the Gibbs free energy for the representative examples of electron-donating groups (EDG) and electron-withdrawing groups (EWG) are small for both experimental and calculated sets. The small differences observed in the experimental data (also consistent with the small value of ρ in the Hammett study) show that the computational model is a reasonable depiction of differences in barriers between EDGs and EWGs. Further studies are currently in progress to further explain the stark differences in rate of oxidative addition with different ligand substituents (*i.e.*, with *p*-substiuted complex **2i•NTf$_2$**).

5.4. *Oxidative addition with 4-vinyliodobenzene*

5.4.1. *Calculated potential energy surface*

The calculated potential energy surface given in Figure S10 below broadly follows the same profile as the previously calculated examples.[5] There is a clear preference for the formation of the vinyl-π-bound intermediate given in gray *vs* the η^2-π-bound intermediate (ΔE = 17.5 kcal mol^{-1}). This additional equilibrium in solution between competing π -bound species would lead to a slower oxidative addition. The geometry of the calculated vinyl-π-bound intermediate in Figure S10 inset (B) is consistent with related structures that have been isolated in the literature.[23]

Figure S10: Calculated potential energy surface for the oxidative addition of 4-vinyliodobenzene (**3i**). See Section 5.1 for computational details. Inset (A) transition state geometry and inset (B) geometry of vinyl-π-bound intermediate with selected bond lengths given in Å.

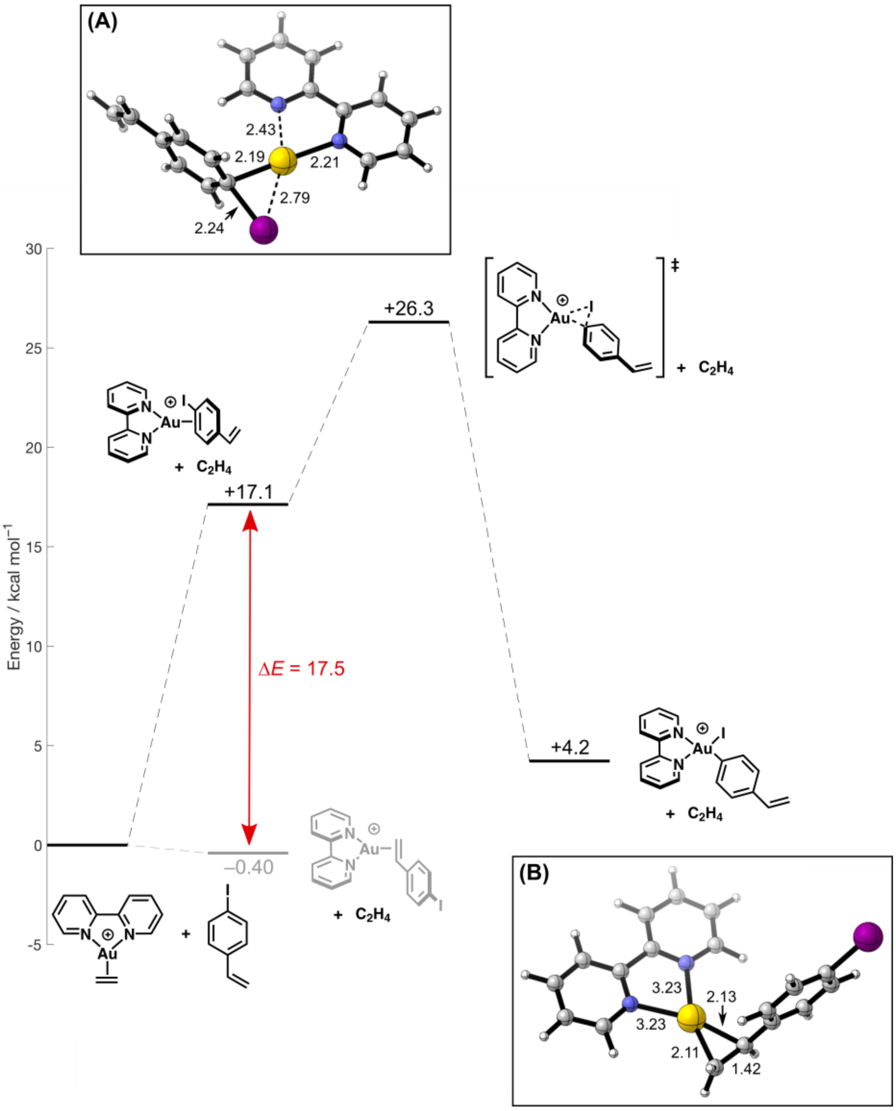

5.4.2. Energies and Cartesian coordinates (Å) for stationary points

C_2H_3-C_6H_4I

SCF = –606.48590 au
ZPE = –606.36173 au

C	0.00000	-0.16810	0.00000
C	-1.38980	-0.05230	0.00000
C	-1.97000	1.21560	0.00000
C	-1.18880	2.38120	0.00000
C	0.20840	2.23510	0.00000
C	0.80230	0.97630	0.00000
I	0.89720	-2.07500	0.00000
H	-2.02620	-0.94090	0.00000
H	-3.06150	1.29920	0.00000
C	-1.86540	3.69470	0.00000
H	0.85760	3.11500	0.00000
H	1.89230	0.89330	0.00000
C	-1.28840	4.90260	0.00000
H	-1.90270	5.81030	0.00000
H	-0.20110	5.04620	0.00000
H	-2.96180	3.64300	0.00000

Au(bipy)(C_2H_3-C_6H_4I)$^+$ η^2-vinyl bound

SCF = –1237.06726 au
ZPE= –1236.77810 au

C	-1.21250	3.53490	-1.27220
C	-3.84010	0.85770	0.71520
C	-5.00050	1.21690	1.40100
C	-5.76310	0.22410	2.01130
C	-5.35280	-1.10240	1.92270
C	-4.18570	-1.38500	1.22220
N	-3.45770	-0.42870	0.64010
H	-5.31640	2.25810	1.46680
H	-6.67370	0.49130	2.55360
H	-5.92090	-1.91120	2.38660
H	-3.81780	-2.40940	1.12280
C	-2.95630	1.84850	0.03260
C	-3.21120	3.22000	0.02490
C	-2.32840	4.07040	-0.63670
C	-1.02360	2.15850	-1.22170
N	-1.87480	1.34780	-0.58830
H	-4.08600	3.63380	0.52700
H	-2.51630	5.14690	-0.65120
H	-0.49410	4.16580	-1.79940
H	-0.16210	1.68680	-1.70170
Au	-1.57700	-0.85710	-0.47470
C	0.10120	-1.83940	-1.34500
C	-0.73420	-2.77490	-0.68830
H	-0.45450	-3.20060	0.28370
H	-1.39740	-3.40360	-1.29530
H	-0.00280	-1.75670	-2.43440
C	3.84120	-0.26360	0.06240
C	3.45200	-0.08730	-1.26520
C	2.23510	-0.61240	-1.69860
C	1.39400	-1.31480	-0.82580
C	1.80690	-1.48260	0.50440
C	3.01930	-0.96410	0.94980
I	5.67520	0.52650	0.73260
H	4.08980	0.45400	-1.96870
H	1.93700	-0.47290	-2.74250
H	1.17590	-2.02270	1.21570
H	3.31840	-1.10870	1.99110

Au(bipy)(C_2H_3-C_6H_4I)$^+$ η^2-arene bound

SCF = –1237.03689 au
ZPE = –1236.75016 au

C	3.47850	2.79140	-1.57880
C	2.95600	-0.85170	0.54310
C	3.99030	-1.66210	1.01420
C	3.67440	-2.83100	1.70260
C	2.33850	-3.16490	1.90430
C	1.36470	-2.30840	1.40180
N	1.67450	-1.19100	0.74310
H	5.03500	-1.39590	0.85390
H	4.47350	-3.47560	2.07780
H	2.04830	-4.07100	2.44000
H	0.29980	-2.52430	1.52820
C	3.19670	0.42240	-0.20070
C	4.47600	0.92580	-0.44030
C	4.61620	2.12320	-1.13710
C	2.23730	2.22910	-1.30370
N	2.10910	1.08000	-0.63530
H	5.36370	0.39830	-0.09030
H	5.61270	2.52870	-1.33020
H	3.54250	3.73320	-2.12750
H	1.31260	2.71410	-1.62710
Au	0.10530	0.19620	-0.16000
C	-2.48310	2.42770	1.08000
C	-2.12250	2.26860	-0.24300
C	-2.62980	1.26000	1.89120
H	-2.93610	1.37830	2.93500
C	-1.85400	0.97710	-0.79420
C	-2.41900	-0.00230	1.39970
H	-2.57600	-0.87670	2.03580
C	-2.00970	-0.18540	0.03430

I	-2.41170	-2.08210	-0.86230
H	-1.84010	0.87970	-1.88460
C	-2.74100	3.74840	1.68880
H	-2.07150	3.12810	-0.91640
C	-2.33910	4.93180	1.21010
H	-2.58540	5.85930	1.73950
H	-1.75010	5.02820	0.28940
H	-3.29940	3.72450	2.63320

Au(bipy)(C_2H_3-C_6H_4I)$^+$ transition state

SCF = –1237.02171 au
ZPE = –1236.73554 au

C	4.61770	-1.48530	0.10420
C	2.27450	2.06690	-0.04800
C	2.80280	3.36100	-0.06870
C	1.92900	4.44530	-0.11110
C	0.55610	4.21670	-0.13230
C	0.11290	2.89710	-0.10980
N	0.95360	1.86700	-0.06860
H	3.87790	3.53900	-0.04950
H	-0.16280	5.03830	-0.16550
H	-0.95390	2.65410	-0.12760
C	3.13550	0.84130	-0.00170
C	4.52980	0.90880	-0.05170
C	5.27660	-0.26440	0.00110
C	3.22950	-1.47880	0.14590
N	2.51600	-0.34860	0.09310
H	5.04230	1.86720	-0.13500
H	5.16110	-2.43110	0.15010
H	2.66420	-2.41140	0.22280
Au	0.30560	-0.47060	0.06300
C	-1.87660	-0.65800	0.04440
C	-2.34110	0.00580	-1.11340
C	-2.30930	-0.22620	1.31440
C	-3.15490	1.12170	-0.98730
C	-3.12540	0.89660	1.41350
C	-3.55650	1.59620	0.27560
H	-2.00980	-0.76560	2.21640
H	-3.48790	1.62630	-1.89770
I	-1.22030	-2.78990	-0.16690
H	-2.05770	-0.35260	-2.10620
H	6.36790	-0.21950	-0.03910
H	2.32540	5.46380	-0.12650
C	-4.39420	2.79700	0.44960
H	-3.43820	1.23620	2.40550
C	-4.78360	3.64660	-0.50930
H	-5.40440	4.51500	-0.26140
H	-4.50890	3.52250	-1.56390
H	-4.70270	2.99660	1.48330

Au(bipy)(C_2H_3-C_6H_4I)$^+$ product

SCF = –1237.05941 au
ZPE = –1236.82305 au

Au	-0.30930	-0.20960	0.00760
I	0.11090	-2.76370	0.04000
C	-1.34560	4.55630	0.10520
C	-0.01140	4.16720	0.12040
C	0.28470	2.81140	0.07880
N	-0.67550	1.88230	0.02450
C	-1.98030	2.23790	0.00420
C	-2.33940	3.58250	0.04460
H	0.80060	4.89540	0.16440
H	1.31680	2.45620	0.09020
H	-3.38780	3.87950	0.03160
C	-5.20760	0.25650	-0.17810
C	-4.67600	-1.02790	-0.17330
C	-3.29500	-1.17280	-0.11410
N	-2.47590	-0.11980	-0.06010
C	-2.97360	1.13440	-0.06180
C	-4.34760	1.35130	-0.12190
H	-5.31260	-1.91370	-0.21550
H	-2.82370	-2.15910	-0.10960
H	-4.75610	2.36160	-0.12480
C	4.43330	0.60370	0.13680
C	3.70030	0.43040	1.32030
C	2.34210	0.11060	1.29590
C	1.69630	-0.03120	0.06860
C	2.40840	0.10990	-1.12520
C	3.76390	0.42880	-1.08580
H	4.20270	0.54820	2.28570
H	1.80100	-0.01880	2.23730
H	1.91600	-0.02300	-2.09250
H	4.30300	0.53610	-2.03120
H	-1.61970	5.61340	0.13910
H	-6.28770	0.41470	-0.22560
C	5.86600	0.95390	0.22870
C	6.69470	1.23280	-0.78450
H	7.74450	1.48030	-0.58990
H	6.37540	1.22950	-1.83370
H	6.26320	0.98590	1.25110

5.5. Additional aryl iodides

5.5.1. Full potential energy surface comparison

Figure S11: Calculated potential energy surfaces for *p*-OMe, *p*-^{t}Bu, *p*-H, *p*-CHO and *p*-CF_3 subsituted aryl iodides. See Section 5.1 for computational details.

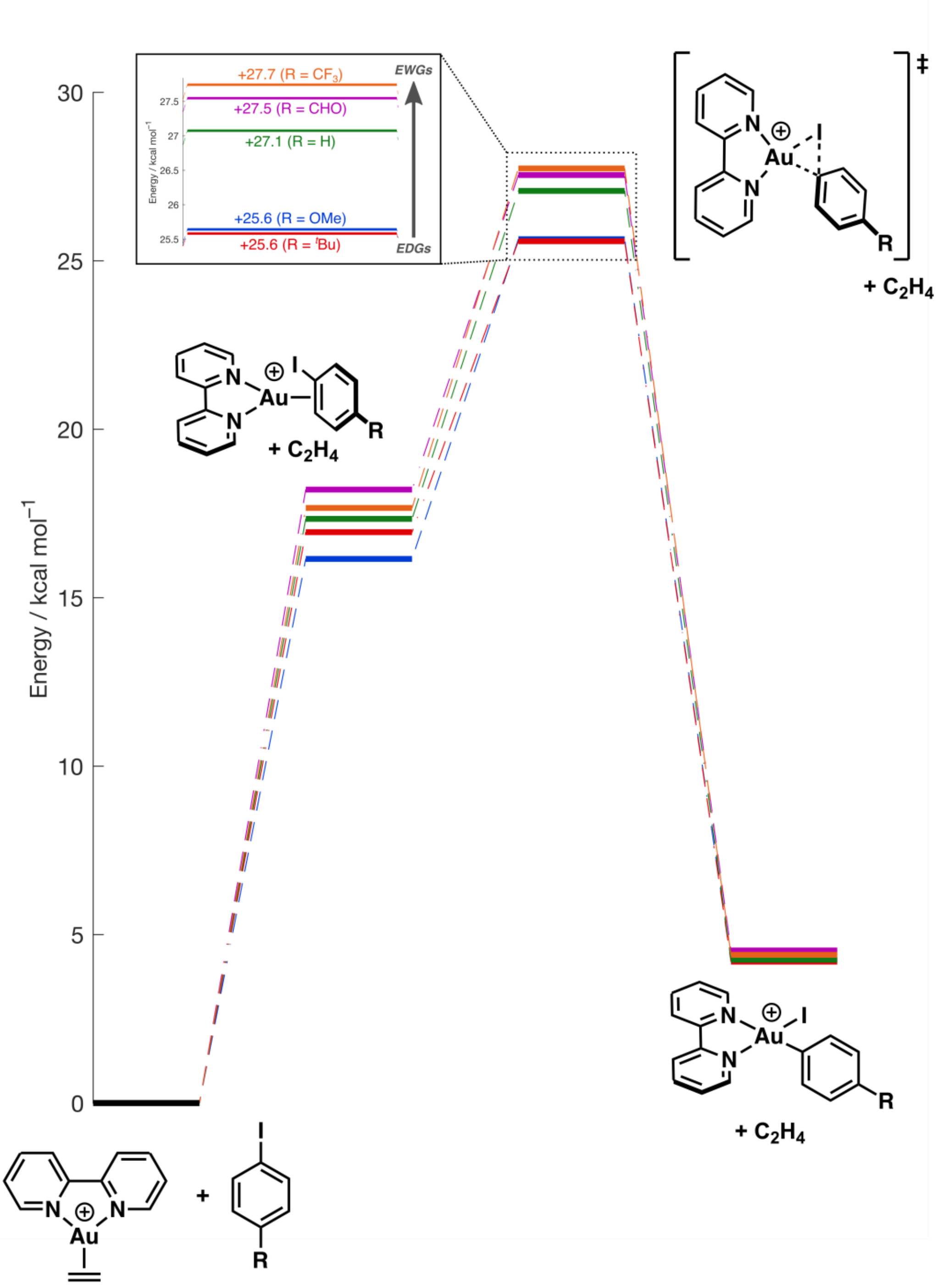

5.5.2. Energies and Cartesian coordinates (Å) for stationary points

(bipy)Au(4-^{t}Bu-C_6H_4I)$^+$ η^2 intermediate

SCF = –1316.81251 au
ZPE = –1316.44589 au

Au	-0.32980	0.01490	-0.27250
C	-3.82630	3.42930	-1.37260
C	-2.57460	3.51590	-1.97440
C	-1.62210	2.55640	-1.65070
N	-1.87180	1.56850	-0.78770
C	-3.07240	1.47280	-0.19380
C	-4.07980	2.39790	-0.47190
H	-2.33240	4.30960	-2.68410
H	-0.62350	2.57770	-2.09510
H	-5.05880	2.32400	0.00210
C	-4.52290	-0.94920	2.35000
C	-3.42850	-1.79640	2.49520
C	-2.28250	-1.52040	1.75700
N	-2.20850	-0.48200	0.92340
C	-3.25480	0.34120	0.76670
C	-4.43870	0.13220	1.47630
H	-3.45300	-2.65550	3.16870
H	-1.39250	-2.15170	1.83330
H	-5.29170	0.80070	1.36070
C	1.48370	-1.15400	-0.13670
C	1.70750	-0.11070	-1.09410
C	2.06220	-1.01010	1.16640
C	2.50240	1.01660	-0.72020
H	1.54960	-0.29510	-2.16180
I	1.01690	-3.13150	-0.79470
C	2.79130	0.11070	1.48420
C	3.03690	1.15540	0.54430
H	1.93160	-1.80580	1.90390
H	3.20550	0.17390	2.49170
H	2.69510	1.76420	-1.49430
H	-5.44340	-1.12560	2.91240
H	-4.60670	4.16010	-1.59890
C	3.88200	2.39070	0.88970
C	4.35610	2.38130	2.34900
C	5.12220	2.42510	-0.02270
C	3.03720	3.65810	0.66140
H	4.94770	3.29120	2.54990
H	3.51010	2.37240	3.05870
H	5.00200	1.51320	2.56760
H	5.74720	3.30470	0.21490
H	5.73950	1.52000	0.11720
H	4.84740	2.48820	-1.08980
H	3.62270	4.55900	0.91840
H	2.71910	3.75570	-0.39090
H	2.12970	3.64990	1.29190

(bipy)Au(4-^{t}Bu-C_6H_4I)$^+$ transition state

SCF = –1316.79777 au
ZPE = –1316.43213 au

C	5.19700	0.06640	0.36980
C	1.80820	2.59910	-0.09170
C	1.85020	3.99530	-0.04470
C	0.66580	4.70970	-0.21200
C	-0.52590	4.02060	-0.42020
C	-0.48210	2.62950	-0.44420
N	0.65310	1.95430	-0.28270
H	2.78390	4.53210	0.12660
H	-1.47360	4.54530	-0.55940
H	-1.38650	2.03220	-0.60090
C	3.02850	1.74570	0.06660
C	4.31610	2.28450	0.10260
C	5.40950	1.43670	0.25590
C	3.88980	-0.40070	0.31730
N	2.84320	0.41820	0.16870
H	4.47670	3.35810	0.00410
H	6.02340	-0.63640	0.49280
H	3.66790	-1.46850	0.39530
Au	0.80130	-0.43210	-0.00540
C	-1.20500	-1.29050	-0.01460
C	-1.87450	-0.89250	-1.18990
C	-1.76070	-0.97810	1.23790
C	-3.03310	-0.13490	-1.09100
C	-2.92290	-0.20820	1.30440
C	-3.58180	0.23480	0.15160
H	-1.28940	-1.32830	2.15970
H	-3.51840	0.17740	-2.01980
H	-3.31040	0.03380	2.29530
I	0.16370	-3.10140	-0.13730
H	-1.48220	-1.16780	-2.17200
H	0.67930	5.80230	-0.17800
H	6.42110	1.84960	0.28300
C	-4.84610	1.10140	0.19500
C	-5.29970	1.39380	1.63110
C	-4.55230	2.44350	-0.50340
C	-5.98850	0.37500	-0.53910
H	-6.20840	2.02040	1.61140
H	-5.54700	0.46960	2.18180
H	-4.53090	1.94320	2.20230
H	-5.45120	3.08550	-0.48840
H	-3.74020	2.98750	0.01170
H	-4.25860	2.30680	-1.55860

H	-6.90770	0.98760	-0.51440
H	-5.74550	0.18240	-1.59820
H	-6.21210	-0.59480	-0.06000

(bipy)Au(4-^{t}Bu-C_6H_4I)$^+$ product

SCF = –1316.83458 au
ZPE = –1316.46621 au

Au	-0.73080	-0.22850	-0.00120
I	-0.50140	-2.81050	-0.02110
C	-1.39170	4.60650	-0.03020
C	-0.09230	4.11340	-0.01090
C	0.09480	2.73780	0.00430
N	-0.93620	1.88630	0.00560
C	-2.20870	2.34340	-0.00440
C	-2.45940	3.71260	-0.02780
H	0.77530	4.77580	-0.00990
H	1.09550	2.30160	0.01500
H	-3.48220	4.08780	-0.04560
C	-5.58700	0.62260	0.04880
C	-5.15850	-0.69960	0.02440
C	-3.79200	-0.95220	0.00100
N	-2.89190	0.03360	-0.00380
C	-3.28890	1.32280	0.01180
C	-4.64300	1.64750	0.04360
H	-5.86470	-1.53210	0.02600
H	-3.39920	-1.97220	-0.01360
H	-4.97170	2.68650	0.06690
C	4.07010	0.24920	0.04140
C	3.34820	0.07740	1.22850
C	1.96800	-0.14920	1.22220
C	1.28390	-0.19720	0.01130
C	1.97820	-0.05310	-1.19090
C	3.35330	0.17340	-1.16440
H	3.85120	0.11610	2.19670
H	1.44330	-0.28060	2.17270
H	1.46000	-0.10680	-2.15260
H	3.87340	0.29120	-2.11950
H	-1.58210	5.68230	-0.04700
H	-6.65250	0.86400	0.07230
C	5.58400	0.50610	0.01400
C	6.18260	0.59440	1.42390
C	6.27910	-0.64430	-0.73830
C	5.86140	1.83510	-0.71350
H	7.26700	0.79010	1.35430
H	5.73440	1.41550	2.01050
H	6.05150	-0.34590	1.98710
H	7.37110	-0.47810	-0.76660
H	6.09370	-1.61230	-0.23960
H	5.92640	-0.72600	-1.78120
H	6.94690	2.04070	-0.73190
H	5.50840	1.81450	-1.75900
H	5.36450	2.67900	-0.20200

(bipy)Au(C_6H_5I)$^+$ η^2 intermediate

SCF = –1159.72260 au
ZPE = –1159.46892 au

C	-3.82560	-2.58720	-1.10080
C	-2.64720	1.26250	0.25880
C	-3.50880	2.33880	0.47880
C	-2.98320	3.54510	0.93570
C	-1.61410	3.65180	1.16060
C	-0.82010	2.53660	0.91610
N	-1.33010	1.38400	0.47990
H	-4.58080	2.25290	0.30070
H	-3.64570	4.39600	1.11370
H	-1.16140	4.57850	1.51890
H	0.26180	2.56570	1.07420
C	-3.11720	-0.07190	-0.22510
C	-4.45880	-0.35160	-0.48970
C	-4.81440	-1.62310	-0.93210
C	-2.51060	-2.23580	-0.81880
N	-2.17540	-1.01440	-0.39540
H	-5.23050	0.40630	-0.35480
H	-5.86190	-1.85460	-1.14150
H	-4.06060	-3.59690	-1.44360
H	-1.69610	-2.95590	-0.93280
Au	-0.05390	-0.45200	0.05770
C	2.05370	-2.66630	2.03830
C	1.71640	-2.84300	0.72050
C	2.41560	-1.37930	2.52090
H	2.69150	-1.25560	3.57180
C	1.71000	-1.73300	-0.18890
C	2.45210	-0.29190	1.68380
H	2.77020	0.68550	2.05460
C	2.09270	-0.43720	0.29890
I	2.85260	1.02870	-1.06110
H	1.72990	-1.94800	-1.26260
H	1.47890	-3.83610	0.32970
H	2.06780	-3.52010	2.72100

(bipy)Au(C_6H_5I)$^+$ transition state

SCF = –1159.70656 au
ZPE = –1159.45341 au

C	3.08970	-3.31730	0.47290

C	2.87680	0.89010	-0.13490
C	3.98490	1.74140	-0.16790
C	3.78220	3.10470	-0.37010
C	2.48660	3.58550	-0.53620
C	1.43770	2.67140	-0.48890
N	1.63930	1.37080	-0.29460
H	4.99810	1.36180	-0.03280
H	2.28550	4.64680	-0.69750
H	0.39730	2.99000	-0.61190
C	2.99900	-0.58770	0.07800
C	4.23610	-1.22810	0.17820
C	4.28100	-2.60460	0.37910
C	1.89590	-2.61610	0.35970
N	1.85900	-1.29310	0.17000
H	5.16640	-0.66610	0.09660
H	3.07630	-4.39750	0.63070
H	0.93250	-3.12900	0.42290
Au	-0.10320	-0.25640	-0.00510
C	-2.07660	0.69020	0.15970
C	-2.22490	1.65030	-0.86290
C	-2.17010	1.07180	1.51370
C	-2.40690	2.98660	-0.52010
C	-2.34270	2.41940	1.83130
C	-2.46370	3.37440	0.82220
H	-2.11600	0.32310	2.30800
I	-2.55760	-1.45850	-0.33820
H	5.24480	-3.11380	0.45940
H	4.63720	3.78540	-0.39550
H	-2.39940	2.71650	2.88220
H	-2.19360	1.34860	-1.91290
H	-2.61370	4.42630	1.08060
H	-2.50810	3.73260	-1.31350

(bipy)Au(C_6H_5I)$^+$ product

SCF = –1159.74544 au
ZPE = –1159.48977 au

Au	0.09760	-0.11370	-0.00710
I	1.43700	-2.33280	0.00600
C	-2.64780	3.91530	-0.02070
C	-1.26480	4.05300	-0.04160
C	-0.48400	2.90500	-0.04040
N	-1.02710	1.68300	-0.02000
C	-2.37050	1.52500	-0.00440
C	-3.20540	2.63930	-0.00280
H	-0.78320	5.03220	-0.05790
H	0.60620	2.96050	-0.05580
H	-4.28880	2.52240	0.01230
C	-4.62160	-1.52620	0.02840
C	-3.64630	-2.51650	0.00820
C	-2.31120	-2.13060	-0.00880
N	-1.94750	-0.84600	-0.00860
C	-2.87970	0.12920	0.00640
C	-4.23550	-0.18750	0.02690
H	-3.90400	-3.57730	0.00700
H	-1.50330	-2.86710	-0.02190
H	-4.99490	0.59430	0.04050
C	4.19210	2.37370	0.02760
C	3.56930	2.03230	1.22870
C	2.41830	1.23980	1.22630
C	1.89170	0.80380	0.00910
C	2.51510	1.12620	-1.19750
C	3.66610	1.91830	-1.18210
H	1.94020	0.97450	2.17360
H	2.11470	0.76970	-2.15060
H	4.15550	2.17480	-2.12650
H	-3.29720	4.79400	-0.01920
H	-5.68270	-1.78670	0.04450
H	5.09490	2.99120	0.03470
H	3.98120	2.37990	2.18090

(bipy)Au(4-CHO-C_6H_4I)$^+$ η^2 intermediate

SCF = –1272.92653 au
ZPE = –1272.66316 au

Au	-0.11430	0.20680	-0.14550
C	-4.62070	2.11190	-1.17070
C	-3.48440	2.79230	-1.59710
C	-2.24070	2.24570	-1.30180
N	-2.11000	1.10060	-0.62730
C	-3.19610	0.43260	-0.20490
C	-4.47740	0.91920	-0.46610
H	-3.55120	3.73130	-2.15020
H	-1.31700	2.74030	-1.61320
H	-5.36370	0.38300	-0.12640
C	-3.66100	-2.80160	1.73300
C	-2.32410	-3.13450	1.92880
C	-1.35290	-2.28440	1.41170
N	-1.66630	-1.17260	0.74400
C	-2.94990	-0.83400	0.54880
C	-3.98060	-1.63950	1.03500
H	-2.03070	-4.03550	2.47140
H	-0.28770	-2.50070	1.53310
H	-5.02620	-1.37420	0.87840
C	2.00080	-0.18520	0.03250
C	1.82320	0.99390	-0.77350
C	2.41850	-0.03950	1.40280
C	2.07740	2.27240	-0.18570
H	1.80710	0.92360	-1.86610

I	2.39790	-2.05710	-0.91050
C	2.62580	1.20910	1.92770
C	2.45970	2.37930	1.13260
H	2.58520	-0.93270	2.00930
H	2.94370	1.31170	2.97000
H	1.99640	3.17130	-0.80280
H	-4.45810	-3.44150	2.12020
H	-5.61870	2.50430	-1.38160
C	2.72110	3.71740	1.72300
O	2.57390	4.75980	1.13170
H	3.07210	3.70470	2.78290

(bipy)Au(4-CHO-C_6H_4I)$^+$ transition state

SCF = –1272.91084 au
ZPE = –1272.64828 au

Au	-0.28710	-0.45200	0.03730
I	1.27200	-2.64600	-0.49730
C	-4.52900	-1.55150	0.86760
C	-2.35160	1.93810	-0.22180
C	-2.92280	3.19730	-0.42280
C	-2.10170	4.26300	-0.78450
C	-0.73470	4.05070	-0.93610
C	-0.24380	2.76720	-0.71650
N	-1.03470	1.75420	-0.37240
H	-3.99370	3.36180	-0.30210
H	-0.05640	4.85810	-1.21970
H	0.82160	2.53910	-0.82280
C	-3.15820	0.73550	0.16000
C	-4.54490	0.78530	0.31500
C	-5.23560	-0.36890	0.67220
C	-3.15100	-1.52880	0.69190
N	-2.49280	-0.41660	0.34990
H	-5.09390	1.71350	0.15700
H	-5.02960	-2.48050	1.14790
H	-2.54940	-2.43150	0.82790
C	1.90420	-0.56390	0.04810
C	2.23870	-0.33260	1.39890
C	2.43680	0.25760	-0.97290
C	3.03280	0.76530	1.72210
C	3.23130	1.33780	-0.62650
C	3.52780	1.60430	0.72030
H	2.21300	0.04840	-2.02160
H	3.27760	0.96200	2.77070
H	3.63390	1.99470	-1.40230
H	1.88530	-1.00460	2.18440
H	-2.53450	5.25360	-0.94620
H	-6.32110	-0.33930	0.79580
C	4.36870	2.77090	1.08130
O	4.81690	3.55670	0.28070
H	4.56780	2.88900	2.17390

(bipy)Au(4-^{t}Bu-C_6H_4I)$^+$ product

SCF = –1272.95017 au
ZPE = –1272.68496 au

Au	-0.29900	-0.20250	0.02410
I	0.19410	-2.74580	-0.02540
C	-1.45510	4.53270	-0.00860
C	-0.11180	4.17860	0.03920
C	0.21870	2.83040	0.05670
N	-0.71730	1.87550	0.03010
C	-2.03080	2.19670	-0.01410
C	-2.42380	3.53230	-0.03540
H	0.68130	4.92840	0.06070
H	1.25980	2.50390	0.09140
H	-3.47910	3.80230	-0.07330
C	-5.20600	0.12770	-0.09290
C	-4.64090	-1.14190	-0.06450
C	-3.25580	-1.25000	-0.02610
N	-2.46370	-0.17520	-0.01410
C	-2.99430	1.06570	-0.03770
C	-4.37390	1.24510	-0.07880
H	-5.25440	-2.04480	-0.07300
H	-2.75930	-2.22350	-0.00620
H	-4.80770	2.24440	-0.09940
C	4.40500	0.67120	0.16000
C	3.69550	0.46980	1.34790
C	2.34100	0.13910	1.31200
C	1.70260	0.01880	0.07590
C	2.40550	0.19740	-1.12060
C	3.75610	0.52950	-1.07330
H	4.20590	0.57190	2.31130
H	1.79510	-0.01920	2.24580
H	1.90710	0.08360	-2.08690
H	4.32280	0.68080	-1.99630
H	-1.75550	5.58300	-0.02640
H	-6.29050	0.25680	-0.12610
C	5.84320	1.03090	0.21380
O	6.53140	1.23760	-0.75690
H	6.26950	1.10640	1.24380

6. Selected spectra

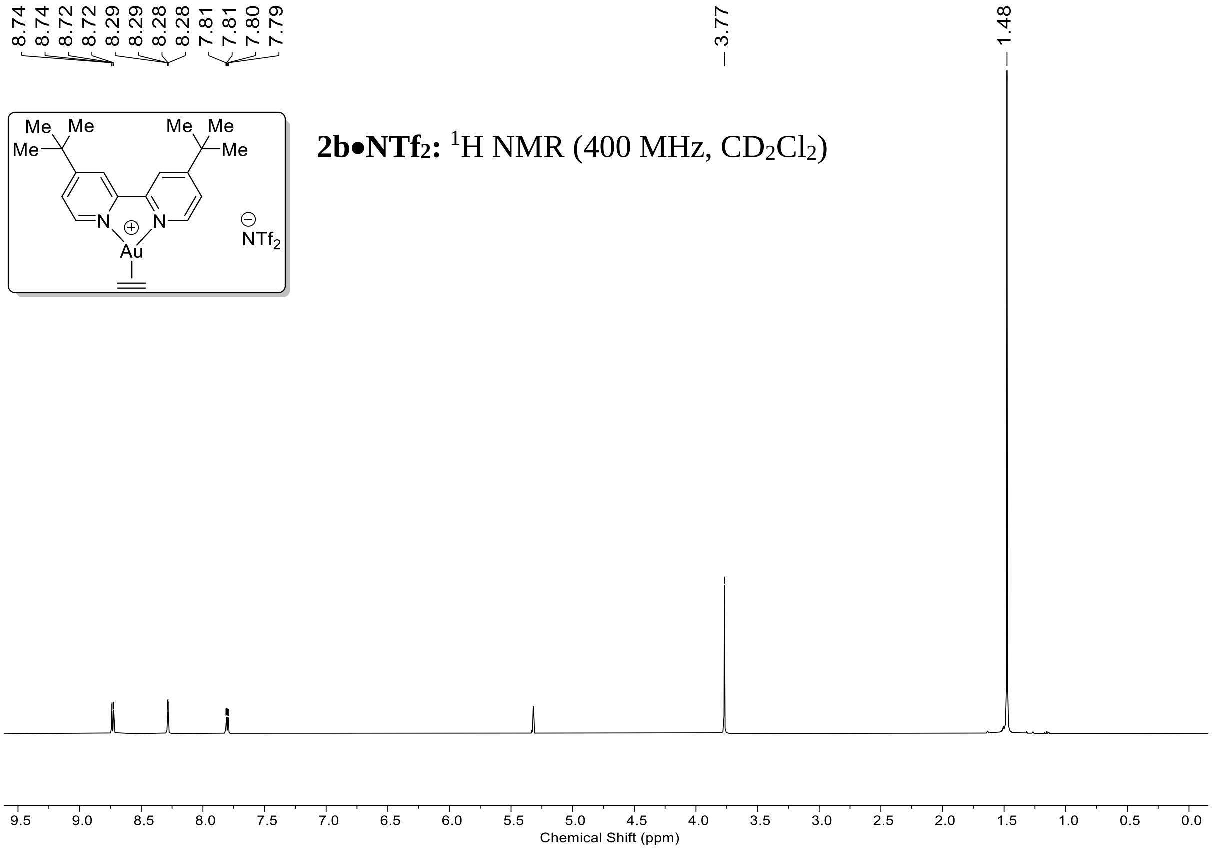

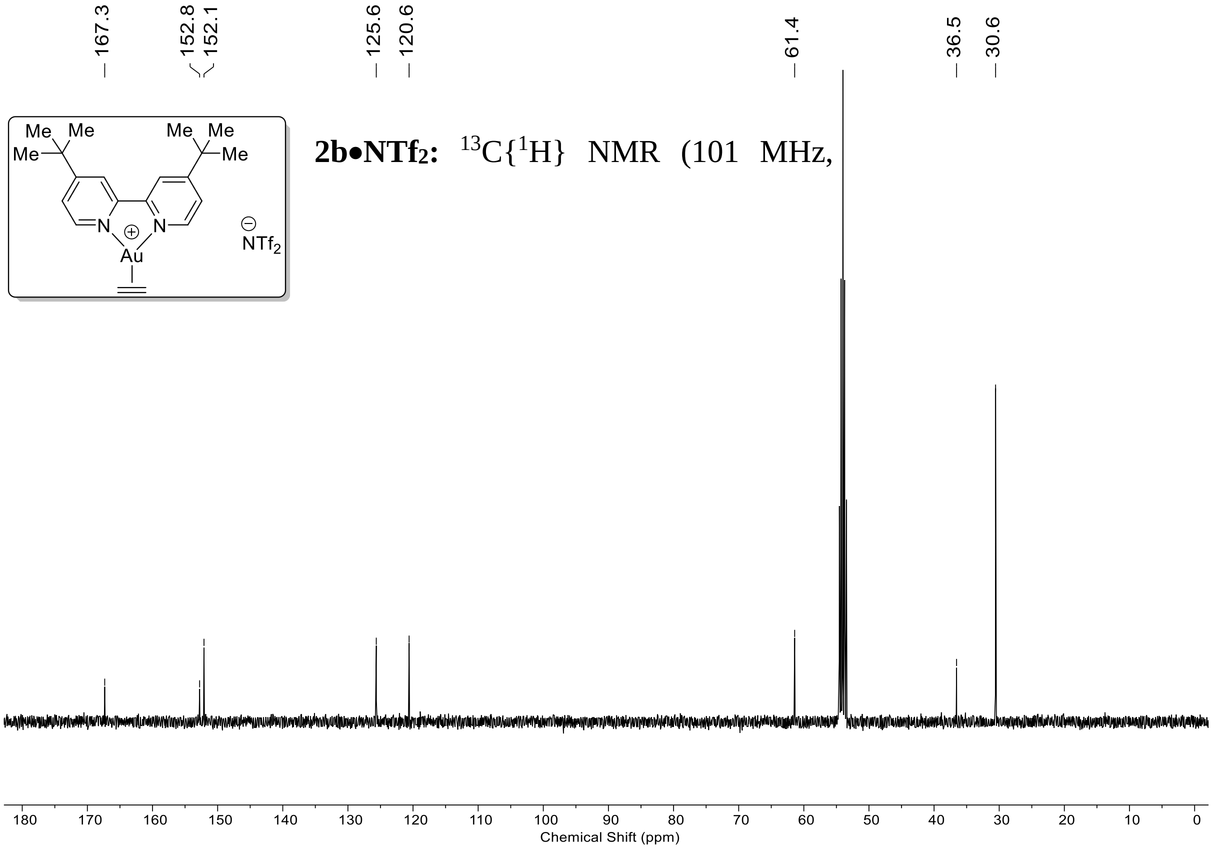

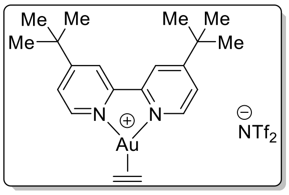

2b•NTf₂: Raman (neat)

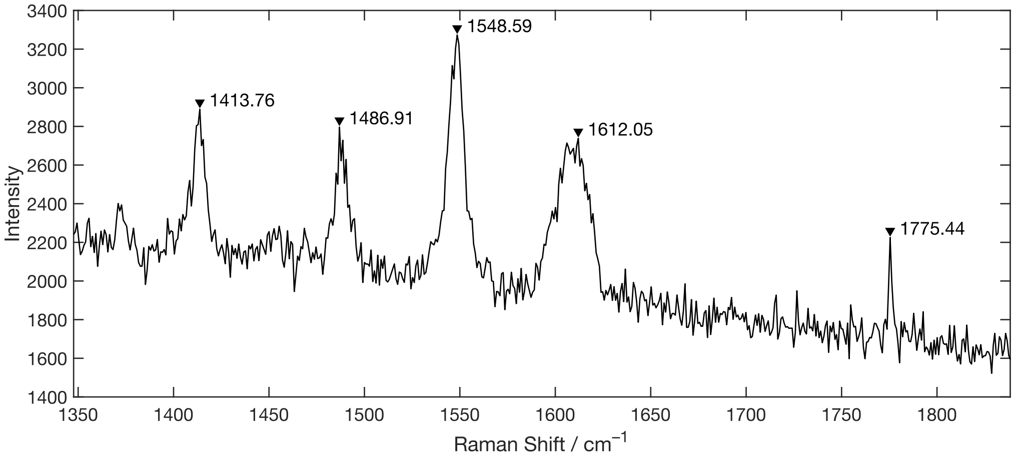

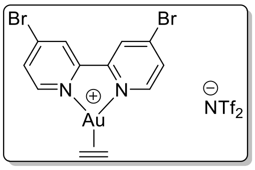

2c•NTf₂: ^{1}H NMR (500 MHz, CD_2Cl_2)

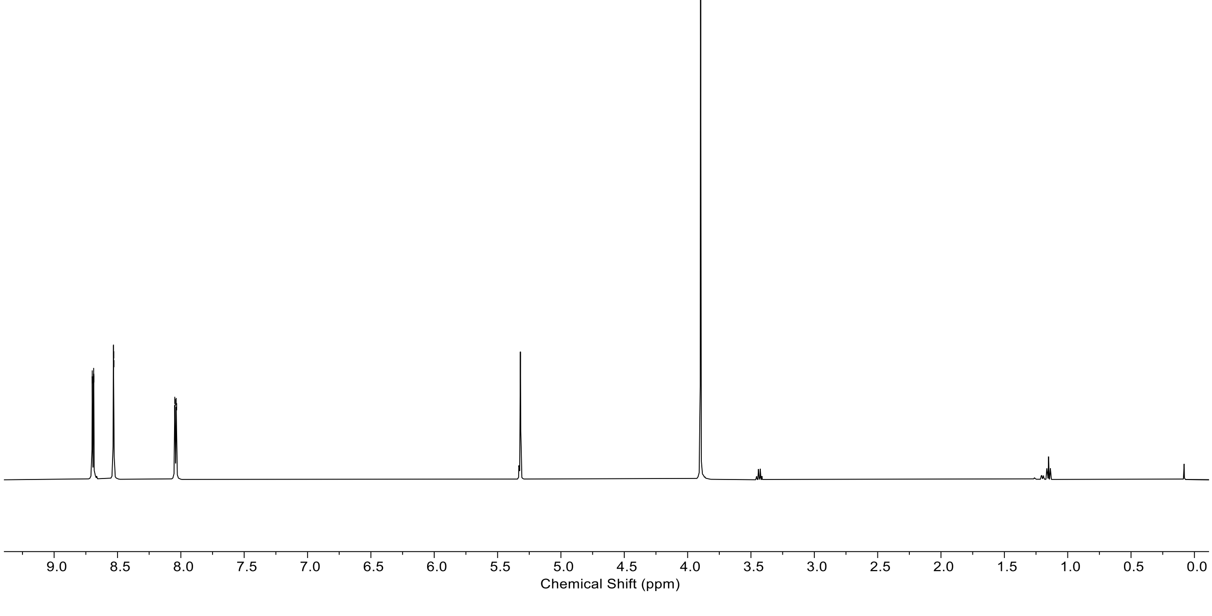

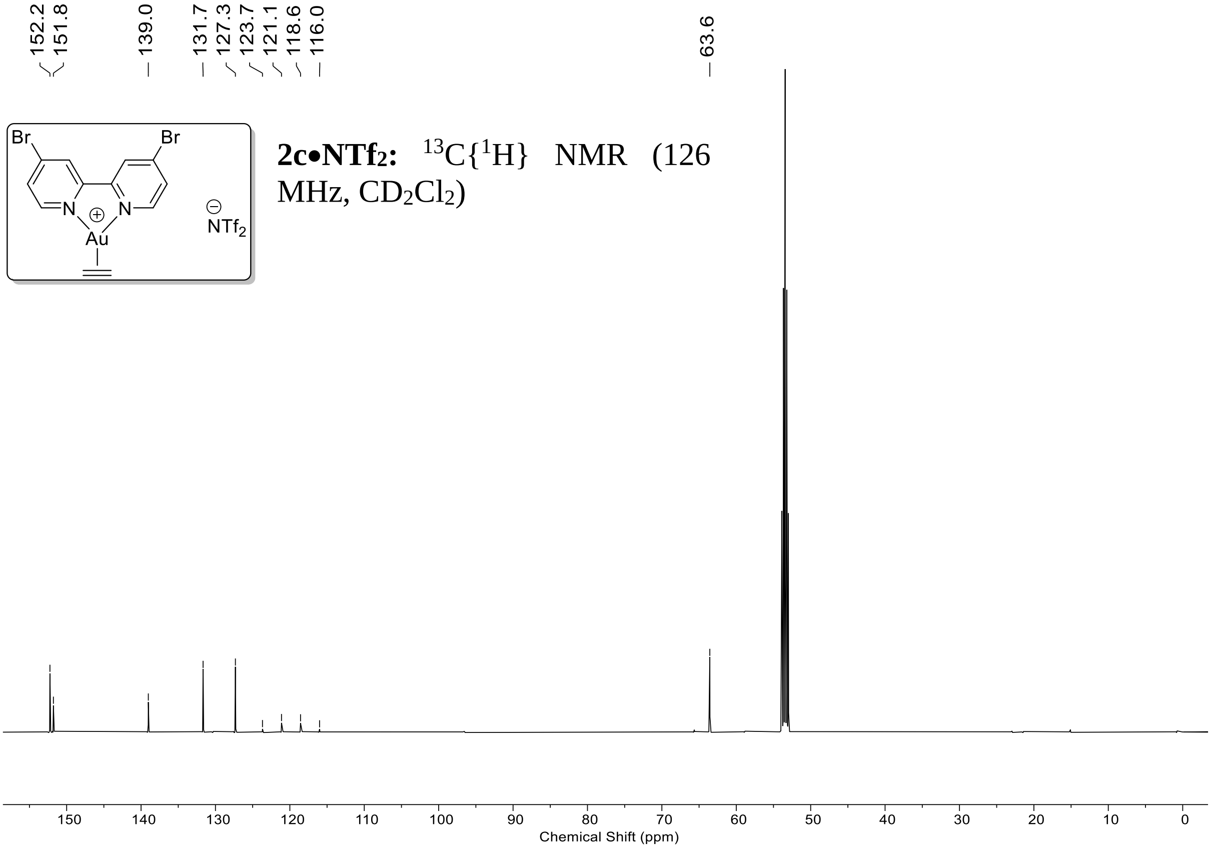

2c•NTf$_2$: ^{13}C{^{1}H} NMR (126 MHz, CD_2Cl_2)

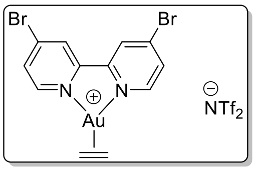

2c•NTf$_2$: Raman (neat)

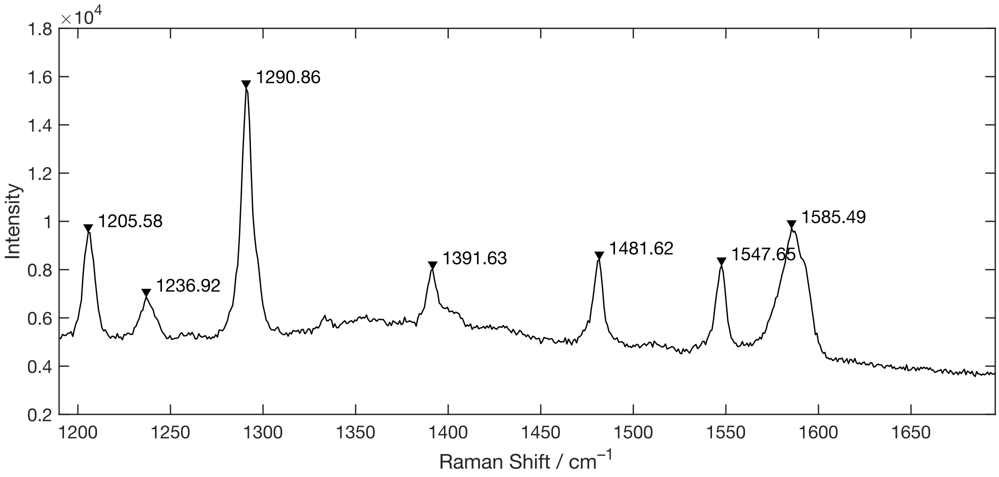

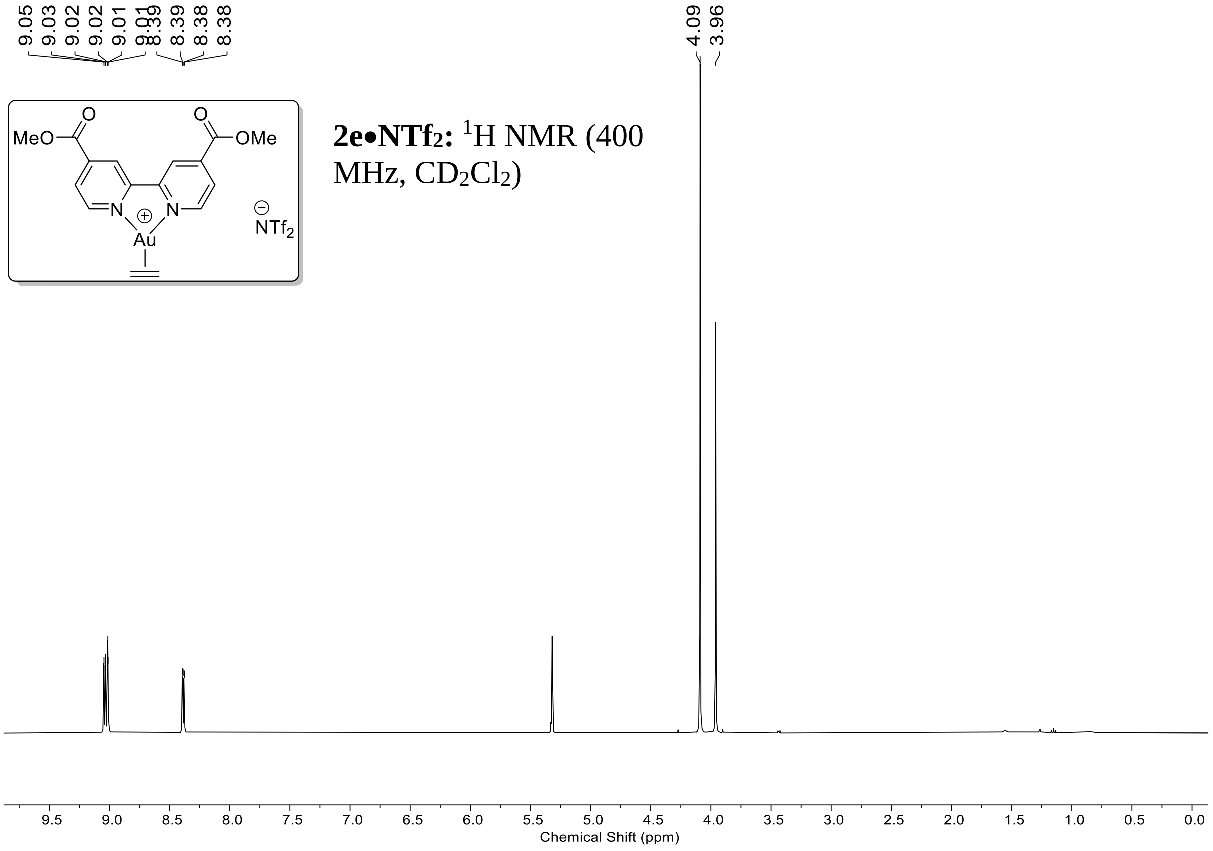

2e•NTf$_2$: ^{1}H NMR (400 MHz, CD_2Cl_2)

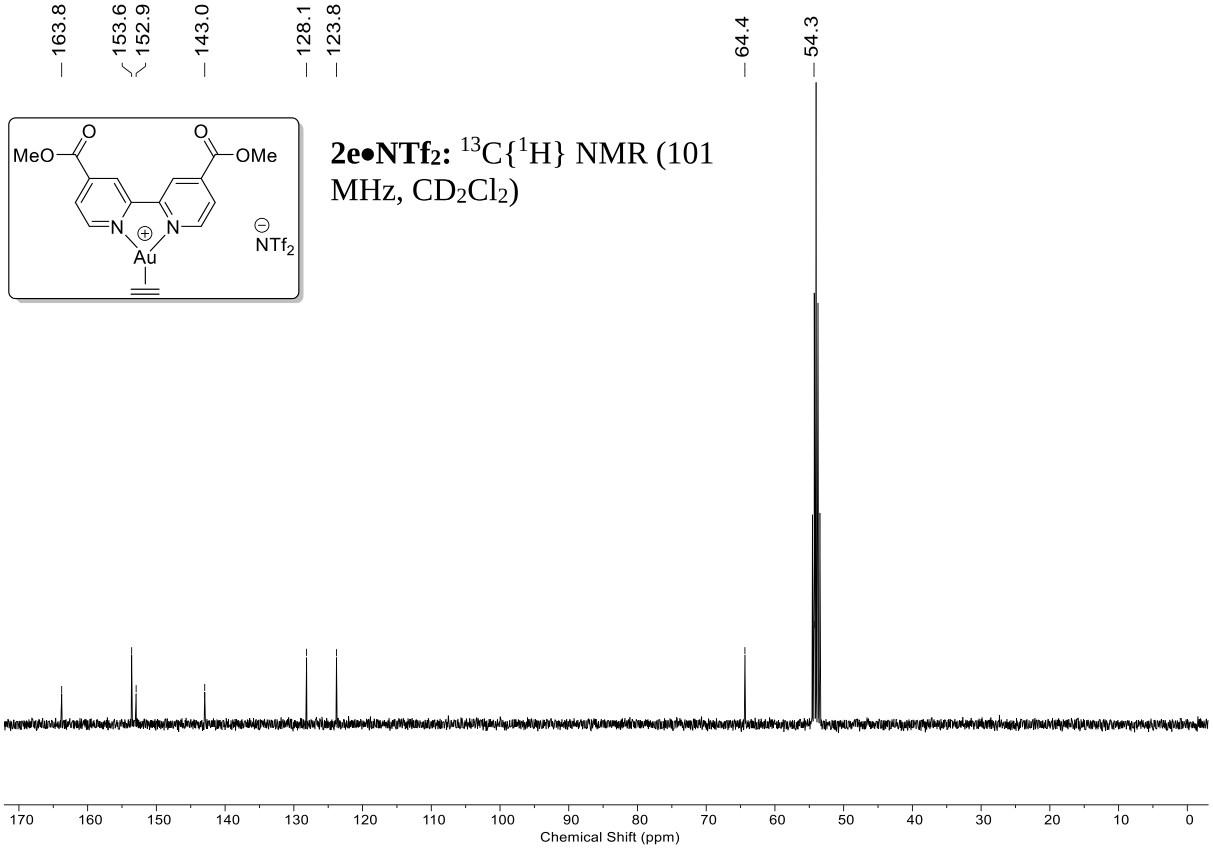

2e•NTf$_2$: ^{13}C{^{1}H} NMR (101 MHz, CD_2Cl_2)

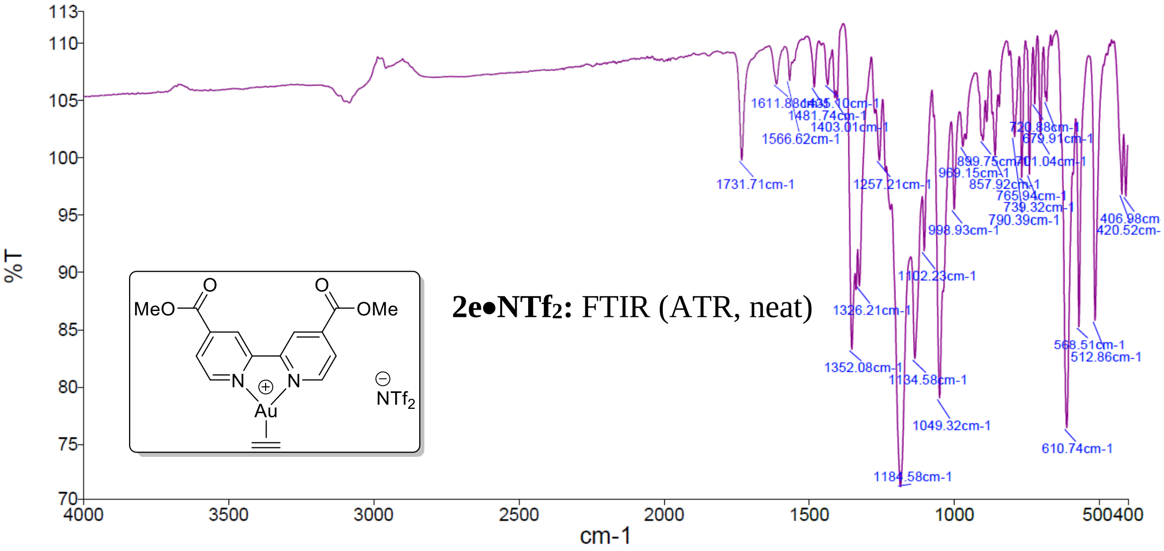

2e•NTf2: FTIR (ATR, neat)

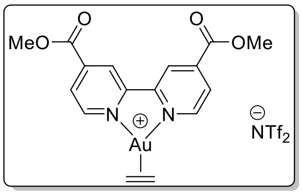

2e•NTf2: Raman (neat)

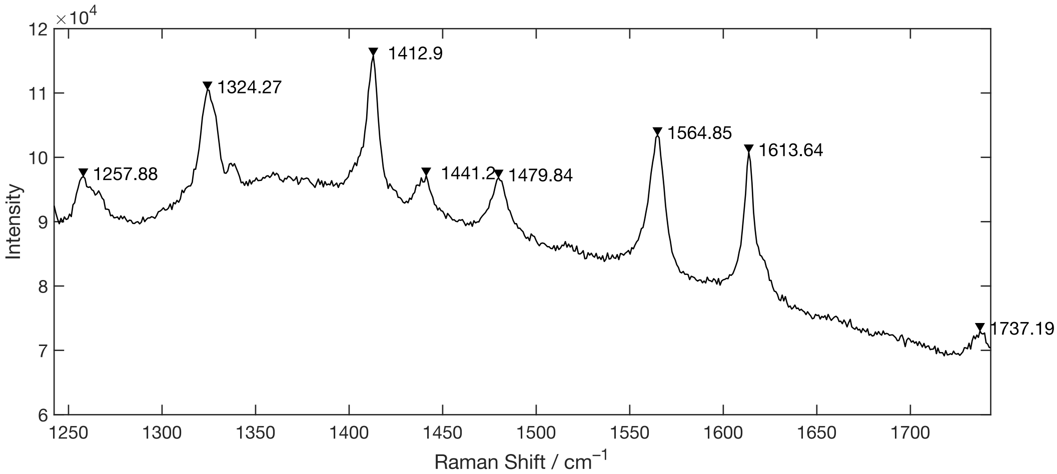

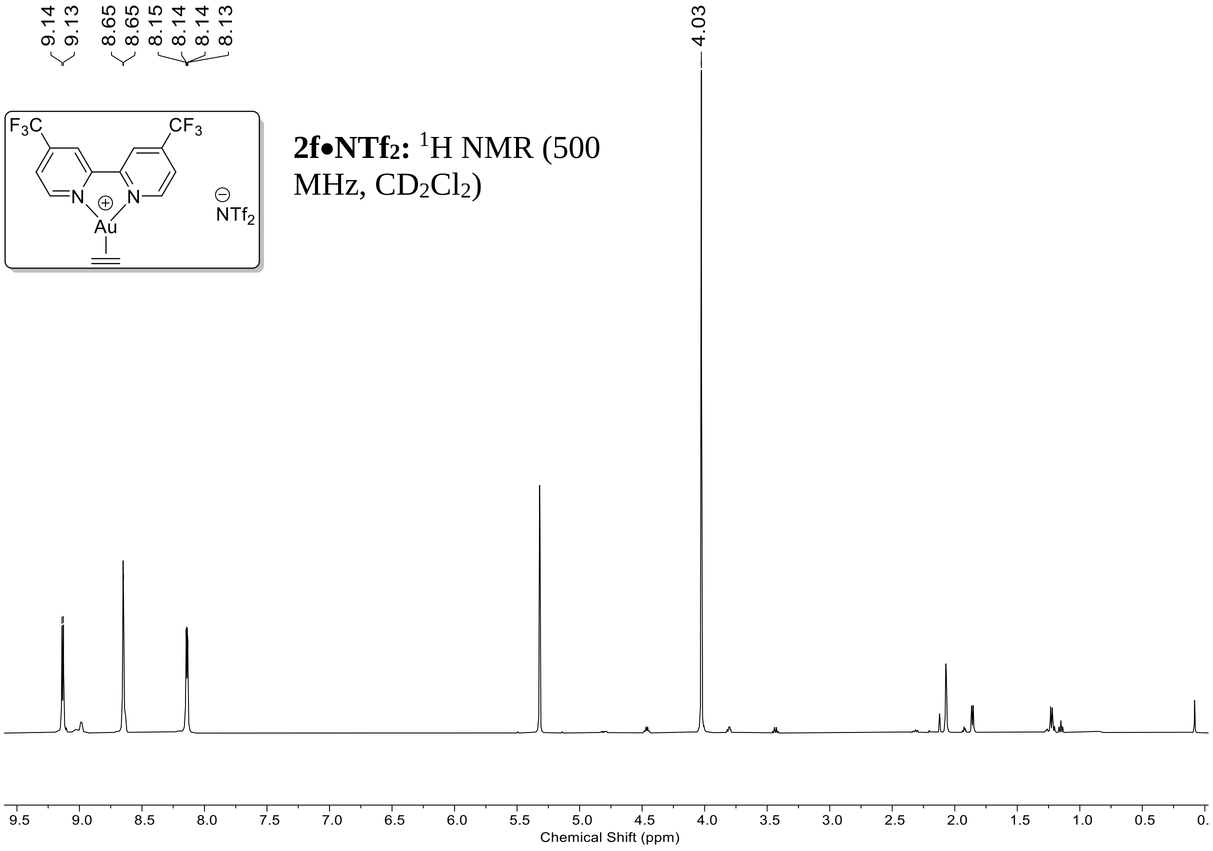
9.14
9.13
8.65
8.65
8.15
8.14
8.14
8.13
4.03
F3C
CF3
N
N
Au
NTf2
2f•NTf2: 1H NMR (500 MHz, CD2Cl2)
9.5 9.0 8.5 8.0 7.5 7.0 6.5 6.0 5.5 5.0 4.5 4.0 3.5 3.0 2.5 2.0 1.5 1.0 0.5
Chemical Shift (ppm)

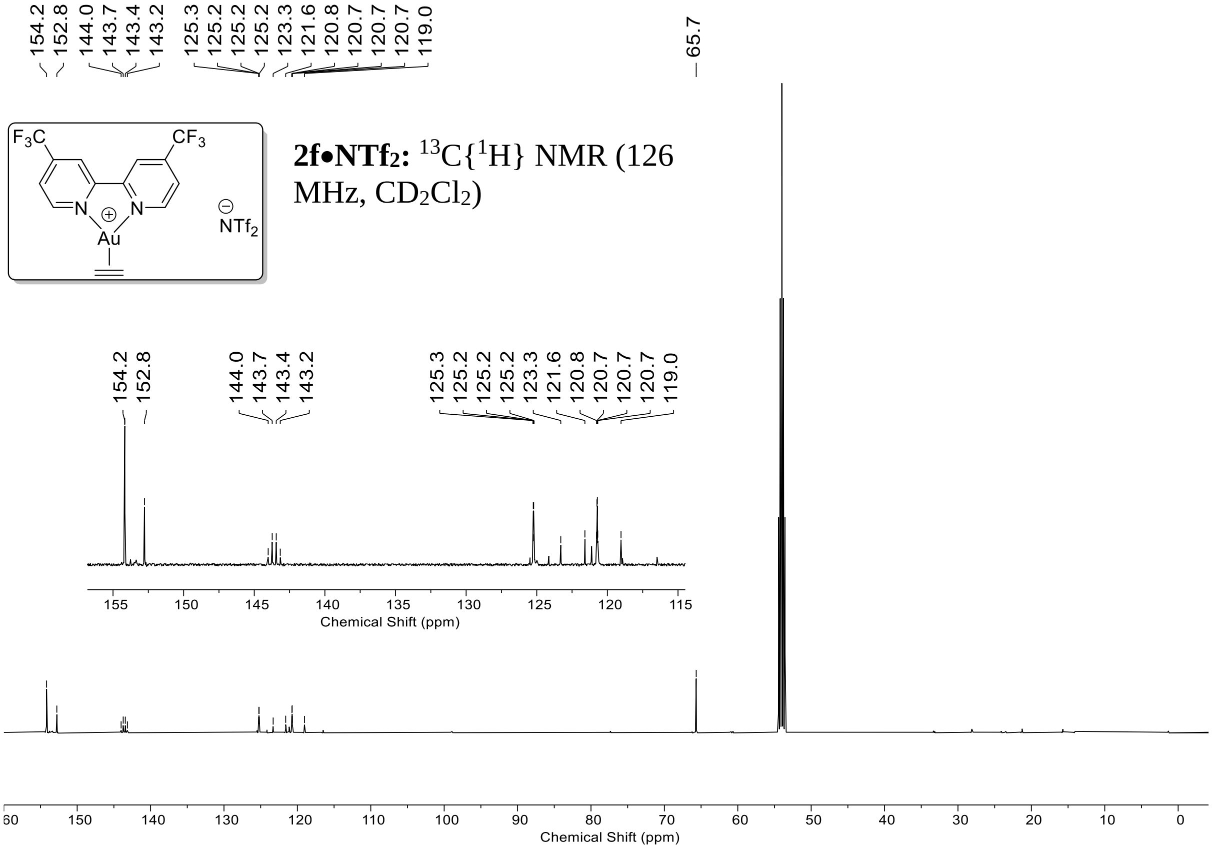
154.2
152.8
144.0
143.7
143.4
143.2
125.3
125.2
125.2
125.2
123.3
121.6
120.8
120.7
120.7
120.7
119.0
65.7
F3C
CF3
N
N
Au
NTf2
2f•NTf2: 13C{1H} NMR (126 MHz, CD2Cl2)
155 150 145 140 135 130 125 120 115
Chemical Shift (ppm)
150 140 130 120 110 100 90 80 70 60 50 40 30 20 10 0
Chemical Shift (ppm)

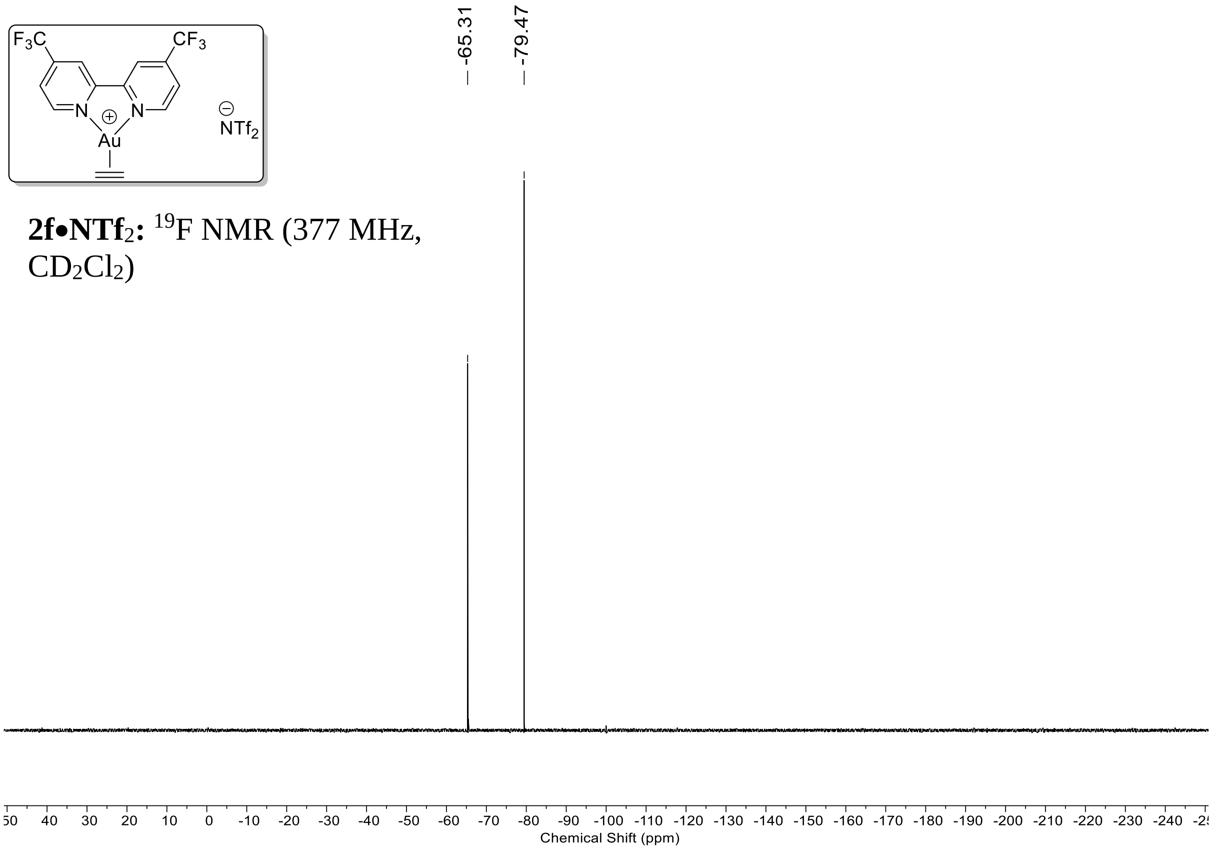

2f•NTf₂: ^{19}F NMR (377 MHz, CD_2Cl_2)

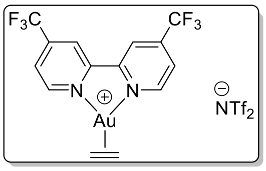

2f•NTf₂: Raman (neat)

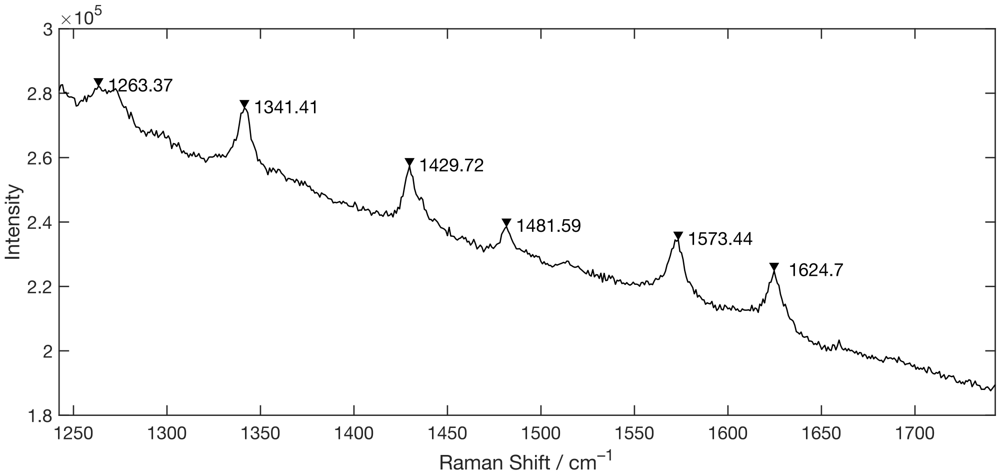

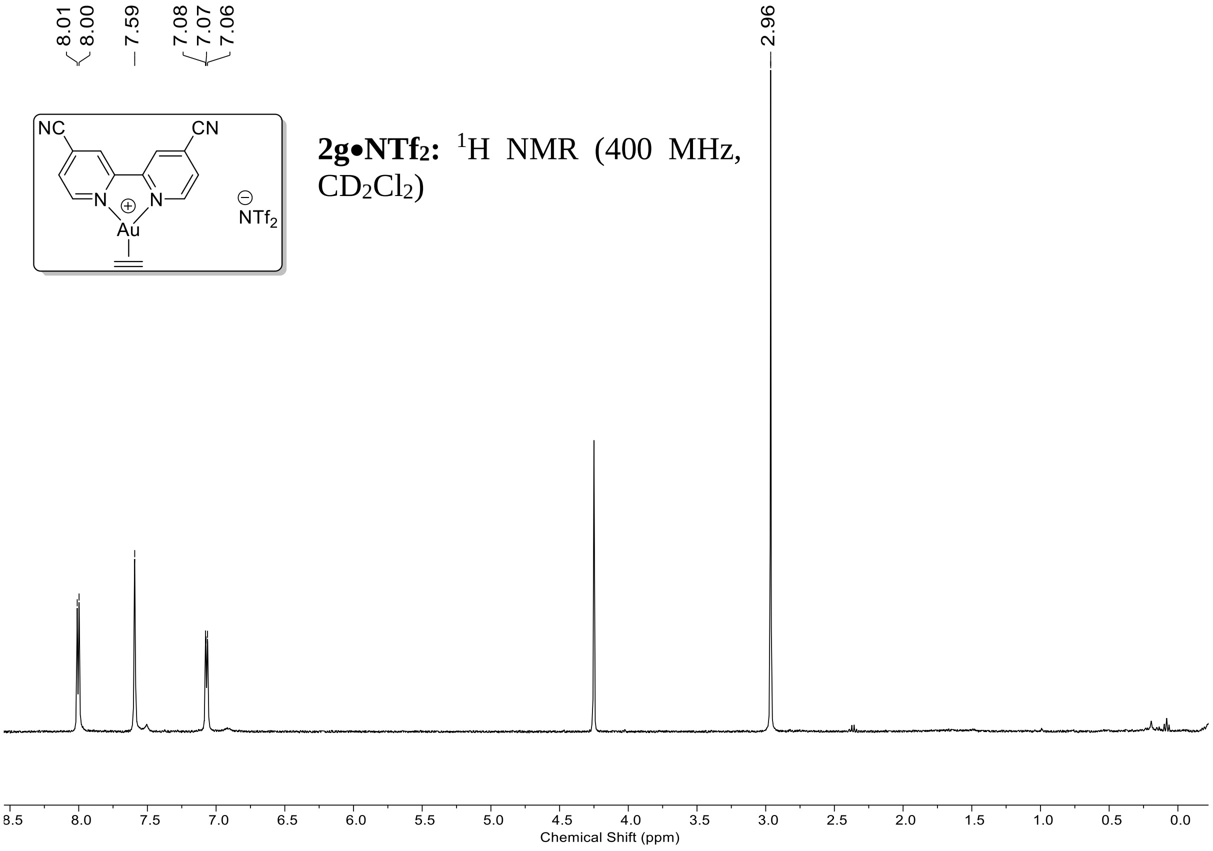

2g•NTf2: ^{1}H NMR (400 MHz, CD_2Cl_2)

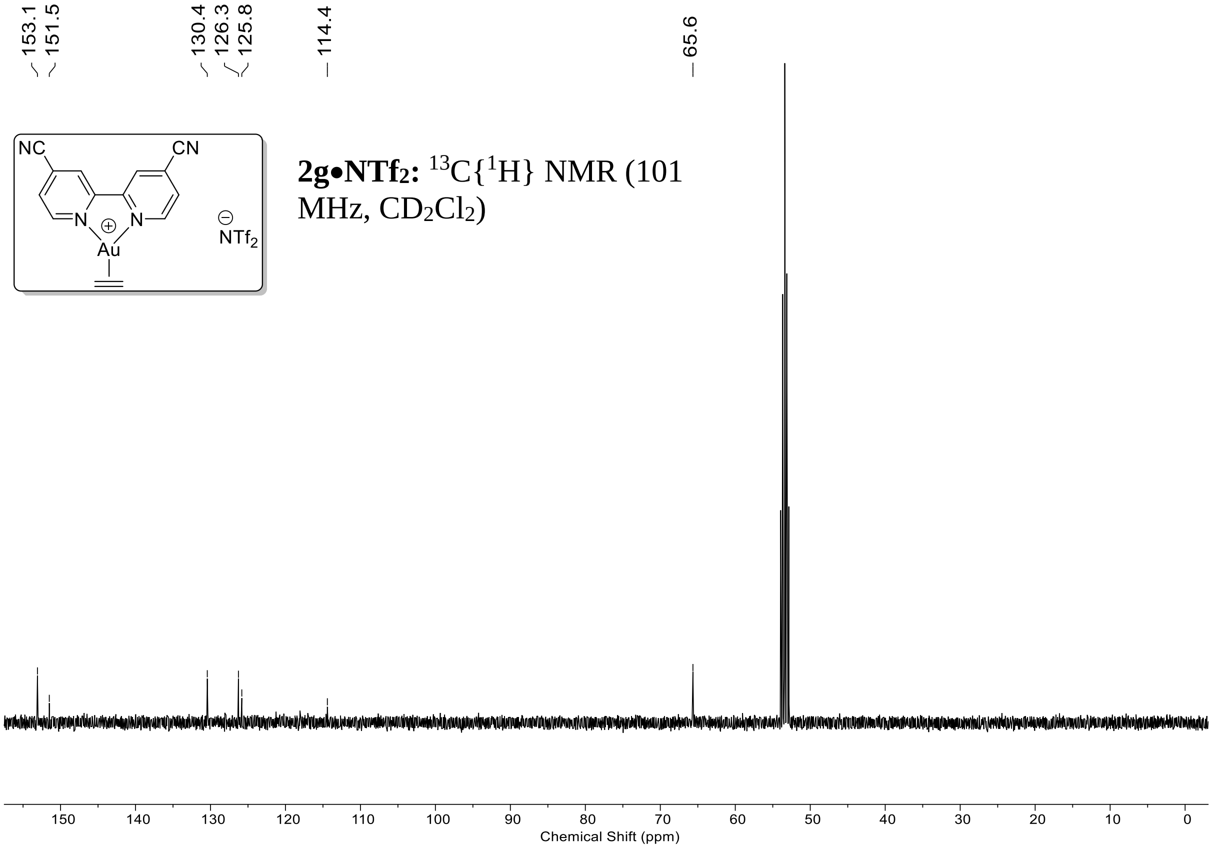

2g•NTf2: $^{13}C\{^{1}H\}$ NMR (101 MHz, CD_2Cl_2)

2h•NTf$_2$: ^{1}H NMR (400 MHz, CD_2Cl_2)

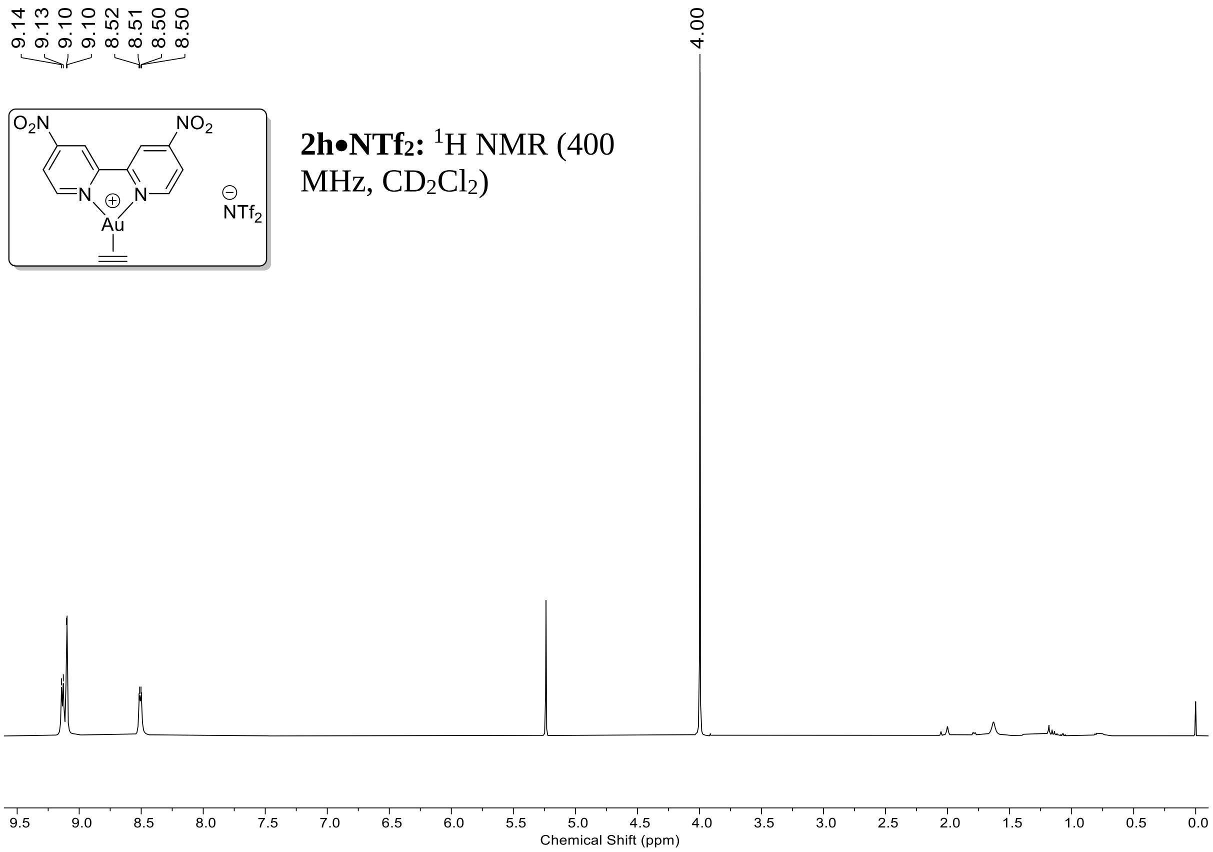

2h•NTf$_2$: ^{13}C{^{1}H} NMR (101 MHz, CD_2Cl_2)

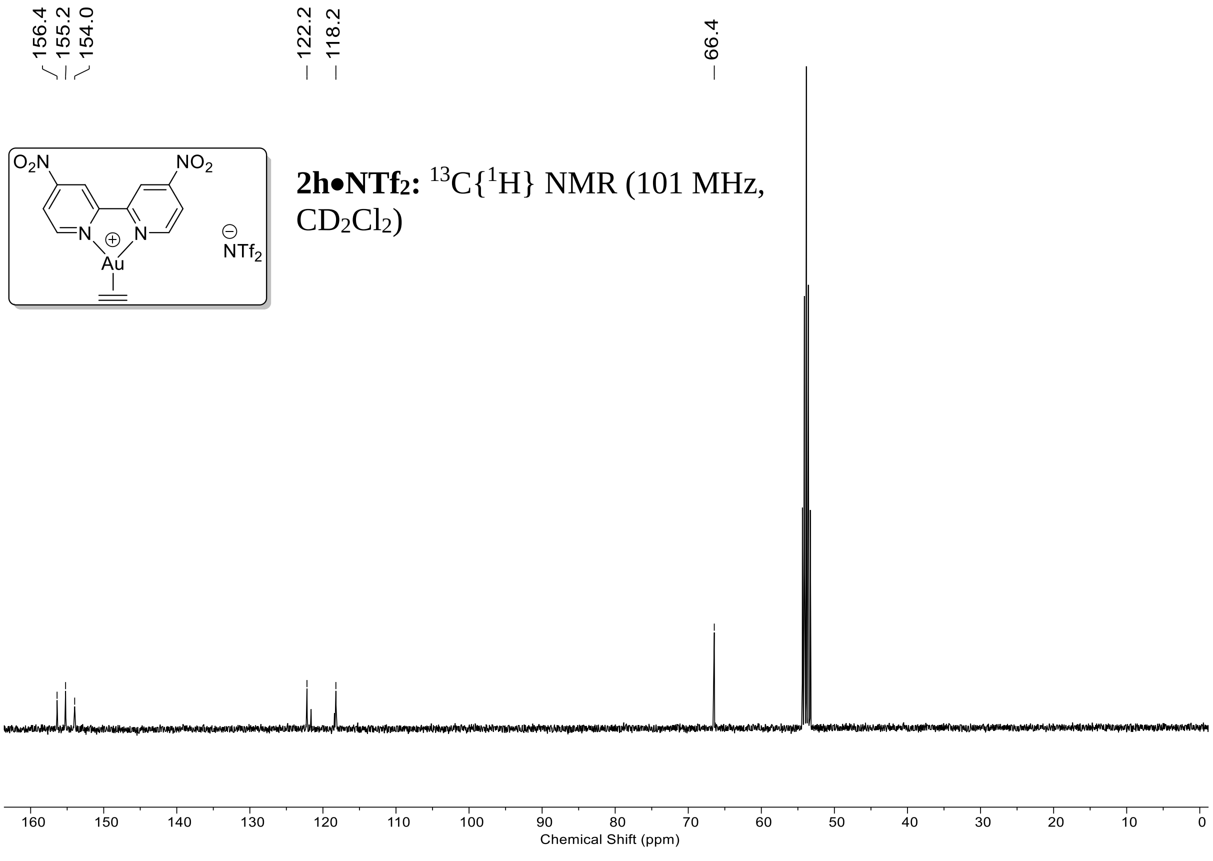

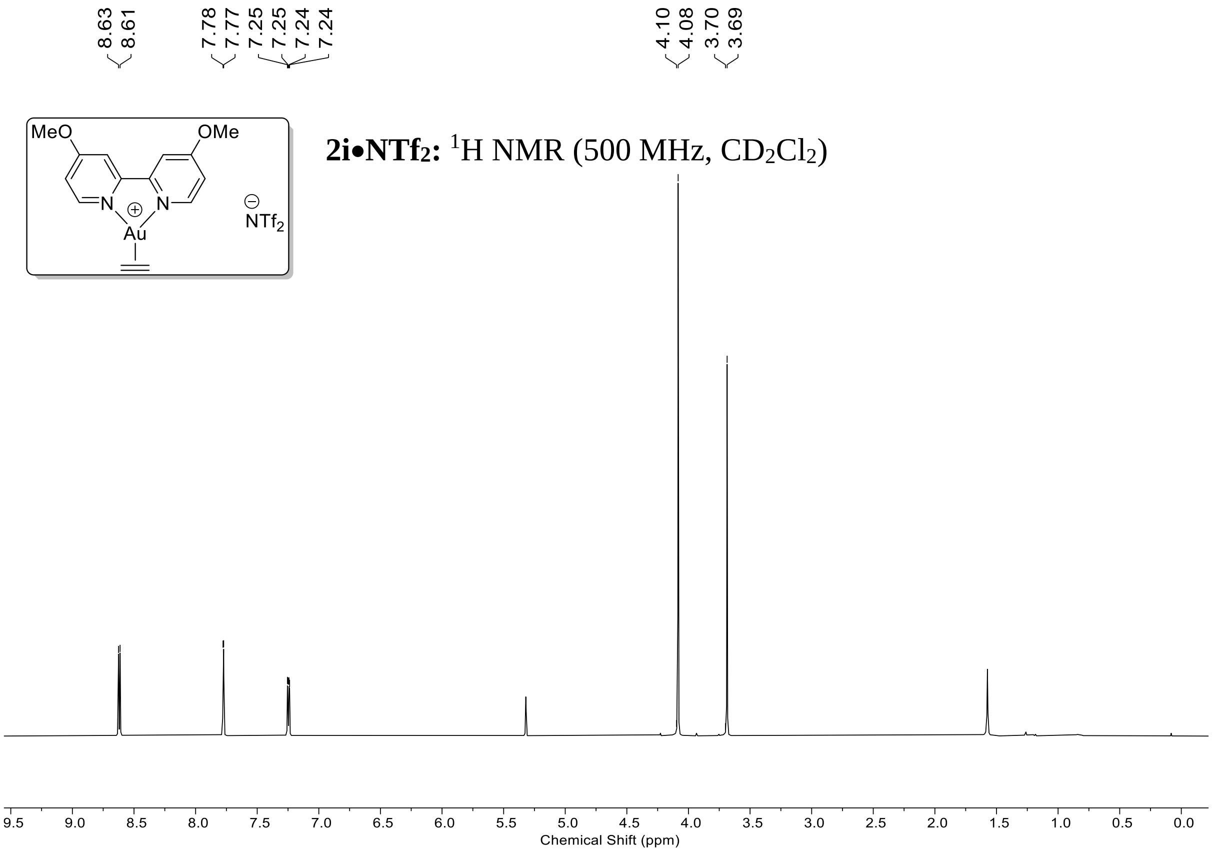

2i•NTf$_2$: ^{1}H NMR (500 MHz, CD_2Cl_2)

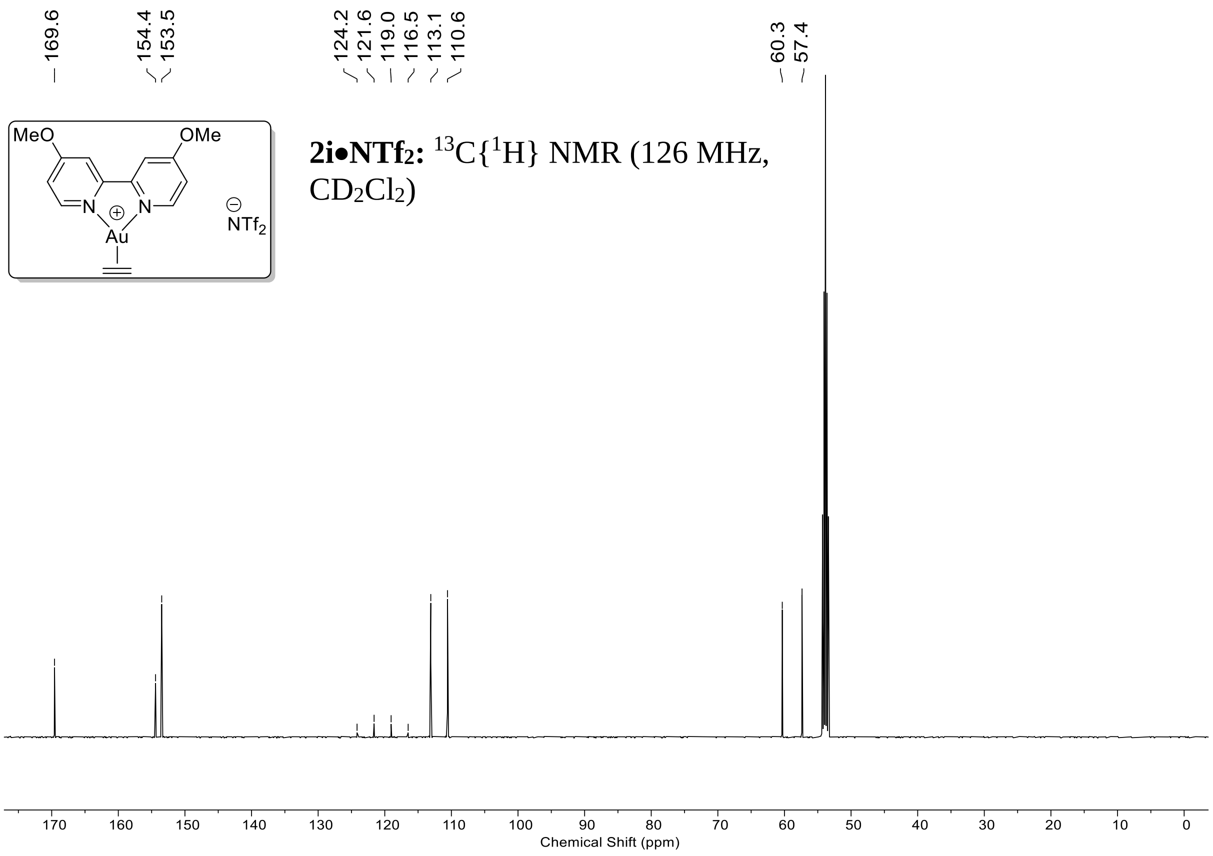

2i•NTf$_2$: ^{13}C{^{1}H} NMR (126 MHz, CD_2Cl_2)

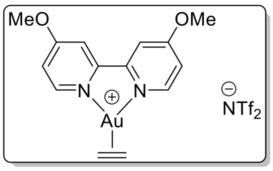

2i•NTf₂: Raman (neat)

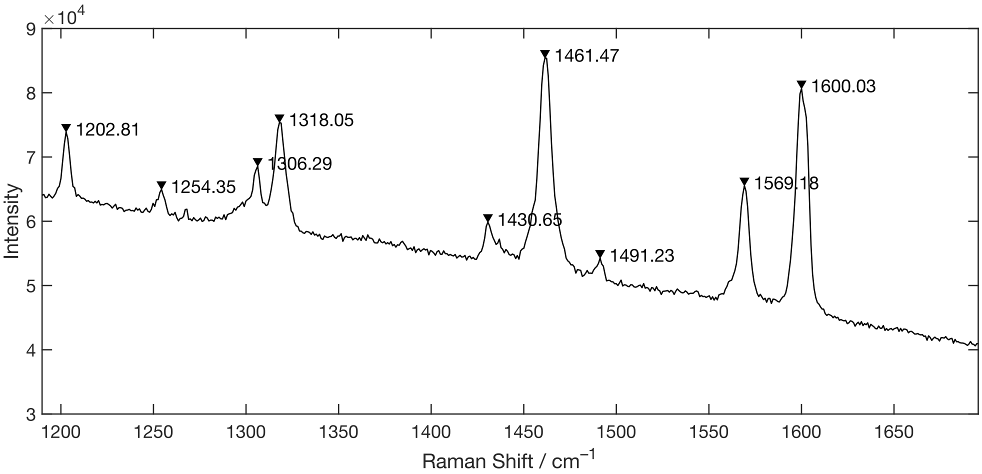

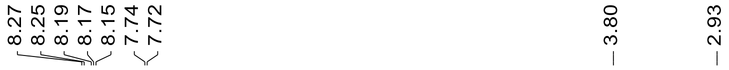

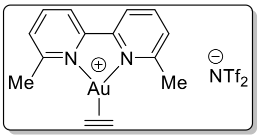

2j•NTf₂: ^{1}H NMR (400 MHz, CD_2Cl_2)

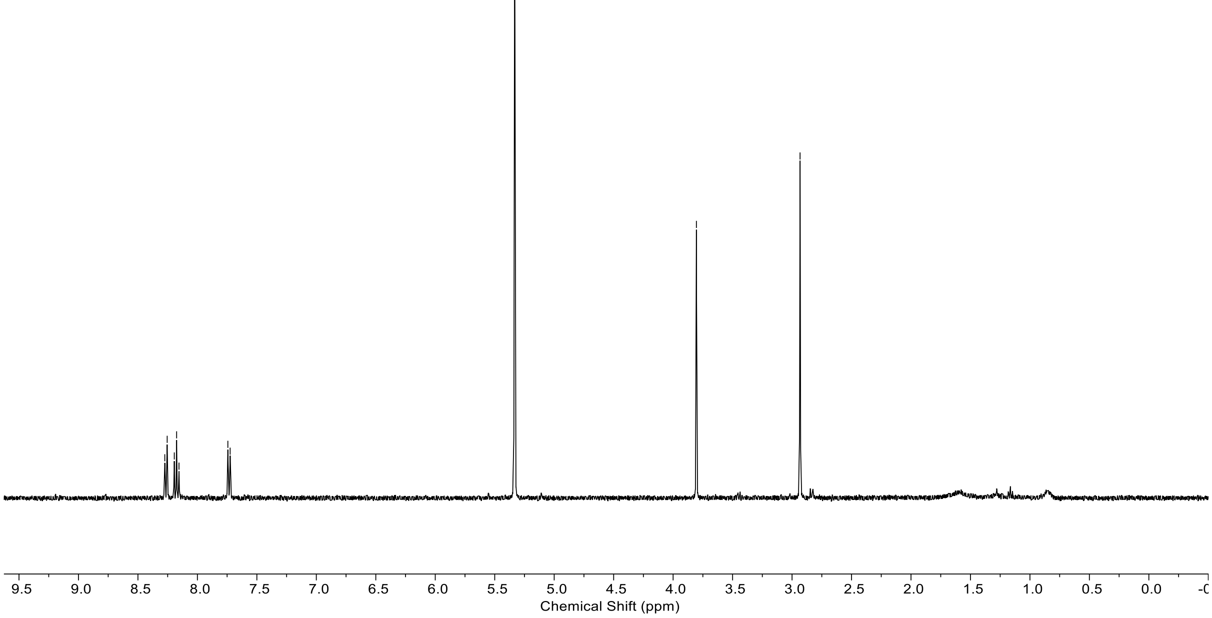

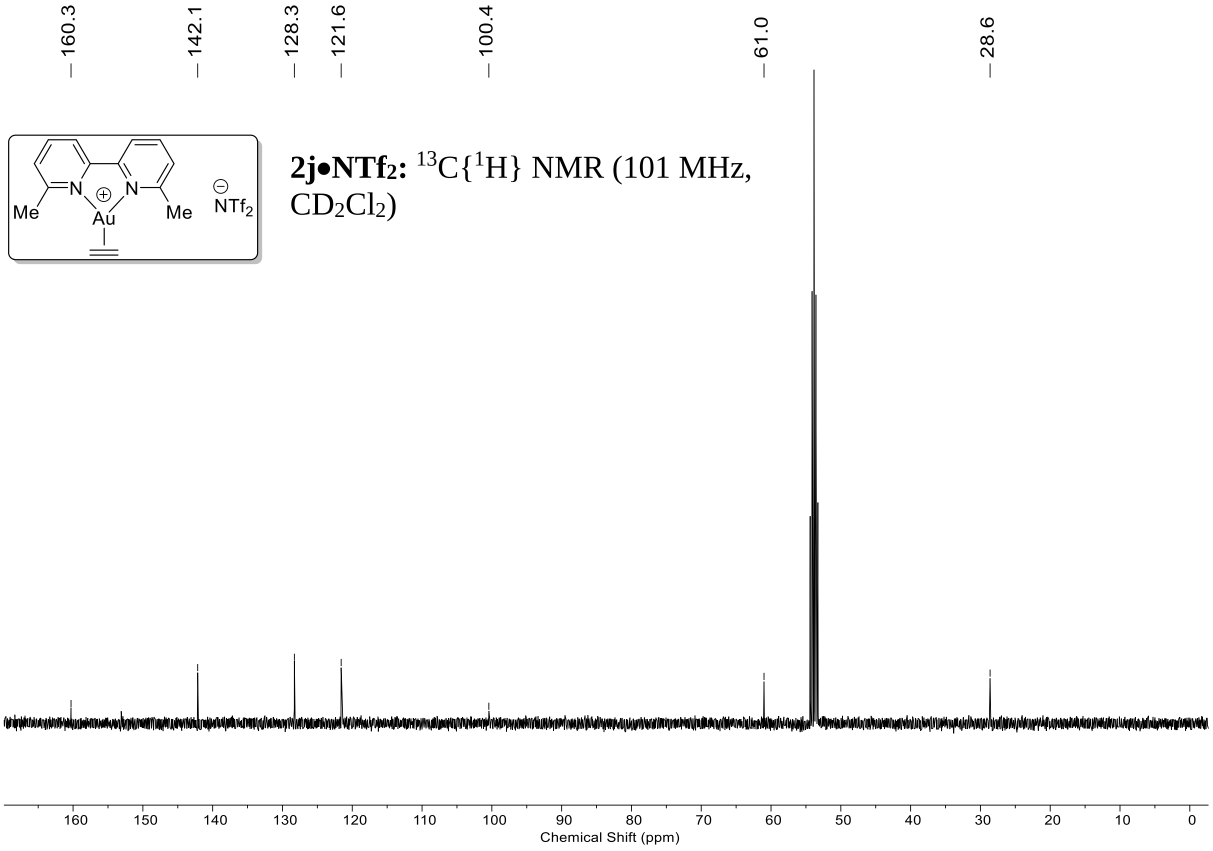
160.3
142.1
128.3
121.6
100.4
61.0
28.6
2j•NTf2: 13C{1H} NMR (101 MHz, CD2Cl2)
Me
N
Au
Me
NTf2
Chemical Shift (ppm)

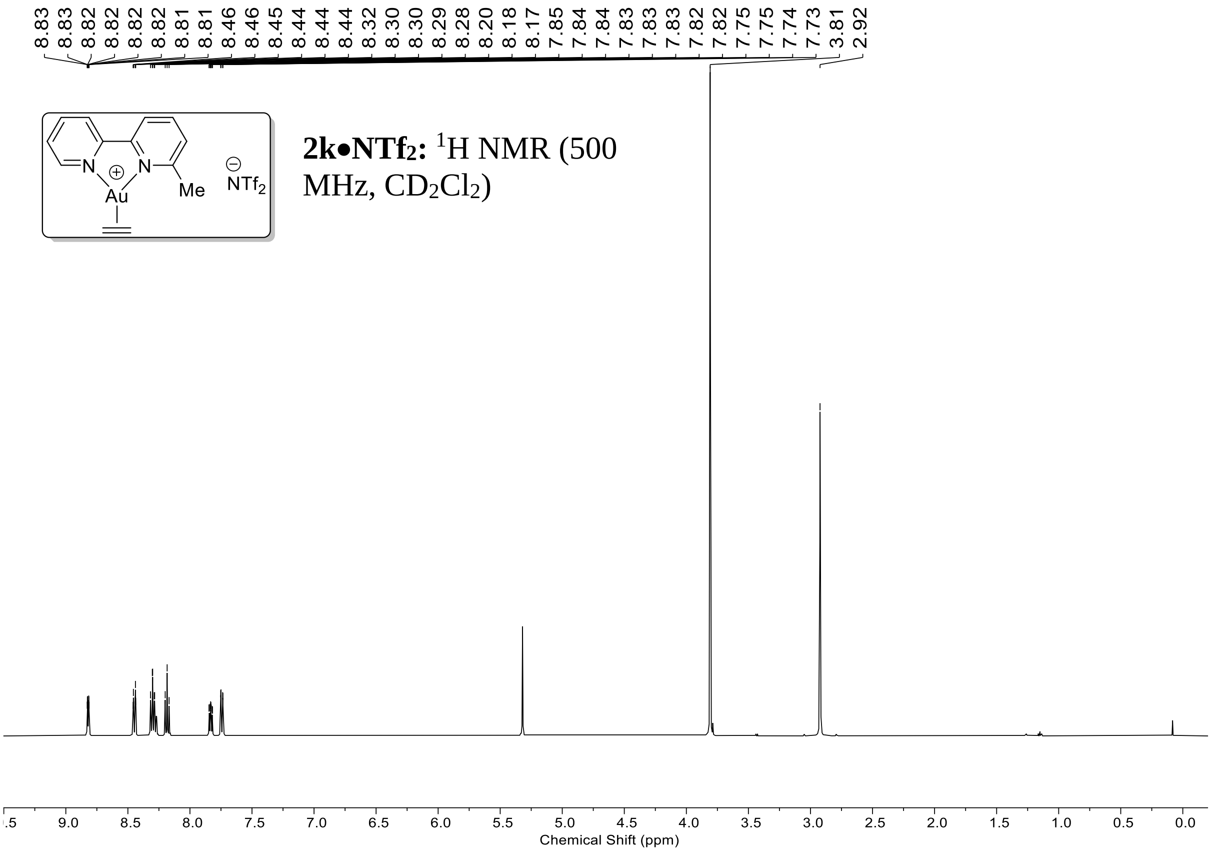
8.83 8.83 8.82 8.82 8.82 8.82 8.81 8.81 8.46 8.46 8.45 8.44 8.44 8.44 8.32 8.30 8.30 8.29 8.28 8.20 8.18 8.17 7.85 7.84 7.83 7.83 7.83 7.82 7.82 7.75 7.75 7.74 7.73 3.81 2.92
2k•NTf2: 1H NMR (500 MHz, CD2Cl2)
N
Au
Me
NTf2
Chemical Shift (ppm)

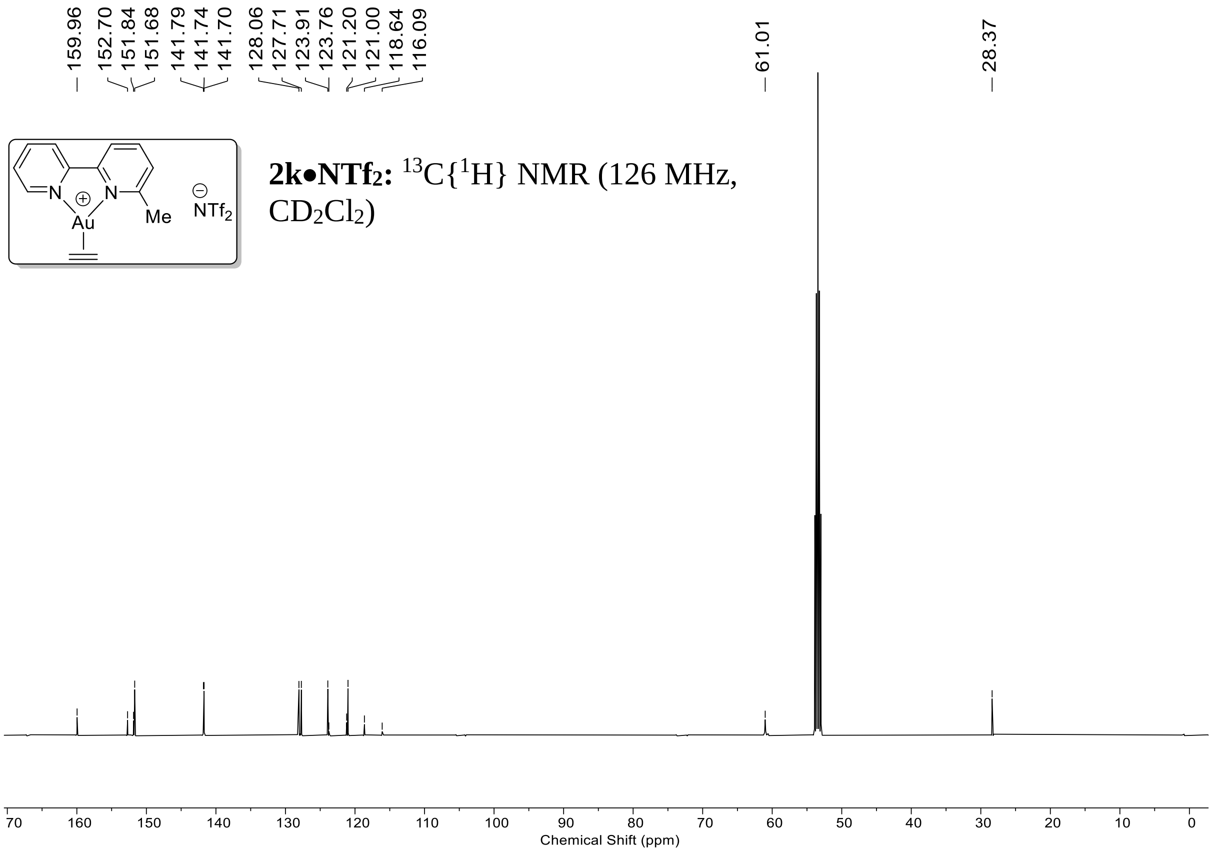

2k•NTf$_2$: $^{13}C\{^1H\}$ NMR (126 MHz, CD_2Cl_2)

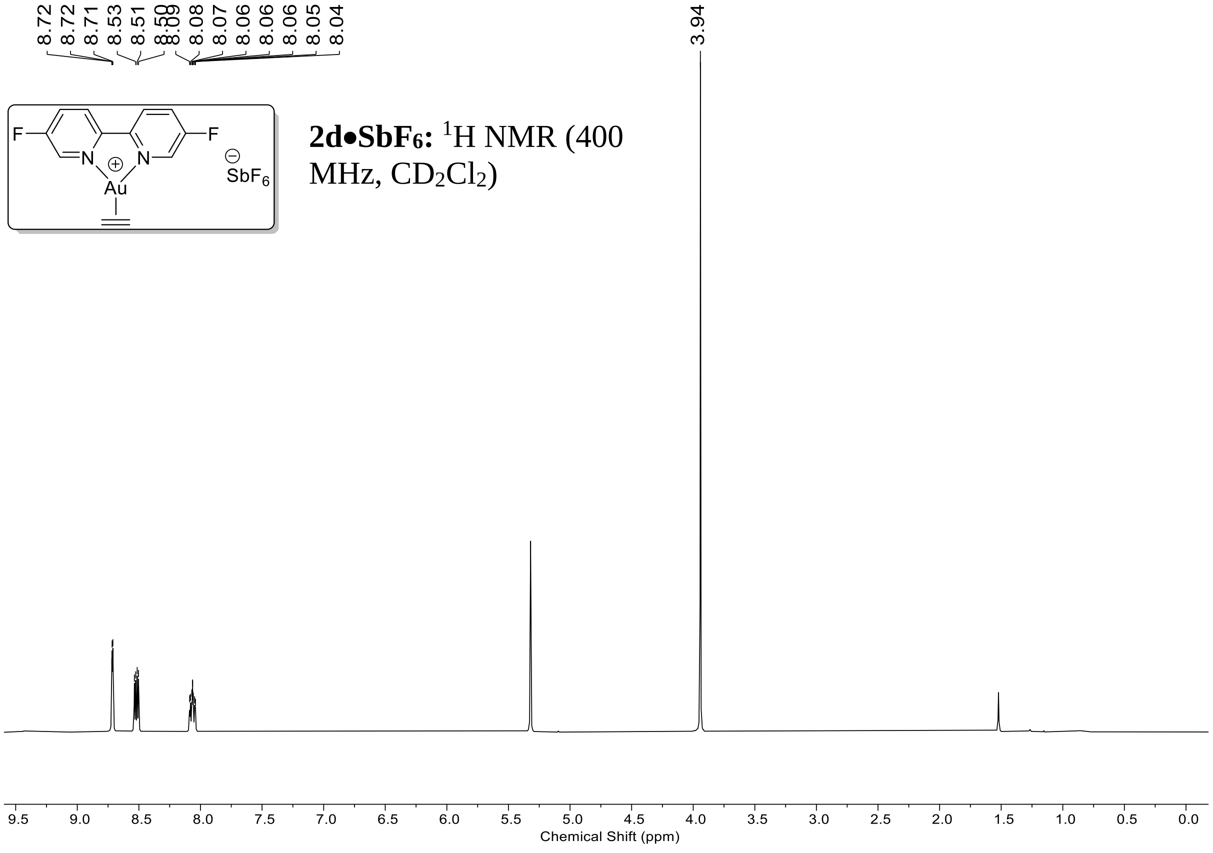

2d•SbF$_6$: ^1H NMR (400 MHz, CD_2Cl_2)

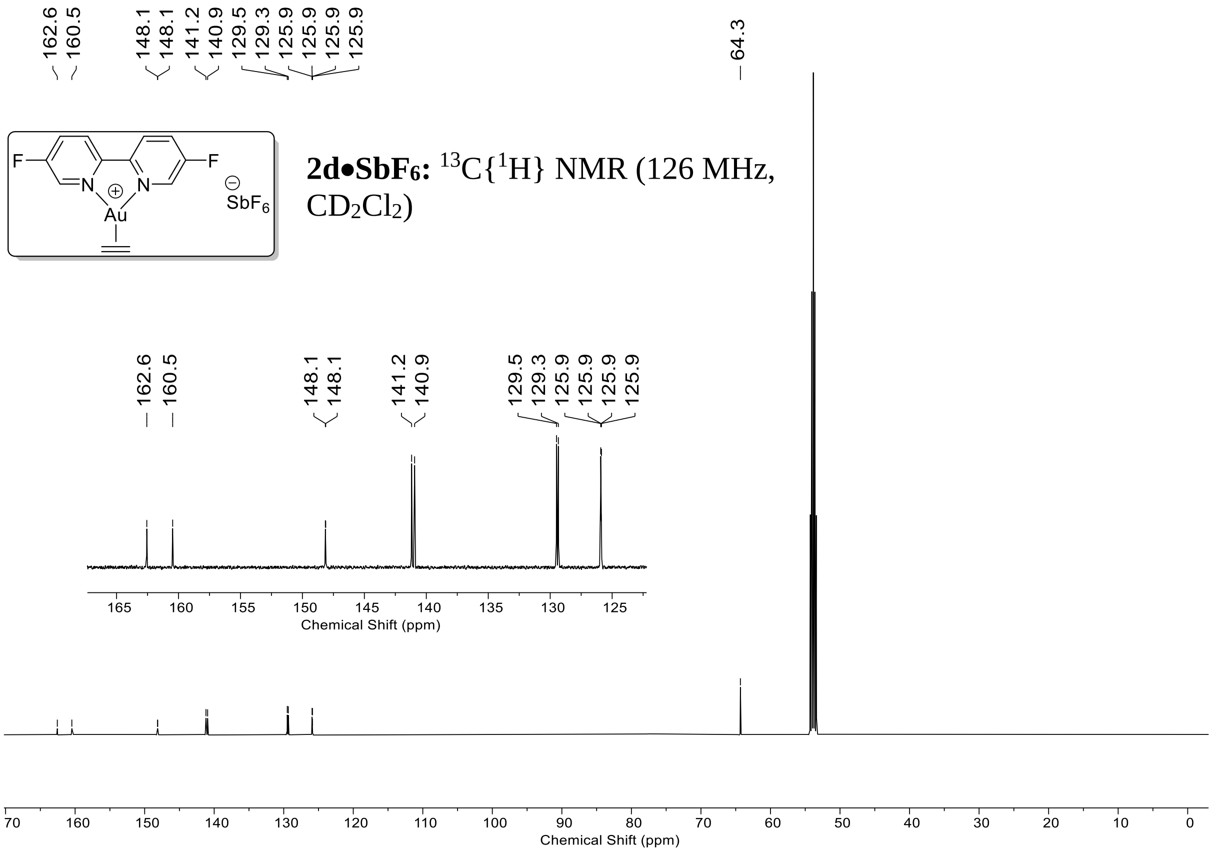

2d•SbF6: $^{13}C\{^{1}H\}$ NMR (126 MHz, CD_2Cl_2)

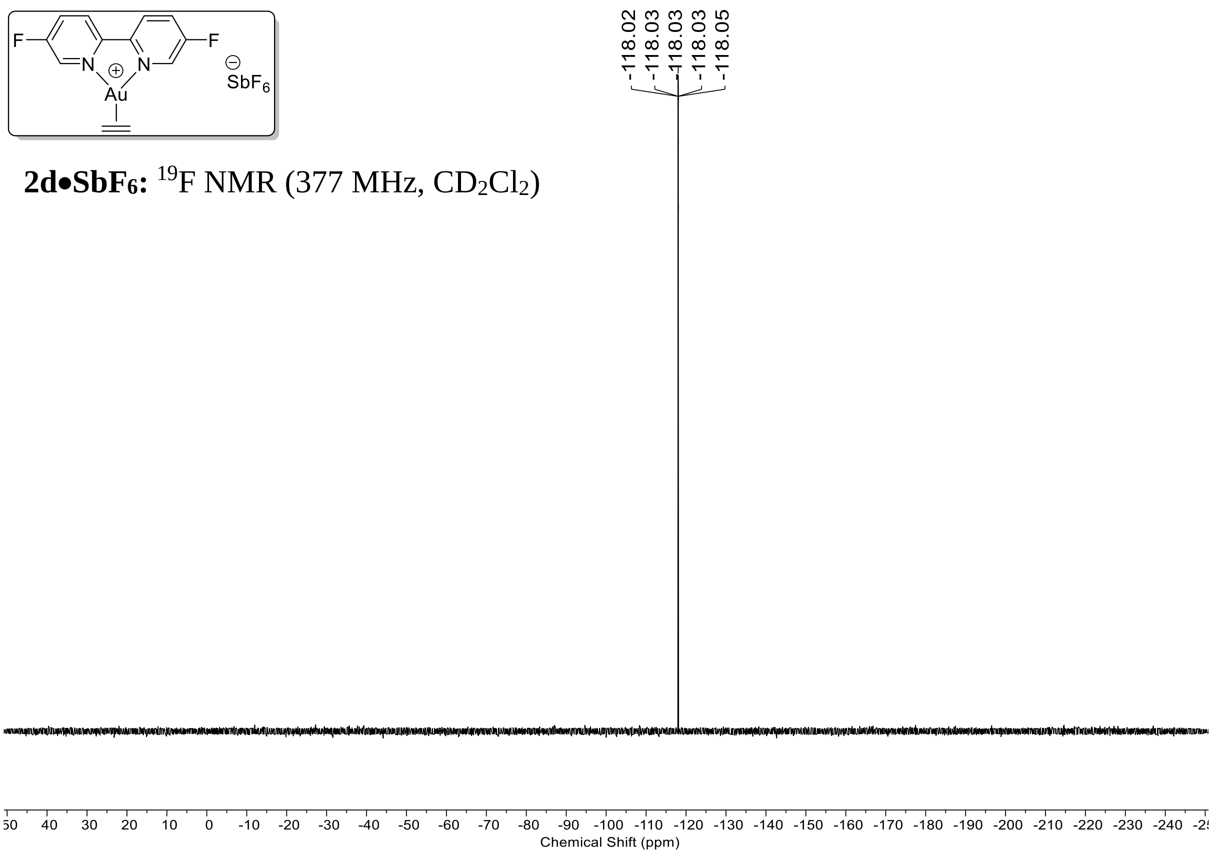

2d•SbF6: ^{19}F NMR (377 MHz, CD_2Cl_2)

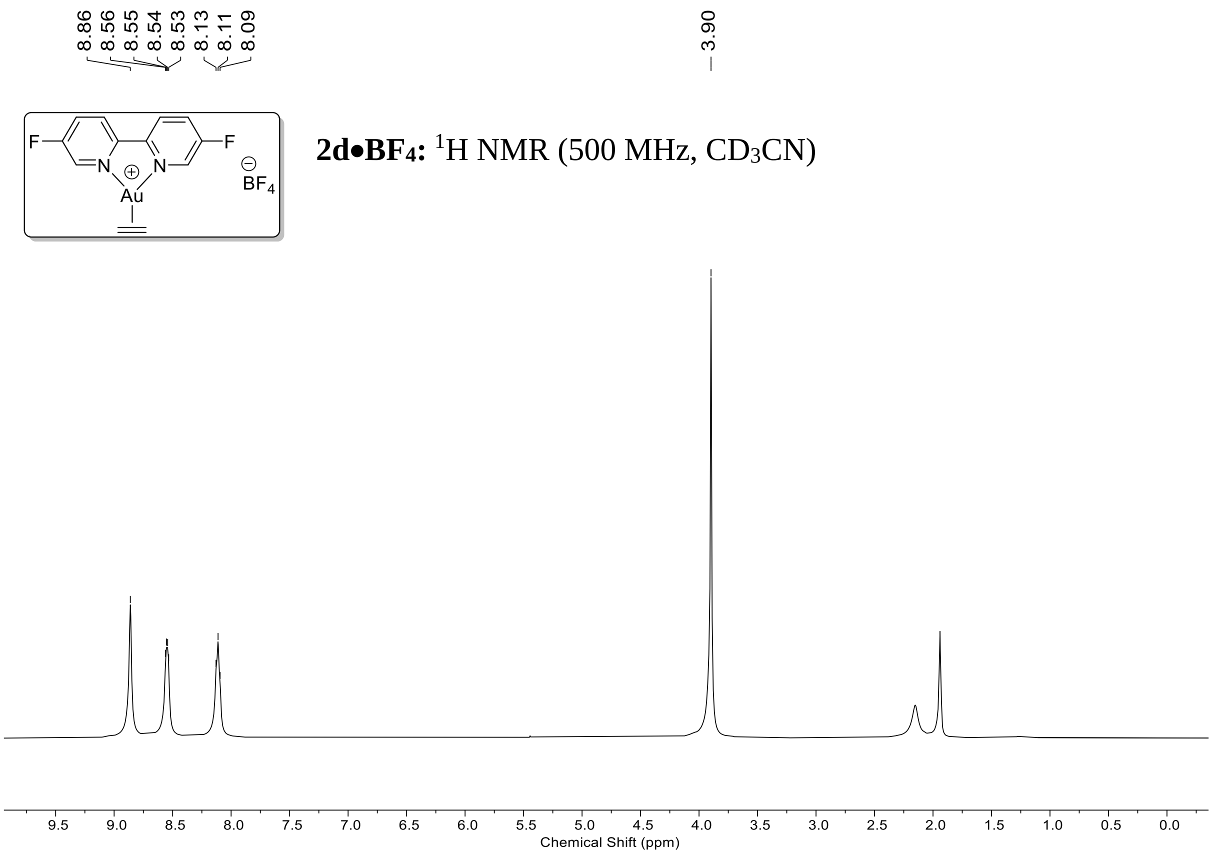

2d•BF_4: ^{1}H NMR (500 MHz, CD_3CN)

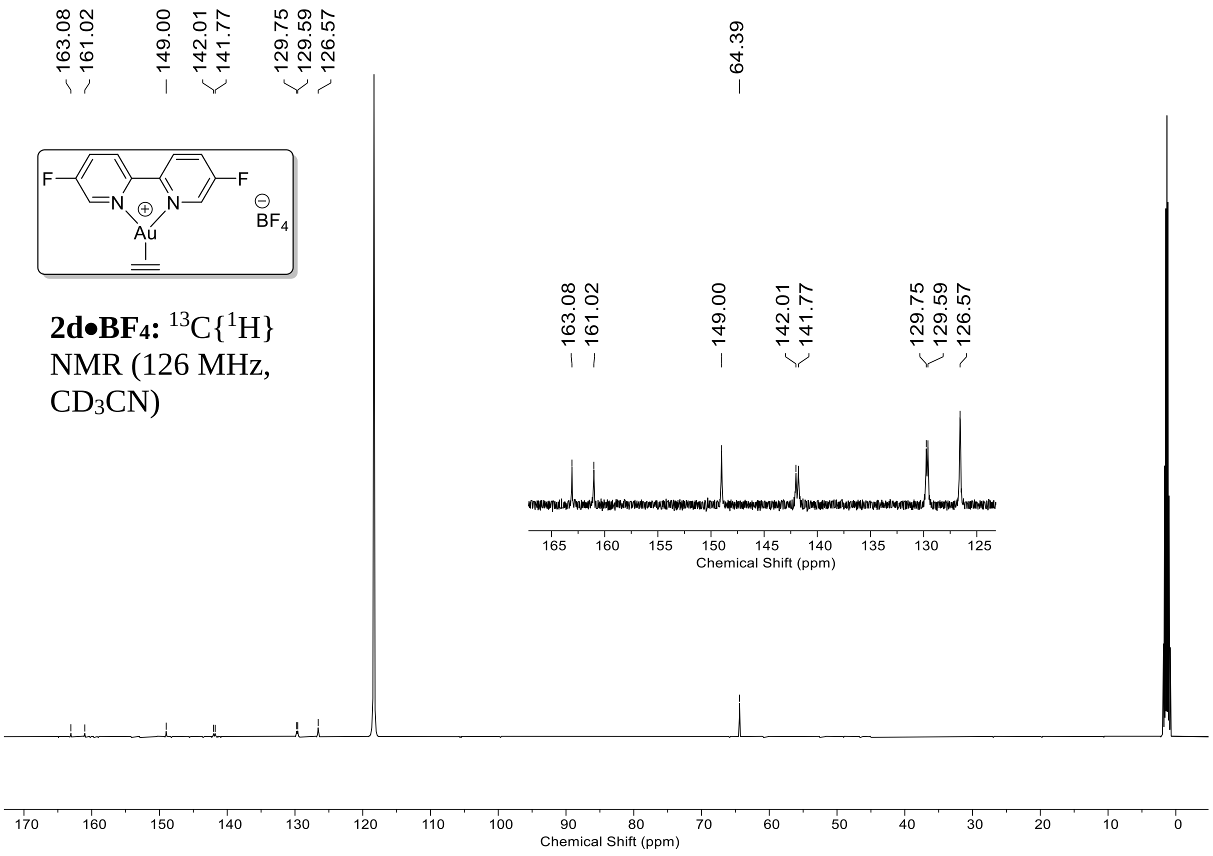

2d•BF_4: ^{13}C{^{1}H} NMR (126 MHz, CD_3CN)

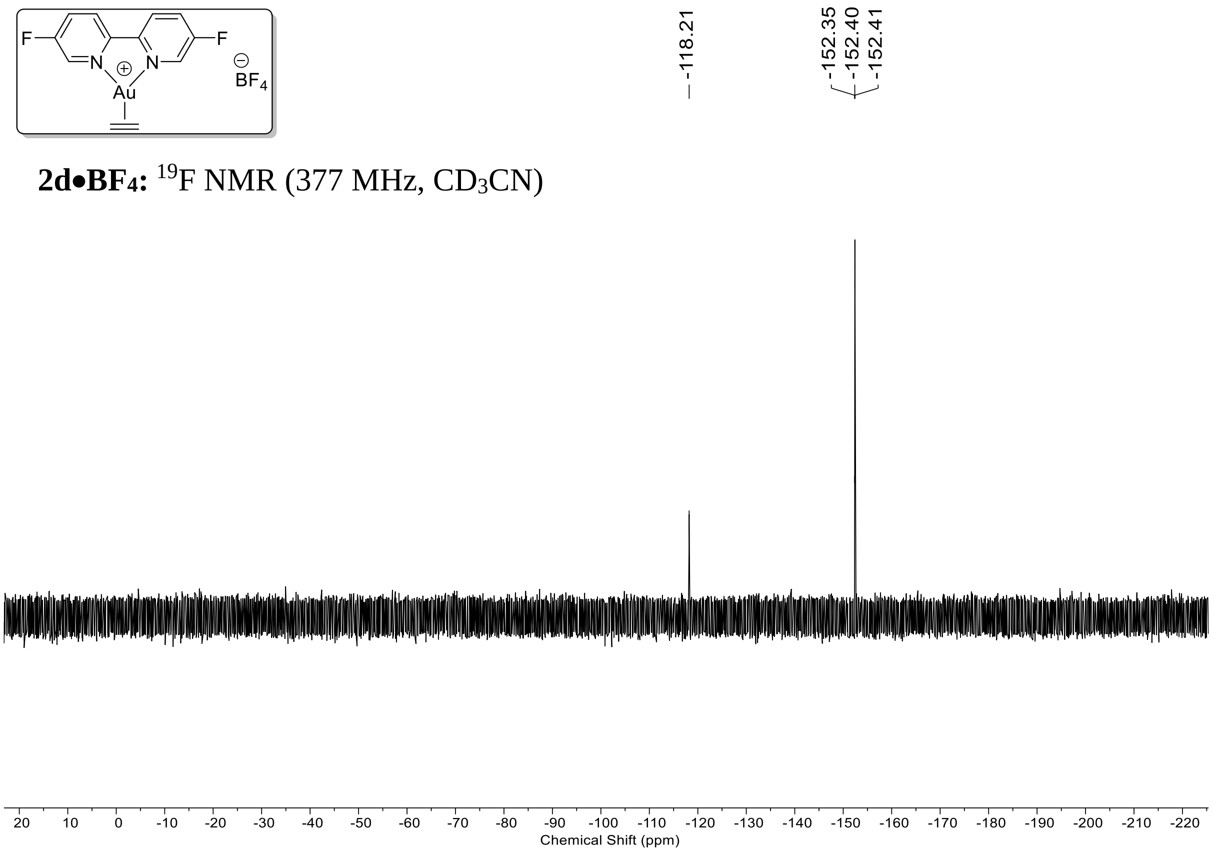

2d•BF$_4$: ^{19}F NMR (377 MHz, CD_3CN)

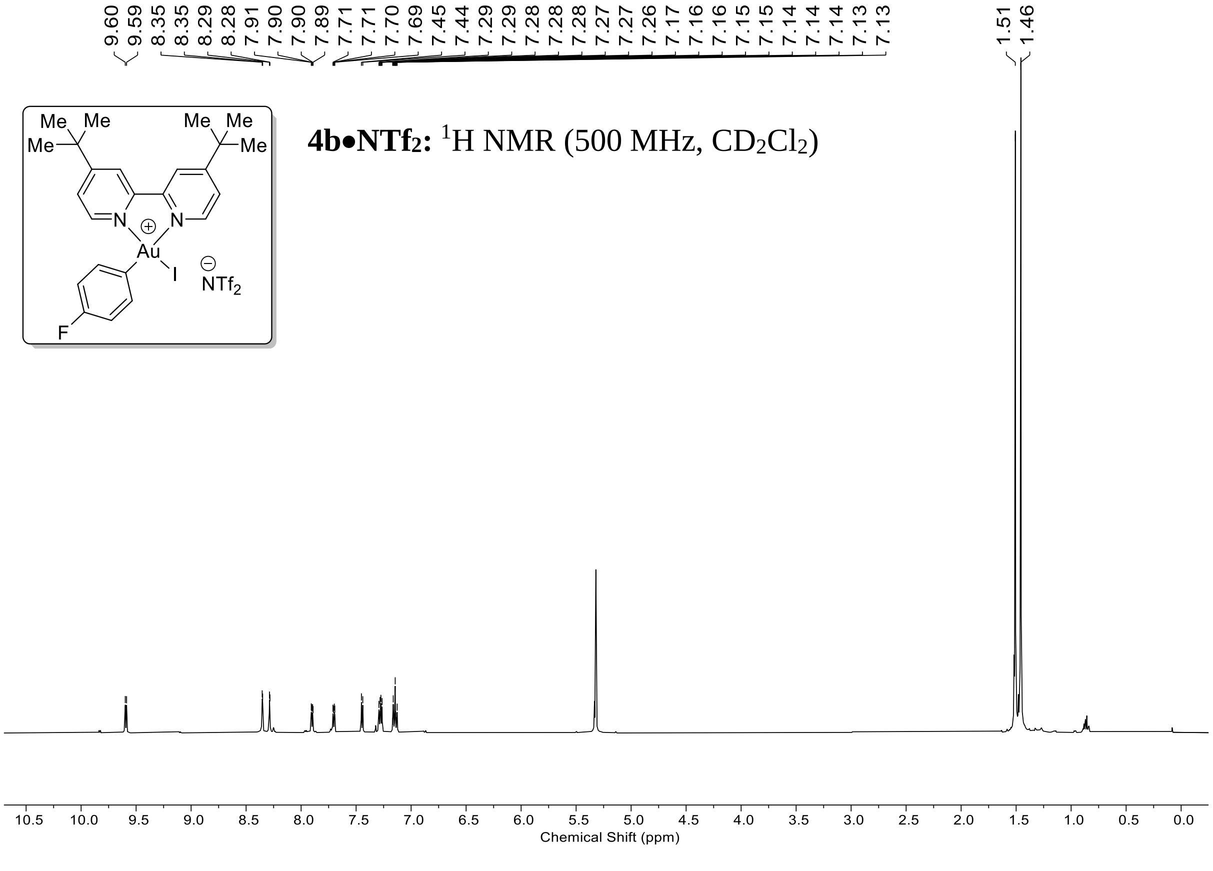

4b•NTf$_2$: ^{1}H NMR (500 MHz, CD_2Cl_2)

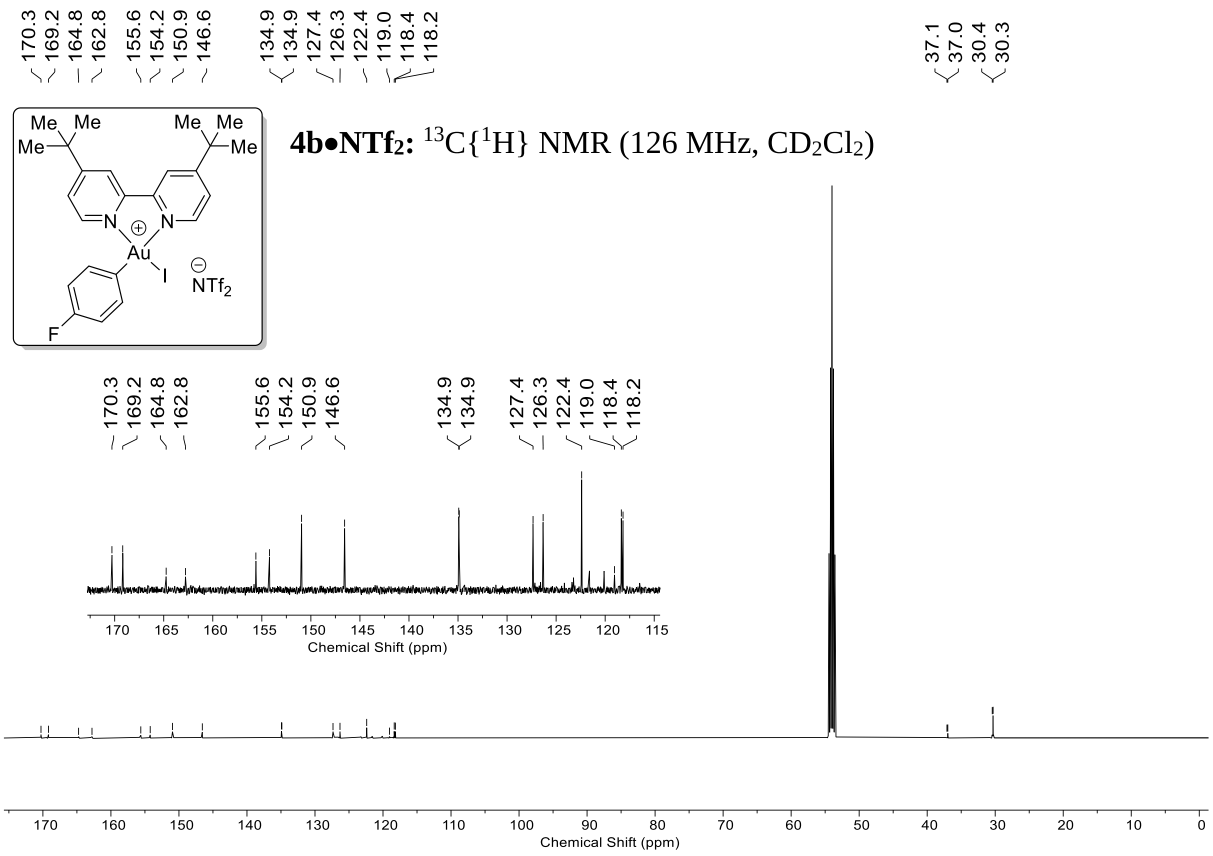

4b•NTf$_2$: ^{13}C{^{1}H} NMR (126 MHz, CD_2Cl_2)

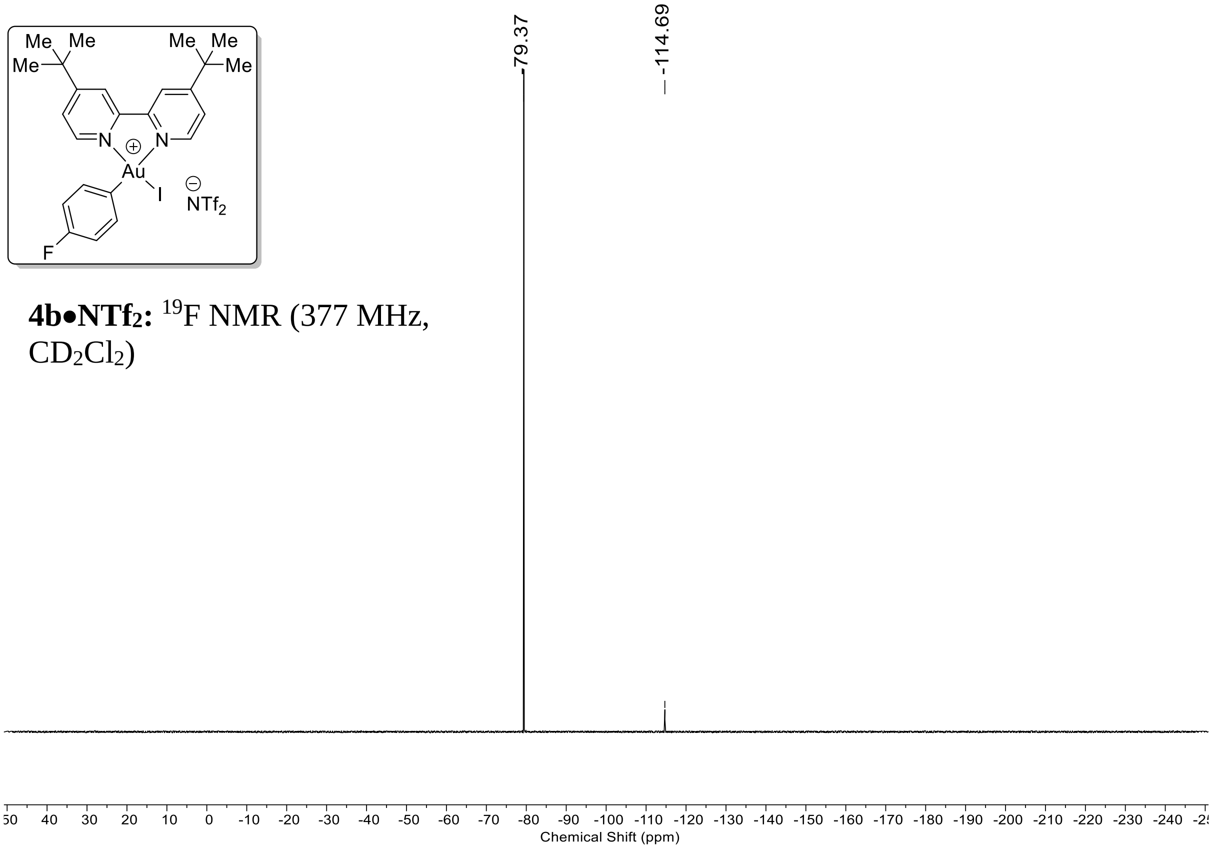

4b•NTf$_2$: ^{19}F NMR (377 MHz, CD_2Cl_2)

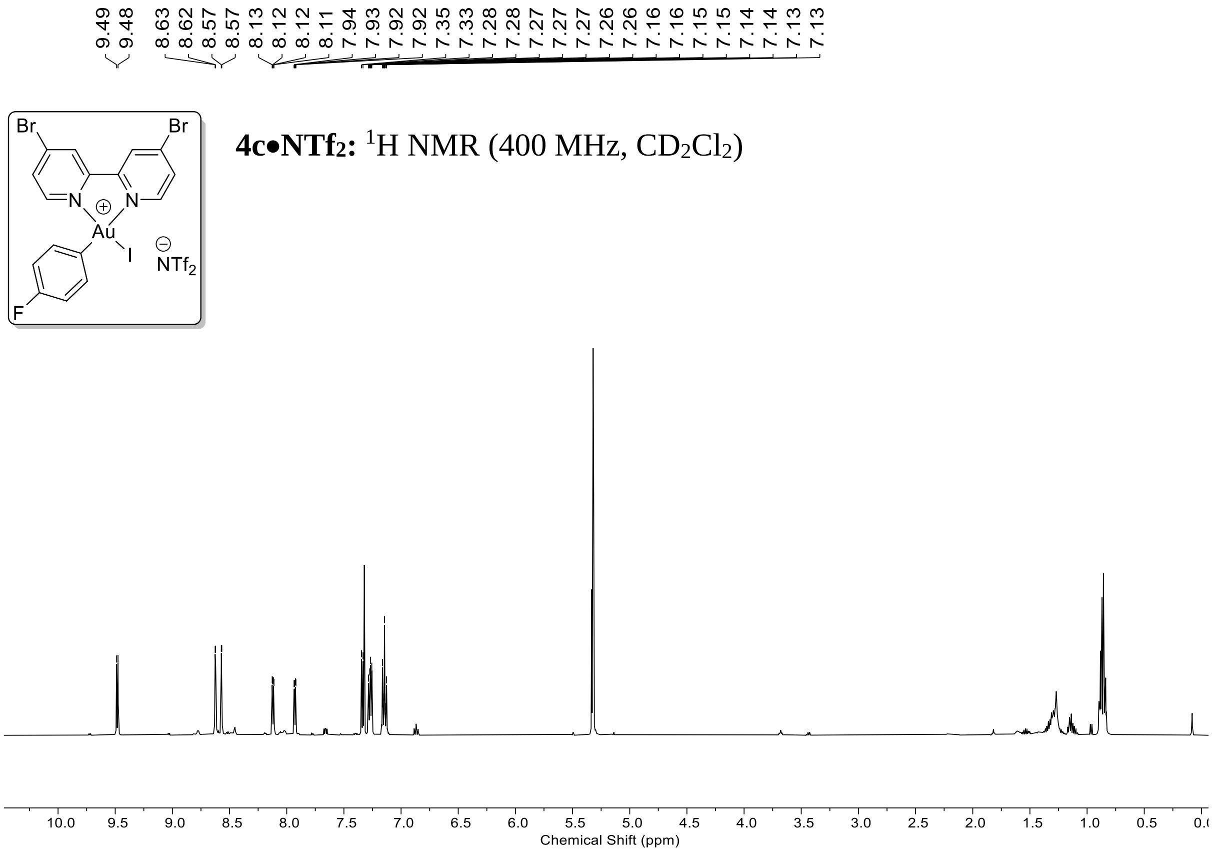

4c•NTf2: ^{1}H NMR (400 MHz, CD_2Cl_2)

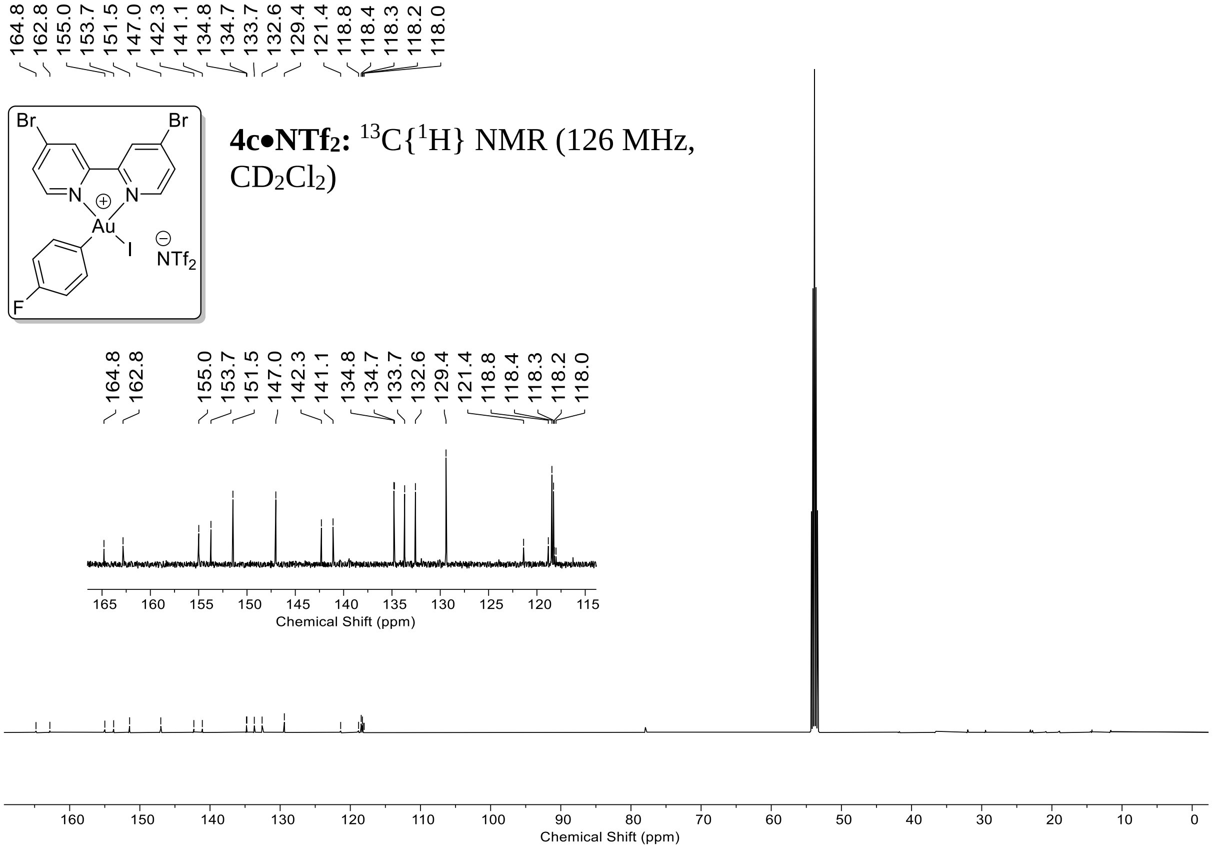

4c•NTf2: ^{13}C{^{1}H} NMR (126 MHz, CD_2Cl_2)

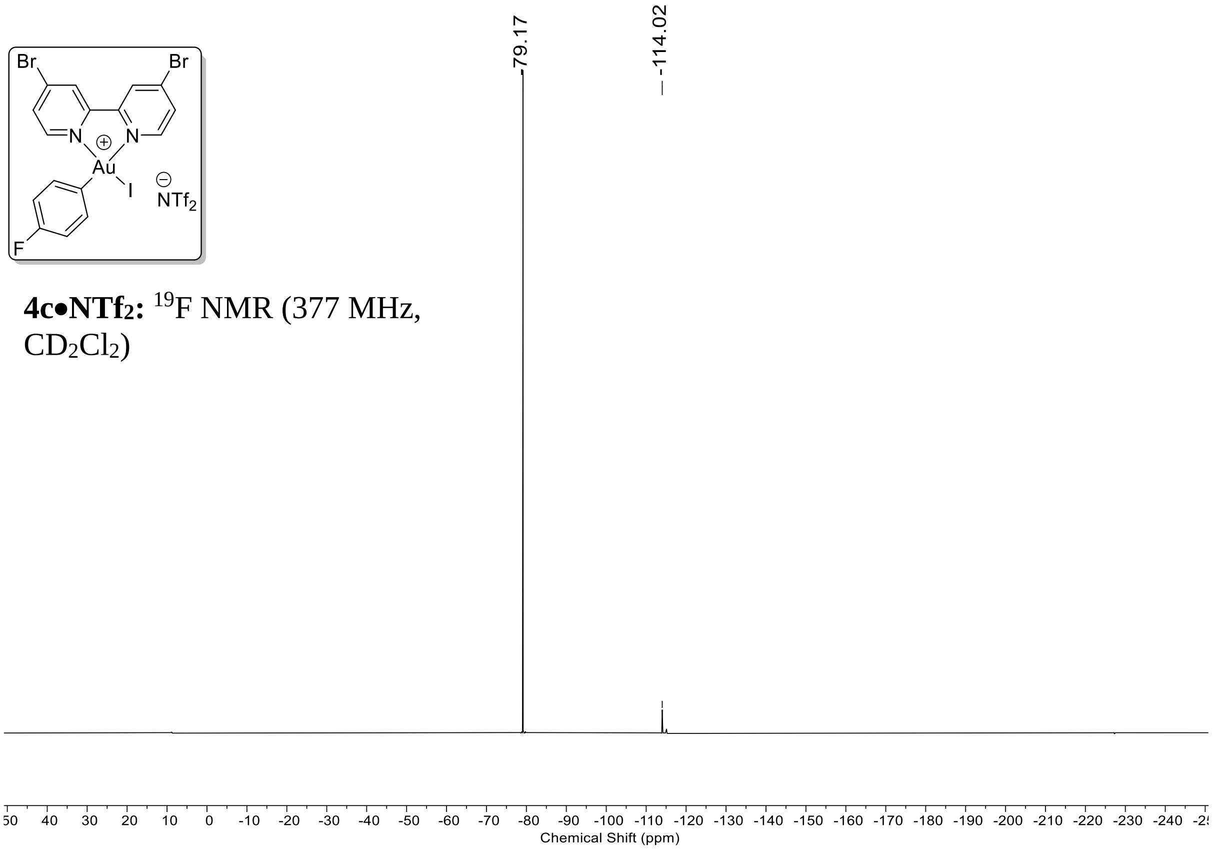

4c•NTf2: ^{19}F NMR (377 MHz, CD_2Cl_2)

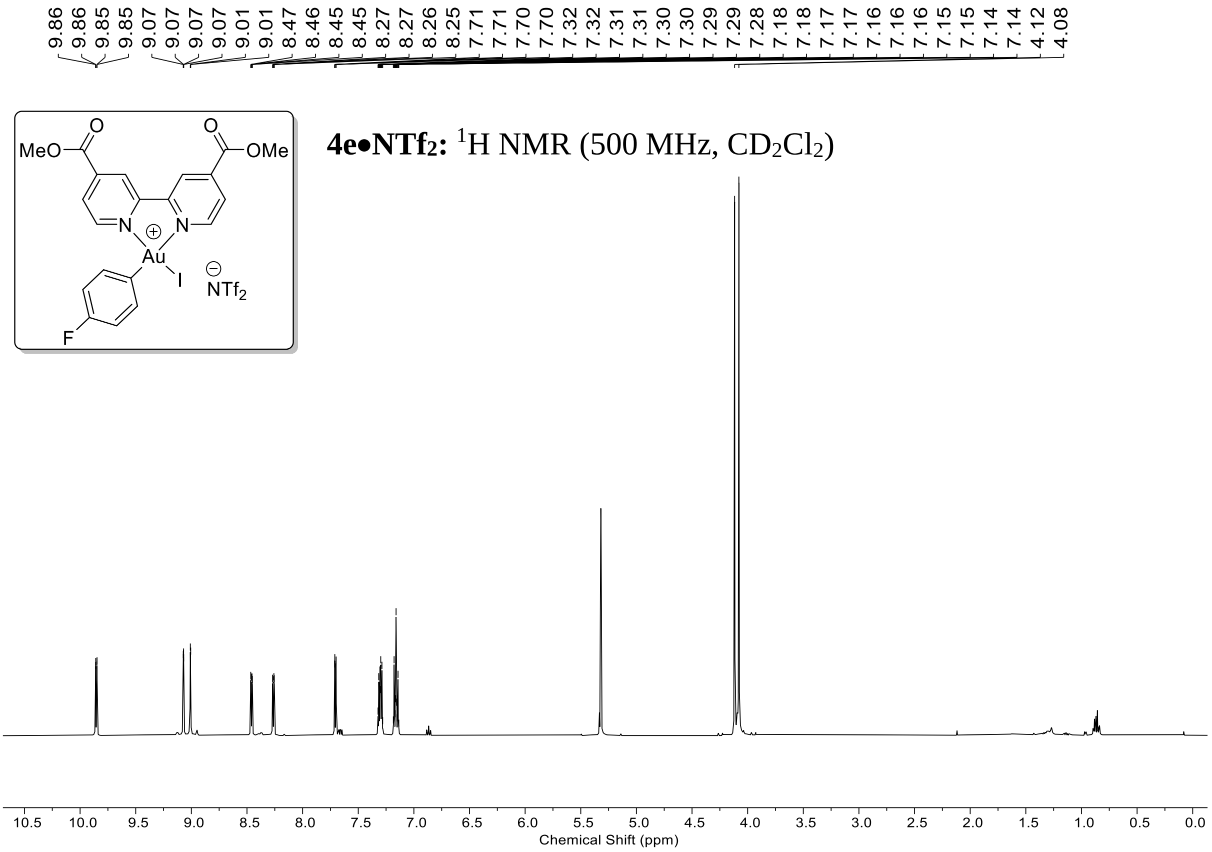

4e•NTf2: ^{1}H NMR (500 MHz, CD_2Cl_2)

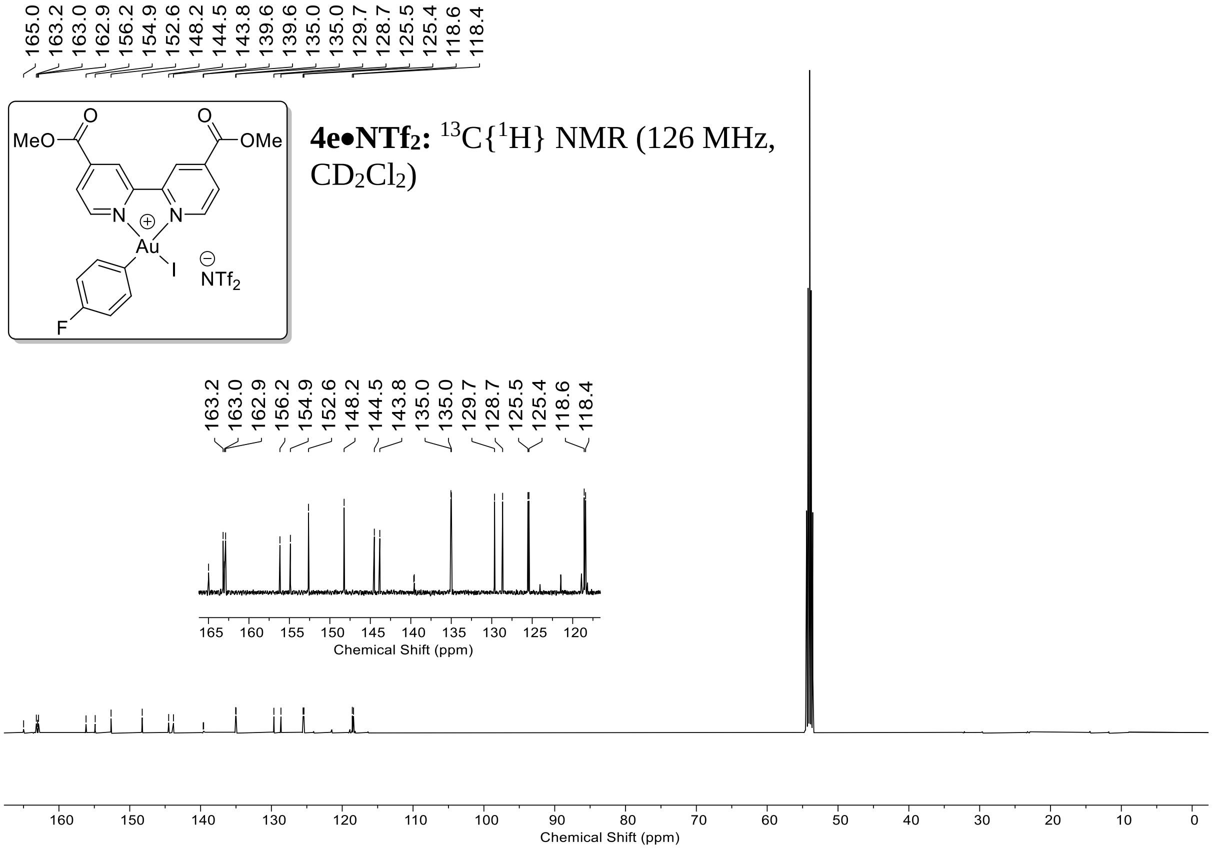

4e•NTf2: $^{13}C\{^{1}H\}$ NMR (126 MHz, CD_2Cl_2)

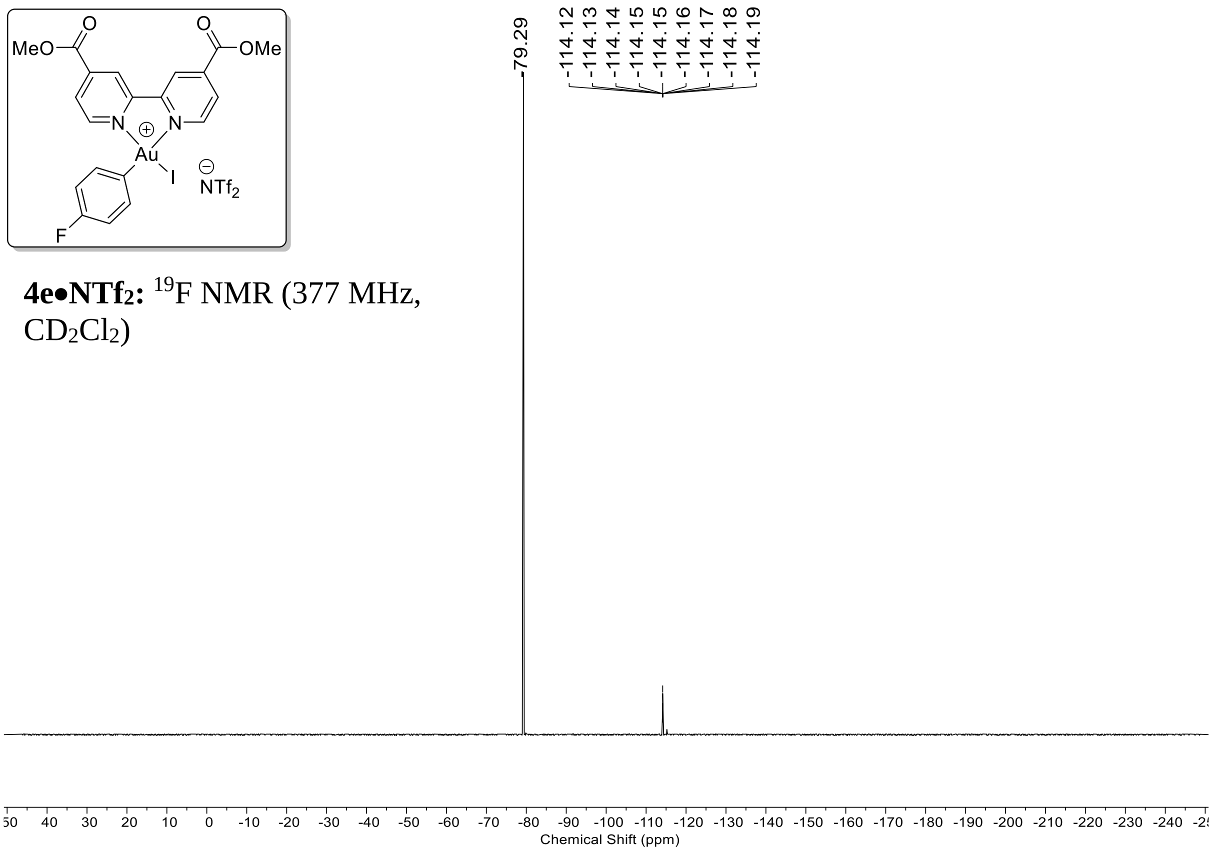

4e•NTf2: ^{19}F NMR (377 MHz, CD_2Cl_2)

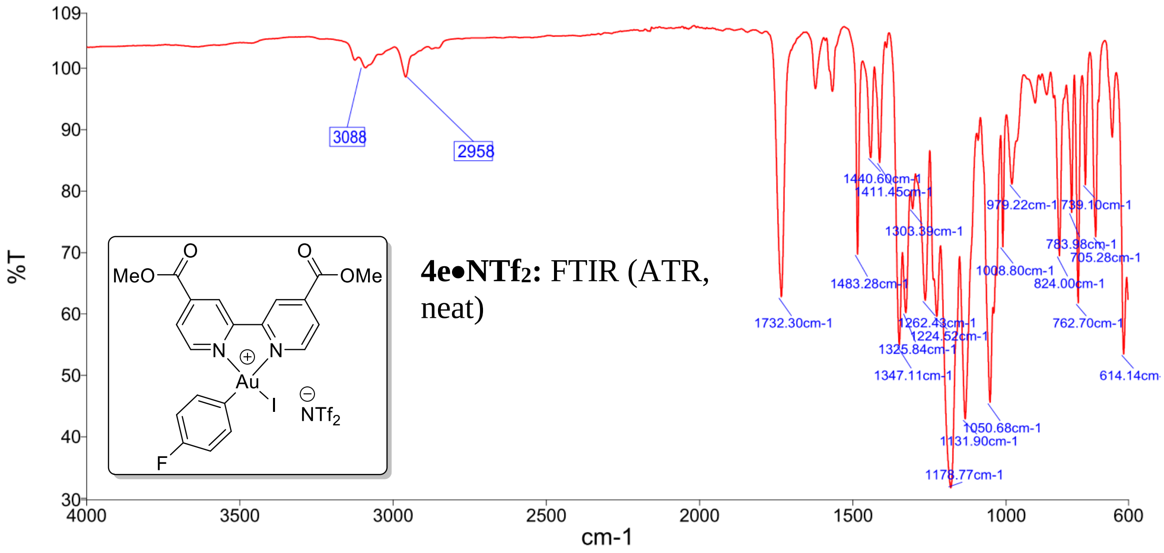

4e•NTf$_2$: FTIR (ATR, neat)

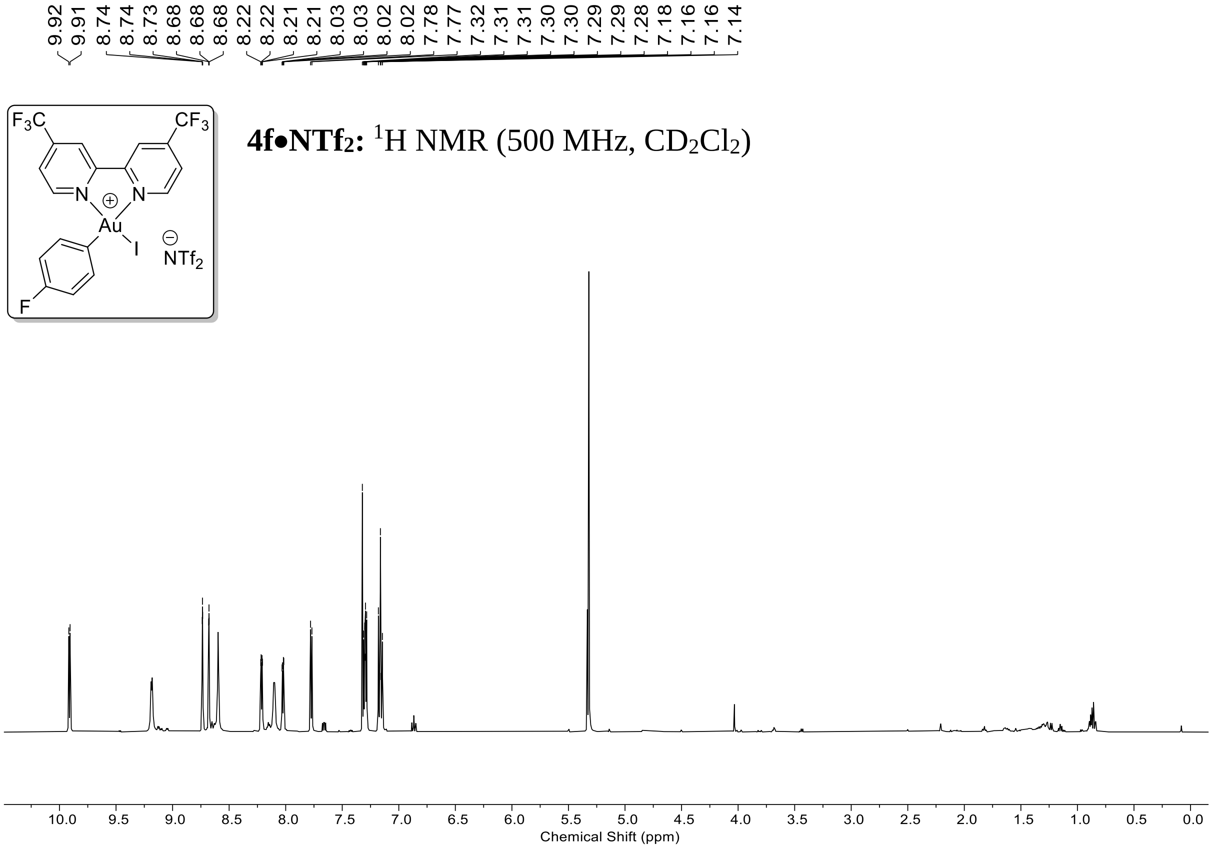

4f•NTf$_2$: ^{1}H NMR (500 MHz, CD_2Cl_2)

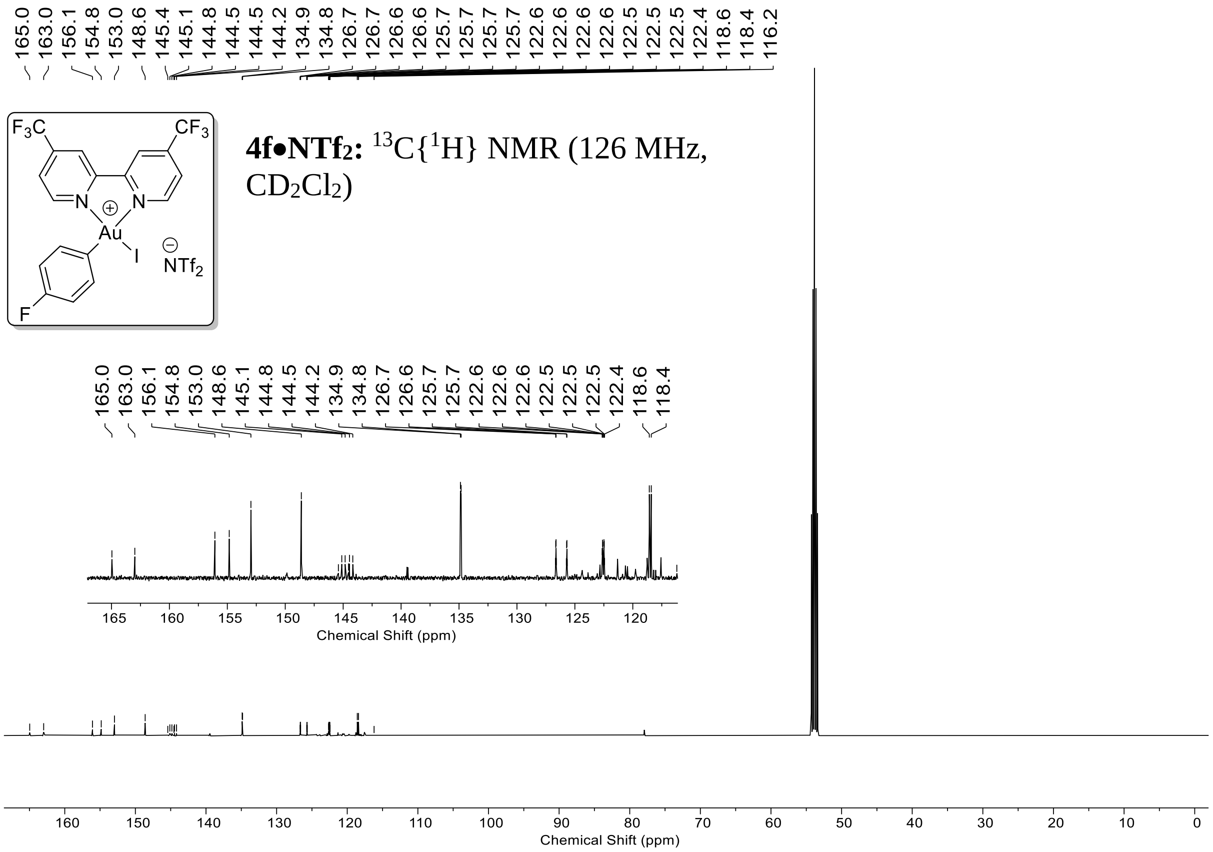

4f•NTf₂: $^{13}C\{^{1}H\}$ NMR (126 MHz, CD_2Cl_2)

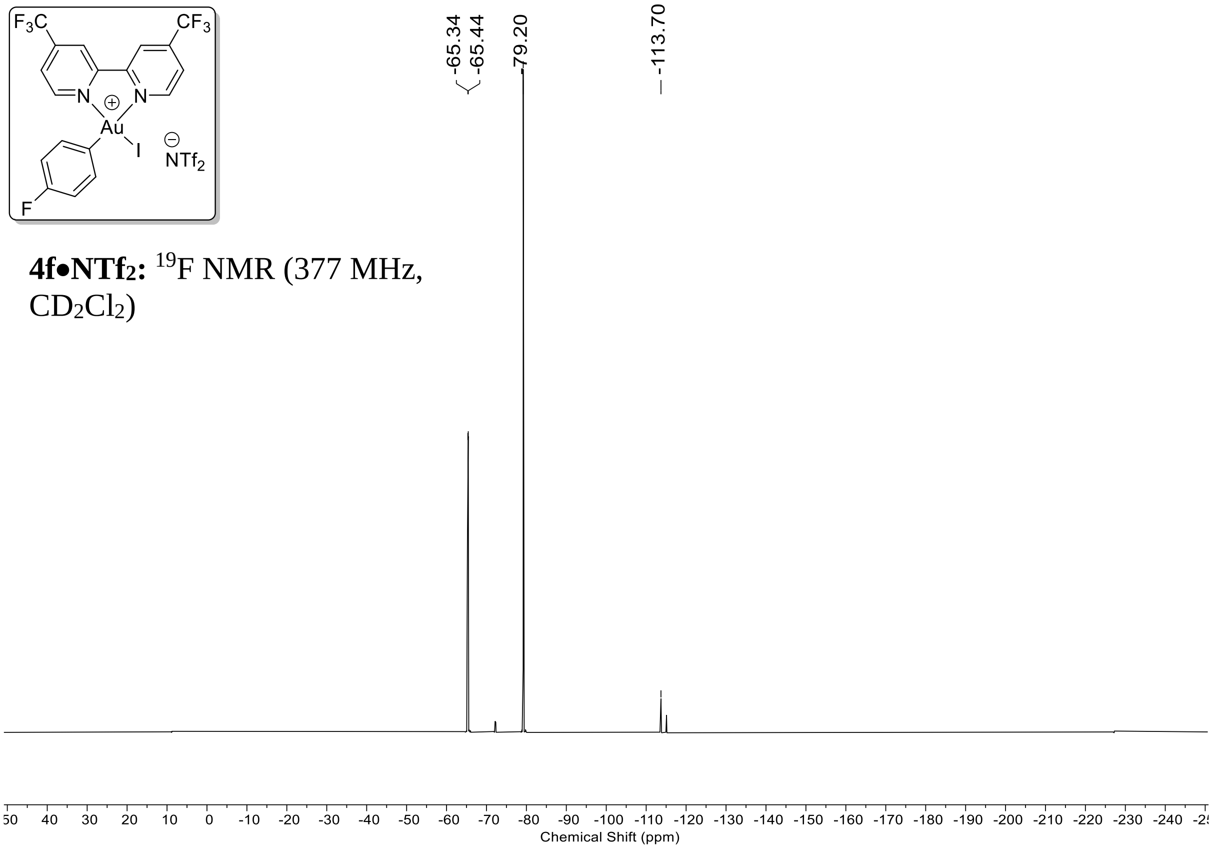

4f•NTf₂: ^{19}F NMR (377 MHz, CD_2Cl_2)

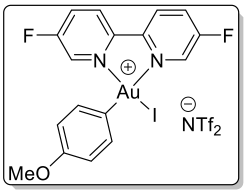

5c•NTf2: ^{1}H NMR (500 MHz, CD_2Cl_2)

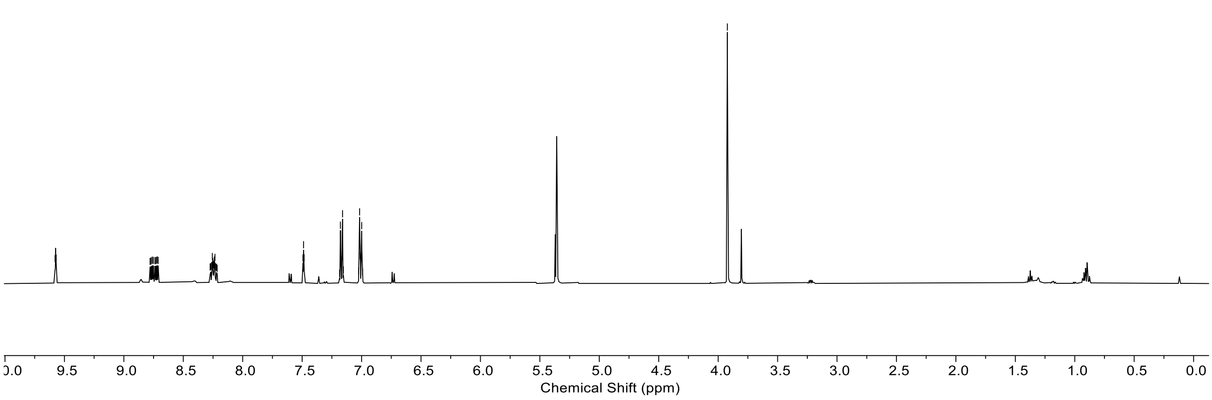

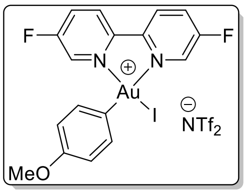

5c•NTf2: ^{13}C{^{1}H} NMR (126 MHz, CD_2Cl_2)

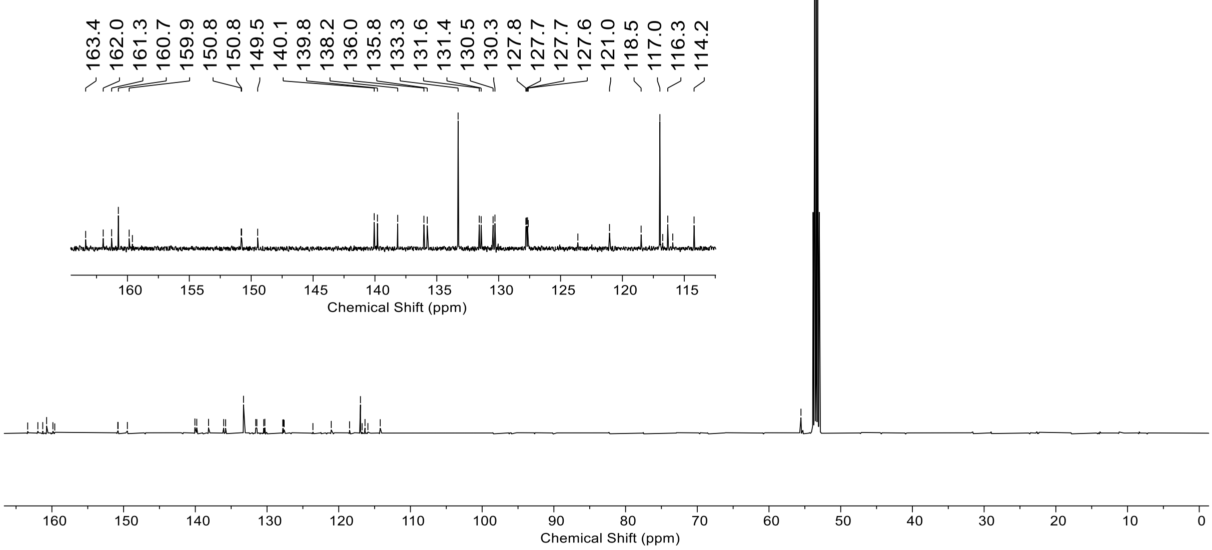

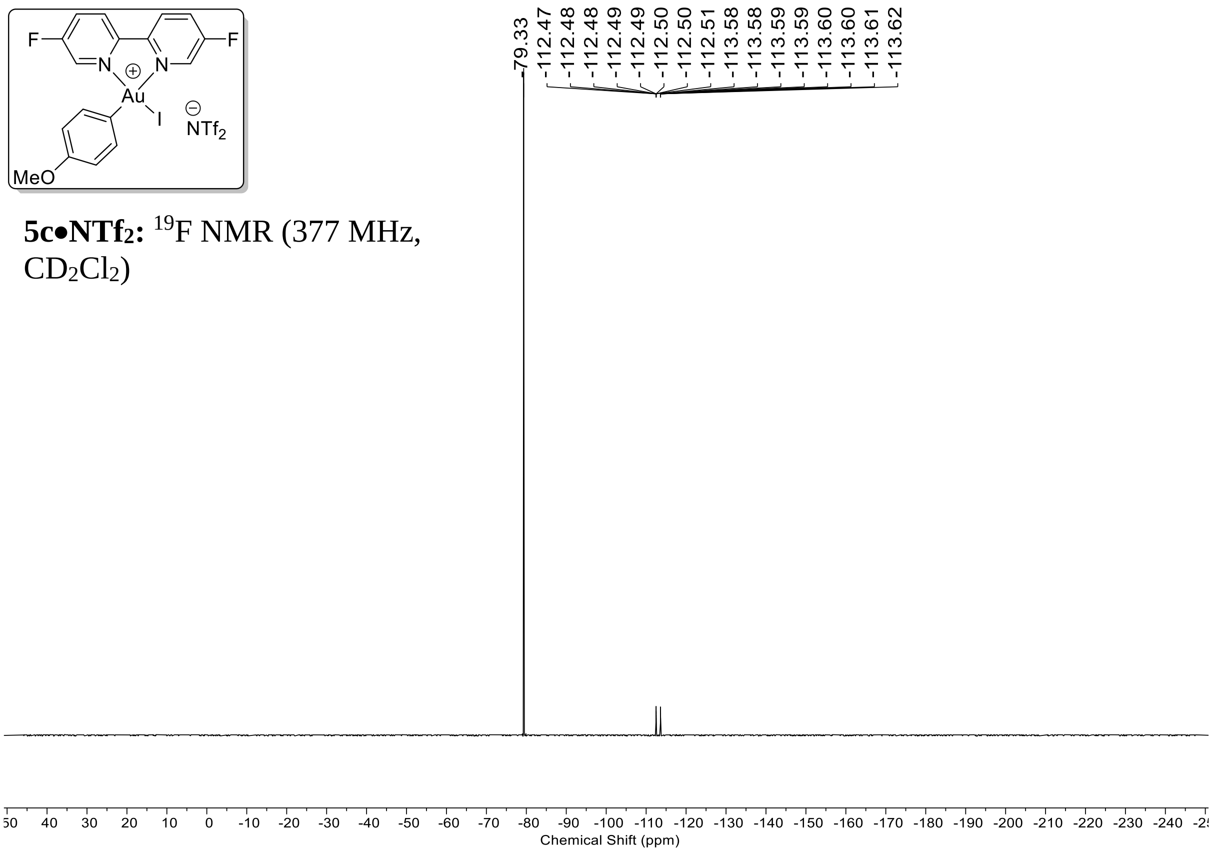

5c•NTf₂: ^{19}F NMR (377 MHz, CD_2Cl_2)

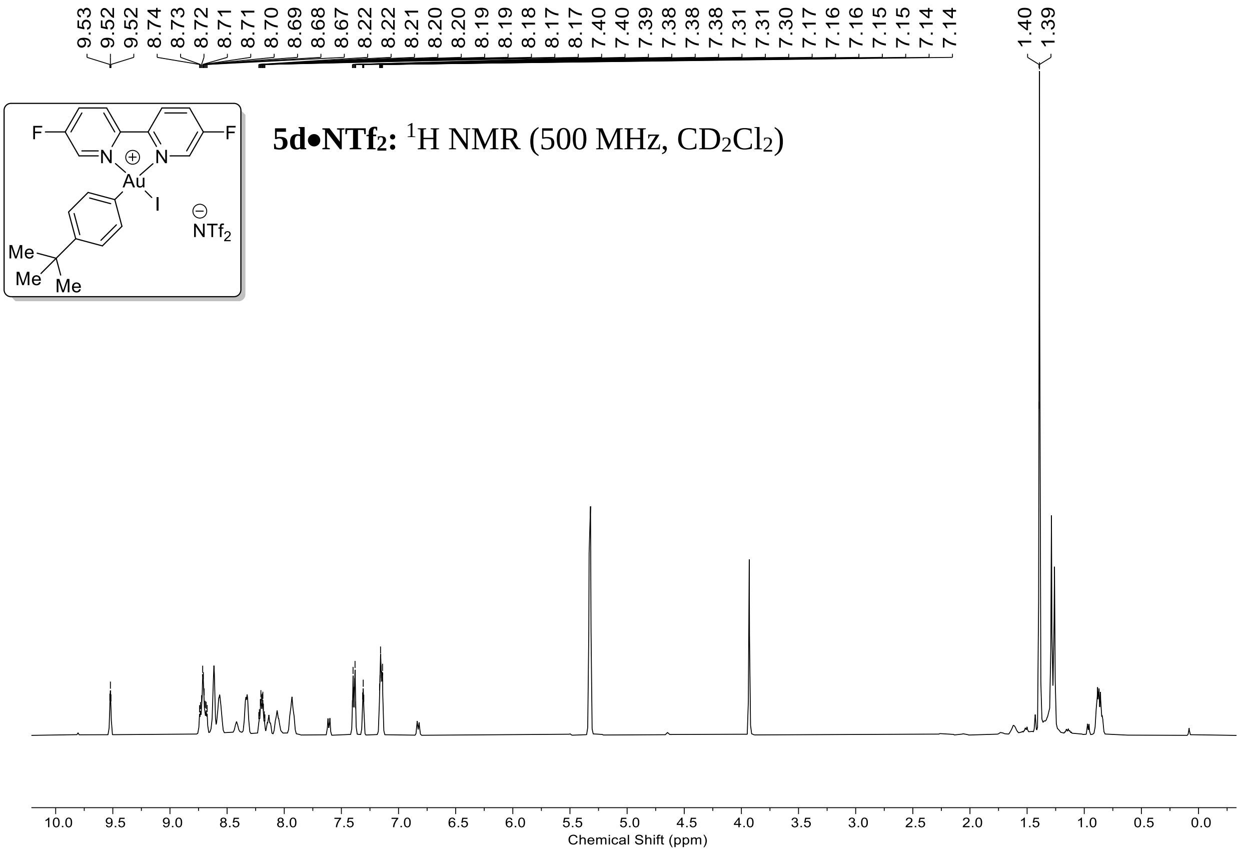

5d•NTf₂: ^{1}H NMR (500 MHz, CD_2Cl_2)

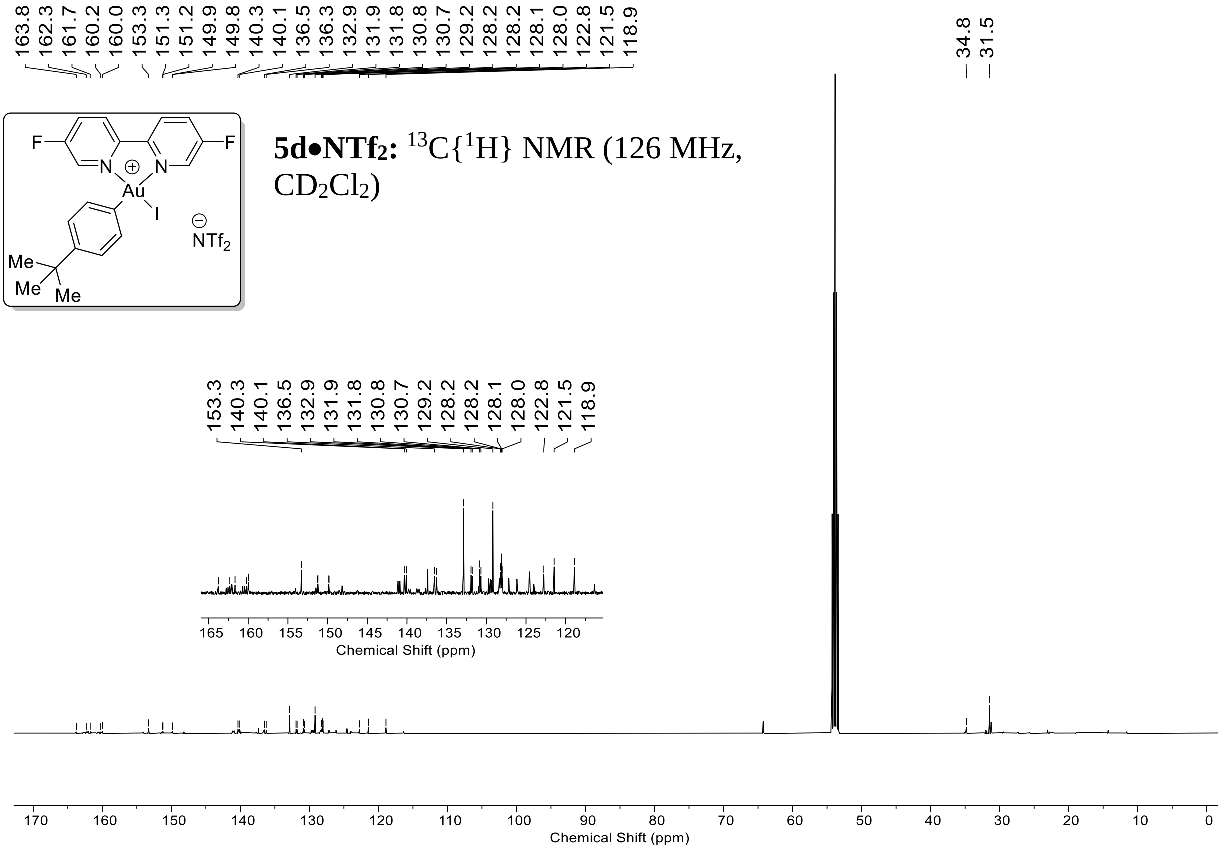

5d•NTf₂: $^{13}C\{^{1}H\}$ NMR (126 MHz, CD_2Cl_2)

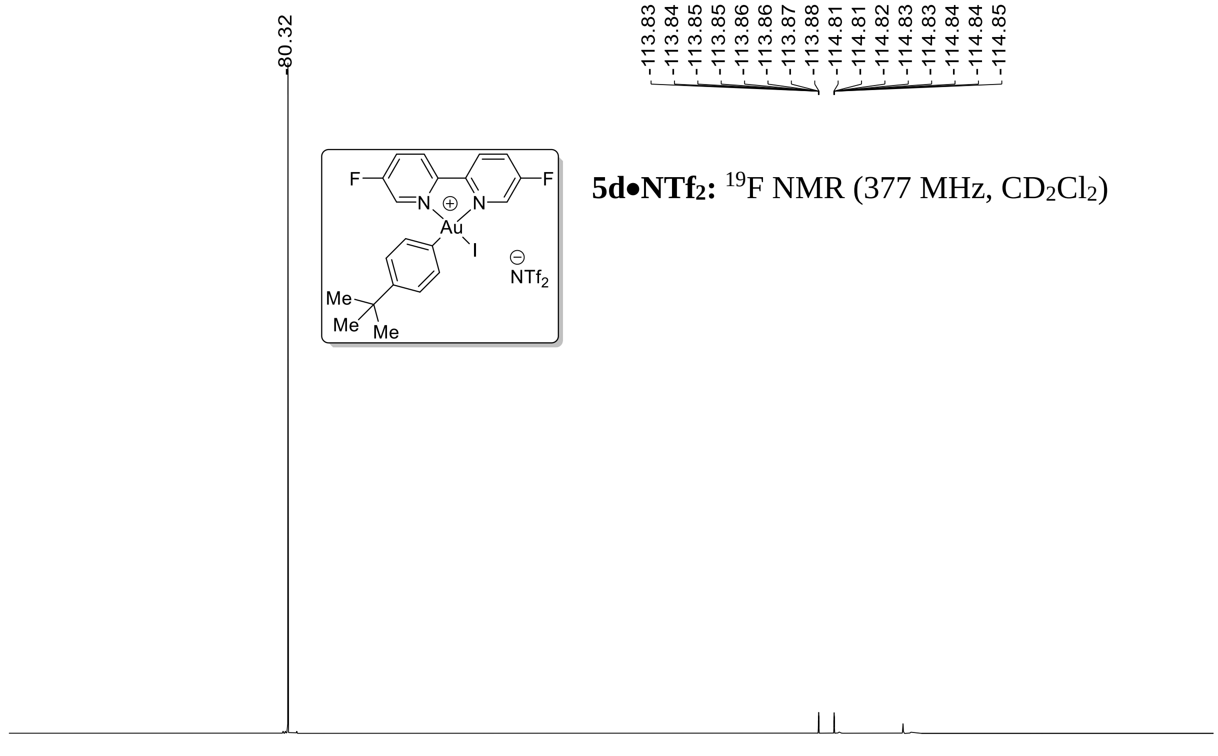

5d•NTf₂: ^{19}F NMR (377 MHz, CD_2Cl_2)

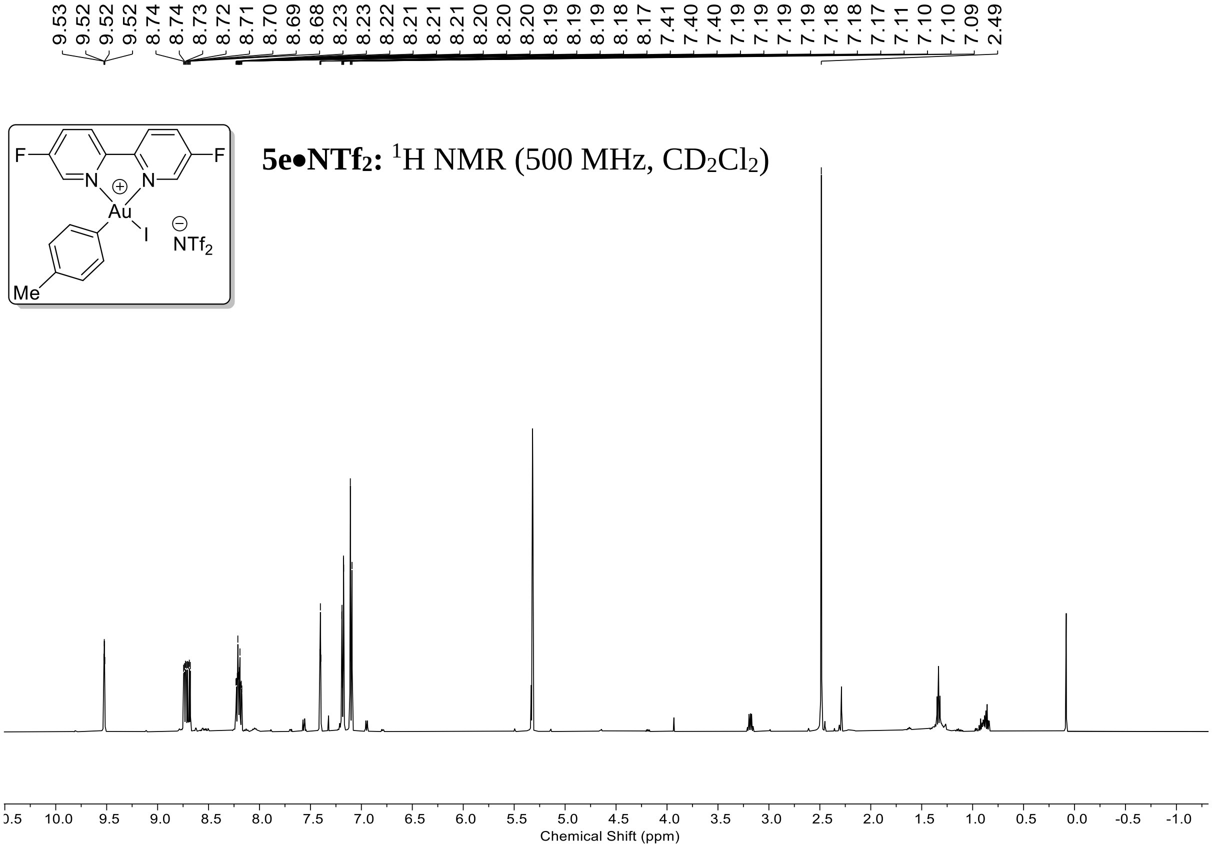

5e•NTf$_2$: ^{1}H NMR (500 MHz, CD_2Cl_2)

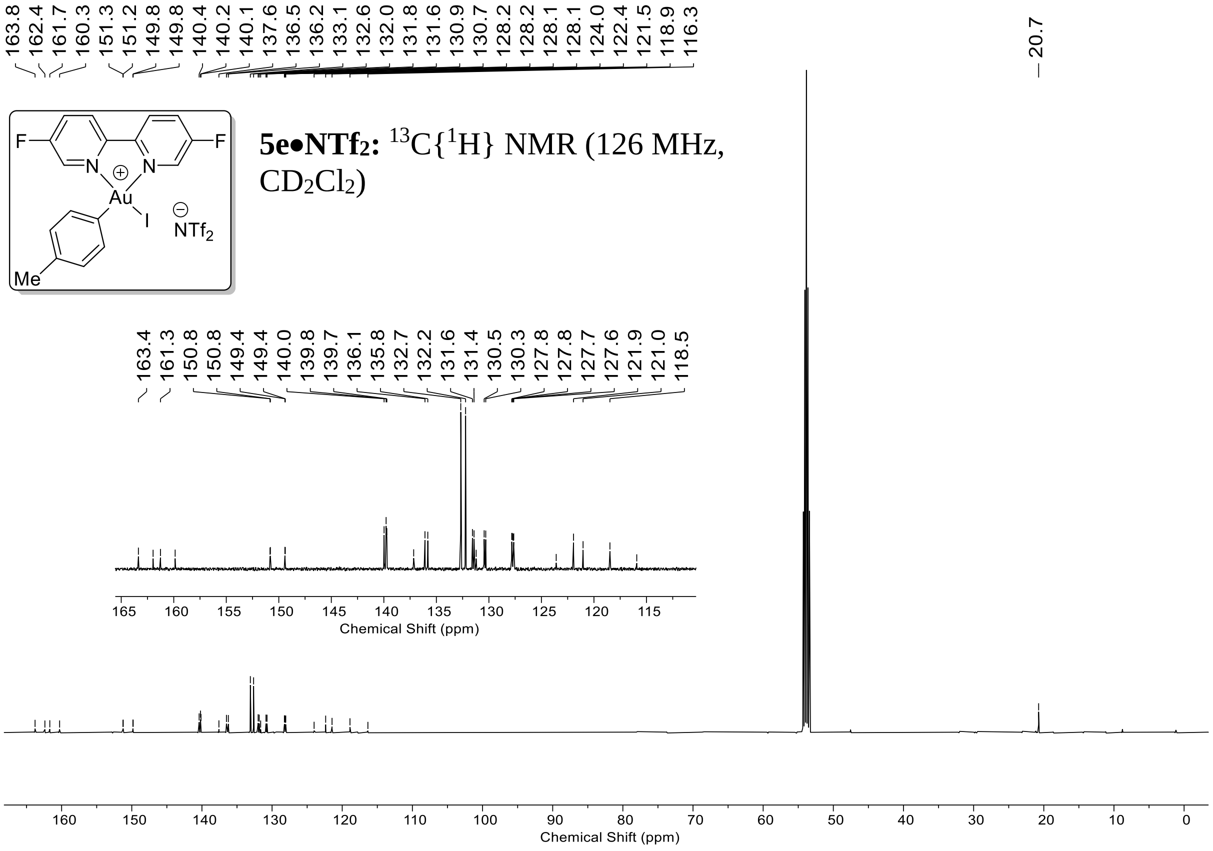

5e•NTf$_2$: ^{13}C{^{1}H} NMR (126 MHz, CD_2Cl_2)

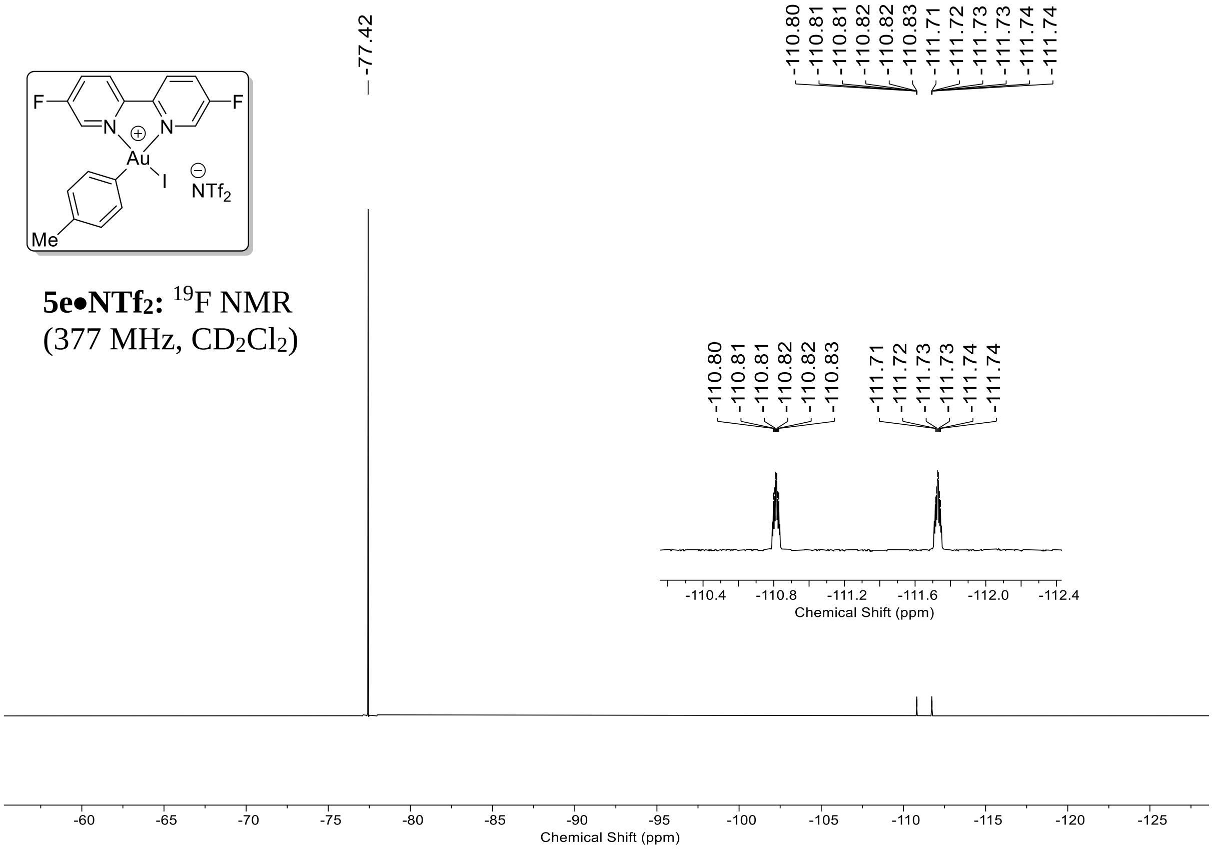

5e•NTf$_2$: ^{19}F NMR (377 MHz, CD_2Cl_2)

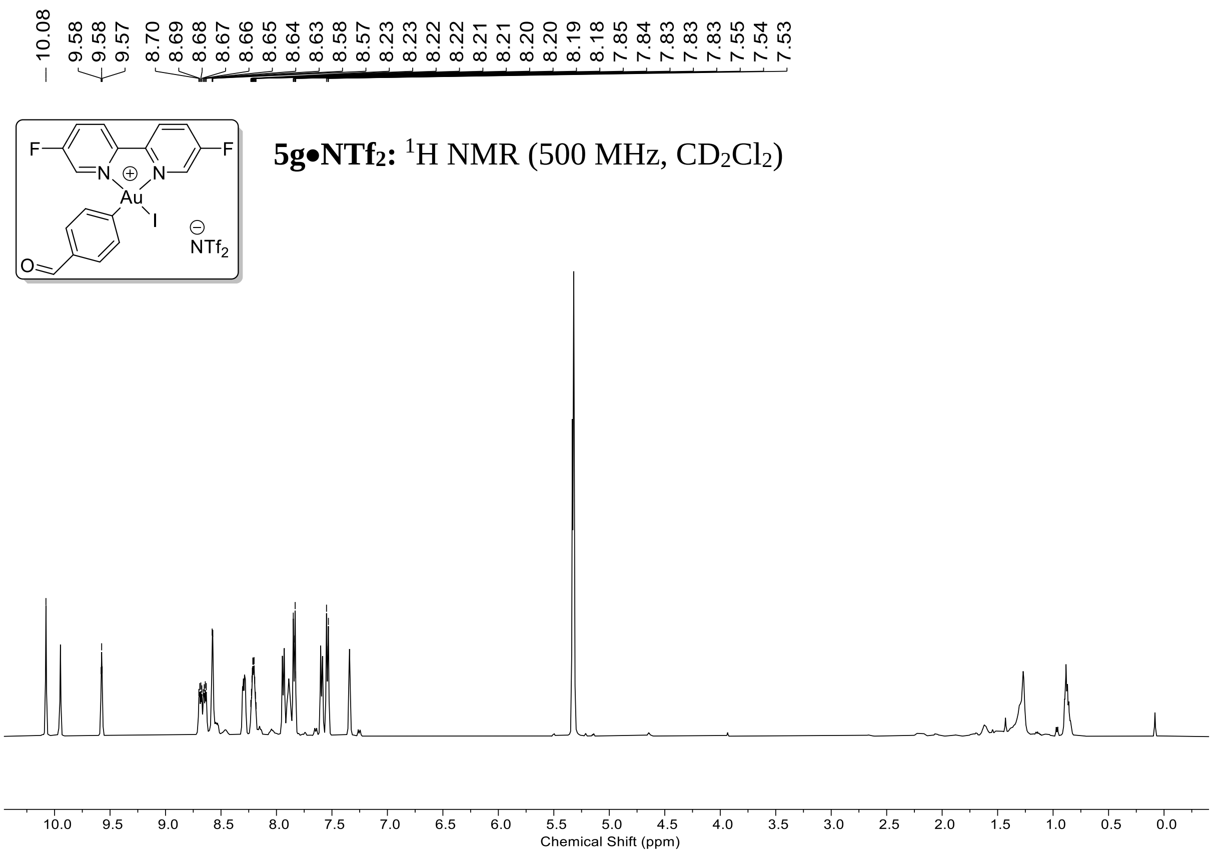

5g•NTf$_2$: ^{1}H NMR (500 MHz, CD_2Cl_2)

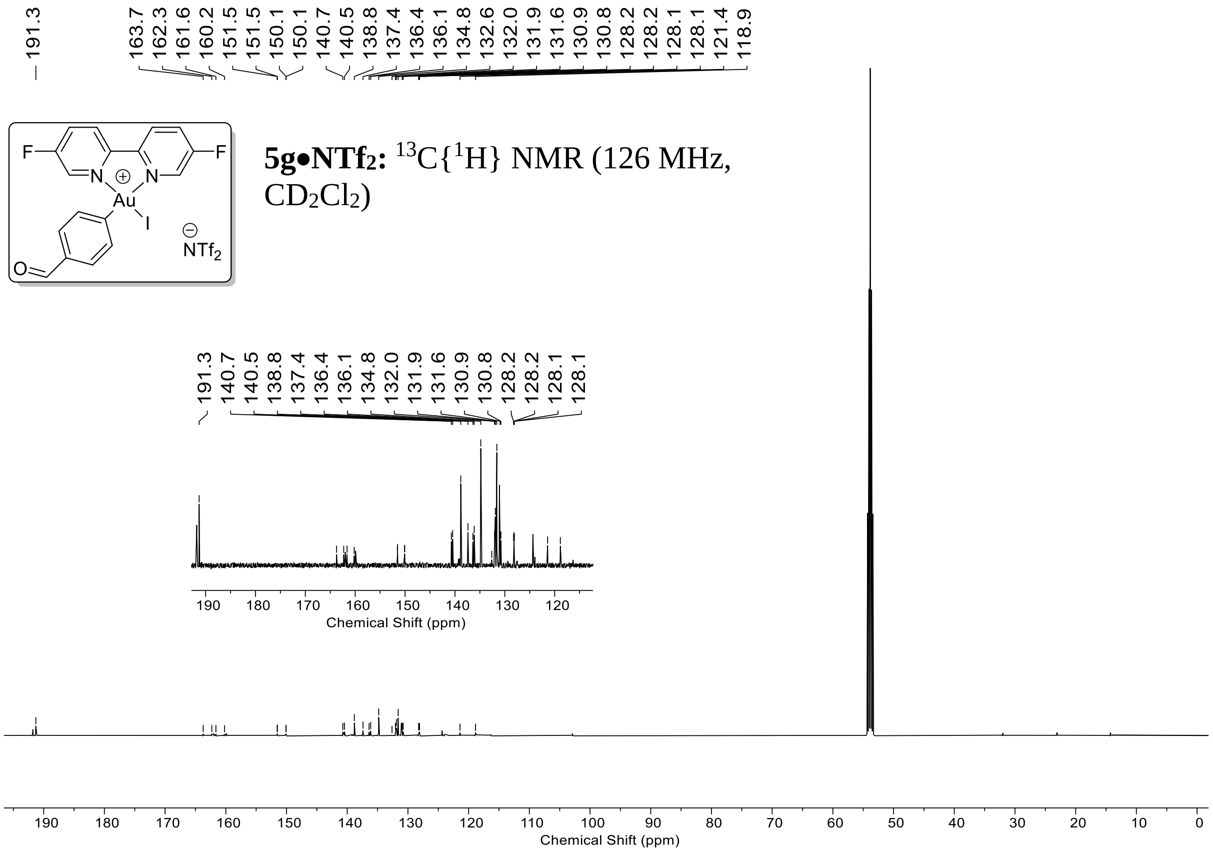

5g•NTf$_2$: $^{13}C\{^{1}H\}$ NMR (126 MHz, CD_2Cl_2)

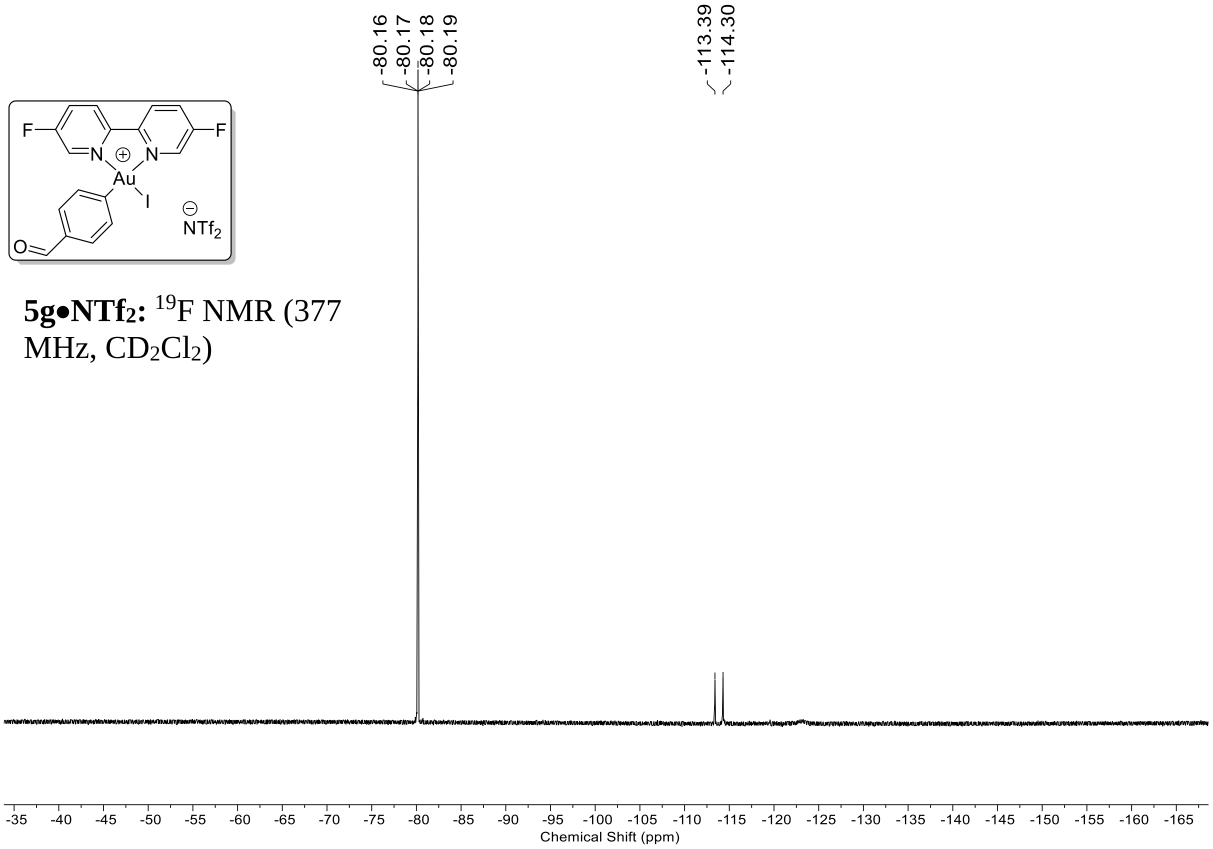

5g•NTf$_2$: ^{19}F NMR (377 MHz, CD_2Cl_2)

7. References

[1] A. B. Pangborn, M. A. Giardello, R. H. Grubbs, R. K. Rosen, F. J. Timmers, *Organometallics* **1996**, *15*, 1518.

[2] L. Shi, B. Zhang; Preparing method of entecavir intermediate. CN108586460 (A), 2018.

[3] H. Cao, H. Jiang, H. Feng, J. M. C. Kwan, X. Liu, J. Wu, *J. Am. Chem. Soc.* **2018**, *140*, 16360.

[4] K. Lee, P. H. Lee, *Tetrahedron Lett.* **2008**, *49*, 4302.

[5] M. J. Harper, C. J. Arthur, J. Crosby, E. J. Emmett, R. L. Falconer, A. J. Fensham-Smith, P. J. Gates, T. Leman, J. E. McGrady, J. F. Bower, C. A. Russell, *J. Am. Chem. Soc.* **2018**, *140*, 4440.

[6] D. Miyoshi, H. Karimata, Z.-M. Wang, K. Koumoto, N. Sugimoto, *J. Am. Chem. Soc.* **2007**, *129*, 5919.

[7] H. Li, J. Oppenheimer, M. R. Smith, R. E. Maleczka, *Tetrahedron Lett.* **2016**, *57*, 2231.

[8] A. Duong, T. Maris, O. Lebel, J. D. Wuest, *J. Org. Chem.* **2011**, *76*, 1333.

[9] X. Huang, Z. Lu, Z. Wang, C. Fan, W. Fan, X. Shi, H. Zhang, M. Pei, *Dyes Pigm.* **2016**, *128*, 33.

[10] Bruker, SAINT+ v8.38A Integration Engine, Data Reduction Software, Bruker Analytical X-ray Instruments Inc.: Madison, WI, U.S.A., 2015.

[11] Bruker, SADABS 2014/15, Bruker AXS area detector scaling and absorbtion correction, Bruker Analytical X-ray Instruments Inc.: Madison, WI, U.S.A., 2014/15.

[12] (a) L. Palatinus, G. Chapuis, *J. Appl. Crystallogr.* **2007**, *40*, 786; (b) L. Palatinus, A. van der Lee, *J. Appl. Crystallogr.* **2008**, *41*, 975; (c) L. Palatinus, S. J. Prathapa, S. van Smaalen, *J. Appl. Crystallogr.* **2012**, *45*, 575.

[13] G. Sheldrick, *Acta Crystallogr., Sect. A* **2015**, *71*, 3.

[14] G. Sheldrick, *Acta Crystallogr., Sect. C* **2015**, *71*, 3.

[15] O. V. Dolomanov, L. J. Bourhis, R. J. Gildea, J. A. K. Howard, H. Puschmann, *J. Appl. Crystallogr.* **2009**, *42*, 339.

[16] D. S. Raiford, C. L. Fisk, E. D. Becker, *Anal. Chem.* **1979**, *51*, 2050.

[17] C. Hansch, A. Leo, R. W. Taft, *Chem. Rev.* **1991**, *91*, 165.

[18] M. J. Frisch, G. W. Trucks, H. B. Schlegel, G. E. Scuseria, M. A. Robb, J. R. Cheeseman, G. Scalmani, V. Barone, G. A. Petersson, H. Nakatsuji, X. Li, M. Caricato, A. Marenich, J. Bloino, B. G. Janesko, R. Gomperts, B. Mennucci, H. P. Hratchian, J. V. Ortiz, A. F. Izmaylov, J. L. Sonnenberg, D. Williams-Young, F. Ding, F. Lipparini,

F. Egidi, J. Goings, B. Peng, A. Petrone, T. Henderson, D. Ranasinghe, V. G. Zakrzewski, J. Gao, N. Rega, G. Zheng, W. Liang, M. Hada, M. Ehara, K. Toyota, R. Fukuda, J. Hasegawa, M. Ishida, T. Nakajima, Y. Honda, O. Kitao, H. Nakai, T. Vreven, K. Throssell, J. J. A. Montgomery, J. E. Peralta, F. Ogliaro, M. Bearpark, J. J. Heyd, E. Brothers, K. N. Kudin, V. N. Staroverov, T. Keith, R. Kobayashi, J. Normand, K. Raghavachari, A. Rendell, J. C. Burant, S. S. Iyengar, J. Tomasi, M. Cossi, J. M. Millam, M. Klene, C. Adamo, R. Cammi, J. W. Ochterski, R. L. Martin, K. Morokuma, O. Farkas, J. B. Foresman, D. J. Fox, Gaussian 09 Rev. D.01, Wallingford, CT, 2009.

[19] J.-D. Chai, M. Head-Gordon, *Phys. Chem. Chem. Phys.* **2008**, *10*, 6615.

[20] F. Weigend, R. Ahlrichs, *Phys. Chem. Chem. Phys.* **2005**, *7*, 3297.

[21] (a) D. Andrae, U. Häußermann, M. Dolg, H. Stoll, H. Preuß, *Theor. Chim. Acta* **1990**, *77*, 123; (b) K. A. Peterson, D. Figgen, E. Goll, H. Stoll, M. Dolg, *J. Chem. Phys.* **2003**, *119*, 11113; (c) B. P. Pritchard, D. Altarawy, B. Didier, T. D. Gibson, T. L. Windus, *J. Chem. Inf. Model.* **2019**, *59*, 4814.

[22] A. V. Marenich, C. J. Cramer, D. G. Truhlar, *J. Phys. Chem. B* **2009**, *113*, 6378.

[23] (a) M. A. Cinellu, G. Minghetti, F. Cocco, S. Stoccoro, A. Zucca, M. Manassero, M. Arca, *Dalton Trans.* **2006**, , 5703; (b) M. Navarro, A. Toledo, M. Joost, A. Amgoune, S. Mallet-Ladeira, D. Bourissou, *Chem. Commun.* **2019**, *55*, 7974; (c) M. Navarro, A. Toledo, S. Mallet-Ladeira, E. D. Sosa Carrizo, K. Miqueu, D. Bourissou, *Chem. Sci.* **2020**, *11*, 2750.
